# True Lies: Using Proteomics to Assess the Accuracy of Transcriptome-Based Venomics in Centipedes Uncovers False Positives and Reveals Startling Intraspecific Variation in *Scolopendra subspinipes*

**DOI:** 10.3390/toxins10030096

**Published:** 2018-02-28

**Authors:** Jennifer J. Smith, Eivind A. B. Undheim

**Affiliations:** 1 Institute for Molecular Bioscience, University of Queensland, Brisbane 4072, Australia; jennifer.smith@imb.uq.edu.au; 2 Centre for Advanced Imaging, University of Queensland, Brisbane 4072, Australia

**Keywords:** centipede, *Scolopendra subspinipes*, venom, intraspecific variation, toxin annotation, transcriptomics, proteomics

## Abstract

Centipede venoms have emerged as a rich source of novel bioactive compounds. However, most centipede species are commonly considered too small for venom extraction and transcriptomics is likely to be an attractive way of probing the molecular diversity of these venoms. Examining the venom composition of *Scolopendra subspinipes*, we test the accuracy of this approach. We compared the proteomically determined venom profile with four common toxin transcriptomic toxin annotation approaches: BLAST search against toxins in UniProt, lineage-specific toxins, or species-specific toxins and comparative expression analyses of venom and non-venom producing tissues. This demonstrated that even toxin annotation based on lineage-specific homology searches is prone to substantial errors compared to a proteomic approach. However, combined comparative transcriptomics and phylogenetic analysis of putative toxin families substantially improves annotation accuracy. Furthermore, comparison of the venom composition of *S. subspinipes* with the closely related *S. subspinipes mutilans* revealed a surprising lack of overlap. This first insight into the intraspecific venom variability of centipedes contrasts the sequence conservation expected from previous findings that centipede toxins evolve under strong negative selection. Our results highlight the importance of proteomic data in studies of even comparably well-characterized venoms and warrants caution when sourcing venom from centipedes of unknown origin.

## 1. Introduction

Animal venoms contain a plethora of bioactive peptides and proteins, many of which have the potential for application as pharmacological tools [1] and in some cases lead molecules for the development of novel therapeutics [2] or agrochemical products [3]. As such, there is great interest in the field of venom research to investigate the diversity of putative toxins in venomous organisms. One method of probing this molecular diversity is through transcriptomic analysis of venom glands [4]. Over the past ten years, DNA sequencing costs have dramatically decreased and the technologies required to generate transcriptomes are now widely accessible. Consequently, venom gland transcriptomes have been acquired for over 100 species worldwide, including snakes, scorpions, sea anemones and spiders.

A group of venomous animals that are attracting increasing research interest are the centipedes. Centipedes are highly successful terrestrial arthropods, with approximately 3500 extant species and a fossil record dating back over 400 million years [5]. Although they are renowned for delivering a painful sting through modified walking legs termed forcipules, efforts to characterize the molecular components of their venom have only been recently begun. Current proteomic and transcriptomic research has revealed the presence of numerous disulphide rich peptides, many of which have novel or rare scaffolds [6]. An example is the SLPTX03 family of centipede peptides that adopt a remarkable ultra-stable three-dimensional structure comprised of four alpha helices [6]. Centipede venoms have also been shown to contain toxins with diverse and unusual pharmacological activities [7]. These toxins affect a range of molecular targets, including voltage-gated sodium, potassium and calcium (Na_V_, K_V_ and Ca_V_) channels [8,9]. An unusual mode of action has been found for the toxin SSD609, which indirectly inhibits K_V_7.1 through interaction with its auxillary subunit [10]. The presence of peptides with unique three-dimensional structures and interesting pharmacologies make centipede venoms a rich and vastly untapped source of molecular tools and potential drug leads [7].

One potential reason for the lack of existing research on centipede venom is that most centipede species are considered too small to obtain sufficient venom for activity testing or proteomic analyses. Venom gland transcriptomics provides a way around this issue by requiring just a few nanograms of RNA to generate a near complete library of all expressed toxin genes. Once annotated, this list of toxin transcripts can then be used for downstream applications such as evolutionary analyses or to generate libraries of synthetic toxins available for pharmacological screening [4,11,12]. However, recent studies have shown that this toxin annotation is anything but trivial, even in relatively well-characterized venomous lineages and can result in both a large number of false positives and lack of identification of novel toxin families [13,14]. In addition to an erroneous overall picture of venom composition, the misidentification of physiological non-venom proteins and peptides as toxins distorts our ability to study the evolution of toxins and identify adaptive traits that underlie their remarkable structural and pharmacological properties [15].

**Figure 1:**
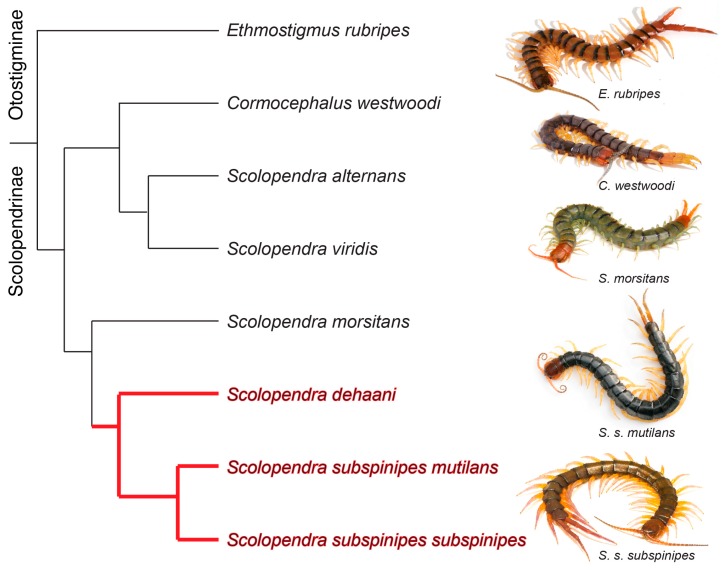
Representative phylogeny of scolopendrid centipedes investigated to date. Topology is adapted from recent molecular phylogenetic studies [17,18] and clade representing the *S. subspinipes* complex is highlighted in red. *S. s. mutilans* photo source: Yasunori Koide, Wikimedia Commons.

Here, we test the reliability of transcriptomics for the study of centipede venoms, comparing the results of common homology and comparative expression approaches to proteomics-based toxin annotation. For our model species, we examine pooled venom from a West Javanese population of the giant centipede *Scolopendra subspinipes*. *S. subspinipes* is one of the most commonly encountered species of giant centipedes in South East Asia and several other tropical locations around the world [16], as well as globally in the pet trade. Venom from this species is also among the best characterized of any centipede, with the vast majority of bioactive centipede toxins having been described from *S. subspinipes mutilans*, recently synonymized with *S. subspinipes* and *S. dehaani,* recently removed from *S. subspinipes* ([6,16,17,18] but see [19]) (Figure 1). These datasets allowed us to not only test the effectiveness of homology searches within a range of organismal relatedness but also provide the first insight into the intraspecific venom variation of any centipede. We show that even careful transcriptomic toxin annotation based on lineage-specific homology searches is prone to substantial errors in toxin annotation but that comparative expression combined with phylogenetic analyses can improve annotation accuracy. Moreover, our data reveal a surprising degree of toxin heterogeneity within *S. subspinipes*, which contrasts the sequence conservation expected from previous findings that centipede toxins evolve under strong negative selection [20].

## 2. Results

### 2.1. Transcriptome Generation

To examine the full suite of toxins expressed in the venom gland of *S. subspinipes*, we sequenced polyA-enriched RNA extracted from regenerating venom glands and non-adjacent forcipular muscle tissue using the Illumina NextSeq platform. This yielded a total of 32,427,576 and 47,484,467 paired reads for venom gland (vg) and muscle, respectively, which were trimmed according to a stringent minimum quality of 30 and minimum length of 120 bp. Paired trimmed reads (vg: 5,168,876; muscle 7,054,216) from both samples were pooled and de novo assembled using Trinity, resulting in 69,964 contigs with an n50 of 1120 bp and mean length of 723.16 bp. The contigs were translated to all possible coding sequences, resulting in 284,992 amino acid sequences, which we then used for subsequent analyses of venom components.

### 2.2. Venom Composition of S. subspinipes

**Figure 2:**
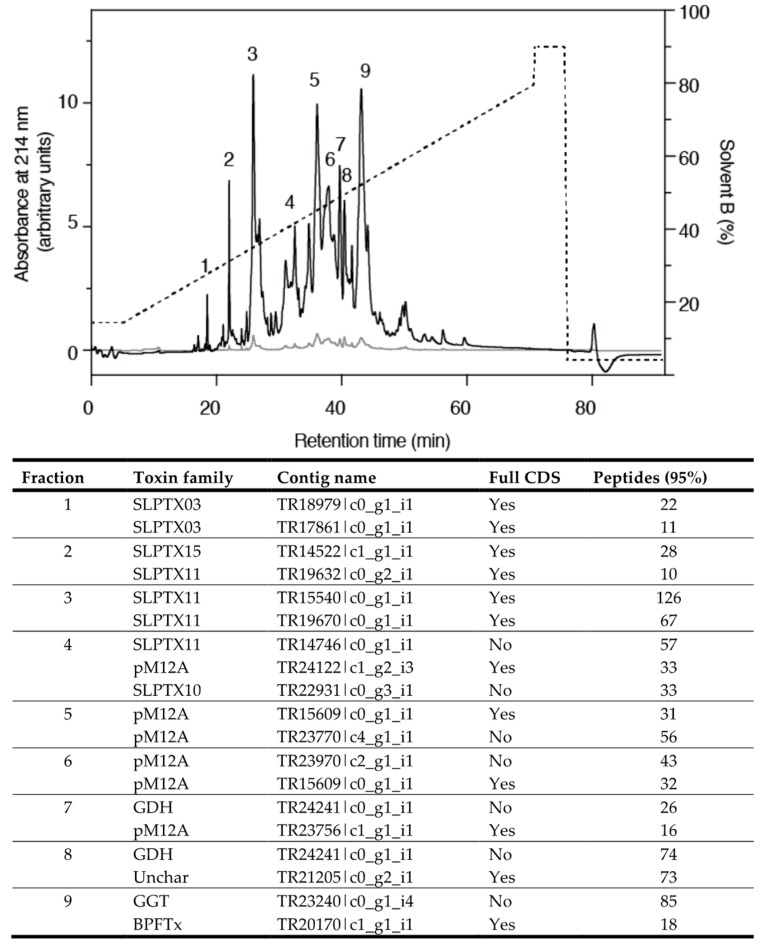
HPLC trace of crude *S. s. subspinipes* venom. Black and grey traces show absorbance at 214 nm and 280 nm, respectively. Main peaks are labelled 1–9 and the predominant peptides and proteins listed in the table (Full lists can be found in Tables S3–S11).

Although the venom of *S. subspinipes* has been previously studied by both activity-based fractionation [8] and venom peptidomics [21], these studies concerned *S. subspinipes mutilans* (henceforth *S. s. mutilans* for clarity) sourced from Chinese populations. In contrast, our specimens were originally collected in West Java (6°47′41.4″ S 106°36′49.5″ E) and were identified by a taxonomic expert (Dr. Warut Siriwut, Chulalongkorn University, Bangkok, Thailand) as a member of the previously recognized subspecies *S. subspinipes subspinipes* (henceforth *S. s. subspinipes* for clarity). We therefore decided to determine the venom profile of *S. s. subspinipes* by a combined proteomic and transcriptomic approach and used tandem MS-based shotgun proteomics to analyse venom collected from the same specimens used for transcriptome sequencing. In line with previous studies, we also gave emphasis to peptide components of the venom and therefore analysed both crude venom and venom pre-fractionated using reverse-phase HPLC (Figure 2). This resulted in identification of 47 protein and peptide families containing a total of 248 unique CDSs (Table S1), of which 86 encoded peptide toxins. Interestingly, searching MS/MS data pooled from all samples returned only 183 unique CDSs, suggesting the prefractionation of similar toxin isoforms is important for the identification of an as complete venom profile as possible (Table S2).

Of the peptide toxins found proteomically in *S. s. subspinipes* venom, the most abundant and diverse belong to the SLPTX11 and SLPTX15 families, with 14 and 16 CDSs detected in the proteome, respectively. Both are cysteine-rich peptide families, with members of SLPTX15 containing 4 cysteines and SLPTX11 ranging from 4 to 16 cysteines. SLPTX15 toxins have been shown to have undergone functional radiation and members have been shown to target a range of voltage-gated ion channels, namely K_V_, Ca_V_ and Na_V_ channels [22]. The SLPTX11 family exhibits interesting structural variability via N- and C-terminal truncations [22]. Functionally, a handful of SLPTX11 toxins have been found to have anticoagulant properties and inhibit K_V_ channels, however further experiments are needed to fully explore the molecular targets of toxins in this family and determine whether structural variants present different pharmacologies.

The most abundant and diverse protein families present in the venom of *S. s. subspinipes* were the putative metalloendoproteases pM12A, the β-pore-forming toxins (β-PFTx) and proteins containing a low-density lipoprotein receptor Class A repeat (LDLA) domain, with 24, 36 and 28 proteomically detected CDSs, respectively. Metalloproteases have been shown to be an important component in centipede venoms and may play a role in tissue damage that assists toxin diffusion during envenomation [5,23,24]. The β-PFTx are believed to contribute to the highly cytolytic, myotoxic and edematogenic properties of centipede venoms and are likely to be involved in prey capture and predator deterrence [22,24]. LDLA-containing proteins are also highly abundant in the venom from other centipede species and thus probably have an important function, however their role in centipede venoms is yet to be determined [22,24]. Other abundant yet less diverse proteins present in *S. s. subspinipes* venom included GDH and CO-Esterase B, of which the biological function in centipede venoms has not been elucidated for either protein class.

It is also interesting to note that this is the first time all three types of centipede CAP proteins have been found together in the venom of a single species, as this has implications for their evolutionary histories as centipede venom components. It is the first time CAP type 1 has been detected in venom from Scolopendrinae, showing this type has not been secondarily lost in this subfamily and supporting a single, early origin as a venom component in centipedes [22]. Moreover, CAP type 3 was previously hypothesized to be of relatively recent origin in centipedes, having been recruited in a clade containing *S. morsitans* but excluding *S. dehaani*. However, our detection of this toxin family in *S. s. subspinipes* brings its recruitment close to the base of the genus *Scolopendra* [16] and shows it is more widespread than previously assumed.

Among the identified venom components, 21 represented protein and peptide families previously not described from centipede venoms (Table 1). In addition to a number of proteins with no recognizable domains or similarity to characterized proteins, the novel families include one new cysteine-rich structural scaffold (SLPTX29), one cysteine-rich scaffold that was previously erroneously assigned to SLPTX17 (SLPTX30) [21] and one non-disulphide crosslinked peptide (SLPTX31). Although their function in the venom remains unknown, it is interesting to note that the cysteine-rich peptide scaffold of SLPTX29 has also been found in the venom of the remipede *Xibalbanus tumulensis* (Xibalbin 7) [14]. Convergence to other venomous lineages is also apparent in the presence of C-type lectin-like proteins, which are found as a potent pro-inflammatory toxin the Brazilian toadfish *Thalassophryne* [25] and as functionally diverse hemotoxins in snake venoms [26]. Similarly, a procoagulant cathepsin L-type cysteine-protease is also released by the parasitic helminth *Fasciola hepatica* [27], while icarapin is one of several allergens found in bee venom [28].

### 2.3. Identifying Toxins by BLAST Annotation

Having proteomically determined the venom profile of *S. s. subspinipes*, we next set out to compare this profile to lists of putative components predicted by BLAST annotation. As sequence databases for our BLAST searches we used all annotated toxin sequences in UniProtKB (ToxProt), a compiled list of all centipede toxins and putative toxins (lineage-specific) and finally the proteomic dataset as a representative of a within-species dataset (species-specific). Interrogating the transcriptomic dataset using either of the BLAST databases returned a much greater number of putative toxins than by proteomics alone (Table 2). Interestingly, while the proportion of unique contigs to unique translated CDS sequences was relatively constant across all four datasets (82–87%), the proportion of these sequences containing a signal peptide was almost half among the ToxProt identified sequences (26%) compared to the three centipede-specific datasets (40–49%). Annotation by ToxProt thus resulted in a much more fragmented putative toxin sequence dataset consisting of more partial contigs and fewer highly expressed, well-assembled contigs.

**Figure 3:**
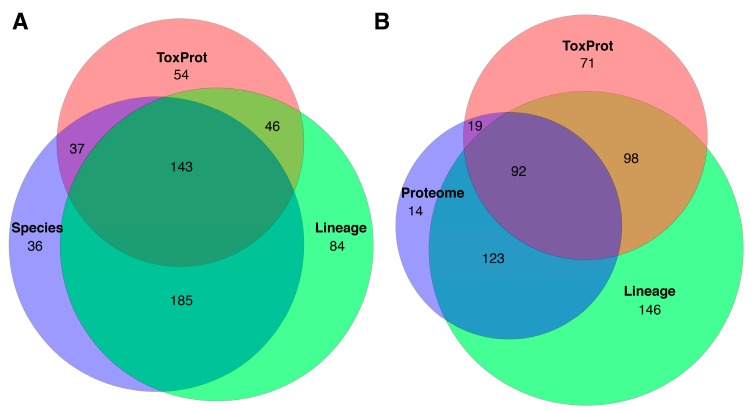
Proportional Venn diagrams of putative toxins identified by BLAST search against all annotated toxins in UniProtKB (ToxProt), BLAST search against all publicly available centipede toxin sequences (Lineage) and either (**A**) BLAST search against all proteomically identified sequences (Species) or (**B**) proteomically identified sequences (Proteome).

Although the numbers of putative toxins identified by BLAST were all greater than that detected proteomically, there were substantial differences in the putative toxins predicted by each BLAST method (Figure 3A). Almost 45% of the unique CDSs with predicted signal peptide identified by ToxProt were not recovered by either of the lineage- or species-specific BLAST annotations, demonstrating the dramatic false positive rate and overestimation of evolutionary convergence between venomous lineages that results from this approach [13]. However, we also found the lineage specific toxin BLAST dataset to contain a surprisingly high proportion (almost 30%) of sequences that were not identified by species-specific BLAST annotation. Comparing the BLAST predicted toxins to the venom proteome of *S. subspinipes* reveals even greater false positive rates for both the ToxProt (60.3%) and lineage-specific (53.3%) BLAST datasets Figure 3B). Additionally, 38.2% of all putative toxins in the species-specific BLAST dataset were not detected in the proteome, suggesting a large portion of this overestimated toxin diversity is due to the inclusion of non-toxin homologues. Thus, BLAST-based toxin annotation of centipede transcriptomes is highly likely to substantially overestimate the number of toxins actually present in the venom, which may bias the inference of toxin convergence and is likely to result in inaccurate estimations of influences of natural selection.

**Figure 4:**
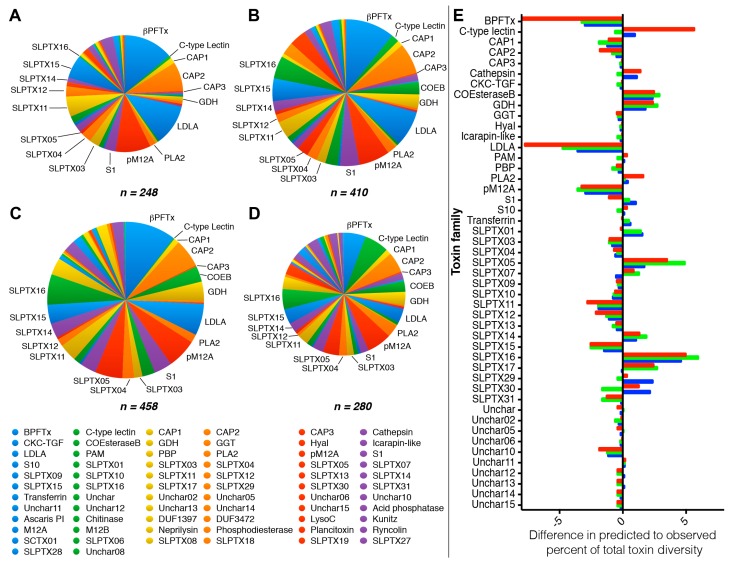
Proportional pie charts of toxin families identified in the transcriptome of *S. s. subspinipes* by (**A**) proteomic analysis of venom; (**B**) BLAST search of transcriptome against all proteomically identified sequences (species-specific); (**C**) BLAST search against all publicly available centipede toxin sequences (lineage-specific); and (**D**) BLAST search against all annotated toxins in UniProtKB (ToxProt); The differences in the relative diversity of each family as identified by BLAST is shown in (**E**). Bars show the change in the percent of all CDSs identified by ToxProt (red), lineage-specific (green) or species-specific (blue) BLAST annotation compared to the venom proteome. Abbreviations: Putative β-pore-forming toxin (βPFTx); Cystine-knot cytokine domain TGF-beta-like peptide (CKC-TGF); Cysteine-rich, allergen and pathogenesis-related protein (CAP); Carboxyl-esterase type B (COEsteraseB); Protein containing a domain of unknown function type 1397 (DUF1397); Protein containing a domain of unknown function type 3472 (DUF3472); Glucose dehydrogenase (GDH); γ-glutamyl transferase (GGT), Hyaluronidase (Hyal); Low-density lipoprotein receptor Class A repeat domain containing protein (LDLA); Lysosome C (LysoC); Peptidylglycine alpha-hydroxylating monooxygenase (PAM); Phosphatidylethanolamine-binding protein (PBP); Putative M12A type protease (pM12A); Phospholipase type A_2_ (PLA2); Type 1 Serine protease (S1).

Given the large number of false positives returned by all BLAST annotation approaches, we next examined the effect this had on the ability of BLAST toxin annotation to predict general venom composition as estimated by relative toxin family diversity (Figure 4). As expected, ToxProt annotation resulted in substantially different predicted venom composition, including a severe underestimation of the five most diverse toxin families of *S. s. subspinipes* venom (βPFTx, pM12A, LDLA, SLPTX11 and SLPTX15). However, we also found similar results for the lineage- and species-specific BLAST datasets, which underestimated the relative diversity of pM12A, LDLA, SLPTX11 and SLPTX15 by up 42%. Conversely, the relative diversities of SLPTX16 and CO-Esterase B were the most overestimated by all BLAST approaches, which predicted about 8 to 16-fold and 4 to 10-fold greater relative diversity, respectively. Even species- or lineage-specific BLAST annotation therefore appears to be by itself unreliable as a tool to estimate venom composition and toxin family diversity.

### 2.4. Identifying Putative Toxins by Comparative Transcriptomics

In addition to BLAST annotation, comparative transcriptome analyses of venom glands and non-venom producing tissues has been used as a method to distinguish putative toxins from non-toxin homologues [4,15,29,30]. Examining separately assembled muscle and venom gland transcriptomes revealed that the majority of putative toxins, including about 80% of those proteomically identified in the venom, were present in both transcriptomes (Supplementary Files S1 and S2). This was further supported by comparative expression analyses of reads mapped to the pooled transcriptome assembly (Table S1). While there was a significant correlation between expression levels of contigs in the venom gland and muscle (*p* < 0.0001, Spearman r 0.584), contigs that were not detected in the muscle did not necessarily show low expression in the venom gland (Table S1). For example, among contigs unique to the venom gland, one was ranked the 21st most highly expressed venom components (FPKM 7486), another three were among the top 55, while about 25% showed low expression (FPKM < 10). Direct comparisons of venom and non-venom producing tissues to identify toxins therefore appears not to be a good strategy for identifying putative toxins in transcriptome datasets.

**Figure 5:**
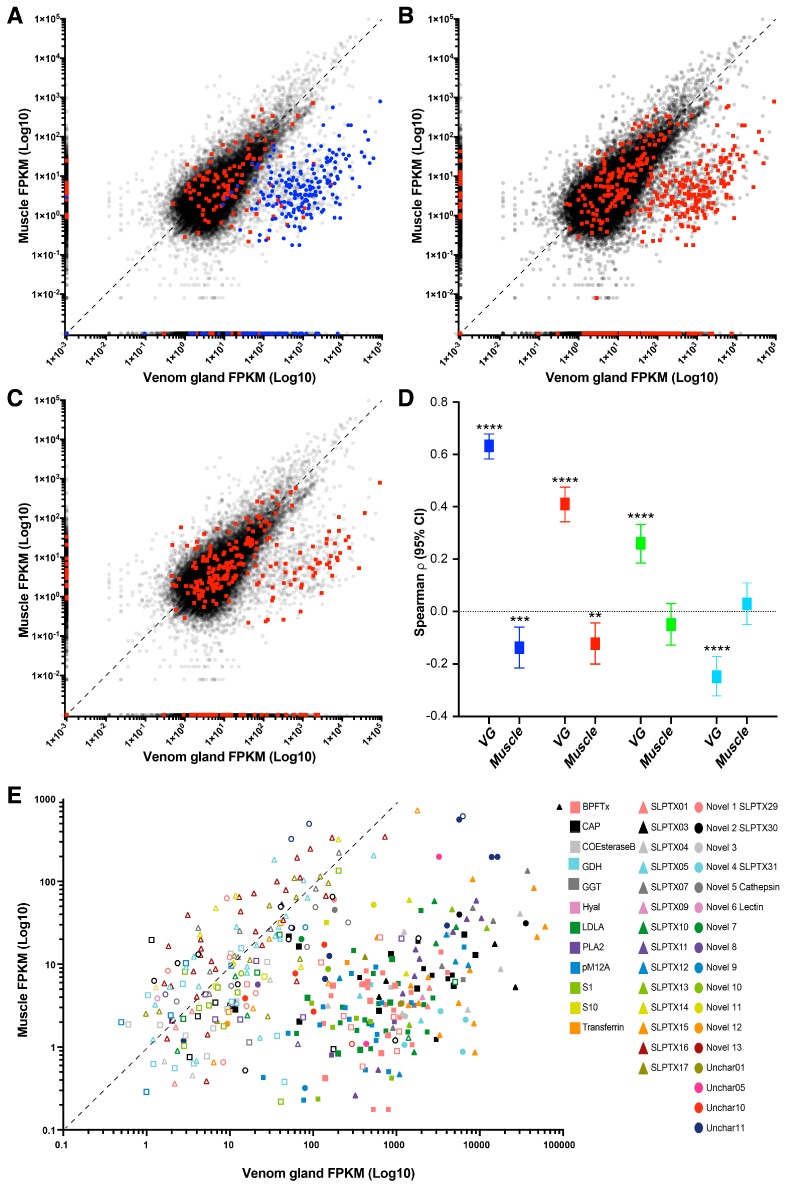
Correlation between toxin annotation and expression level in the venom gland and muscle tissue of *S. s. subspinipes.* Plots of expression values calculated as reads per kilobase million (FPKM) are shown for (**A**) contigs identified by proteomics (blue) and species-specific BLAST (red); (**B**) lineage specific BLAST; or (**C**) ToxProt BLAST. Expression estimates for all other contigs are shown as grey circles and the 1:1 ratio between the two tissues is indicated by the dashed line; (**D**) Spearman correlation and two-tailed significance test between detection by proteomics (blue), species-specific BLAST (cyan), lineage-specific BLAST (green), or ToxProt BLAST (red) and expression in venom gland (vg) or muscle. **** denotes *p* < 0.0001, *** indicates *p* < 0.001, while ** indicates *p* < 0.005; (**E**) Family partitioned plot of expression values for contigs identified by species-specific BLAST (open symbols) and contigs detected by venom proteomics (filled symbols).

Although a presence/absence comparison of transcripts in venom gland and muscle tissues is an ineffective way of identifying putative toxins, it is intuitive that toxin encoding genes should be upregulated in response to depletion of venom from the venom gland. Indeed, we found significant positive correlations between detection in proteome and expression in the venom gland but not muscle tissue (Figure 5). This was also the case for contigs identified by species- and lineage-specific BLAST annotation, although with lower correlation values. In contrast, contigs identified by ToxProt BLAST showed a significant negative correlation with expression level in the venom gland. This observation supports the notion that less related sequences are less useful in identifying putative toxin sequences but also supports previous findings suggesting that relative expression level may be a useful approach to evaluate whether or not a CDS represents a toxin or a homologous non-toxin [15].

**Figure 6:**
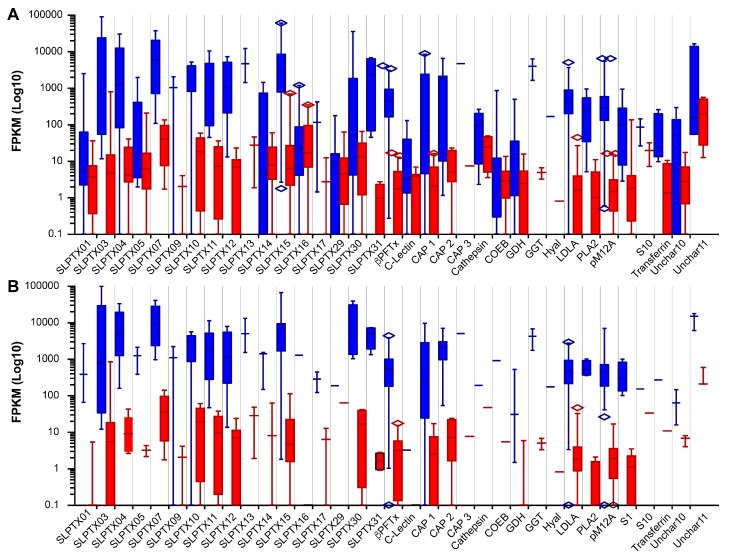
Expression levels of each toxin family in venom gland (blue) and muscle (red) shown as box and whiskers plot of (**A**) all sequences identified by species specific BLAST or (**B**) only sequences identified in the venom proteome. Whiskers indicate 5–95 confidence intervals. Abbreviations: Putative β-pore-forming toxin (βPFTx); Cysteine-rich, allergen and pathogenesis-related protein (CAP); Carboxyl-esterase type B (COEB); Protein containing a domain of unknown function type 1397 (DUF1397); Protein containing a domain of unknown function type 3472 (DUF3472); Glucose dehydrogenase (GDH); γ-glutamyl transferase (GGT), Hyaluronidase (Hyal); Low-density lipoprotein receptor Class A repeat domain containing protein (LDLA); Lysosome C (LysoC); Putative M12A type protease (pM12A); Phospholipase type A_2_ (PLA2); Type 1 Serine protease (S1).

To further test relative gene expression as a way to refine BLAST generated lists of putative toxins, we examined the expression levels of contigs identified by proteomics and species-specific BLAST for each centipede toxin family (Figure 6). This revealed that most toxin families identified by species-specific BLAST show a clear trend of having higher expression values in the venom gland compared to muscle tissue (Figure 6A). Overexpression in the venom gland was even more apparent among the proteomically confirmed venom components (Figure 6B), suggesting that both expression level and relative expression in the venom gland compared to non-venom producing tissue may be useful in filtering non-toxin homologues.

**Figure 7:**
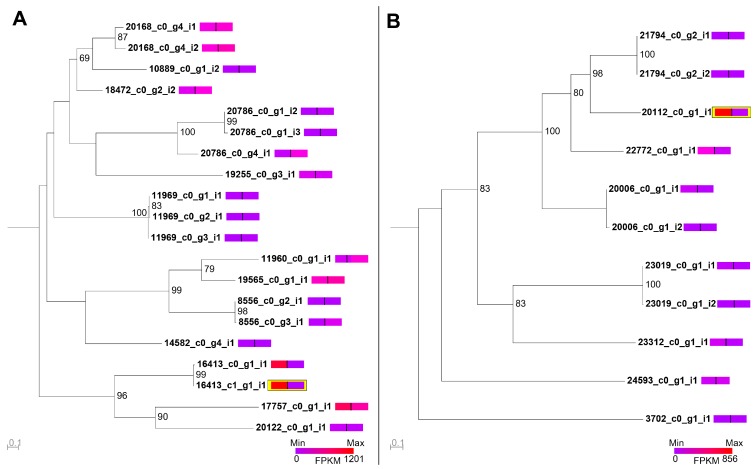
Expression levels and presence/absence in venom of sequences in *S. s. subspinipes* venom, mapped onto maximum likelihood (ML) bootstrapping consensus trees of all (**A**) SLPTX16 and (**B**) carboxylesterase Type B sequences detected in the combined transcriptome. Both trees are displayed as midpoint rooted, normalised expression levels (FPKM) are shown as heat maps in coloured boxes divided in half for venom gland (left) or muscle (right) and expression boxes of the sequences detected in venom by proteomics are highlighted in yellow. Horizontal bars indicate genetic distance calculated under the (**A**) VT + I + G4 or (**B**) LG + I + G4 model of amino acid substitution rates selected by ModelFinder.

While additional work is required to find optimal filtering conditions for refining BLAST-based toxin annotations, we tested the general effectiveness of this approach using parameters approximated based on the above analyses. Indeed, using a cut-off value of 100 FPKM and a minimum of 10-fold overexpression in the venom gland retained 80.2% of all proteomically identified sequences but substantially reduced the mismatch between the venom proteome and three BLAST datasets. Filtering the ToxProt, lineage-specific and species-specific BLAST datasets using these conditions yielded a total of 73, 216 and 224 sequences, respectively, of which 76.7%, 80.1% and 88.8% were also identified in the venom proteome. These figures are marked improvements from the unfiltered BLAST datasets, which differed from the proteomic data by approximately 40–60%. Additionally, only 8 of the 73 putative toxins in the filtered ToxProt BLAST dataset were not found in the venom proteome or either of the centipede datasets. Moreover, comparative expression effectively reduced the mismatch within toxin families, such as in the two families whose diversities were the most overestimated by all BLAST approaches (SLPTX16 and carboxylesterase Type B; Figure 4 and Figure 7). This suggests relative expression as an additional criterion for toxin annotation is an effective means of limiting overestimation of toxin convergence between venomous lineages.

**Figure 8:**
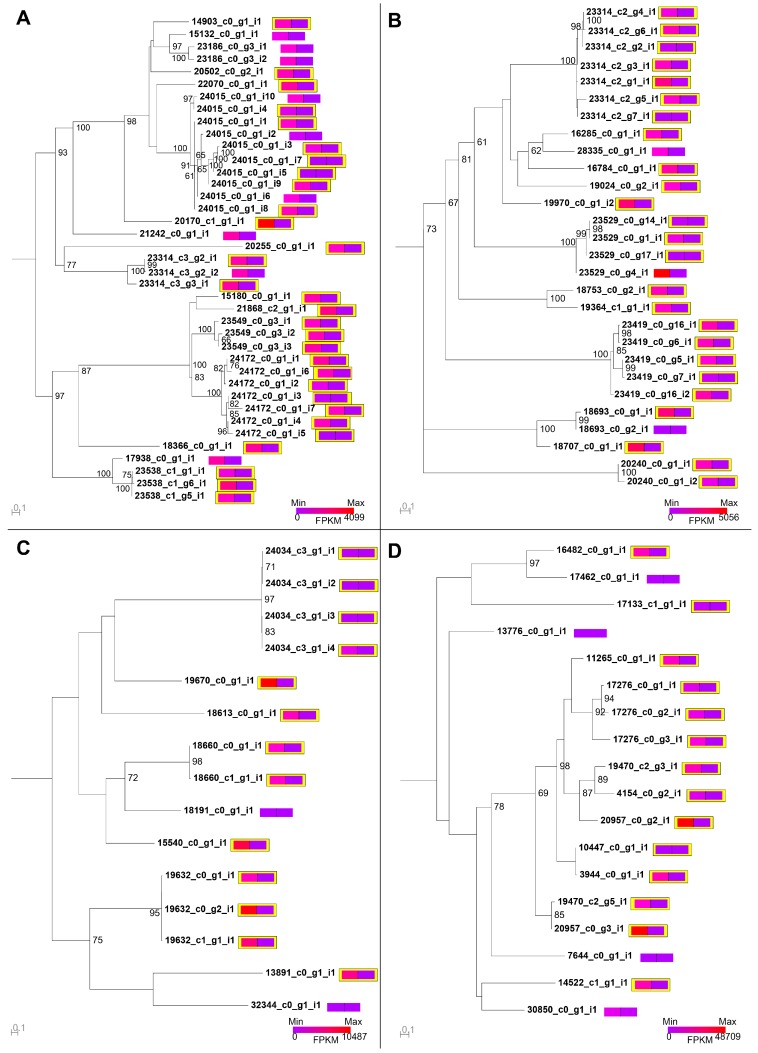
Expression levels and presence/absence in venom of sequences from four of the most diverse toxin families in *S. s. subspinipes* mapped onto maximum likelihood (ML) bootstrapping consensus trees of all (**A**) βPFTx; (**B**) LDLA; (**C**) SLPTX11; or (**D**) SLPTX15 identified in the combined muscle and venom gland transcriptome. All trees are displayed as midpoint rooted, normalised expression levels (FPKM) are shown as heat maps in coloured boxes divided in half for venom gland (left) or muscle (right) and expression boxes of the sequences detected in venom by proteomics are highlighted in yellow. Horizontal bars indicate genetic distance calculated under the (**A**) WAG + F + R4; (**B**) VT + R3; (**C**) FLU + F + R3; or (**D**) VT + I + G4 model of amino acid substitution rates selected by ModelFinder.

Although filtering on relative expression results in a reduction of false positives, there remains a large discrepancy between the venom proteome and the species- and lineage-specific BLAST data. To investigate whether this is mainly due to the identification of non-toxin homologues or untranslated toxin isoforms, we mapped expression values and proteomic detection onto phylogenetic reconstructions of all toxin families detected in *S. s. subspinipes* venom (Supplementary File S3 and S4). As exemplified by the four most diverse toxin families in *S. s. subspinipes* this strategy revealed that virtually all clades containing highly upregulated contigs in the venom gland also contained representatives that were detected in the venom (Figure 8). However, proteomically detected sequences were not consistently the most highly upregulated contigs within these clades. This suggests filtering BLAST annotations by expression levels is likely to be effective at removing non-toxin homologues but not necessarily at providing an accurate estimate of translated toxin diversity. In contrast, combined comparative expression and phylogenetic analyses of toxin families identifies these variably expressed toxin isoforms while filtering out likely non-toxin clades.

The inflated toxin diversity estimates by species specific BLAST annotation and discrepancy between the expression level and proteomic detection of putative toxins also begs the question as to the underlying causes. While this could be due to a number of different reasons, these either relate to inherent weaknesses of the proteomic analyses or the accuracy of the transcriptome assembly. Proteomic shortfalls include differences of various venom components in sample solubility or effectiveness of ionization and is very likely to lead to the missed identification of some venom components, as indicated by the higher number of identifications obtained by prefractionating the venom (Section 2.2). Transcriptome-related factors include an inflated number of contigs resulting from the sequencing and assembly processes, which result in inflated contig diversity. This inflated contig diversity also results in a more fragmented assembly, which can affect the ability to detect toxins by the spectral matching approach used here because of missed CDS prediction (CDS fragment is shorter than the minimum length) or because the CDS fragment lacks the region encoding the mature peptide/protein.

Distinguishing between these effects is difficult, or even impossible, without genomic data. However, to get an indication of whether the mismatch in the observed and predicted number of toxins is more likely to be primarily due to fragmented contigs or inflated contig numbers, we examined the proportion of incomplete annotated contigs with predicted signal peptide. Because we annotated predicted CDSs, not nucleotide sequences, a strong effect from CDS fragmentation would result in a skewed distribution of the proportion of partial CDSs between the proteomically identified (more complete) and BLAST predicted datasets (more incomplete). This is not the case, however, with complete CDSs constituting 33% of all unique CDSs identified by both proteomics and species-specific BLAST (Table 2).

Another indication of an effect of CDS fragmentation is that among CDSs containing a signal peptide one would also expect the difference in toxin diversity estimates to be correlated with sequence length. This is because peptide CDS fragments with predicted signal peptides are more likely to lack the mature peptide that is observable by MS. Indeed, examining the overestimation of toxin diversity by BLAST revealed that, after removing the two longest peptide families (SLPTX11 and 16), 43.7% of peptides and 56.3% of proteins annotated by species-specific BLAST were also identified by proteomics. Examining the proportion of complete CDSs among peptides and proteins also revealed that peptides identified by proteomics consisted of more complete CDSs than those only predicted by BLAST (88.9% vs. 81.5%), while proteins identified only by BLAST consisted of more complete CDSs than those identified by proteomics (66.7% vs. 49.4%).

Although these data are by no means definitive in terms of determining the underlying causes of the mismatch in predicted and observed toxin diversity in venom gland transcriptomes, they do suggest that both CDS fragmentation and contig diversity inflation are contributing factors. There are, however, many other factors that influence the distribution of incomplete CDSs, such as expression level and gene family diversity. Without the genomic data required to assess the accuracy of the assembly, we are also unable to distinguishing these experimental effects from biological factors, such as gene silencing by antisense RNAs. It will be interesting to see future studies dissect the contributions of these experimental and biological factors to the commonly observed discrepancy between proteomic and transcriptomic venom diversity.

### 2.5. Intraspecific Variation in the Venom of S. subspinipes

Because *S. subspinipes* is both one of the largest and globally the most commonly available centipede species, it is likely to be an important source of centipede venom derived bioactive compounds. We therefore wanted to investigate the potential variability between differently sourced specimens in order to assess both the expected reproducibility of crude venom experiments and the ease of which toxins of interest can be sourced, for example from specimens purchased from the pet trade. Given the peptidomic focus of previous studies on *S. s. mutilans*, we compared all published peptide toxins to our identified peptides, using the full species-specific BLAST database in order to minimize effects from intra-population variability. Clustering by CD-HIT after removal of signal peptide domains using SignalP revealed that the two populations of *S. subspinipes* do not share a single peptide toxin with greater than 95% sequence similarity. Moreover, there were only two clusters of peptides with greater than 90% sequence similarity, one belonging to SLPTX10 (TR18770|c2_g11_i1, TR18770|c2_g7_i1 and SLPTX10-Ssm1b [JZ722869]) and one belonging to SLPTX04 (TR7755|c0_g1_i1 and SLPTX04-Ssm3a [JZ722849]).

**Figure 9:**
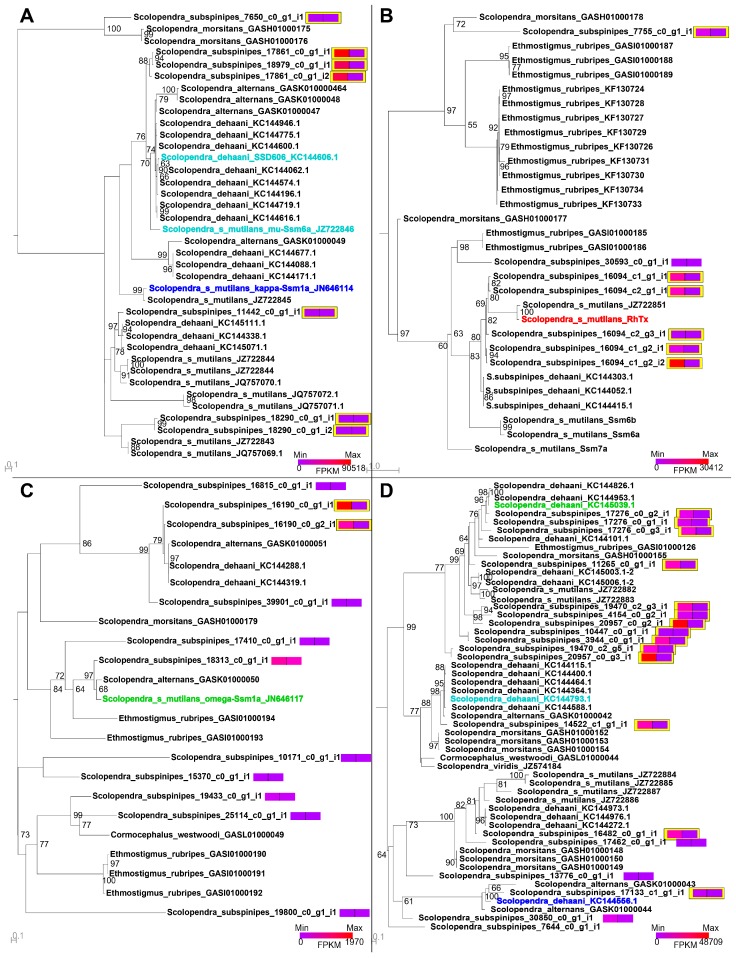
Maximum likelihood phylogenetic reconstructions of members of (**A**) SLPTX03, (**B**) SLPTX04, (**C**) SLPTX05 and (**D**) SLPTX15, with previously described toxins acting on Na_V_ (cyan), K_V_ (blue), Ca_V_ (green), or TRPV1 (red) are shown in bold. Normalized expression levels (FPKM) are shown as heat maps in coloured boxes divided in half for venom gland (left) or muscle (right) and highlighted in yellow for sequences detected in the venom. Bootstrapping consensus trees are shown as mid-point rooted, while horizontal bars indicate genetic distance calculated under the (**A**) JTT + R2, (**B**) VT + G4, (**C**) FLU + R3, or (**D**) VT + I + G4 model of amino acid substitution rates selected by ModelFinder.

The surprising degree of heterogeneity between the two populations prompted us to further examine the intraspecific variability of *S. subspinipes* venom. We therefore examined the phylogenetic distribution of *S. subspinipes* sequences within four toxin families containing known bioactive peptides (SLPTX03, SLPTX04, SLPTX05, SLPTX15), using all other published centipede sequences as outgroups (Figure 9). Interestingly, the phylogenetic analyses revealed that the venom of *S. s. subspinipes* does not contain any toxins in SLPTX03–05 that cluster with previously described bioactive toxins from either *S. s. mutilans* or *S. dehaani.* Moreover, the only sequence from *S. s. subspinipes* that clustered with a previously described active toxin from *S. s. mutilans* (ω-SLPTX-Sm1a) was highly expressed in both venom gland and muscle and not detected in the venom, suggesting it serves a non-toxin function outside the venom.

Unlike toxins in families SLPTX03–05, *S. s. subspinipes* did contain multiple proteomically detected and/or venom gland upregulated sequences from SLPTX15 that clustered with known ion channel modulators (Figure 9D). However, all these ion channel toxins were described from *S. dehaani*, rather than *S. s. mutilans*. In contrast, no SLPTX15 sequences from *S. s. mutilans* clustered with any pharmacologically characterized toxins. Furthermore, most *S. s. subspinipes and S. s. mutilans* sequences did not cluster together but instead formed paraphyletic clades that were either interleaved by toxins from other *Scolopendra* species or lacked one of the two subspecies altogether. A similar trend was also evident in most other SLPTX families (Supplementary File S5 and S6), suggesting there is substantial intraspecific variation in venom composition of *S. subspinipes*.

## 3. Discussion

Venom gland transcriptomes represent a cost-effective way of obtaining a large number of toxin sequences [4]. However, the approach does have its limitations, even beyond the lack of ability to detect novel toxin classes such as the 13 families detected here (Table 1) [31]. In addition to the difficulties of predicting the final translated and modified toxin products, even identifying which contigs encode toxins can be challenging [14]. Despite these issues, sequence homology-based searches of known toxins against transcriptomic data is a common strategy for identifying putative toxins, particularly in lineages where venom is difficult to obtain [14,29,32,33]. It is also arguably a useful method for extracting additional isoforms from the transcriptomic data that were not identified by proteomic methods e.g., due to sensitivity issues or intraspecific variability between the sources of the venom vs. transcriptomic data [22]. However, because toxins are derived from “every-day” physiological proteins that are also retrieved by sequence-homology searches of toxins, this approach can result in a large proportion of false positives, i.e., the incorrect annotation of non-toxin proteins as toxins [13,15].

Our results show that this is also the case for centipede venoms, where drastically different conclusions regarding toxin diversity and evolution can be drawn depending on the method used for identification of putative toxins. Indiscriminate inclusion of sequences identified by BLAST searches against toxin databases results in lists of “toxins” mostly populated by non-toxins or untranslated toxin homologues. These lead to inflated estimates of either toxin diversity (lineage- and species-specific toxin BLAST databases) or evolutionary convergence between venomous lineages (non-lineage-specific toxin BLAST database). Using comparative transcriptomics of venom producing and non-venom producing tissues can filter out many of these false positives [15]. However, in centipedes this approach can result in an underestimated toxin diversity (non-lineage-specific toxin BLAST database) and does not necessarily provide an accurate picture of which toxins are actually translated and present in the venom (lineage- and species-specific toxin BLAST databases).

Instead, comparative transcriptomics combined with phylogenetic analyses of putative toxin families appears to be the most accurate approach. Mapping proteomic and expression levels onto phylogenetic reconstructions of toxin families revealed that most of the “false positives” obtained by the species and lineage-specific BLAST searches were untranslated toxin homologues nested within clades of *bona fide* venom components, as opposed to non-toxin homologues. In fact, lineage and species-specific BLAST searches returned few, if any, putative ancestral non-toxin sequences in most families. Moreover, similar to previous findings in other venomous lineages [14,15,34], we found that most transcripts encoding proteomically identified venom components were expressed in both venom gland and muscle tissues. Thus, despite the high number of “false positives” as judged by our proteomic data, our results suggest most toxin-like sequences identified in both tissues were most likely toxins. These findings warrant caution when using non-venom producing tissues of a venomous species to identify putative ancestral sequences, without also including additional analyses of relative venom gland expression levels and/or proteomic analyses of venom.

Mapping proteomic and expression levels onto phylogenetic reconstructions of toxin families also revealed a striking lack of overlap between the venoms of *S. s. subspinipes* and *S. s. mutilans.* Contrary to our expectations, we found no toxins with greater than 95% sequence similarity shared between the two subspecies. With the exception of SLPTX04, we also did not find any contigs in *S. s. subspinipes* that clustered with bioactive toxins previously described from *S. s. mutilans*. Although several toxin contigs belonging to SLPTX15 clustered with known Na_V_, K_V_ and Ca_V_ neurotoxins, these were described from the venom of *S. dehaani*, which was recently removed from *S. subspinipes* [18].

This toxin heterogeneity observed within *S. subspinipes* venom is significant given the availability and size of this species, which suggests it is likely to be a common source of venom for the discovery of novel bioactive molecules. It also contrasts recent findings that centipede toxins evolve under extreme negative selection, which would imply strong conservation of toxins and a high overlap between populations/subspecies. Thus, it appears that rather than consisting of highly conserved and more or less uniform sets of toxins, the venom compositions of scolopendrid species vary significantly throughout their range. This not only raises questions regarding co-evolutionary forces acting on toxin genes but also highlight scolopendrid venoms as rich sources of novel bioactive peptides.

## 4. Conclusions

Comparative evolutionary studies hold the key to a better understanding of the fundamental principles that govern the functional and structural evolution of toxins. In order to accomplish this, more venomous lineages need to be examined. However, despite advances in analytical technology, many of these lineages remain too small or inaccessible for efficient venom extraction, leaving venomics by transcriptomics the only viable alternative for identification and characterization of toxins. Our results suggest BLAST searches against lineage-specific toxin families followed by combined comparative expression and phylogenetic analyses of toxin families may be the most effective approach for annotation of toxins in transcriptomic datasets. This approach maximizes the identification of putative toxin isoforms while avoiding inaccurate annotation of non-toxins as toxins. Ultimately, minimizing these inaccuracies maximizes our ability to accurately reconstruct and interpret the molecular evolution of toxins and identify the adaptive traits that can guide molecular engineering efforts and facilitate their application as molecular tools and potential development into therapeutics and biopesticides. 

## 5. Materials and Methods

### 5.1. Venom Collection and Transcriptome Sequencing

Venom was obtained by electrostimulation as described previously [6,22] from five specimens of *S. subspinipes* purchased from Zoohaus-WS, Ludwigshafen, Germany. The specimens were collected from west Java and the species identification confirmed by personal examination by Dr. Siriwut at the Natural History Museum, London, UK and Chulalongkorn University, Bangkok, Thailand. Collected venom was pooled and kept at −20 °C before it was lyophilized prior to transport to Australia, where it was stored at −80 °C until further analysis. Whole forcipules were removed three days after venom extraction and stored in RNAlater (ThermoFisher, Waltham, MA, USA) at ambient temperature during transport to Australia and then −80 °C until RNA extraction. In order to compare the presence and expression levels of toxins between venom glands and non-venom producing tissue, total RNA was extracted from venom glands as well as non-adjacent forcipular muscle tissue by standard TRIzol protocol (ThermoFisher, Waltham, MA, USA) and enriched for polyA RNA using a DynaBeads Direct mRNA kit (ThermoFisher, Waltham, MA, USA). The two polyA enriched RNA samples were submitted to the University of Queensland Institute for Molecular Bioscience Sequencing Facility for library preparation and sequencing. Paired end libraries with 180 bp insert size was constructed using the Illumina TruSeq-3 Stranded mRNA kit and sequenced on an Illumina NextSeq using a 300 cycle (2 × 150 bp) Mid Output Run. Raw sequence data has been deposited in the NCBI SRA (SRP126801) under BioProject PRJNA422449.

### 5.2. Transcriptome Assembly and Analyses

The resulting reads were trimmed using Trimmomatic v0.35 [35] to remove adapter sequences and low-quality reads. Window function-based quality trimming was performed using a window size of 75 and a window quality of 30 and sequences with a resulting length of <100 bp after trimming were removed. After quality control, paired-end sequences from both libraries were de novo assembled into contigs by Trinity v2.0.6 [36] using default parameters.

Following assembly by Trinity, the trimmed paired reads from each tissue was mapped back to the assembly using bowtie v2.2.6 [37] and expression values estimated as fragments per kilobase million (FPKM) using RSEM v1.2.31 [38]. Although we lacked technical replicates and individual biological replicates, we considered our pooling of five specimens a sufficiently robust with respect to biological variability to examine large-scale differences in expression levels between the two tissues. This lack of biological and technical replicates is also common among venomic studies and our results are therefore representative of common practices in the field.

### 5.3. Venom Proteome Analyses

To examine the composition of the venom of *S. subspinipes,* we analysed venom obtained from the same specimens used for our transcriptome studies. 0.7 mg crude lyophilized venom was fractionated by reverse phase HPLC using a Phenomenex Aeris Peptide XB-C18 column (4.6 × 250 mm, 3.6 μm particle size). The venom was redissolved in 50% solvent B (90% *vol*/*vol* acetonitrile [ACN], 0.043% trifluoroacetic acid [TFA]) in solvent A (0.05% TFA), eluted across a gradient of 14–80% solvent B in solvent A over 66 min and fractions collected manually. Each fraction was dried by vacuum centrifugation, re-dissolved in 40 μL 50% ACN in 0.1% formic acid [FA] and analysed by MALDI-MS and LC-MS/MS. For MALDI-MS, equal amounts of sample and 7 mg/mL α-cyano-4-hydroxycinnamic acid (CHCA) in 60% ACN 0.1% FA were spotted using the dried droplet method and analysed on a 4700 Proteomics Analyser MALDI-TOF/TOF operated in positive reflectron mode.

For LC-MS/MS, 5 µL from each fraction and 5 µg crude venom was reduced and alkylated as described previously [14]. Briefly, samples were dried and re-dissolved in 4 M urea 10% ACN 100 mM ammonium bicarbonate, pH 8. Cystines were then reduced by incubating with 5 mM dithiothreitol at 70 °C for 5 min and alkylated with 10 mM iodoacetamide at 37 °C for 90 min. The reduced and alkylated samples were then digested by incubating with 30 µg/µL trypsin overnight at 37 °C in 2 M urea 10% ACN 100 mM ammonium bicarbonate, pH 8, at a final substrate to enzyme ratio of approximately 100:1. The digested sample was desalted using a C18 ZipTip (ThermoFisher, Waltham, MA, USA), dried using vacuum centrifugation, dissolved in 0.5% FA and 2 µg analysed on an AB Sciex 5600 TripleTOF equipped with a Turbo-V source heated to 550 °C. Tryptic peptides were fractionated on a Shimadzu (Kyoto, Japan) Nexera UHPLC with an Agilent Zorbax stable-bond C18 column (Agilent, Santa Clara, CA, USA) (2.1 × 100 mm, 1.8 µm particle size, 300 Å pore size), using a flow rate of 180 µL/min and a gradient of 1–40% solvent B (90% ACN, 0.1% formic acid [FA]) in 0.1% FA over 60 min. MS1 spectra were acquired at 300–1800 *m*/*z* with an accumulation time of 250 ms and selecting the 20 most intense ions for MS2 scans acquired at 80–1400 *m*/*z* with an accumulation time of 100 ms and optimized for high resolution. Precursor ions with a charge of +2 to +5 and an intensity of at least 120 counts/s were selected, with a unit mass precursor ion inclusion window of ±0.7 Da and excluding isotopes within ±2 Da for MS/MS.

To identify proteins and peptides, Protein Pilot v5.0 (AB SCIEX, Framingham, MA, USA) was used to search the resulting MS/MS spectra against the full Trinity assembled transcriptome translated to all possible open reading frames longer than 40 amino acids using the Galaxy tool ‘Get open reading frames (ORFs) or coding sequences (CDSs)’ [39]. While biological modifications were allowed, we did not allow for amino acid substitutions in an attempt to reduce the number of false positive identifications of any similar non-toxin homologues. False positives were identified using decoy-based false discovery rates (FDR) as estimated by Protein Pilot and only protein identifications with a corresponding local FDR of <0.5% were considered significant.

### 5.4. Toxin Annotation

For toxin annotation, we used BLAST+ blastp [40] to search the translated assembled transcriptomes against three separate sequence databases: (i) all annotated toxin and venom protein entries in UniProtKB [41,42] (“ToxProt”; www.uniprot.org/program/Toxins; accessed July 2017; 6534 sequences); (ii) all published centipede toxins available in the NCBI nr, EST and TSA databases (“lineage-specific”; accession numbers from [8,9,21,22,43] as well as manual inspection of all centipede non-ribosomal sequences; accessed July 2017; 760 sequences); or (iii) all proteomically identified sequences combined with all published *S. s. mutilans* toxins available in the NCBI nr, EST and TSA databases (“species-specific”; accessed July 2017; 255 sequences). For BLAST searches, we used an e-value threshold of 0.001 and default blastp parameters (BLOSUM62, word size 3, minimum query coverage 0%). Sequences were organized into families according to their best hit [6,22], sequences filtered based on the presence of a signal peptide as predicted using SignalP v.4.1 [44,45], identical sequences removed using CD-HIT [46] and finally inspected manually to remove any false positives. Correlations and graphical analyses of proteomic detection and expression levels were carried out in Prism v7 (GraphPad), while Venn diagrams were constructed using the proportional Venn diagram tool available through Galaxy Queensland [47]. All identified putative and proteomically confirmed venom components have been submitted to NCBI TSA as a targeted submission and are available under BioProject PRJNA422449.

### 5.5. Phylogenetic Analyses

Grouped sequences were aligned using MAFFT v.7.304 (l-insi command) [48]. For phylogenetic analyses by Maximum Likelihood we used IQ-Tree v1.5.5 [49]. Evolutionary models were determined using ModelFinder [50] and support values estimated by ultrafast bootstrap using 10,000 iterations [51]. The resulting trees were visualized using Archaeopteryx v0.9916 [52], which was also used to automatically plot expression values and detection in venom.

## Figures and Tables

**Table 1:** Novel centipede venom proteins and peptides with predicted signal peptides. Details of all identified contigs, contigs detected in venom and their expression levels are listed in Table S1.

Group	Description	Number Cysteines	CDS Length	Contigs in Transcriptome	Contigs in Venom
**Novel 1**	*SLPTX29*—Lepidopteran putative fungal protease inhibitor-like peptide. Structurally related to insulin-like growth factor domain containing protein (sIGFBP)	12–14	97–128	11	1
**Novel 2**	*SLPTX30*—Pheromone binding protein-like peptide (IPR006170)	3–6	88–180	16	4
**Novel 3**	Cystine-knot cytokine domain containing TGF-beta-like peptide	10–12	127	1	1
**Novel 4**	*SLPTX31*—No BLAST or InterPro hits	0	45–70	6	4
**Novel 5**	Cathepsin L-type cysteine-protease	7	334–335	2	1
**Novel 6**	C-type lectin-like	8	15–236	7	2
**Novel 7**	Phosphatidylethanolamine-binding protein (IPR008914)	4	183–213	2	2
**Novel 8**	No BLAST or InterPro hits (Uncharacterized family 12)	10–12	190–204	2	1
**Novel 9**	No InterPro hits, similar to uncharacterised protein from *Strigamia maritima* (T1J4X8) (Uncharacterised family 13)	6	300–313	2	1
**Novel 10**	Peptidylglycine alpha-hydroxylating monooxygenase	8–13	344–919	2	1
**Novel 11**	Icarapin-like (Venom carbohydrate-rich protein)	0	227	1	1
**Novel 12**	No InterPro hits, similar to uncharacterised protein from *Strigamia maritima* (T1J2G0) (Uncharacterized family 14)	5	222	1	1
**Novel 13**	No BLAST or InterPro hits (Uncharacterized family 15)	8	275	1	1

**Table 2:** Comparison of four methods for identifying putative toxins from *S. s. subspinipes*. BLAST searches were done against all annotated toxin sequences in UniProtKB (ToxProt), all published centipede toxin sequences (lineage-specific), or all protein sequences obtained by Protein Pilot (species-specific).

	Proteome	Species Specific	Lineage Specific	ToxProt
**Unique contigs**	550	956	1357	1214
**Unique CDS**	469	811	1115	1058
**Unique CDS with signal peptide**	248	401	458	280
**Unique full-length CDS**	156	269	291	184

## Supplementary Materials

The following are available online at http://www.mdpi.com/2072-6651/10/3/96/s1, Table S1: Putative and identified toxins, expression values, proteomic detection and completeness of ORF; Table S2: Protein Pilot protein summaries of all identified CDSs in the combined proteomic data as well as additional contigs identified by concatenated individual Protein Pilot data searches; Tables S3–S11: Protein Pilot protein summaries of data from the rpHPLC fractions shown in Figure 2; Supplementary Files S1 and S2: Maximum likelihood phylogenies and sequence alignments of contigs from the venom gland and muscle transcriptomes within each identified toxin family; Supplementary Files S3 and S4: Expression values and proteomic detection mapped onto phylogenetic reconstructions of contigs from the combined tissue transcriptome for all toxin families detected in *S. s. subspinipes* venom, along with their respective sequence alignments. Normalized expression levels (FPKM) are shown as heat maps in coloured boxes divided in half for venom gland (left) or muscle (right) and highlighted in yellow for sequences detected in the venom. Bootstrapping consensus trees are shown as mid-point rooted, while horizontal bars indicate genetic distance; Supplementary Files S5 and S6: Expression values and proteomic detection mapped onto phylogenetic reconstructions of all known toxins in the toxin families detected in *S. s. subspinipes* venom, along with their respective sequence alignments. S. s. subspinipes contigs are from the combined tissue transcriptome. Normalized expression levels (FPKM) are shown as heat maps in coloured boxes divided in half for venom gland (left) or muscle (right) and highlighted in yellow for sequences detected in the venom. Bootstrapping consensus trees are shown as mid-point rooted, while horizontal bars indicate genetic distance.
