# Supplementary material for: True Lies: Using Proteomics to Assess the Accuracy of Transcriptome-Based Venomics in Centipedes Uncovers False Positives and Reveals Startling Intraspecific Variation in Scolopendra subspinipes
Source: Toxins (Basel). 2018 Feb 28;10(3):96. doi: 10.3390/toxins10030096 (PMC5869384; doi:10.3390/toxins10030096)
Supplement: Supplementary file 1 [file toxins-10-00096-s001.zip › Supplementary file 5.pdf]

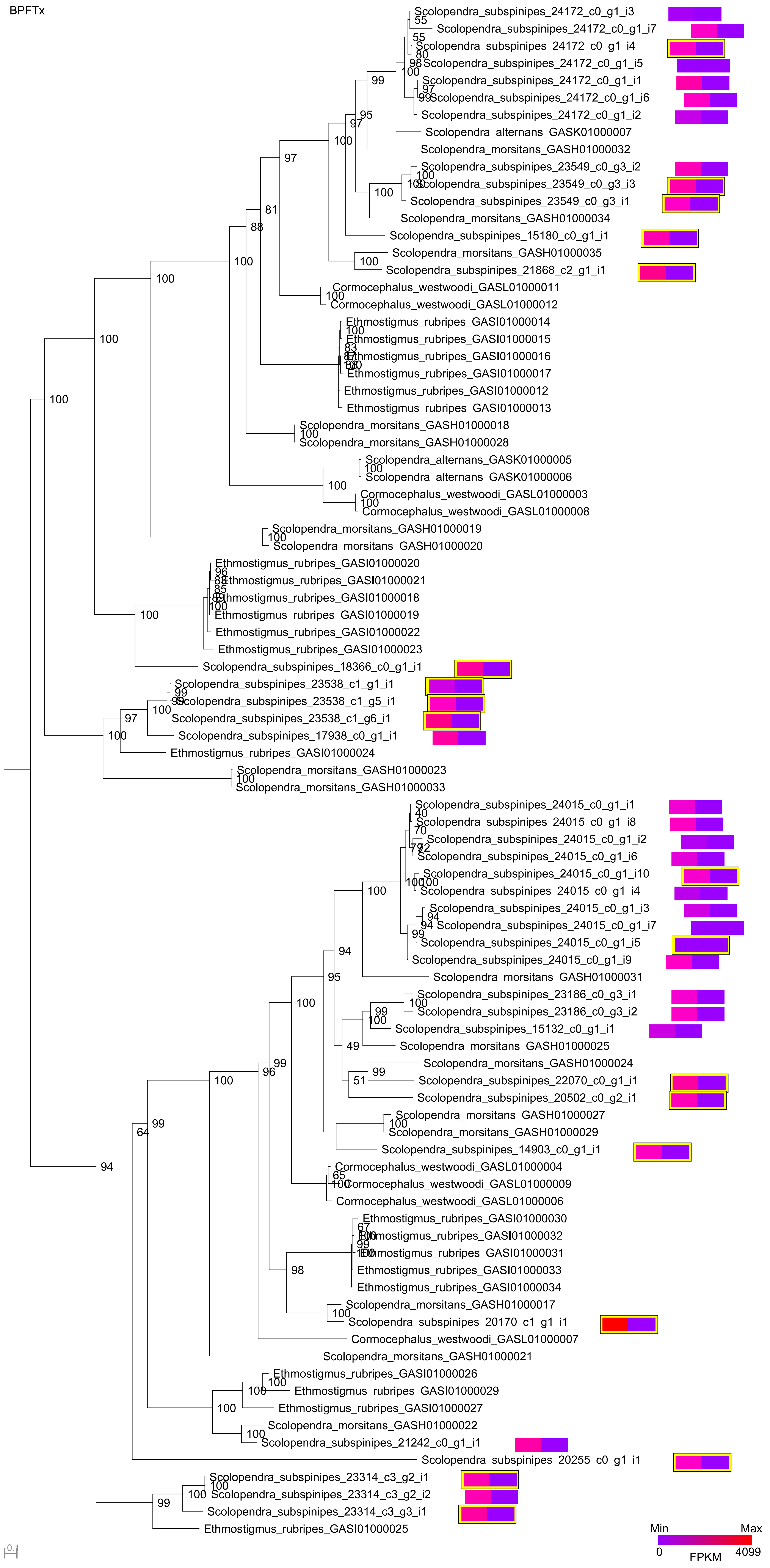
BPFTx
Scolopendra_subspinipes_24172_c0_g1_i3
Scolopendra_subspinipes_24172_c0_g1_i7
Scolopendra_subspinipes_24172_c0_g1_i4
Scolopendra_subspinipes_24172_c0_g1_i5
Scolopendra_subspinipes_24172_c0_g1_i1
Scolopendra_subspinipes_24172_c0_g1_i6
Scolopendra_subspinipes_24172_c0_g1_i2
Scolopendra_alternans_GASK01000007
Scolopendra_morsitans_GASH01000032
Scolopendra_subspinipes_23549_c0_g3_i2
Scolopendra_subspinipes_23549_c0_g3_i3
Scolopendra_subspinipes_23549_c0_g3_i1
Scolopendra_morsitans_GASH01000034
Scolopendra_subspinipes_15180_c0_g1_i1
Scolopendra_morsitans_GASH01000035
Scolopendra_subspinipes_21868_c2_g1_i1
Cormocephalus_westwoodi_GASL01000011
Cormocephalus_westwoodi_GASL01000012
Ethmostigmus_rubripes_GASI01000014
Ethmostigmus_rubripes_GASI01000015
Ethmostigmus_rubripes_GASI01000016
Ethmostigmus_rubripes_GASI01000017
Ethmostigmus_rubripes_GASI01000012
Ethmostigmus_rubripes_GASI01000013
Scolopendra_morsitans_GASH01000018
Scolopendra_morsitans_GASH01000028
Scolopendra_alternans_GASK01000005
Scolopendra_alternans_GASK01000006
Cormocephalus_westwoodi_GASL01000003
Cormocephalus_westwoodi_GASL01000008
Scolopendra_morsitans_GASH01000019
Scolopendra_morsitans_GASH01000020
Ethmostigmus_rubripes_GASI01000020
Ethmostigmus_rubripes_GASI01000021
Ethmostigmus_rubripes_GASI01000018
Ethmostigmus_rubripes_GASI01000019
Ethmostigmus_rubripes_GASI01000022
Ethmostigmus_rubripes_GASI01000023
Scolopendra_subspinipes_18366_c0_g1_i1
Scolopendra_subspinipes_23538_c1_g1_i1
Scolopendra_subspinipes_23538_c1_g5_i1
Scolopendra_subspinipes_23538_c1_g6_i1
Scolopendra_subspinipes_17938_c0_g1_i1
Ethmostigmus_rubripes_GASI01000024
Scolopendra_morsitans_GASH01000023
Scolopendra_morsitans_GASH01000033
Scolopendra_subspinipes_24015_c0_g1_i1
Scolopendra_subspinipes_24015_c0_g1_i8
Scolopendra_subspinipes_24015_c0_g1_i2
Scolopendra_subspinipes_24015_c0_g1_i6
Scolopendra_subspinipes_24015_c0_g1_i10
Scolopendra_subspinipes_24015_c0_g1_i4
Scolopendra_subspinipes_24015_c0_g1_i3
Scolopendra_subspinipes_24015_c0_g1_i7
Scolopendra_subspinipes_24015_c0_g1_i5
Scolopendra_subspinipes_24015_c0_g1_i9
Scolopendra_morsitans_GASH01000031
Scolopendra_subspinipes_23186_c0_g3_i1
Scolopendra_subspinipes_23186_c0_g3_i2
Scolopendra_subspinipes_15132_c0_g1_i1
Scolopendra_morsitans_GASH01000025
Scolopendra_morsitans_GASH01000024
Scolopendra_subspinipes_22070_c0_g1_i1
Scolopendra_subspinipes_20502_c0_g2_i1
Scolopendra_morsitans_GASH01000027
Scolopendra_morsitans_GASH01000029
Scolopendra_subspinipes_14903_c0_g1_i1
Cormocephalus_westwoodi_GASL01000004
Cormocephalus_westwoodi_GASL01000009
Cormocephalus_westwoodi_GASL01000006
Ethmostigmus_rubripes_GASI01000030
Ethmostigmus_rubripes_GASI01000032
Ethmostigmus_rubripes_GASI01000031
Ethmostigmus_rubripes_GASI01000033
Ethmostigmus_rubripes_GASI01000034
Scolopendra_morsitans_GASH01000017
Scolopendra_subspinipes_20170_c1_g1_i1
Cormocephalus_westwoodi_GASL01000007
Scolopendra_morsitans_GASH01000021
Ethmostigmus_rubripes_GASI01000026
Ethmostigmus_rubripes_GASI01000029
Ethmostigmus_rubripes_GASI01000027
Scolopendra_morsitans_GASH01000022
Scolopendra_subspinipes_21242_c0_g1_i1
Scolopendra_subspinipes_20255_c0_g1_i1
Scolopendra_subspinipes_23314_c3_g2_i1
Scolopendra_subspinipes_23314_c3_g2_i2
Scolopendra_subspinipes_23314_c3_g3_i1
Ethmostigmus_rubripes_GASI01000025
Min
Max
0
FPKM
4099
0.1

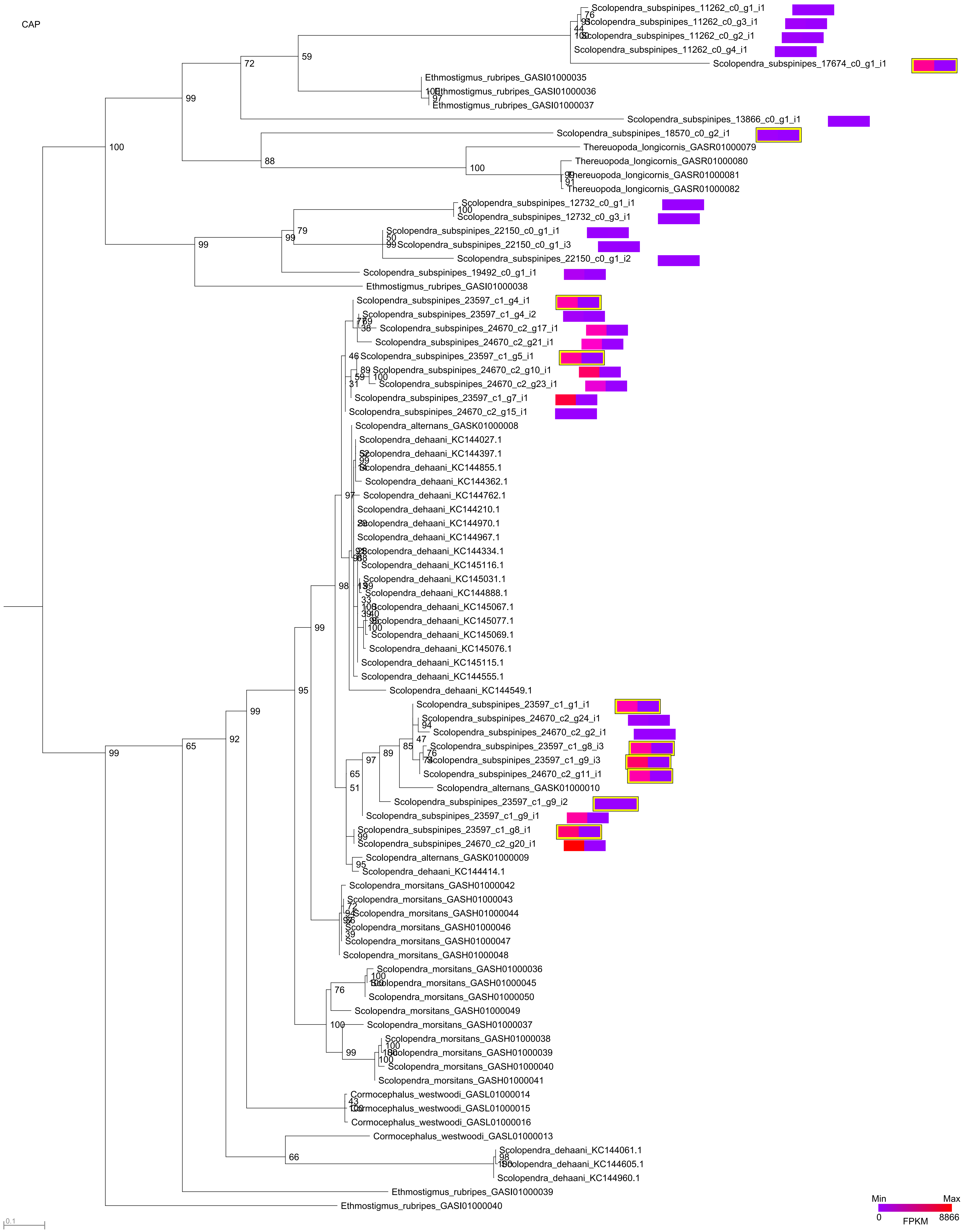
CAP
Scolopendra_subspinipes_11262_c0_g1_i1
Scolopendra_subspinipes_11262_c0_g3_i1
Scolopendra_subspinipes_11262_c0_g2_i1
Scolopendra_subspinipes_11262_c0_g4_i1
Scolopendra_subspinipes_17674_c0_g1_i1
Ethmostigmus_rubripes_GASI01000035
Ethmostigmus_rubripes_GASI01000036
Ethmostigmus_rubripes_GASI01000037
Scolopendra_subspinipes_13866_c0_g1_i1
Scolopendra_subspinipes_18570_c0_g2_i1
Thereuopoda_longicornis_GASR01000079
Thereuopoda_longicornis_GASR01000080
Thereuopoda_longicornis_GASR01000081
Thereuopoda_longicornis_GASR01000082
Scolopendra_subspinipes_12732_c0_g1_i1
Scolopendra_subspinipes_12732_c0_g3_i1
Scolopendra_subspinipes_22150_c0_g1_i1
Scolopendra_subspinipes_22150_c0_g1_i3
Scolopendra_subspinipes_22150_c0_g1_i2
Scolopendra_subspinipes_19492_c0_g1_i1
Ethmostigmus_rubripes_GASI01000038
Scolopendra_subspinipes_23597_c1_g4_i1
Scolopendra_subspinipes_23597_c1_g4_i2
Scolopendra_subspinipes_24670_c2_g17_i1
Scolopendra_subspinipes_24670_c2_g21_i1
Scolopendra_subspinipes_23597_c1_g5_i1
Scolopendra_subspinipes_24670_c2_g10_i1
Scolopendra_subspinipes_24670_c2_g23_i1
Scolopendra_subspinipes_23597_c1_g7_i1
Scolopendra_subspinipes_24670_c2_g15_i1
Scolopendra_alternans_GASK01000008
Scolopendra_dehaani_KC144027.1
Scolopendra_dehaani_KC144397.1
Scolopendra_dehaani_KC144855.1
Scolopendra_dehaani_KC144362.1
Scolopendra_dehaani_KC144762.1
Scolopendra_dehaani_KC144210.1
Scolopendra_dehaani_KC144970.1
Scolopendra_dehaani_KC144967.1
Scolopendra_dehaani_KC144334.1
Scolopendra_dehaani_KC145116.1
Scolopendra_dehaani_KC145031.1
Scolopendra_dehaani_KC144888.1
Scolopendra_dehaani_KC145067.1
Scolopendra_dehaani_KC145077.1
Scolopendra_dehaani_KC145069.1
Scolopendra_dehaani_KC145076.1
Scolopendra_dehaani_KC145115.1
Scolopendra_dehaani_KC144555.1
Scolopendra_dehaani_KC144549.1
Scolopendra_subspinipes_23597_c1_g1_i1
Scolopendra_subspinipes_24670_c2_g24_i1
Scolopendra_subspinipes_24670_c2_g2_i1
Scolopendra_subspinipes_23597_c1_g8_i3
Scolopendra_subspinipes_23597_c1_g9_i3
Scolopendra_subspinipes_24670_c2_g11_i1
Scolopendra_alternans_GASK01000010
Scolopendra_subspinipes_23597_c1_g9_i2
Scolopendra_subspinipes_23597_c1_g9_i1
Scolopendra_subspinipes_23597_c1_g8_i1
Scolopendra_subspinipes_24670_c2_g20_i1
Scolopendra_alternans_GASK01000009
Scolopendra_dehaani_KC144414.1
Scolopendra_morsitans_GASH01000042
Scolopendra_morsitans_GASH01000043
Scolopendra_morsitans_GASH01000044
Scolopendra_morsitans_GASH01000046
Scolopendra_morsitans_GASH01000047
Scolopendra_morsitans_GASH01000048
Scolopendra_morsitans_GASH01000036
Scolopendra_morsitans_GASH01000045
Scolopendra_morsitans_GASH01000050
Scolopendra_morsitans_GASH01000049
Scolopendra_morsitans_GASH01000037
Scolopendra_morsitans_GASH01000038
Scolopendra_morsitans_GASH01000039
Scolopendra_morsitans_GASH01000040
Scolopendra_morsitans_GASH01000041
Cormocephalus_westwoodi_GASL01000014
Cormocephalus_westwoodi_GASL01000015
Cormocephalus_westwoodi_GASL01000016
Cormocephalus_westwoodi_GASL01000013
Scolopendra_dehaani_KC144061.1
Scolopendra_dehaani_KC144605.1
Scolopendra_dehaani_KC144960.1
Ethmostigmus_rubripes_GASI01000039
Ethmostigmus_rubripes_GASI01000040
0.1
Min
Max
0
FPKM
8866

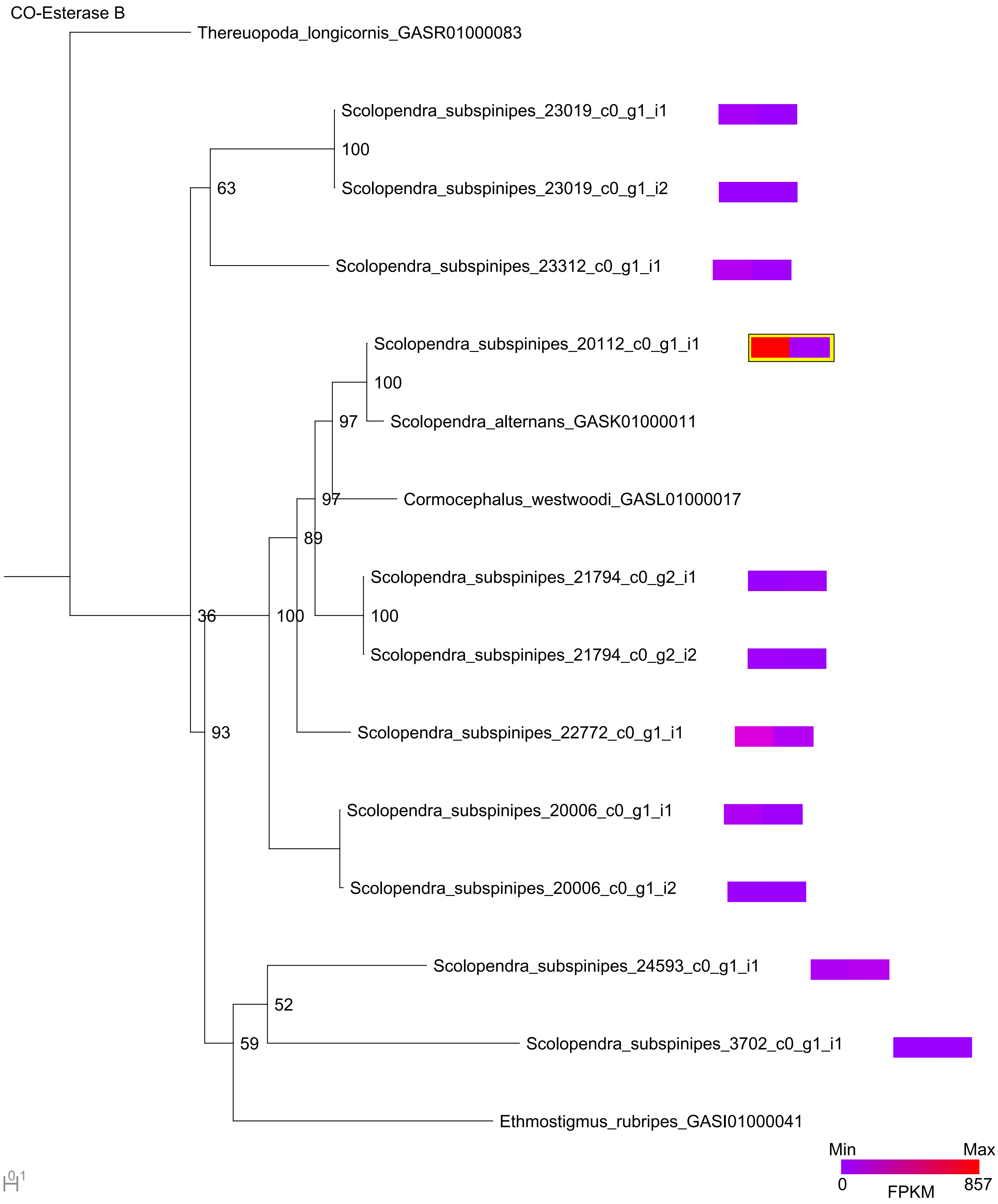
CO-Esterase B
Thereuopoda_longicornis_GASR01000083
Scolopendra_subspinipes_23019_c0_g1_i1
100
Scolopendra_subspinipes_23019_c0_g1_i2
63
Scolopendra_subspinipes_23312_c0_g1_i1
Scolopendra_subspinipes_20112_c0_g1_i1
100
97
Scolopendra_alternans_GASK01000011
97
Cormocephalus_westwoodi_GASL01000017
89
Scolopendra_subspinipes_21794_c0_g2_i1
36
100
100
Scolopendra_subspinipes_21794_c0_g2_i2
93
Scolopendra_subspinipes_22772_c0_g1_i1
Scolopendra_subspinipes_20006_c0_g1_i1
Scolopendra_subspinipes_20006_c0_g1_i2
Scolopendra_subspinipes_24593_c0_g1_i1
52
59
Scolopendra_subspinipes_3702_c0_g1_i1
Ethmostigmus_rubripes_GASI01000041
Min
Max
0
FPKM
857
0.1

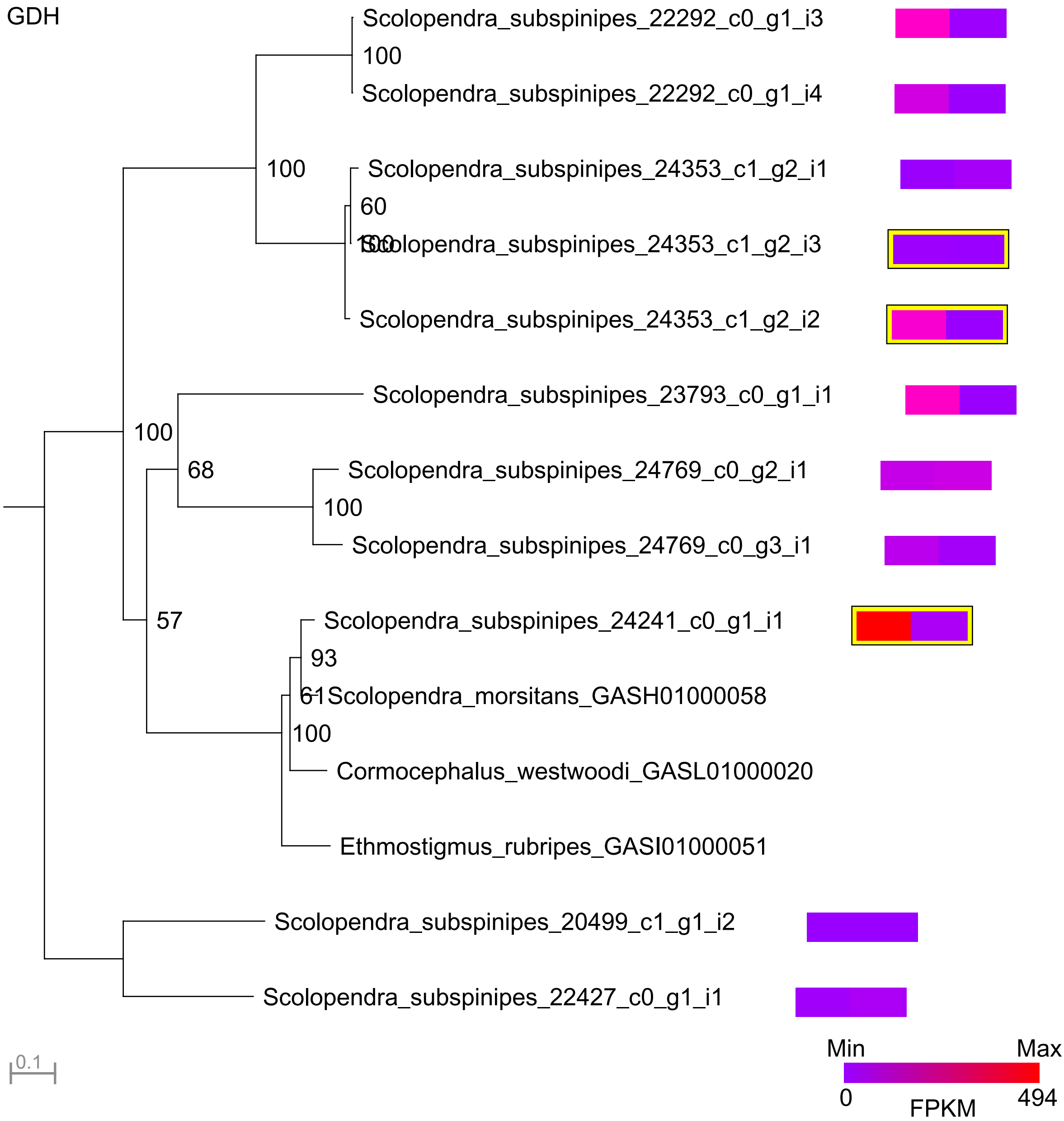
GDH
Scolopendra_subspinipes_22292_c0_g1_i3
100
Scolopendra_subspinipes_22292_c0_g1_i4
100
Scolopendra_subspinipes_24353_c1_g2_i1
60
100
Scolopendra_subspinipes_24353_c1_g2_i3
Scolopendra_subspinipes_24353_c1_g2_i2
Scolopendra_subspinipes_23793_c0_g1_i1
100
68
Scolopendra_subspinipes_24769_c0_g2_i1
100
Scolopendra_subspinipes_24769_c0_g3_i1
57
Scolopendra_subspinipes_24241_c0_g1_i1
93
61
Scolopendra_morsitans_GASH01000058
100
Cormocephalus_westwoodi_GASL01000020
Ethmostigmus_rubripes_GASI01000051
Scolopendra_subspinipes_20499_c1_g1_i2
Scolopendra_subspinipes_22427_c0_g1_i1
0.1
Min
Max
0
FPKM
494

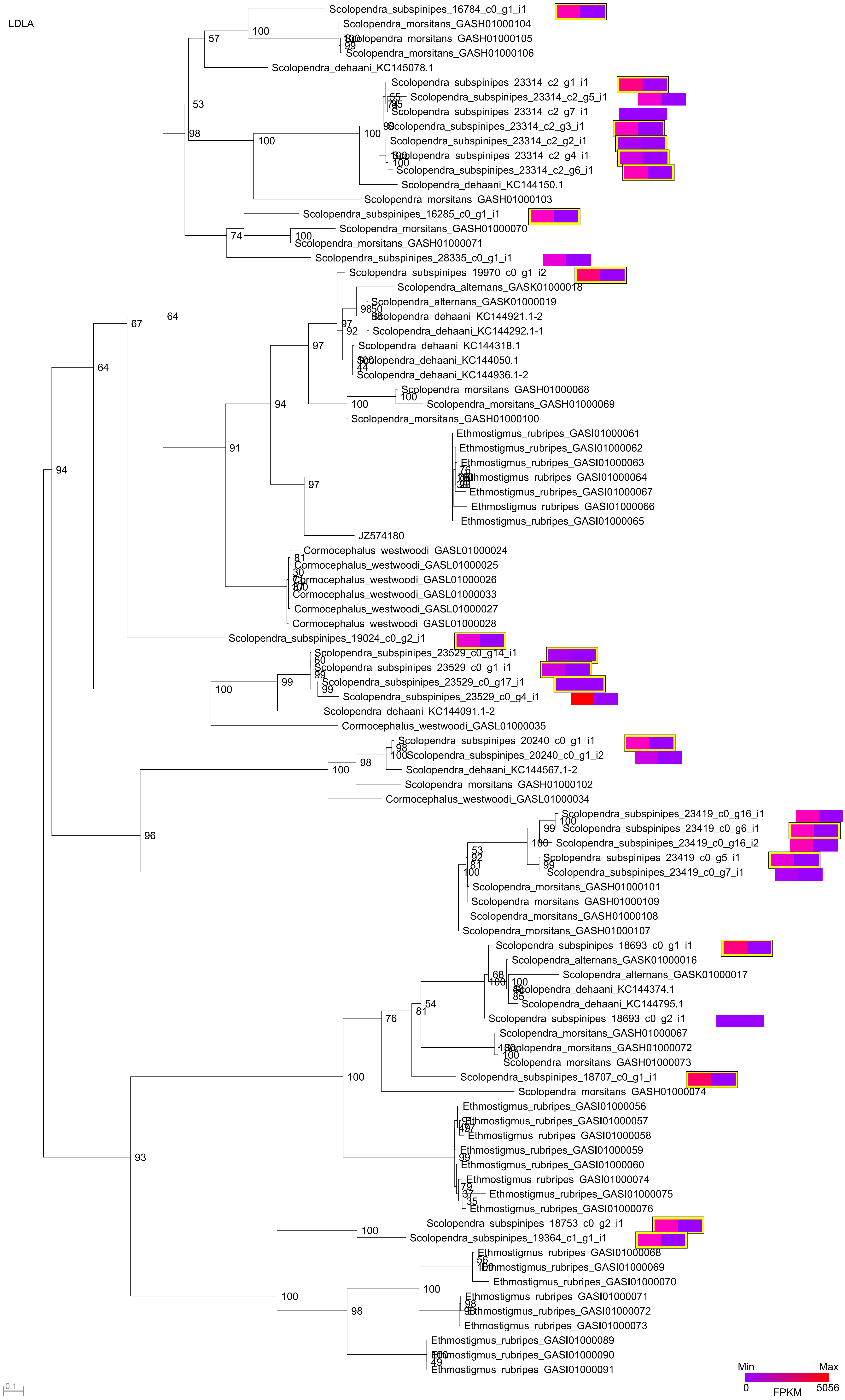
LDLA
Scolopendra_subspinipes_16784_c0_g1_i1
Scolopendra_morsitans_GASH01000104
Scolopendra_morsitans_GASH01000105
Scolopendra_morsitans_GASH01000106
Scolopendra_dehaani_KC145078.1
Scolopendra_subspinipes_23314_c2_g1_i1
Scolopendra_subspinipes_23314_c2_g5_i1
Scolopendra_subspinipes_23314_c2_g7_i1
Scolopendra_subspinipes_23314_c2_g3_i1
Scolopendra_subspinipes_23314_c2_g2_i1
Scolopendra_subspinipes_23314_c2_g4_i1
Scolopendra_subspinipes_23314_c2_g6_i1
Scolopendra_dehaani_KC144150.1
Scolopendra_morsitans_GASH01000103
Scolopendra_subspinipes_16285_c0_g1_i1
Scolopendra_morsitans_GASH01000070
Scolopendra_morsitans_GASH01000071
Scolopendra_subspinipes_28335_c0_g1_i1
Scolopendra_subspinipes_19970_c0_g1_i2
Scolopendra_alternans_GASK01000018
Scolopendra_alternans_GASK01000019
Scolopendra_dehaani_KC144921.1-2
Scolopendra_dehaani_KC144292.1-1
Scolopendra_dehaani_KC144318.1
Scolopendra_dehaani_KC144050.1
Scolopendra_dehaani_KC144936.1-2
Scolopendra_morsitans_GASH01000068
Scolopendra_morsitans_GASH01000069
Scolopendra_morsitans_GASH01000100
Ethmostigmus_rubripes_GASI01000061
Ethmostigmus_rubripes_GASI01000062
Ethmostigmus_rubripes_GASI01000063
Ethmostigmus_rubripes_GASI01000064
Ethmostigmus_rubripes_GASI01000067
Ethmostigmus_rubripes_GASI01000066
Ethmostigmus_rubripes_GASI01000065
JZ574180
Cormocephalus_westwoodi_GASL01000024
Cormocephalus_westwoodi_GASL01000025
Cormocephalus_westwoodi_GASL01000026
Cormocephalus_westwoodi_GASL01000033
Cormocephalus_westwoodi_GASL01000027
Cormocephalus_westwoodi_GASL01000028
Scolopendra_subspinipes_19024_c0_g2_i1
Scolopendra_subspinipes_23529_c0_g14_i1
Scolopendra_subspinipes_23529_c0_g1_i1
Scolopendra_subspinipes_23529_c0_g17_i1
Scolopendra_subspinipes_23529_c0_g4_i1
Scolopendra_dehaani_KC144091.1-2
Cormocephalus_westwoodi_GASL01000035
Scolopendra_subspinipes_20240_c0_g1_i1
Scolopendra_subspinipes_20240_c0_g1_i2
Scolopendra_dehaani_KC144567.1-2
Scolopendra_morsitans_GASH01000102
Cormocephalus_westwoodi_GASL01000034
Scolopendra_subspinipes_23419_c0_g16_i1
Scolopendra_subspinipes_23419_c0_g6_i1
Scolopendra_subspinipes_23419_c0_g16_i2
Scolopendra_subspinipes_23419_c0_g5_i1
Scolopendra_subspinipes_23419_c0_g7_i1
Scolopendra_morsitans_GASH01000101
Scolopendra_morsitans_GASH01000109
Scolopendra_morsitans_GASH01000108
Scolopendra_morsitans_GASH01000107
Scolopendra_subspinipes_18693_c0_g1_i1
Scolopendra_alternans_GASK01000016
Scolopendra_alternans_GASK01000017
Scolopendra_dehaani_KC144374.1
Scolopendra_dehaani_KC144795.1
Scolopendra_subspinipes_18693_c0_g2_i1
Scolopendra_morsitans_GASH01000067
Scolopendra_morsitans_GASH01000072
Scolopendra_morsitans_GASH01000073
Scolopendra_subspinipes_18707_c0_g1_i1
Scolopendra_morsitans_GASH01000074
Ethmostigmus_rubripes_GASI01000056
Ethmostigmus_rubripes_GASI01000057
Ethmostigmus_rubripes_GASI01000058
Ethmostigmus_rubripes_GASI01000059
Ethmostigmus_rubripes_GASI01000060
Ethmostigmus_rubripes_GASI01000074
Ethmostigmus_rubripes_GASI01000075
Ethmostigmus_rubripes_GASI01000076
Scolopendra_subspinipes_18753_c0_g2_i1
Scolopendra_subspinipes_19364_c1_g1_i1
Ethmostigmus_rubripes_GASI01000068
Ethmostigmus_rubripes_GASI01000069
Ethmostigmus_rubripes_GASI01000070
Ethmostigmus_rubripes_GASI01000071
Ethmostigmus_rubripes_GASI01000072
Ethmostigmus_rubripes_GASI01000073
Ethmostigmus_rubripes_GASI01000089
Ethmostigmus_rubripes_GASI01000090
Ethmostigmus_rubripes_GASI01000091
0.1
Min
Max
0
FPKM
5056

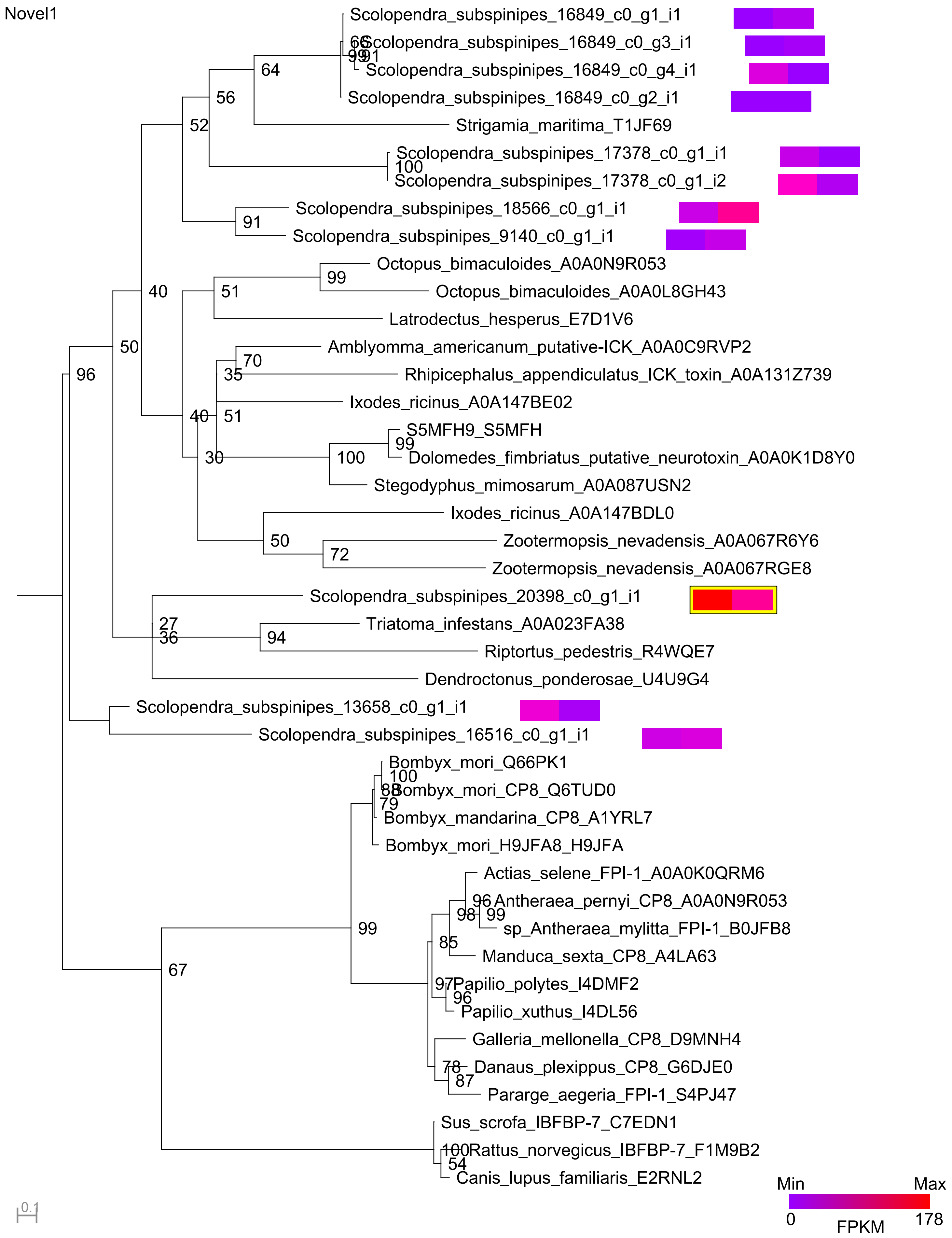

Novel1
Scolopendra_subspinipes_16849_c0_g1_i1
Scolopendra_subspinipes_16849_c0_g3_i1
Scolopendra_subspinipes_16849_c0_g4_i1
Scolopendra_subspinipes_16849_c0_g2_i1
Strigamia_maritima_T1JF69
Scolopendra_subspinipes_17378_c0_g1_i1
Scolopendra_subspinipes_17378_c0_g1_i2
Scolopendra_subspinipes_18566_c0_g1_i1
Scolopendra_subspinipes_9140_c0_g1_i1
Octopus_bimaculoides_A0A0N9R053
Octopus_bimaculoides_A0A0L8GH43
Latrodectus_hesperus_E7D1V6
Amblyomma_americanum_putative-ICK_A0A0C9RVP2
Rhipicephalus_appendiculatus_ICK_toxin_A0A131Z739
Ixodes_ricinus_A0A147BE02
S5MFH9_S5MFH
Dolomedes_fimbriatus_putative_neurotoxin_A0A0K1D8Y0
Stegodyphus_mimosarum_A0A087USN2
Ixodes_ricinus_A0A147BDL0
Zootermopsis_nevadensis_A0A067R6Y6
Zootermopsis_nevadensis_A0A067RGE8
Scolopendra_subspinipes_20398_c0_g1_i1
Triatoma_infestans_A0A023FA38
Riptortus_pedestris_R4WQE7
Dendroctonus_ponderosae_U4U9G4
Scolopendra_subspinipes_13658_c0_g1_i1
Scolopendra_subspinipes_16516_c0_g1_i1
Bombyx_mori_Q66PK1
Bombyx_mori_CP8_Q6TUD0
Bombyx_mandarina_CP8_A1YRL7
Bombyx_mori_H9JFA8_H9JFA
Actias_selene_FPI-1_A0A0K0QRM6
Antheraea_pernyi_CP8_A0A0N9R053
sp_Antheraea_mylitta_FPI-1_B0JFB8
Manduca_sexta_CP8_A4LA63
Papilio_polytes_I4DMF2
Papilio_xuthus_I4DL56
Galleria_mellonella_CP8_D9MNH4
Danaus_plexippus_CP8_G6DJE0
Pararge_aegeria_FPI-1_S4PJ47
Sus_scrofa_IBFBP-7_C7EDN1
Rattus_norvegicus_IBFBP-7_F1M9B2
Canis_lupus_familiaris_E2RNL2
0.1
Min
Max
0
178
FPKM

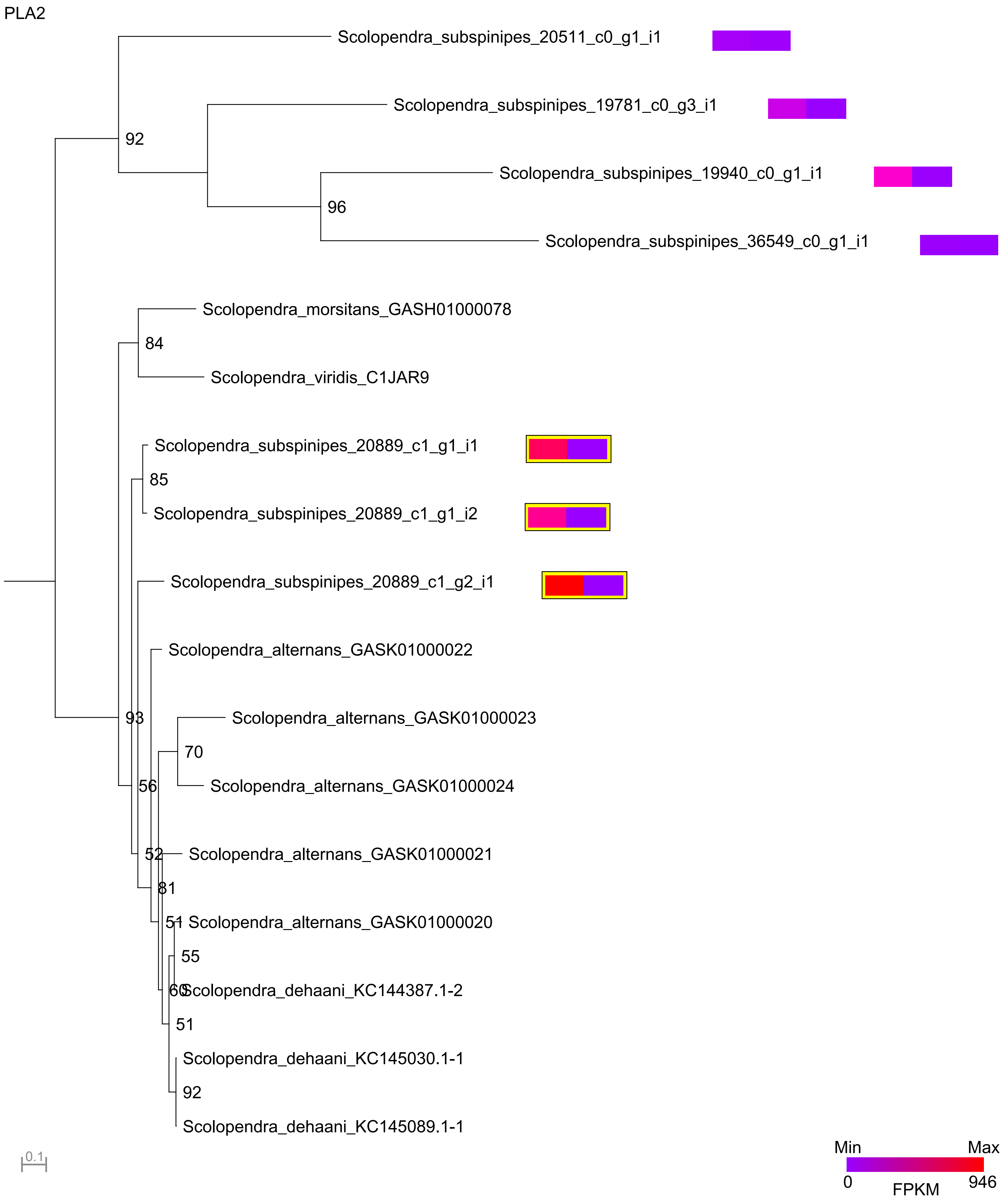
PLA2
Scolopendra_subspinipes_20511_c0_g1_i1
Scolopendra_subspinipes_19781_c0_g3_i1
92
Scolopendra_subspinipes_19940_c0_g1_i1
96
Scolopendra_subspinipes_36549_c0_g1_i1
Scolopendra_morsitans_GASH01000078
84
Scolopendra_viridis_C1JAR9
Scolopendra_subspinipes_20889_c1_g1_i1
85
Scolopendra_subspinipes_20889_c1_g1_i2
Scolopendra_subspinipes_20889_c1_g2_i1
Scolopendra_alternans_GASK01000022
Scolopendra_alternans_GASK01000023
93
70
Scolopendra_alternans_GASK01000024
56
52
Scolopendra_alternans_GASK01000021
81
51
Scolopendra_alternans_GASK01000020
55
60
Scolopendra_dehaani_KC144387.1-2
51
Scolopendra_dehaani_KC145030.1-1
92
Scolopendra_dehaani_KC145089.1-1
0.1
Min
Max
0
FPKM
946

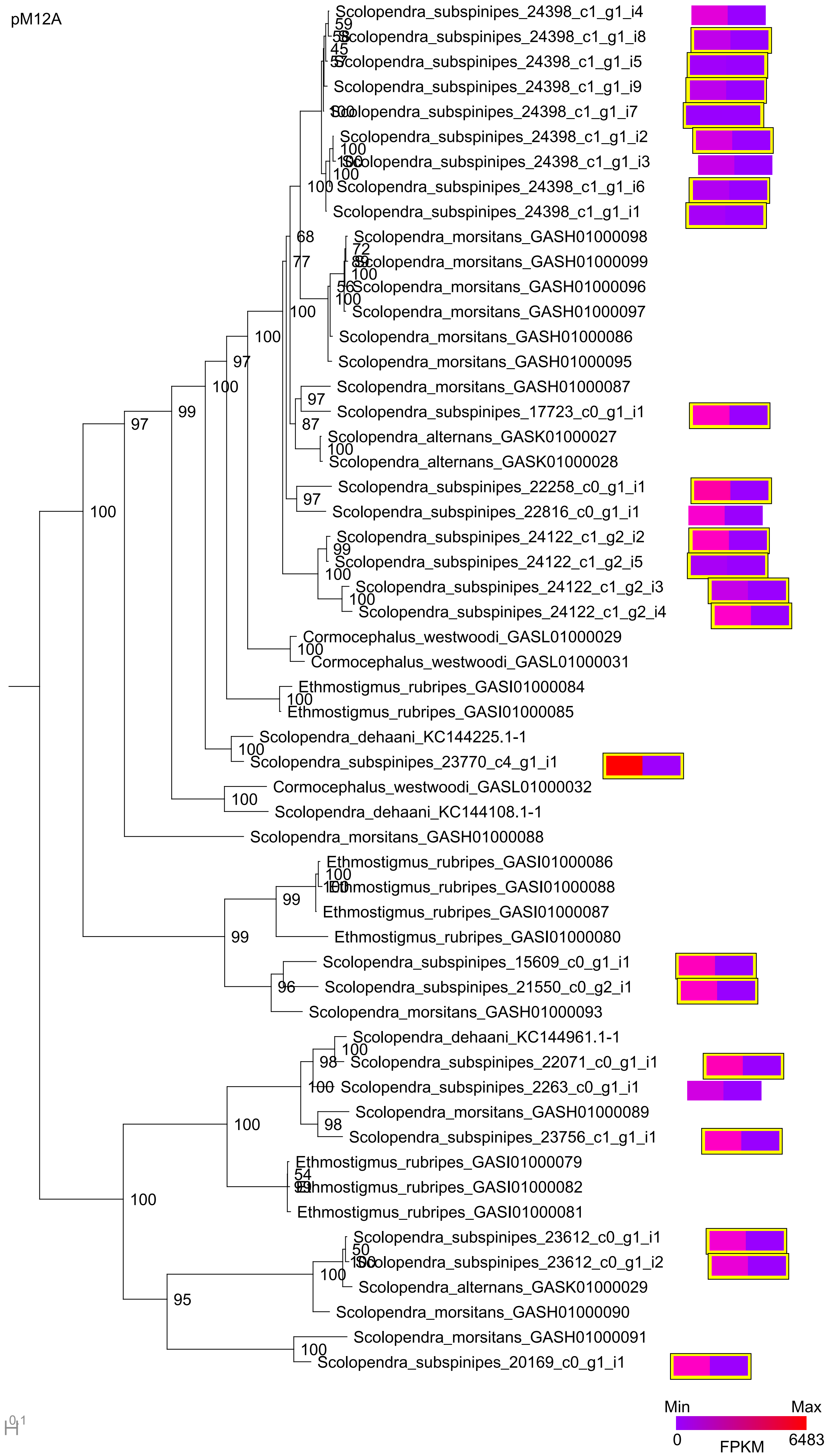

pM12A
Scolopendra_subspinipes_24398_c1_g1_i4
Scolopendra_subspinipes_24398_c1_g1_i8
Scolopendra_subspinipes_24398_c1_g1_i5
Scolopendra_subspinipes_24398_c1_g1_i9
Scolopendra_subspinipes_24398_c1_g1_i7
Scolopendra_subspinipes_24398_c1_g1_i2
Scolopendra_subspinipes_24398_c1_g1_i3
Scolopendra_subspinipes_24398_c1_g1_i6
Scolopendra_subspinipes_24398_c1_g1_i1
Scolopendra_morsitans_GASH01000098
Scolopendra_morsitans_GASH01000099
Scolopendra_morsitans_GASH01000096
Scolopendra_morsitans_GASH01000097
Scolopendra_morsitans_GASH01000086
Scolopendra_morsitans_GASH01000095
Scolopendra_morsitans_GASH01000087
Scolopendra_subspinipes_17723_c0_g1_i1
Scolopendra_alternans_GASK01000027
Scolopendra_alternans_GASK01000028
Scolopendra_subspinipes_22258_c0_g1_i1
Scolopendra_subspinipes_22816_c0_g1_i1
Scolopendra_subspinipes_24122_c1_g2_i2
Scolopendra_subspinipes_24122_c1_g2_i5
Scolopendra_subspinipes_24122_c1_g2_i3
Scolopendra_subspinipes_24122_c1_g2_i4
Cormocephalus_westwoodi_GASL01000029
Cormocephalus_westwoodi_GASL01000031
Ethmostigmus_rubripes_GASI01000084
Ethmostigmus_rubripes_GASI01000085
Scolopendra_dehaani_KC144225.1-1
Scolopendra_subspinipes_23770_c4_g1_i1
Cormocephalus_westwoodi_GASL01000032
Scolopendra_dehaani_KC144108.1-1
Scolopendra_morsitans_GASH01000088
Ethmostigmus_rubripes_GASI01000086
Ethmostigmus_rubripes_GASI01000088
Ethmostigmus_rubripes_GASI01000087
Ethmostigmus_rubripes_GASI01000080
Scolopendra_subspinipes_15609_c0_g1_i1
Scolopendra_subspinipes_21550_c0_g2_i1
Scolopendra_morsitans_GASH01000093
Scolopendra_dehaani_KC144961.1-1
Scolopendra_subspinipes_22071_c0_g1_i1
Scolopendra_subspinipes_2263_c0_g1_i1
Scolopendra_morsitans_GASH01000089
Scolopendra_subspinipes_23756_c1_g1_i1
Ethmostigmus_rubripes_GASI01000079
Ethmostigmus_rubripes_GASI01000082
Ethmostigmus_rubripes_GASI01000081
Scolopendra_subspinipes_23612_c0_g1_i1
Scolopendra_subspinipes_23612_c0_g1_i2
Scolopendra_alternans_GASK01000029
Scolopendra_morsitans_GASH01000090
Scolopendra_morsitans_GASH01000091
Scolopendra_subspinipes_20169_c0_g1_i1
0.1
Min
Max
0
FPKM
6483

S1

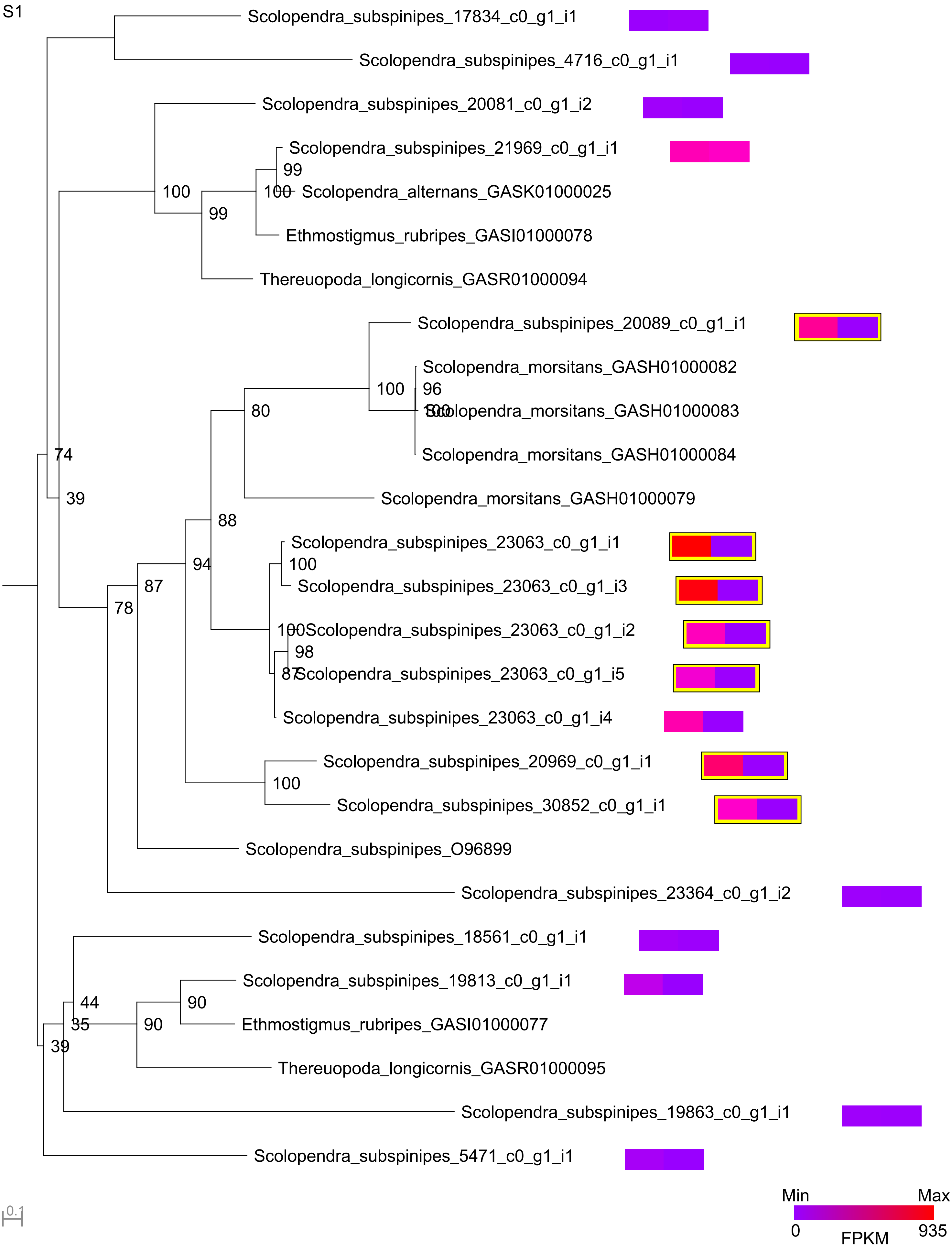

Scolopendra_subspinipes_17834_c0_g1_i1
Scolopendra_subspinipes_4716_c0_g1_i1
Scolopendra_subspinipes_20081_c0_g1_i2
Scolopendra_subspinipes_21969_c0_g1_i1
99
100
Scolopendra_alternans_GASK01000025
100
99
Ethmostigmus_rubripes_GASI01000078
Thereuopoda_longicornis_GASR01000094
Scolopendra_subspinipes_20089_c0_g1_i1
Scolopendra_morsitans_GASH01000082
100
96
100
Scolopendra_morsitans_GASH01000083
80
Scolopendra_morsitans_GASH01000084
74
39
Scolopendra_morsitans_GASH01000079
88
Scolopendra_subspinipes_23063_c0_g1_i1
94
100
Scolopendra_subspinipes_23063_c0_g1_i3
87
78
100
Scolopendra_subspinipes_23063_c0_g1_i2
98
87
Scolopendra_subspinipes_23063_c0_g1_i5
Scolopendra_subspinipes_23063_c0_g1_i4
Scolopendra_subspinipes_20969_c0_g1_i1
100
Scolopendra_subspinipes_30852_c0_g1_i1
Scolopendra_subspinipes_O96899
Scolopendra_subspinipes_23364_c0_g1_i2
Scolopendra_subspinipes_18561_c0_g1_i1
Scolopendra_subspinipes_19813_c0_g1_i1
44
90
35
90
Ethmostigmus_rubripes_GASI01000077
39
Thereuopoda_longicornis_GASR01000095
Scolopendra_subspinipes_19863_c0_g1_i1
Scolopendra_subspinipes_5471_c0_g1_i1
0.1
Min
Max
0
935
FPKM

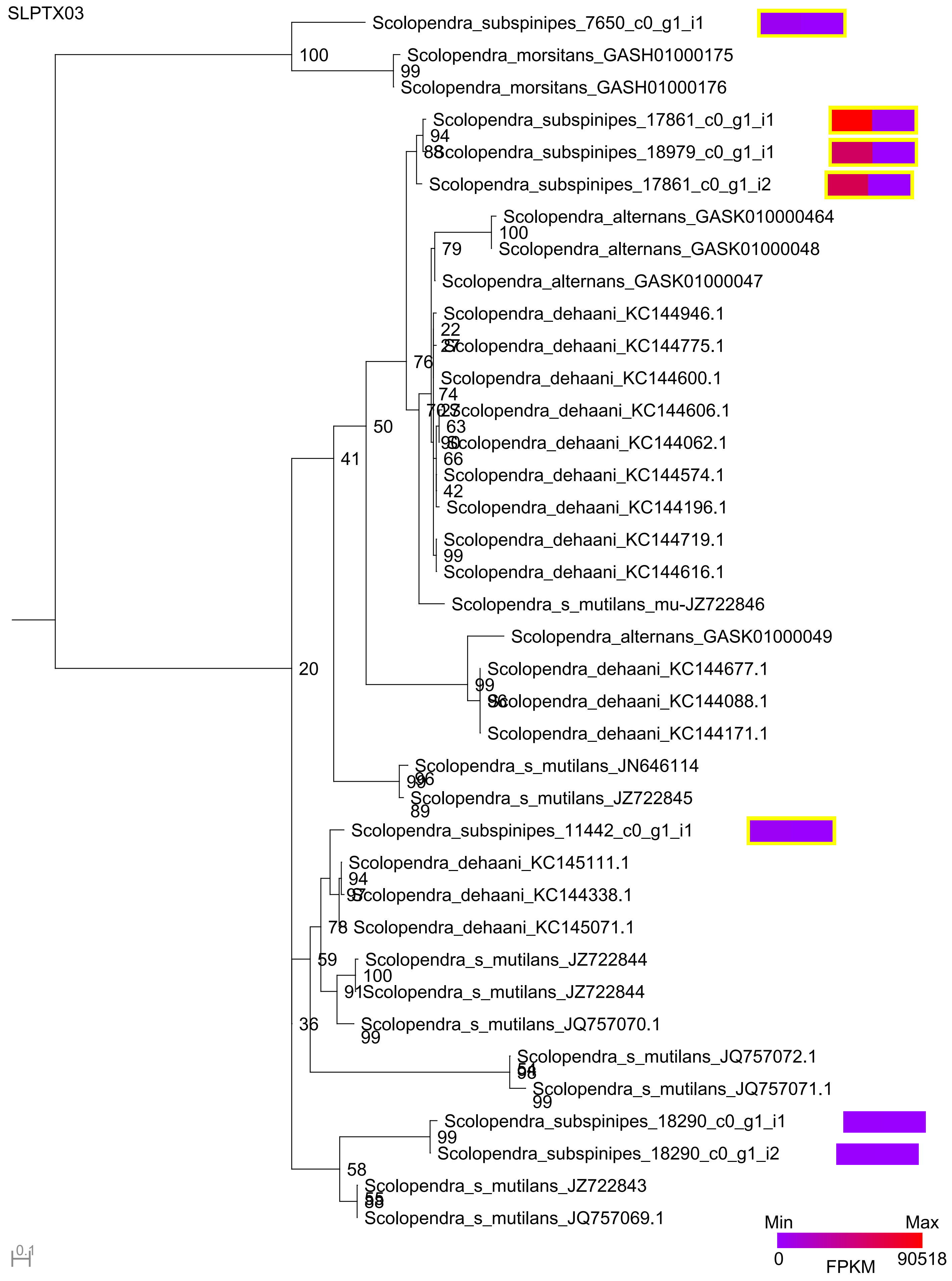
SLPTX03
Scolopendra_subspinipes_7650_c0_g1_i1
Scolopendra_morsitans_GASH01000175
Scolopendra_morsitans_GASH01000176
Scolopendra_subspinipes_17861_c0_g1_i1
Scolopendra_subspinipes_18979_c0_g1_i1
Scolopendra_subspinipes_17861_c0_g1_i2
Scolopendra_alternans_GASK010000464
Scolopendra_alternans_GASK01000048
Scolopendra_alternans_GASK01000047
Scolopendra_dehaani_KC144946.1
Scolopendra_dehaani_KC144775.1
Scolopendra_dehaani_KC144600.1
Scolopendra_dehaani_KC144606.1
Scolopendra_dehaani_KC144062.1
Scolopendra_dehaani_KC144574.1
Scolopendra_dehaani_KC144196.1
Scolopendra_dehaani_KC144719.1
Scolopendra_dehaani_KC144616.1
Scolopendra_s_mutilans_mu-JZ722846
Scolopendra_alternans_GASK01000049
Scolopendra_dehaani_KC144677.1
Scolopendra_dehaani_KC144088.1
Scolopendra_dehaani_KC144171.1
Scolopendra_s_mutilans_JN646114
Scolopendra_s_mutilans_JZ722845
Scolopendra_subspinipes_11442_c0_g1_i1
Scolopendra_dehaani_KC145111.1
Scolopendra_dehaani_KC144338.1
Scolopendra_dehaani_KC145071.1
Scolopendra_s_mutilans_JZ722844
Scolopendra_s_mutilans_JZ722844
Scolopendra_s_mutilans_JQ757070.1
Scolopendra_s_mutilans_JQ757072.1
Scolopendra_s_mutilans_JQ757071.1
Scolopendra_subspinipes_18290_c0_g1_i1
Scolopendra_subspinipes_18290_c0_g1_i2
Scolopendra_s_mutilans_JZ722843
Scolopendra_s_mutilans_JQ757069.1
0.1
Min
Max
0
90518
FPKM

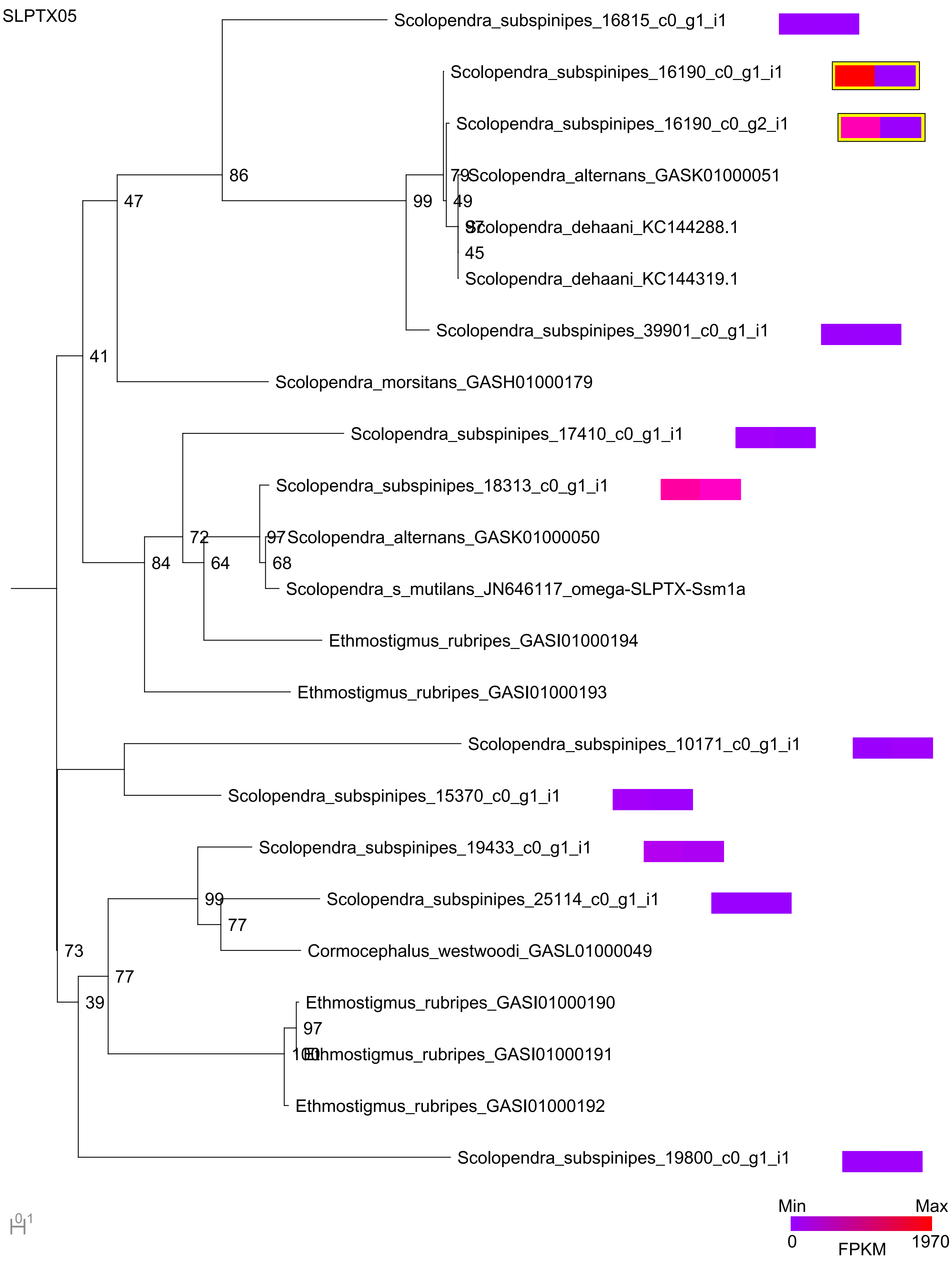
SLPTX05
Scolopendra_subspinipes_16815_c0_g1_i1
Scolopendra_subspinipes_16190_c0_g1_i1
Scolopendra_subspinipes_16190_c0_g2_i1
Scolopendra_alternans_GASK01000051
Scolopendra_dehaani_KC144288.1
Scolopendra_dehaani_KC144319.1
Scolopendra_subspinipes_39901_c0_g1_i1
Scolopendra_morsitans_GASH01000179
Scolopendra_subspinipes_17410_c0_g1_i1
Scolopendra_subspinipes_18313_c0_g1_i1
Scolopendra_alternans_GASK01000050
Scolopendra_s_mutilans_JN646117_omega-SLPTX-Ssm1a
Ethmostigmus_rubripes_GASI01000194
Ethmostigmus_rubripes_GASI01000193
Scolopendra_subspinipes_10171_c0_g1_i1
Scolopendra_subspinipes_15370_c0_g1_i1
Scolopendra_subspinipes_19433_c0_g1_i1
Scolopendra_subspinipes_25114_c0_g1_i1
Cormocephalus_westwoodi_GASL01000049
Ethmostigmus_rubripes_GASI01000190
Ethmostigmus_rubripes_GASI01000191
Ethmostigmus_rubripes_GASI01000192
Scolopendra_subspinipes_19800_c0_g1_i1
86
47
41
99
79
49
97
45
72
97
84
64
68
99
77
73
77
39
97
100
0.1
Min
Max
0
FPKM
1970

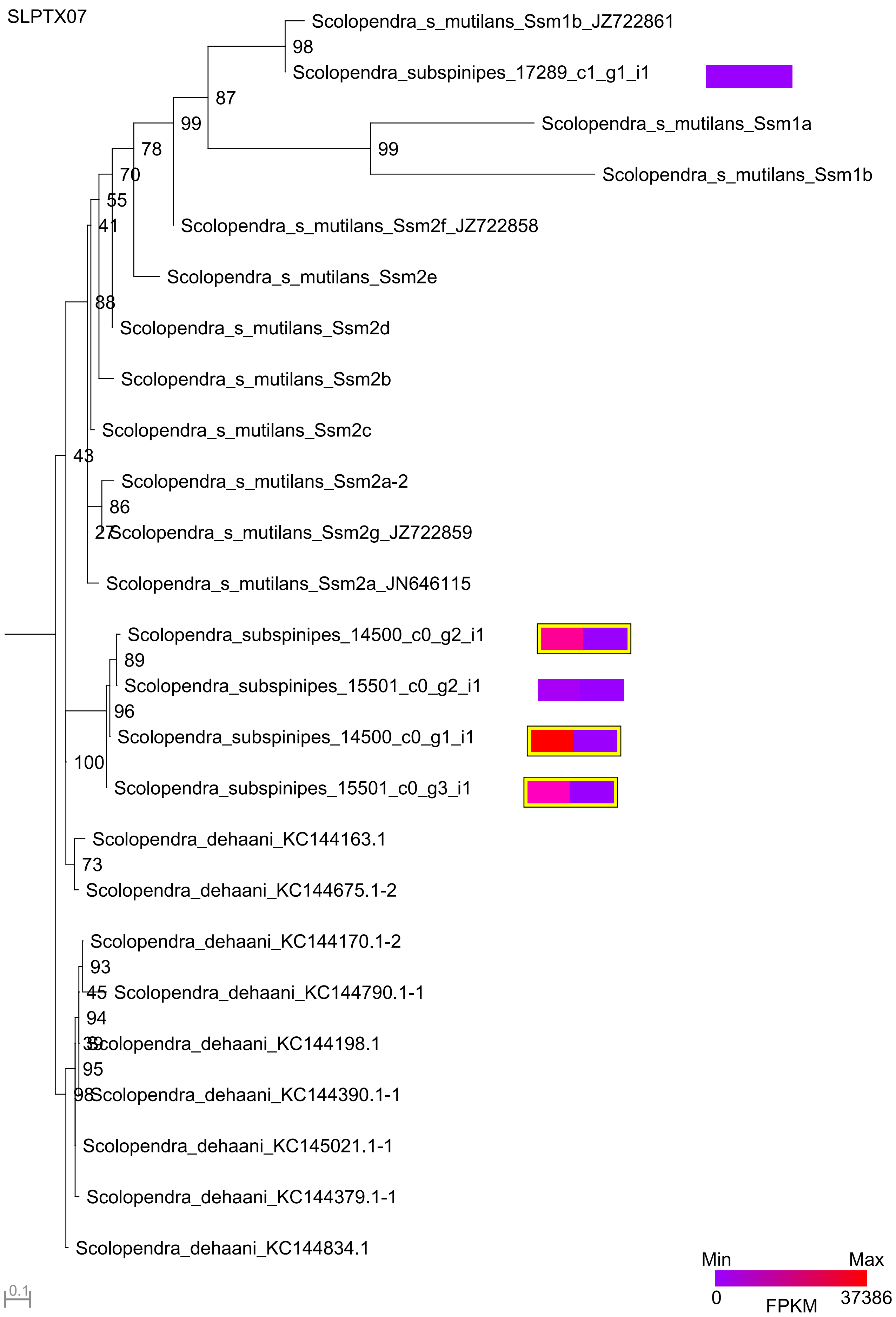

SLPTX07
Scolopendra_s_mutilans_Ssm1b_JZ722861
98
Scolopendra_subspinipes_17289_c1_g1_i1
87
Scolopendra_s_mutilans_Ssm1a
99
78
99
Scolopendra_s_mutilans_Ssm1b
70
55
41
Scolopendra_s_mutilans_Ssm2f_JZ722858
Scolopendra_s_mutilans_Ssm2e
88
Scolopendra_s_mutilans_Ssm2d
Scolopendra_s_mutilans_Ssm2b
Scolopendra_s_mutilans_Ssm2c
43
Scolopendra_s_mutilans_Ssm2a-2
86
27
Scolopendra_s_mutilans_Ssm2g_JZ722859
Scolopendra_s_mutilans_Ssm2a_JN646115
Scolopendra_subspinipes_14500_c0_g2_i1
89
Scolopendra_subspinipes_15501_c0_g2_i1
96
Scolopendra_subspinipes_14500_c0_g1_i1
100
Scolopendra_subspinipes_15501_c0_g3_i1
Scolopendra_dehaani_KC144163.1
73
Scolopendra_dehaani_KC144675.1-2
Scolopendra_dehaani_KC144170.1-2
93
45
Scolopendra_dehaani_KC144790.1-1
94
39
Scolopendra_dehaani_KC144198.1
95
98
Scolopendra_dehaani_KC144390.1-1
Scolopendra_dehaani_KC145021.1-1
Scolopendra_dehaani_KC144379.1-1
Scolopendra_dehaani_KC144834.1
0.1
Min
Max
0
FPKM
37386

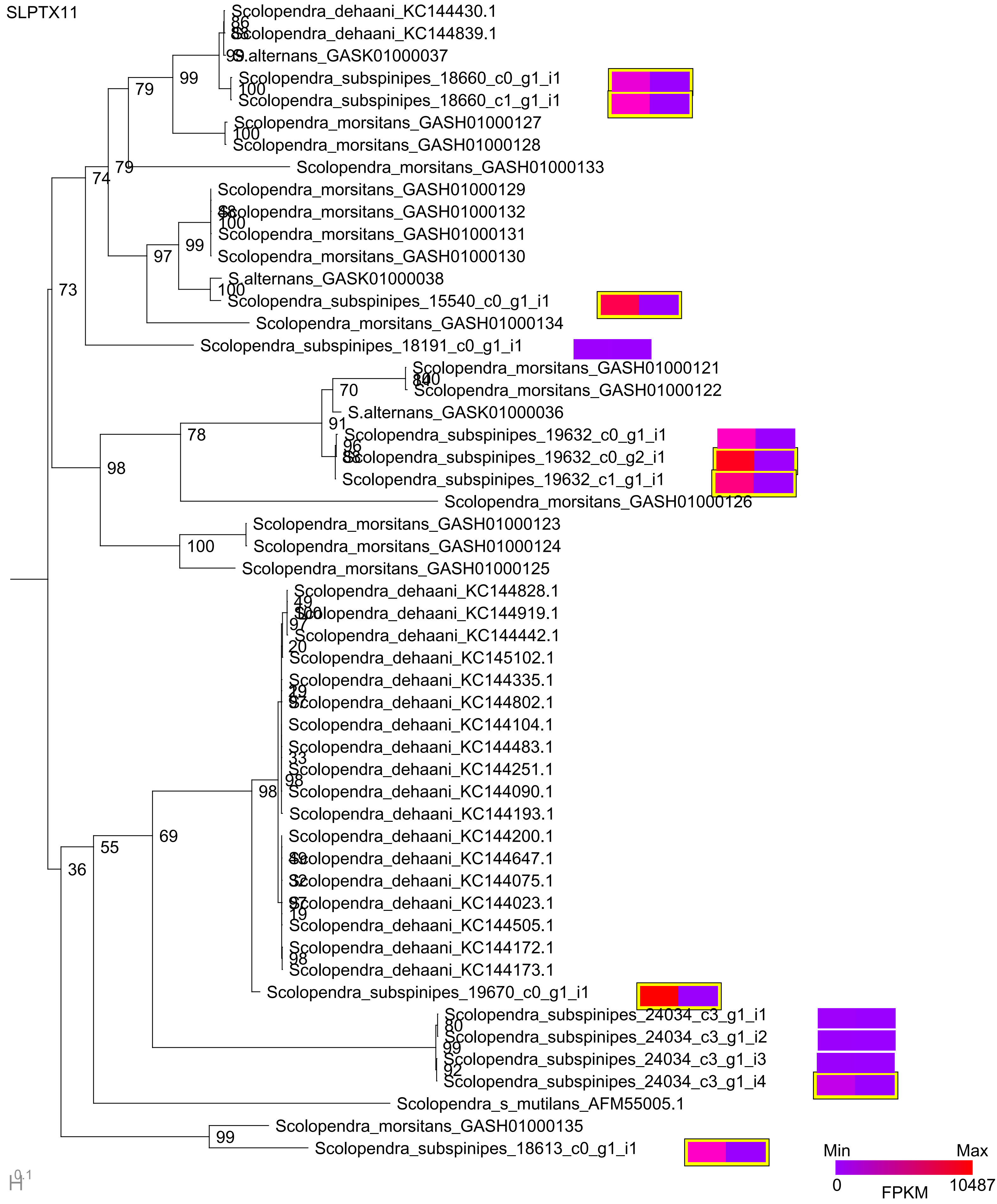
SLPTX11
Scolopendra_dehaani_KC144430.1
Scolopendra_dehaani_KC144839.1
S.alternans_GASK01000037
Scolopendra_subspinipes_18660_c0_g1_i1
Scolopendra_subspinipes_18660_c1_g1_i1
Scolopendra_morsitans_GASH01000127
Scolopendra_morsitans_GASH01000128
Scolopendra_morsitans_GASH01000133
Scolopendra_morsitans_GASH01000129
Scolopendra_morsitans_GASH01000132
Scolopendra_morsitans_GASH01000131
Scolopendra_morsitans_GASH01000130
S.alternans_GASK01000038
Scolopendra_subspinipes_15540_c0_g1_i1
Scolopendra_morsitans_GASH01000134
Scolopendra_subspinipes_18191_c0_g1_i1
Scolopendra_morsitans_GASH01000121
Scolopendra_morsitans_GASH01000122
S.alternans_GASK01000036
Scolopendra_subspinipes_19632_c0_g1_i1
Scolopendra_subspinipes_19632_c0_g2_i1
Scolopendra_subspinipes_19632_c1_g1_i1
Scolopendra_morsitans_GASH01000126
Scolopendra_morsitans_GASH01000123
Scolopendra_morsitans_GASH01000124
Scolopendra_morsitans_GASH01000125
Scolopendra_dehaani_KC144828.1
Scolopendra_dehaani_KC144919.1
Scolopendra_dehaani_KC144442.1
Scolopendra_dehaani_KC145102.1
Scolopendra_dehaani_KC144335.1
Scolopendra_dehaani_KC144802.1
Scolopendra_dehaani_KC144104.1
Scolopendra_dehaani_KC144483.1
Scolopendra_dehaani_KC144251.1
Scolopendra_dehaani_KC144090.1
Scolopendra_dehaani_KC144193.1
Scolopendra_dehaani_KC144200.1
Scolopendra_dehaani_KC144647.1
Scolopendra_dehaani_KC144075.1
Scolopendra_dehaani_KC144023.1
Scolopendra_dehaani_KC144505.1
Scolopendra_dehaani_KC144172.1
Scolopendra_dehaani_KC144173.1
Scolopendra_subspinipes_19670_c0_g1_i1
Scolopendra_subspinipes_24034_c3_g1_i1
Scolopendra_subspinipes_24034_c3_g1_i2
Scolopendra_subspinipes_24034_c3_g1_i3
Scolopendra_subspinipes_24034_c3_g1_i4
Scolopendra_s_mutilans_AFM55005.1
Scolopendra_morsitans_GASH01000135
Scolopendra_subspinipes_18613_c0_g1_i1
Min
Max
0
FPKM
10487
0.1

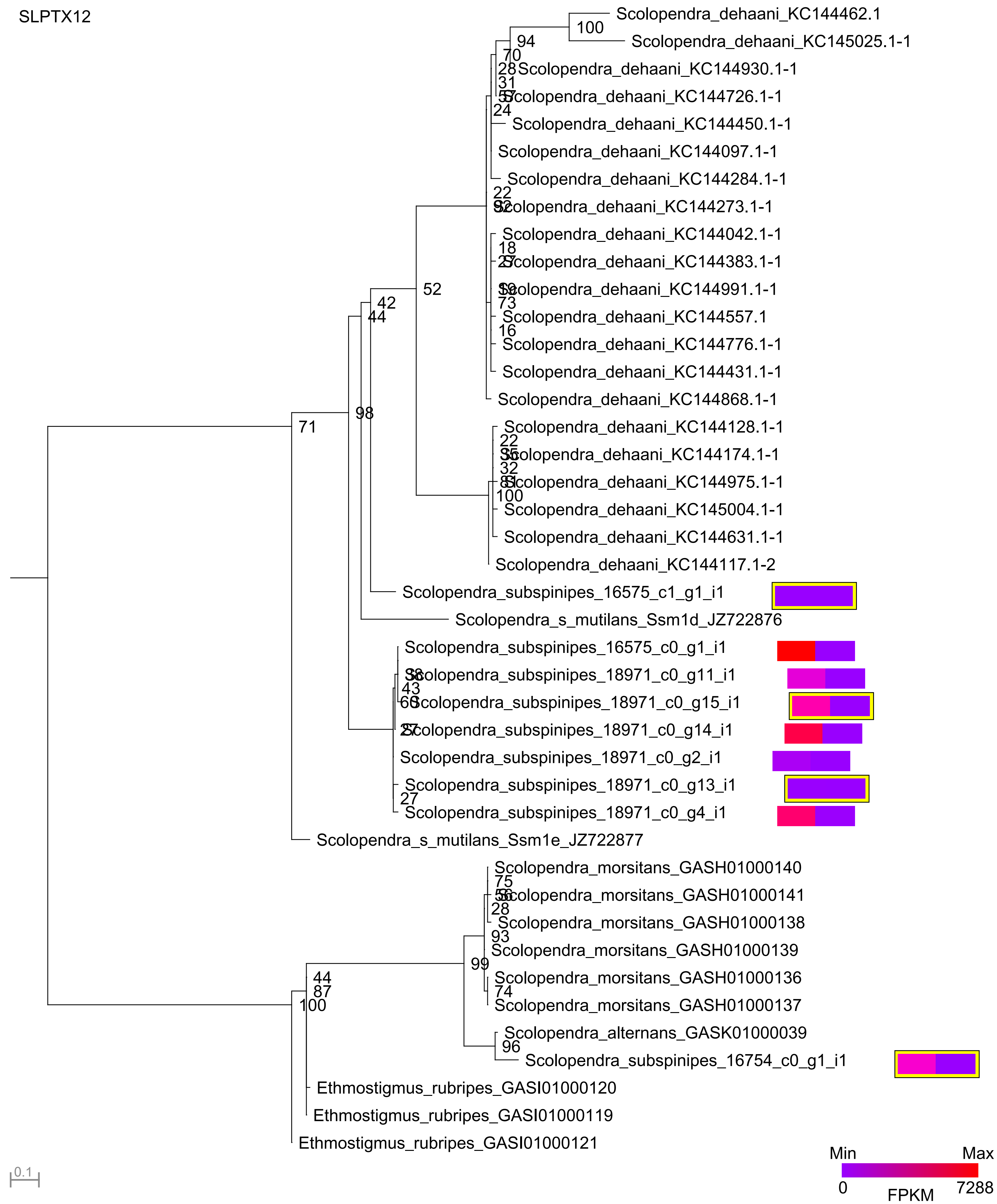

SLPTX12
Scolopendra_dehaani_KC144462.1
Scolopendra_dehaani_KC145025.1-1
Scolopendra_dehaani_KC144930.1-1
Scolopendra_dehaani_KC144726.1-1
Scolopendra_dehaani_KC144450.1-1
Scolopendra_dehaani_KC144097.1-1
Scolopendra_dehaani_KC144284.1-1
Scolopendra_dehaani_KC144273.1-1
Scolopendra_dehaani_KC144042.1-1
Scolopendra_dehaani_KC144383.1-1
Scolopendra_dehaani_KC144991.1-1
Scolopendra_dehaani_KC144557.1
Scolopendra_dehaani_KC144776.1-1
Scolopendra_dehaani_KC144431.1-1
Scolopendra_dehaani_KC144868.1-1
Scolopendra_dehaani_KC144128.1-1
Scolopendra_dehaani_KC144174.1-1
Scolopendra_dehaani_KC144975.1-1
Scolopendra_dehaani_KC145004.1-1
Scolopendra_dehaani_KC144631.1-1
Scolopendra_dehaani_KC144117.1-2
Scolopendra_subspinipes_16575_c1_g1_i1
Scolopendra_s_mutilans_Ssm1d_JZ722876
Scolopendra_subspinipes_16575_c0_g1_i1
Scolopendra_subspinipes_18971_c0_g11_i1
Scolopendra_subspinipes_18971_c0_g15_i1
Scolopendra_subspinipes_18971_c0_g14_i1
Scolopendra_subspinipes_18971_c0_g2_i1
Scolopendra_subspinipes_18971_c0_g13_i1
Scolopendra_subspinipes_18971_c0_g4_i1
Scolopendra_s_mutilans_Ssm1e_JZ722877
Scolopendra_morsitans_GASH01000140
Scolopendra_morsitans_GASH01000141
Scolopendra_morsitans_GASH01000138
Scolopendra_morsitans_GASH01000139
Scolopendra_morsitans_GASH01000136
Scolopendra_morsitans_GASH01000137
Scolopendra_alternans_GASK01000039
Scolopendra_subspinipes_16754_c0_g1_i1
Ethmostigmus_rubripes_GASI01000120
Ethmostigmus_rubripes_GASI01000119
Ethmostigmus_rubripes_GASI01000121
0.1
Min
Max
0
7288
FPKM

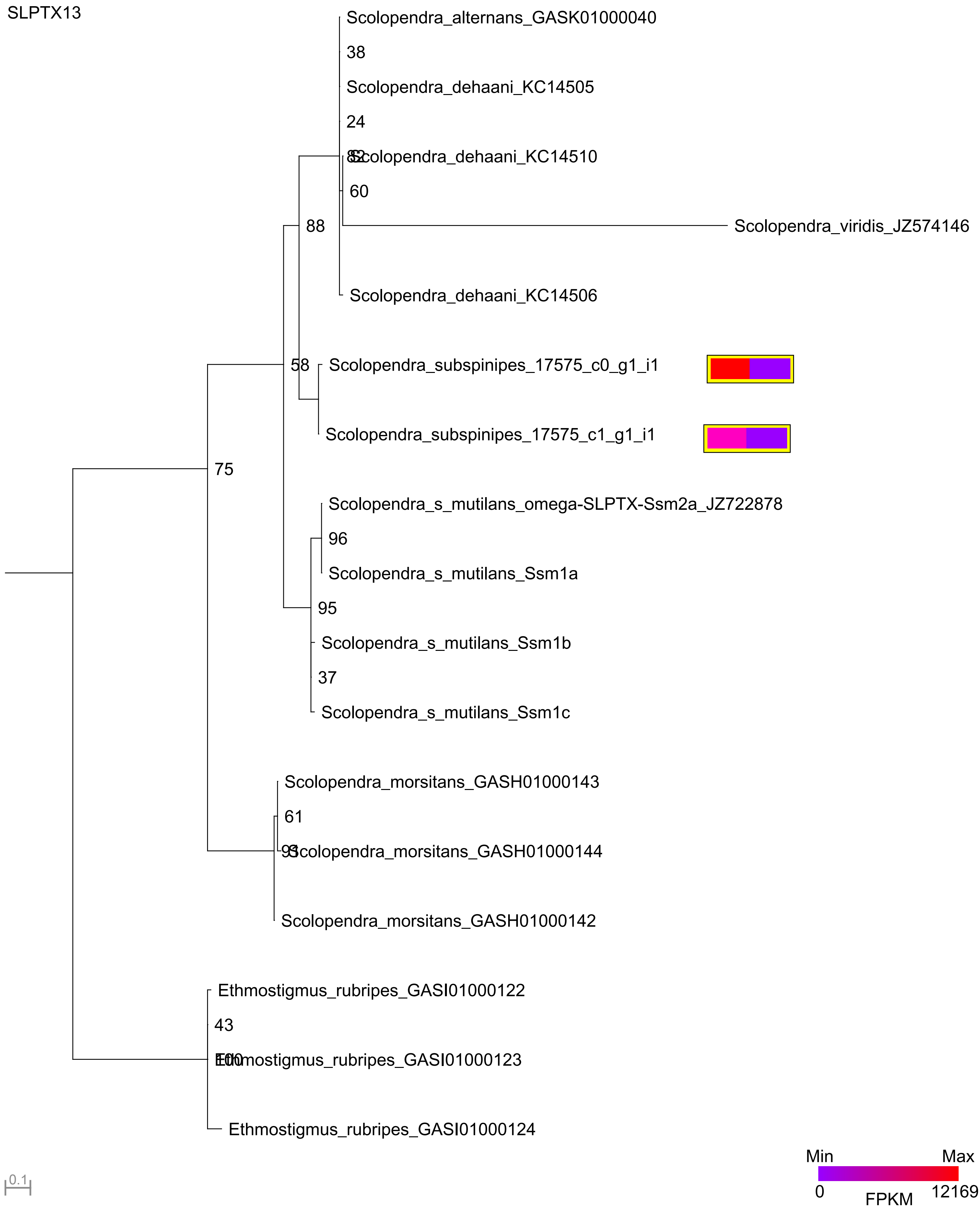
SLPTX13
Scolopendra_alternans_GASK01000040
38
Scolopendra_dehaani_KC14505
24
Scolopendra_dehaani_KC14510
60
Scolopendra_viridis_JZ574146
88
Scolopendra_dehaani_KC14506
58
Scolopendra_subspinipes_17575_c0_g1_i1
Scolopendra_subspinipes_17575_c1_g1_i1
75
Scolopendra_s_mutilans_omega-SLPTX-Ssm2a_JZ722878
96
Scolopendra_s_mutilans_Ssm1a
95
Scolopendra_s_mutilans_Ssm1b
37
Scolopendra_s_mutilans_Ssm1c
Scolopendra_morsitans_GASH01000143
61
Scolopendra_morsitans_GASH01000144
Scolopendra_morsitans_GASH01000142
Ethmostigmus_rubripes_GASI01000122
43
Ethmostigmus_rubripes_GASI01000123
Ethmostigmus_rubripes_GASI01000124
0.1
Min
Max
0
FPKM
12169

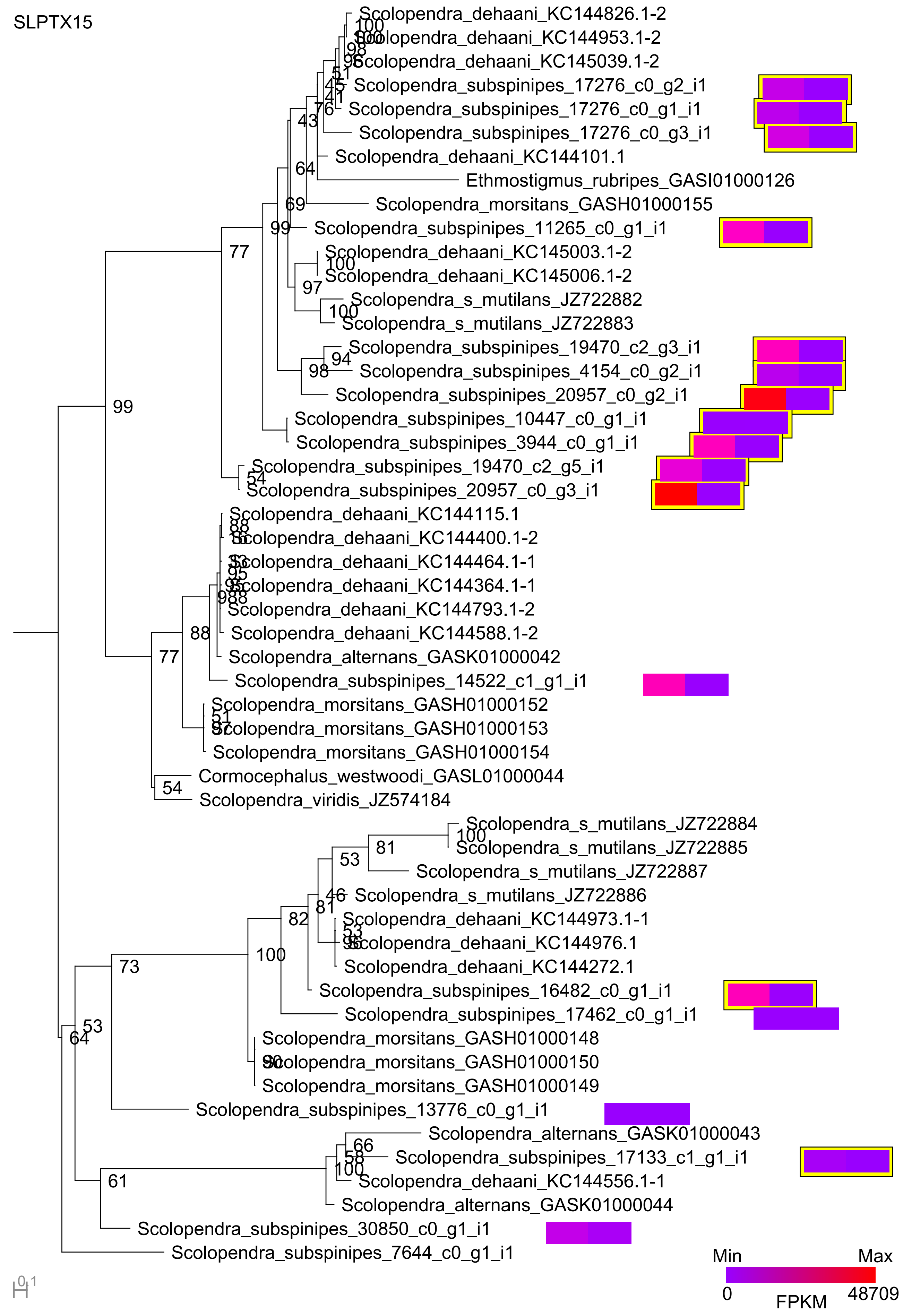
SLPTX15
Scolopendra_dehaani_KC144826.1-2
Scolopendra_dehaani_KC144953.1-2
Scolopendra_dehaani_KC145039.1-2
Scolopendra_subspinipes_17276_c0_g2_i1
Scolopendra_subspinipes_17276_c0_g1_i1
Scolopendra_subspinipes_17276_c0_g3_i1
Scolopendra_dehaani_KC144101.1
Ethmostigmus_rubripes_GASI01000126
Scolopendra_morsitans_GASH01000155
Scolopendra_subspinipes_11265_c0_g1_i1
Scolopendra_dehaani_KC145003.1-2
Scolopendra_dehaani_KC145006.1-2
Scolopendra_s_mutilans_JZ722882
Scolopendra_s_mutilans_JZ722883
Scolopendra_subspinipes_19470_c2_g3_i1
Scolopendra_subspinipes_4154_c0_g2_i1
Scolopendra_subspinipes_20957_c0_g2_i1
Scolopendra_subspinipes_10447_c0_g1_i1
Scolopendra_subspinipes_3944_c0_g1_i1
Scolopendra_subspinipes_19470_c2_g5_i1
Scolopendra_subspinipes_20957_c0_g3_i1
Scolopendra_dehaani_KC144115.1
Scolopendra_dehaani_KC144400.1-2
Scolopendra_dehaani_KC144464.1-1
Scolopendra_dehaani_KC144364.1-1
Scolopendra_dehaani_KC144793.1-2
Scolopendra_dehaani_KC144588.1-2
Scolopendra_alternans_GASK01000042
Scolopendra_subspinipes_14522_c1_g1_i1
Scolopendra_morsitans_GASH01000152
Scolopendra_morsitans_GASH01000153
Scolopendra_morsitans_GASH01000154
Cormocephalus_westwoodi_GASL01000044
Scolopendra_viridis_JZ574184
Scolopendra_s_mutilans_JZ722884
Scolopendra_s_mutilans_JZ722885
Scolopendra_s_mutilans_JZ722887
Scolopendra_s_mutilans_JZ722886
Scolopendra_dehaani_KC144973.1-1
Scolopendra_dehaani_KC144976.1
Scolopendra_dehaani_KC144272.1
Scolopendra_subspinipes_16482_c0_g1_i1
Scolopendra_subspinipes_17462_c0_g1_i1
Scolopendra_morsitans_GASH01000148
Scolopendra_morsitans_GASH01000150
Scolopendra_morsitans_GASH01000149
Scolopendra_subspinipes_13776_c0_g1_i1
Scolopendra_alternans_GASK01000043
Scolopendra_subspinipes_17133_c1_g1_i1
Scolopendra_dehaani_KC144556.1-1
Scolopendra_alternans_GASK01000044
Scolopendra_subspinipes_30850_c0_g1_i1
Scolopendra_subspinipes_7644_c0_g1_i1
0.1
Min
Max
0
FPKM
48709

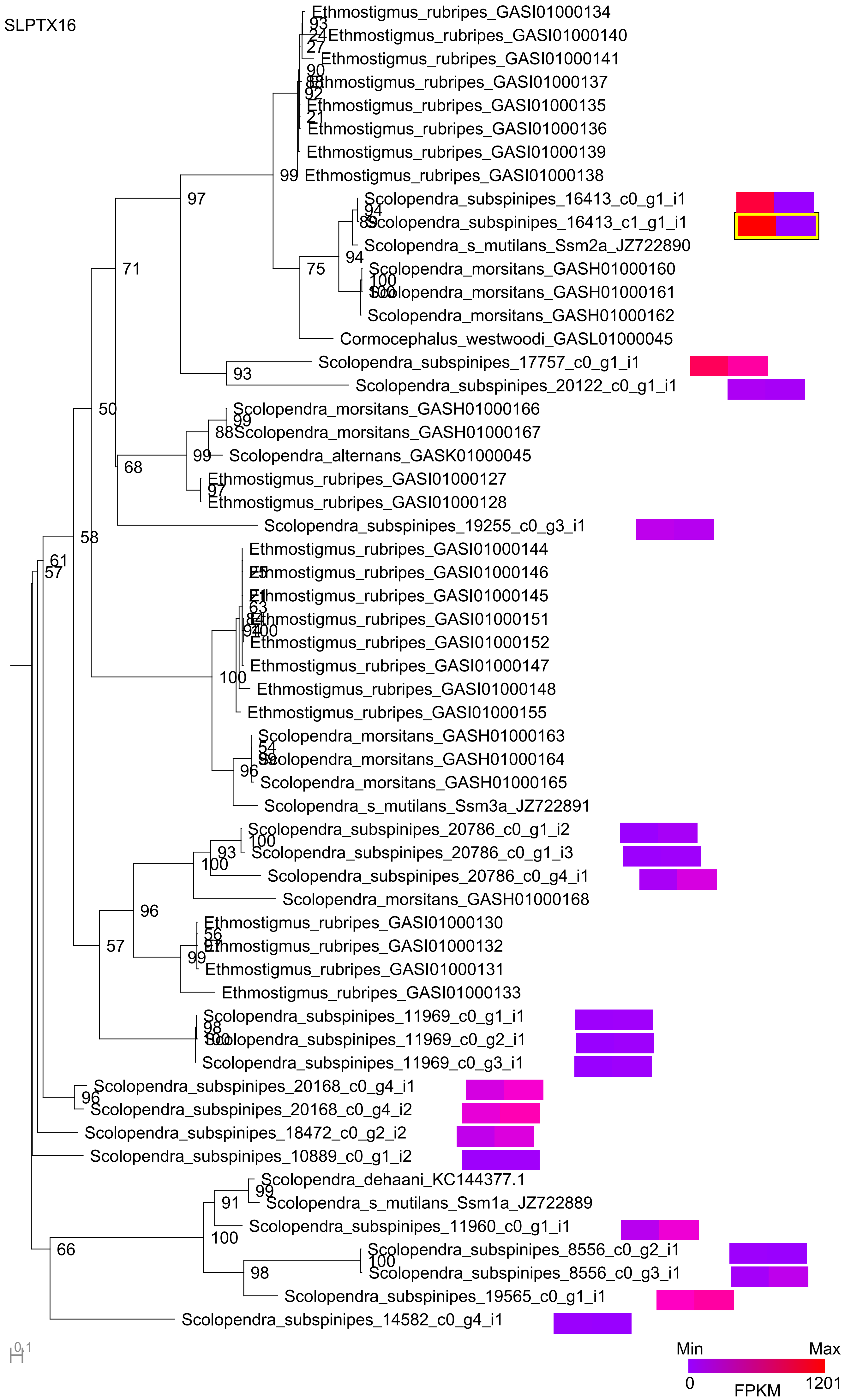
SLPTX16
Ethmostigmus_rubripes_GASI01000134
Ethmostigmus_rubripes_GASI01000140
Ethmostigmus_rubripes_GASI01000141
Ethmostigmus_rubripes_GASI01000137
Ethmostigmus_rubripes_GASI01000135
Ethmostigmus_rubripes_GASI01000136
Ethmostigmus_rubripes_GASI01000139
Ethmostigmus_rubripes_GASI01000138
Scolopendra_subspinipes_16413_c0_g1_i1
Scolopendra_subspinipes_16413_c1_g1_i1
Scolopendra_s_mutilans_Ssm2a_JZ722890
Scolopendra_morsitans_GASH01000160
Scolopendra_morsitans_GASH01000161
Scolopendra_morsitans_GASH01000162
Cormocephalus_westwoodi_GASL01000045
Scolopendra_subspinipes_17757_c0_g1_i1
Scolopendra_subspinipes_20122_c0_g1_i1
Scolopendra_morsitans_GASH01000166
Scolopendra_morsitans_GASH01000167
Scolopendra_alternans_GASK01000045
Ethmostigmus_rubripes_GASI01000127
Ethmostigmus_rubripes_GASI01000128
Scolopendra_subspinipes_19255_c0_g3_i1
Ethmostigmus_rubripes_GASI01000144
Ethmostigmus_rubripes_GASI01000146
Ethmostigmus_rubripes_GASI01000145
Ethmostigmus_rubripes_GASI01000151
Ethmostigmus_rubripes_GASI01000152
Ethmostigmus_rubripes_GASI01000147
Ethmostigmus_rubripes_GASI01000148
Ethmostigmus_rubripes_GASI01000155
Scolopendra_morsitans_GASH01000163
Scolopendra_morsitans_GASH01000164
Scolopendra_morsitans_GASH01000165
Scolopendra_s_mutilans_Ssm3a_JZ722891
Scolopendra_subspinipes_20786_c0_g1_i2
Scolopendra_subspinipes_20786_c0_g1_i3
Scolopendra_subspinipes_20786_c0_g4_i1
Scolopendra_morsitans_GASH01000168
Ethmostigmus_rubripes_GASI01000130
Ethmostigmus_rubripes_GASI01000132
Ethmostigmus_rubripes_GASI01000131
Ethmostigmus_rubripes_GASI01000133
Scolopendra_subspinipes_11969_c0_g1_i1
Scolopendra_subspinipes_11969_c0_g2_i1
Scolopendra_subspinipes_11969_c0_g3_i1
Scolopendra_subspinipes_20168_c0_g4_i1
Scolopendra_subspinipes_20168_c0_g4_i2
Scolopendra_subspinipes_18472_c0_g2_i2
Scolopendra_subspinipes_10889_c0_g1_i2
Scolopendra_dehaani_KC144377.1
Scolopendra_s_mutilans_Ssm1a_JZ722889
Scolopendra_subspinipes_11960_c0_g1_i1
Scolopendra_subspinipes_8556_c0_g2_i1
Scolopendra_subspinipes_8556_c0_g3_i1
Scolopendra_subspinipes_19565_c0_g1_i1
Scolopendra_subspinipes_14582_c0_g4_i1
0.1
Min
Max
0
FPKM
1201

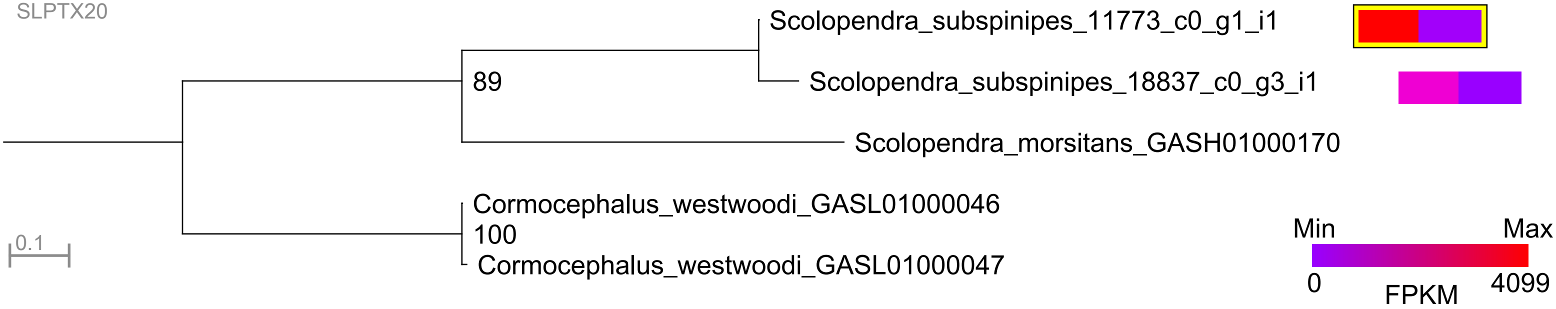
SLPTX20
Scolopendra_subspinipes_11773_c0_g1_i1
89
Scolopendra_subspinipes_18837_c0_g3_i1
Scolopendra_morsitans_GASH01000170
Cormocephalus_westwoodi_GASL01000046
100
Cormocephalus_westwoodi_GASL01000047
0.1
Min
Max
0
4099
FPKM

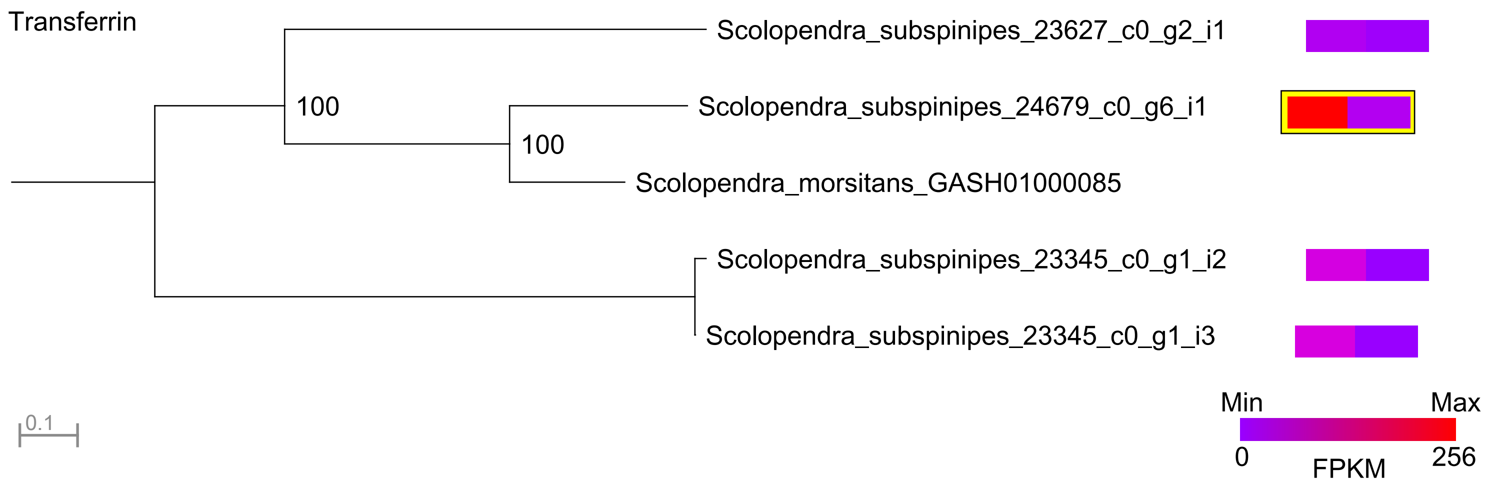
Transferrin
Scolopendra_subspinipes_23627_c0_g2_i1
100
Scolopendra_subspinipes_24679_c0_g6_i1
100
Scolopendra_morsitans_GASH01000085
Scolopendra_subspinipes_23345_c0_g1_i2
Scolopendra_subspinipes_23345_c0_g1_i3
0.1
Min
Max
0
256
FPKM

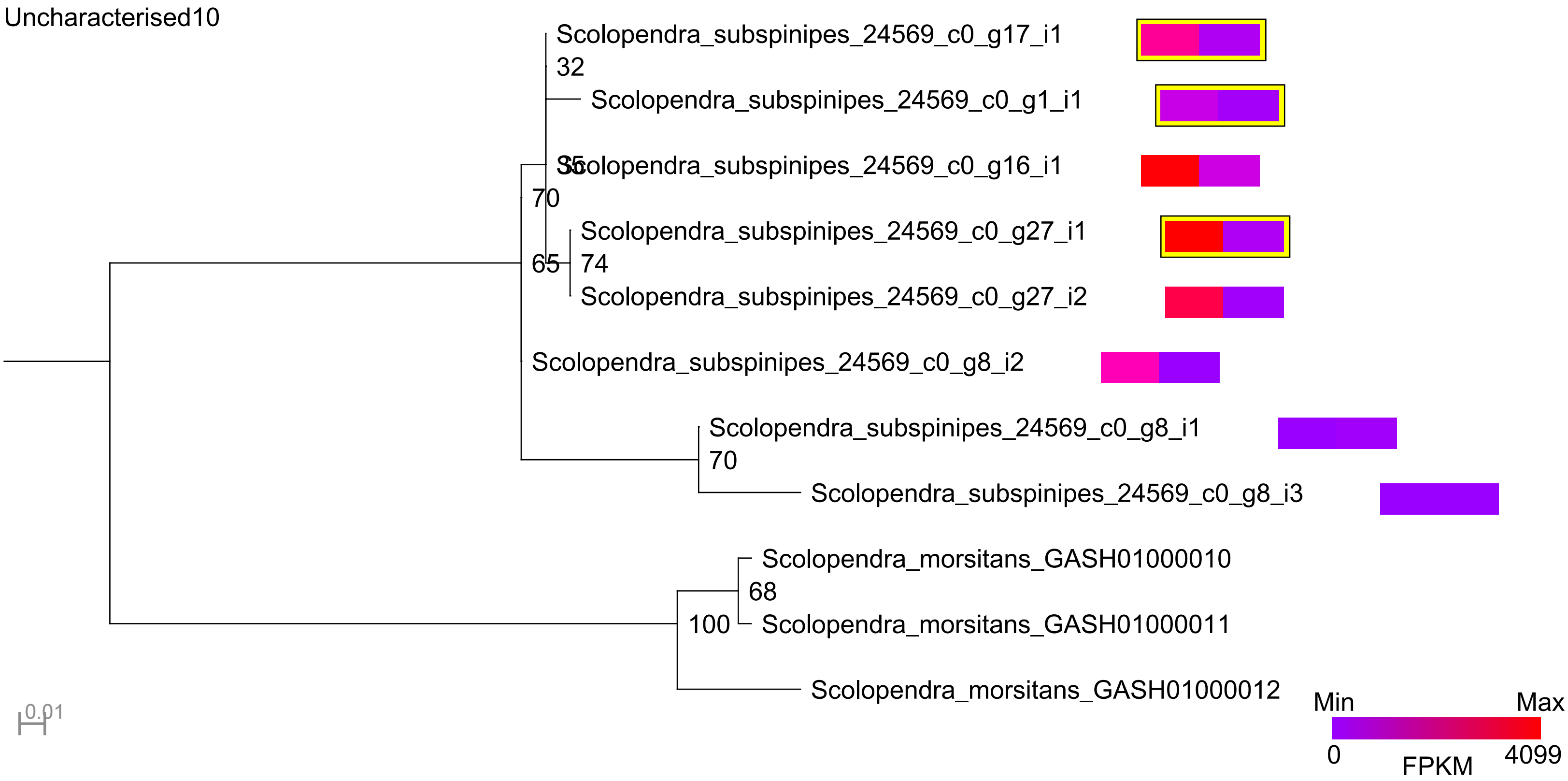

Uncharacterised10
Scolopendra_subspinipes_24569_c0_g17_i1
32
Scolopendra_subspinipes_24569_c0_g1_i1
35
Scolopendra_subspinipes_24569_c0_g16_i1
70
Scolopendra_subspinipes_24569_c0_g27_i1
65
74
Scolopendra_subspinipes_24569_c0_g27_i2
Scolopendra_subspinipes_24569_c0_g8_i2
Scolopendra_subspinipes_24569_c0_g8_i1
70
Scolopendra_subspinipes_24569_c0_g8_i3
Scolopendra_morsitans_GASH01000010
68
100
Scolopendra_morsitans_GASH01000011
Scolopendra_morsitans_GASH01000012
0.01
Min
Max
0
4099
FPKM

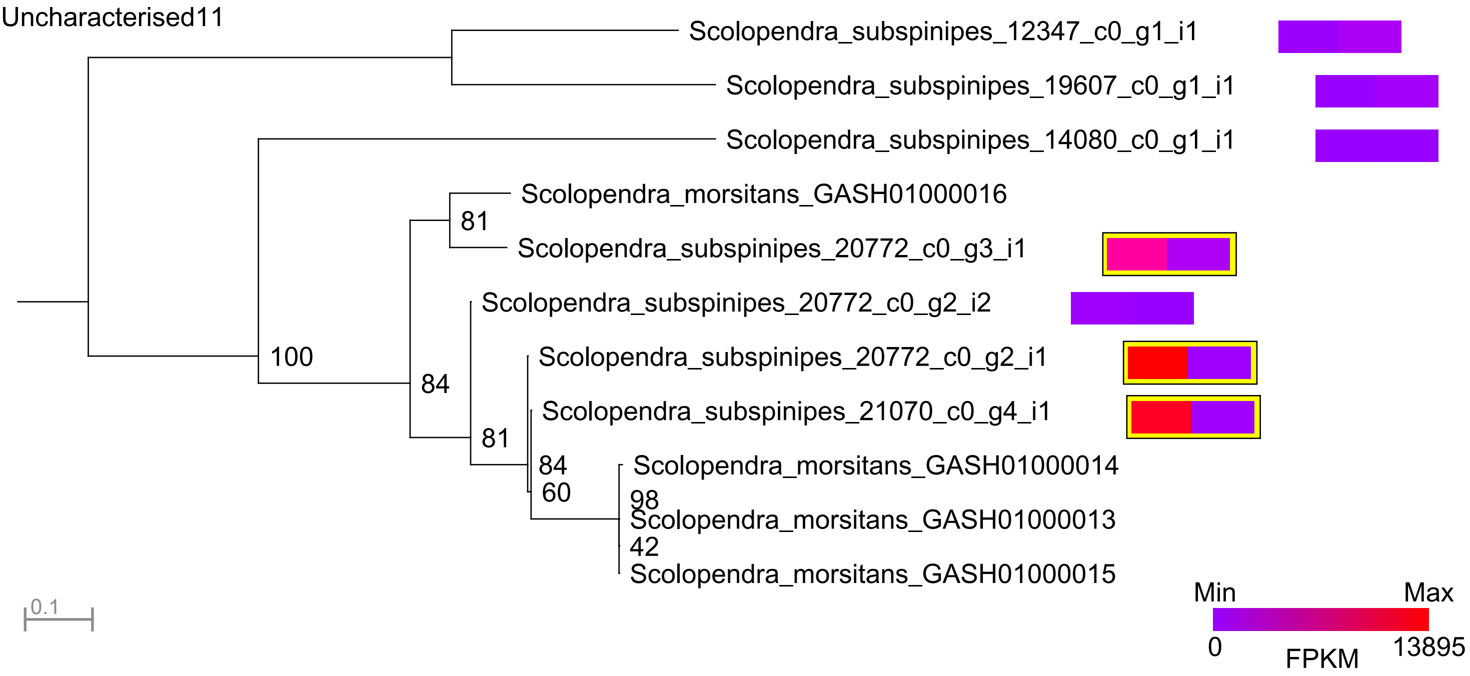
Uncharacterised11
Scolopendra_subspinipes_12347_c0_g1_i1
Scolopendra_subspinipes_19607_c0_g1_i1
Scolopendra_subspinipes_14080_c0_g1_i1
Scolopendra_morsitans_GASH01000016
81
Scolopendra_subspinipes_20772_c0_g3_i1
Scolopendra_subspinipes_20772_c0_g2_i2
100
Scolopendra_subspinipes_20772_c0_g2_i1
84
Scolopendra_subspinipes_21070_c0_g4_i1
81
Scolopendra_morsitans_GASH01000014
84
60
98
Scolopendra_morsitans_GASH01000013
42
Scolopendra_morsitans_GASH01000015
0.1
Min
Max
0
FPKM
13895

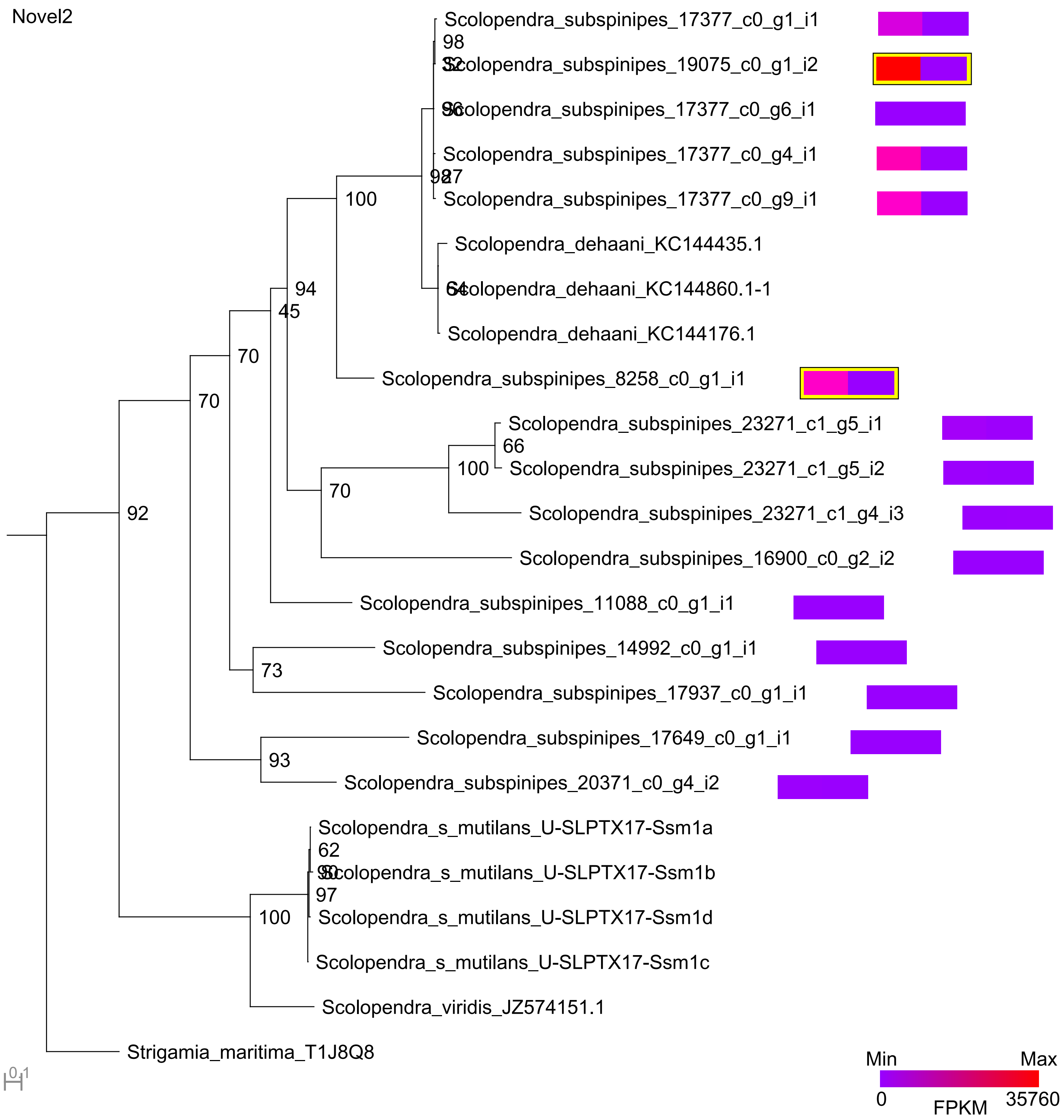

Novel2
Scolopendra_subspinipes_17377_c0_g1_i1
98
32
Scolopendra_subspinipes_19075_c0_g1_i2
96
Scolopendra_subspinipes_17377_c0_g6_i1
Scolopendra_subspinipes_17377_c0_g4_i1
92
27
Scolopendra_subspinipes_17377_c0_g9_i1
100
Scolopendra_dehaani_KC144435.1
94
64
Scolopendra_dehaani_KC144860.1-1
45
Scolopendra_dehaani_KC144176.1
70
Scolopendra_subspinipes_8258_c0_g1_i1
70
Scolopendra_subspinipes_23271_c1_g5_i1
66
100
Scolopendra_subspinipes_23271_c1_g5_i2
70
Scolopendra_subspinipes_23271_c1_g4_i3
92
Scolopendra_subspinipes_16900_c0_g2_i2
Scolopendra_subspinipes_11088_c0_g1_i1
Scolopendra_subspinipes_14992_c0_g1_i1
73
Scolopendra_subspinipes_17937_c0_g1_i1
Scolopendra_subspinipes_17649_c0_g1_i1
93
Scolopendra_subspinipes_20371_c0_g4_i2
Scolopendra_s_mutilans_U-SLPTX17-Ssm1a
62
93
Scolopendra_s_mutilans_U-SLPTX17-Ssm1b
97
Scolopendra_s_mutilans_U-SLPTX17-Ssm1d
100
Scolopendra_s_mutilans_U-SLPTX17-Ssm1c
Scolopendra_viridis_JZ574151.1
Strigamia_maritima_T1J8Q8
0.1
Min
Max
0
FPKM
35760

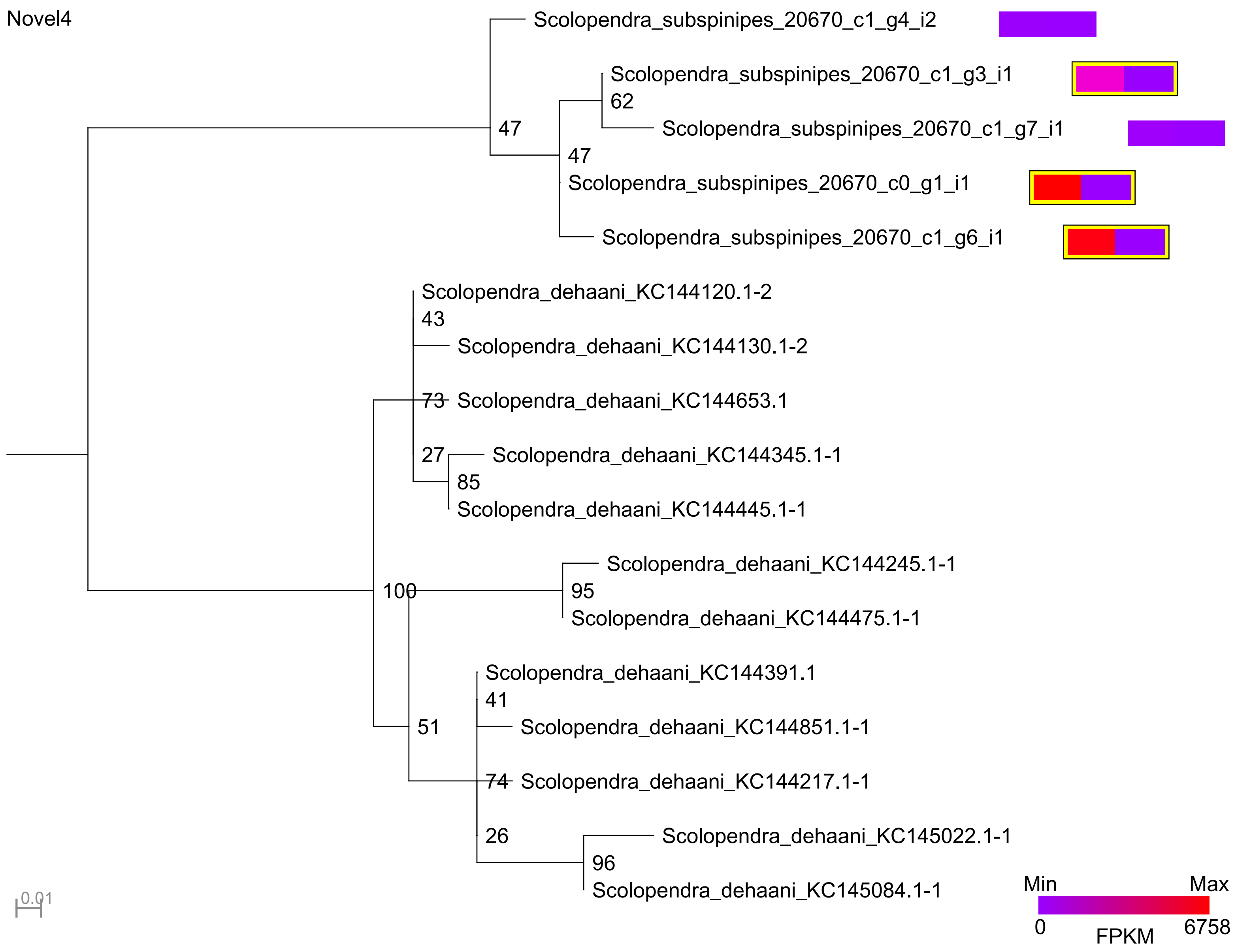

Novel4
Scolopendra_subspinipes_20670_c1_g4_i2
Scolopendra_subspinipes_20670_c1_g3_i1
62
Scolopendra_subspinipes_20670_c1_g7_i1
47
47
Scolopendra_subspinipes_20670_c0_g1_i1
Scolopendra_subspinipes_20670_c1_g6_i1
Scolopendra_dehaani_KC144120.1-2
43
Scolopendra_dehaani_KC144130.1-2
73
Scolopendra_dehaani_KC144653.1
27
Scolopendra_dehaani_KC144345.1-1
85
Scolopendra_dehaani_KC144445.1-1
Scolopendra_dehaani_KC144245.1-1
100
95
Scolopendra_dehaani_KC144475.1-1
Scolopendra_dehaani_KC144391.1
41
51
Scolopendra_dehaani_KC144851.1-1
74
Scolopendra_dehaani_KC144217.1-1
26
Scolopendra_dehaani_KC145022.1-1
96
Scolopendra_dehaani_KC145084.1-1
0.01
Min
Max
0
6758
FPKM

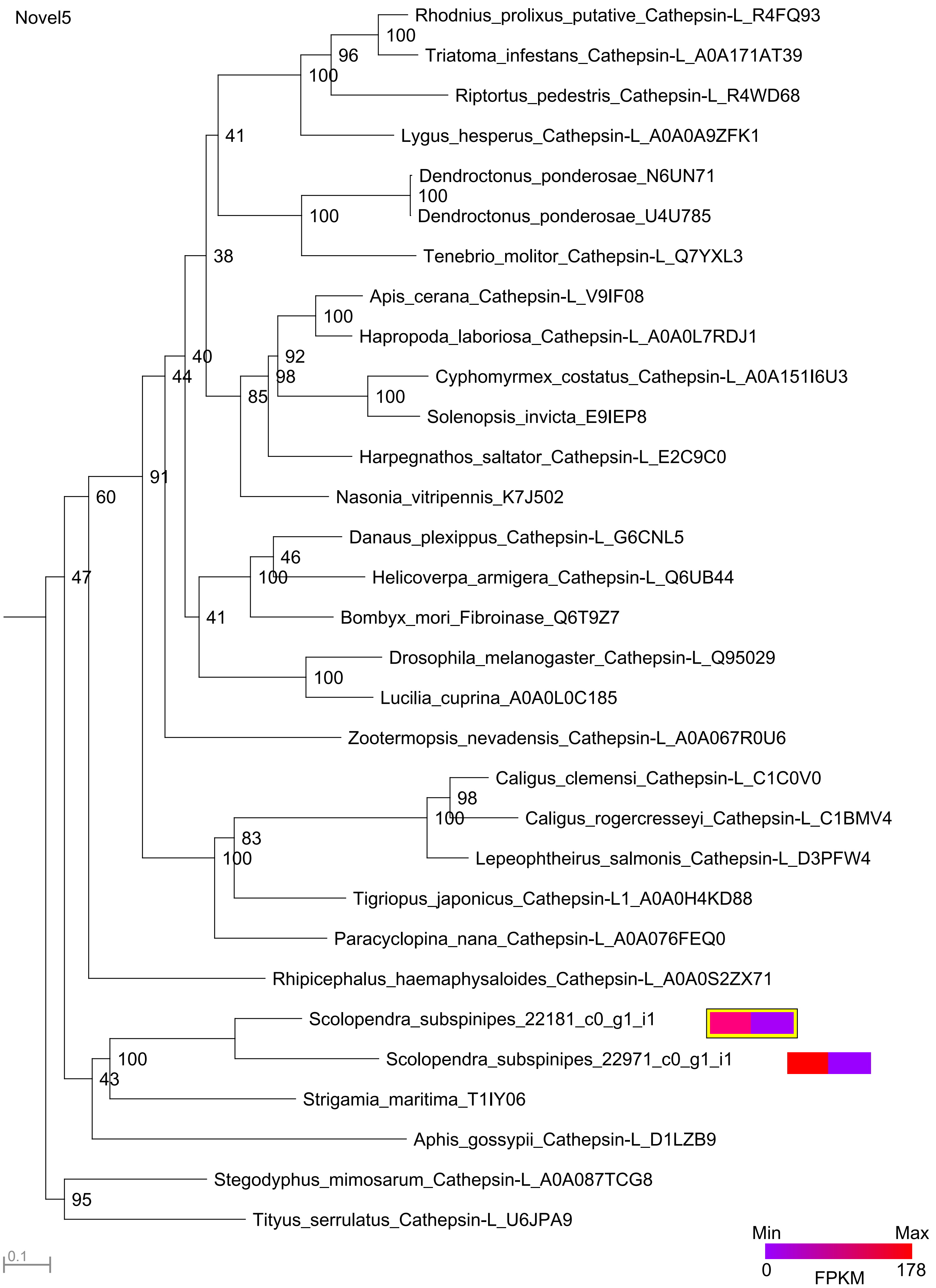
Novel5
Rhodnius_prolixus_putative_Cathepsin-L_R4FQ93
100
Triatoma_infestans_Cathepsin-L_A0A171AT39
96
100
Riptortus_pedestris_Cathepsin-L_R4WD68
41
Lygus_hesperus_Cathepsin-L_A0A0A9ZFK1
Dendroctonus_ponderosae_N6UN71
100
Dendroctonus_ponderosae_U4U785
100
Tenebrio_molitor_Cathepsin-L_Q7YXL3
38
Apis_cerana_Cathepsin-L_V9IF08
100
Hapropoda_laboriosa_Cathepsin-L_A0A0L7RDJ1
92
40
44
98
Cyphomyrmex_costatus_Cathepsin-L_A0A151I6U3
100
85
Solenopsis_invicta_E9IEP8
Harpegnathos_saltator_Cathepsin-L_E2C9C0
91
60
Nasonia_vitripennis_K7J502
Danaus_plexippus_Cathepsin-L_G6CNL5
46
47
100
Helicoverpa_armigera_Cathepsin-L_Q6UB44
41
Bombyx_mori_Fibroinase_Q6T9Z7
Drosophila_melanogaster_Cathepsin-L_Q95029
100
Lucilia_cuprina_A0A0L0C185
Zootermopsis_nevadensis_Cathepsin-L_A0A067R0U6
Caligus_clemensi_Cathepsin-L_C1C0V0
98
100
Caligus_rogercresseyi_Cathepsin-L_C1BMV4
83
100
Lepeophtheirus_salmonis_Cathepsin-L_D3PFW4
Tigriopus_japonicus_Cathepsin-L1_A0A0H4KD88
Paracyclopina_nana_Cathepsin-L_A0A076FEQ0
Rhipicephalus_haemaphysaloides_Cathepsin-L_A0A0S2ZX71
Scolopendra_subspinipes_22181_c0_g1_i1
100
Scolopendra_subspinipes_22971_c0_g1_i1
43
Strigamia_maritima_T1IY06
Aphis_gossypii_Cathepsin-L_D1LZB9
Stegodyphus_mimosarum_Cathepsin-L_A0A087TCG8
95
Tityus_serrulatus_Cathepsin-L_U6JPA9
0.1
Min
Max
0
178
FPKM

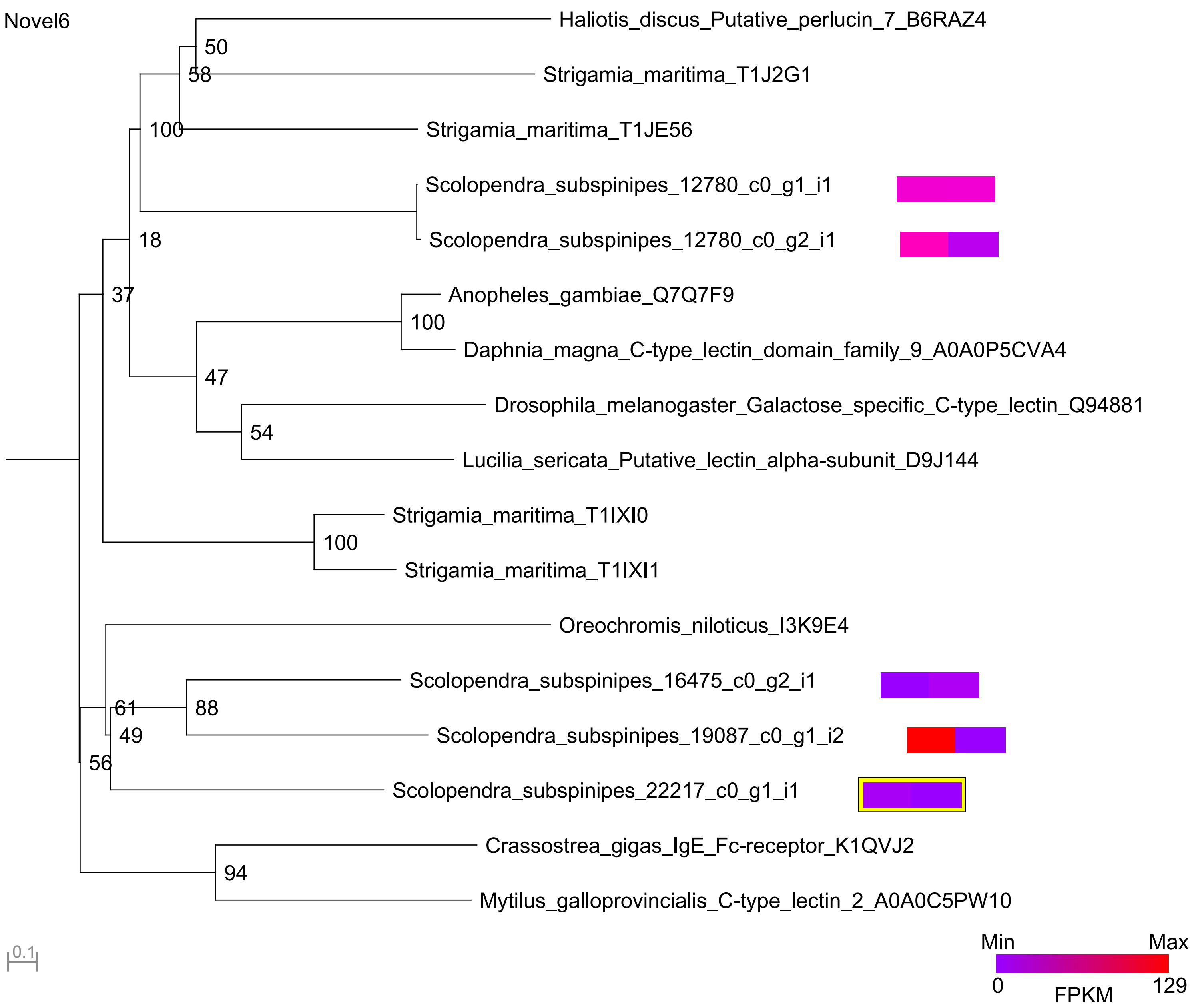

Novel6
Haliotis_discus_Putative_perlucin_7_B6RAZ4
50
Strigamia_maritima_T1J2G1
58
Strigamia_maritima_T1JE56
100
Scolopendra_subspinipes_12780_c0_g1_i1
Scolopendra_subspinipes_12780_c0_g2_i1
18
Anopheles_gambiae_Q7Q7F9
37
100
Daphnia_magna_C-type_lectin_domain_family_9_A0A0P5CVA4
47
Drosophila_melanogaster_Galactose_specific_C-type_lectin_Q94881
54
Lucilia_sericata_Putative_lectin_alpha-subunit_D9J144
Strigamia_maritima_T1IXI0
100
Strigamia_maritima_T1IXI1
Oreochromis_niloticus_I3K9E4
Scolopendra_subspinipes_16475_c0_g2_i1
61
88
Scolopendra_subspinipes_19087_c0_g1_i2
49
56
Scolopendra_subspinipes_22217_c0_g1_i1
Crassostrea_gigas_IgE_Fc-receptor_K1QVJ2
94
Mytilus_galloprovincialis_C-type_lectin_2_A0A0C5PW10
0.1
Min
Max
0
FPKM
129
