# Supplementary material for: True Lies: Using Proteomics to Assess the Accuracy of Transcriptome-Based Venomics in Centipedes Uncovers False Positives and Reveals Startling Intraspecific Variation in Scolopendra subspinipes
Source: Toxins (Basel). 2018 Feb 28;10(3):96. doi: 10.3390/toxins10030096 (PMC5869384; doi:10.3390/toxins10030096)
Supplement: Supplementary file 1 [file toxins-10-00096-s001.zip › Supplementary Tables.pdf]

TABLE S1: Putative and identified toxins, expression values, proteomic detection and completeness of ORF

Family	Contig	VG FPKM	Muscle FPKM	Proteome	Species BLAST	Lineage BLAST	ToxProt BLAST	Complete ORF
Alpha-latrotoxin-Lh1a (Alpha-LTX-Lh1a) (Alpha-latrotoxin) (Fragment)	TR21806|c0_g1_i1	14.253	7.253	0	0	0	1	N
BPFTx	TR1482|c0_g1_i1	636.523	0.001	0	0	1	0	N
BPFTx	TR14903|c0_g1_i1	526.626	0.177	1	1	1	0	Y
BPFTx	TR15132|c0_g1_i1	160.53	0.001	0	1	1	0	N
BPFTx	TR15180|c0_g1_i1	798.512	0.177	1	1	1	0	Y
BPFTx	TR16730|c0_g1_i1	928.508	2.311	0	0	1	0	Y
BPFTx	TR17938|c0_g1_i1	1077.94	1.535	0	1	1	0	N
BPFTx	TR18366|c0_g1_i1	1172.68	3.415	1	1	1	0	Y
BPFTx	TR20170|c1_g1_i1	4098.79	7.953	1	1	1	0	Y
BPFTx	TR20255|c0_g1_i1	381.867	5.692	1	1	1	0	Y
BPFTx	TR20502|c0_g2_i1	683.35	7.868	1	1	1	0	N
BPFTx	TR21242|c0_g1_i1	1015.22	0.894	0	1	1	0	Y
BPFTx	TR21868|c2_g1_i1	1499.22	4.579	1	1	1	0	Y
BPFTx	TR22070|c0_g1_i1	1108.45	3.289	1	1	1	0	Y
BPFTx	TR22084|c0_g1_i1	133.198	1.931	1	1	1	0	Y
BPFTx	TR22530|c0_g2_i2	2.881	0.008	0	0	1	0	Y
BPFTx	TR23186|c0_g3_i1	284.74	1.729	0	1	1	0	N
BPFTx	TR23186|c0_g3_i2	392.396	0.582	0	1	1	0	N
BPFTx	TR23314|c3_g2_i1	744.381	3.281	1	1	1	0	Y
BPFTx	TR23314|c3_g2_i2	791.682	1.383	1	1	1	0	Y
BPFTx	TR23314|c3_g3_i1	1093.73	2.294	1	1	1	0	Y
BPFTx	TR23538|c1_g1_i1	164.052	4.158	1	1	1	0	N
BPFTx	TR23538|c1_g5_i1	433.329	8.324	1	1	1	0	N
BPFTx	TR23538|c1_g6_i1	1545.09	1.063	1	1	1	0	N
BPFTx	TR23549|c0_g3_i1	352.127	0.001	1	1	1	0	N
BPFTx	TR23549|c0_g3_i2	659.859	0.843	1	1	1	0	N
BPFTx	TR23549|c0_g3_i3	928.864	2.8	1	1	1	0	Y
BPFTx	TR24015|c0_g1_i1	272.787	0.001	0	1	1	0	Y
BPFTx	TR24015|c0_g1_i2	77.182	0.001	0	1	1	0	Y
BPFTx	TR24015|c0_g1_i3	173.064	5.515	1	1	1	0	N
BPFTx	TR24015|c0_g1_i4	111.463	0.001	1	1	1	0	Y
BPFTx	TR24015|c0_g1_i5	2.668	0.001	1	1	1	0	N
BPFTx	TR24015|c0_g1_i6	231.76	2.37	0	1	1	0	Y
BPFTx	TR24015|c0_g1_i7	0.001	0.001	1	1	1	0	N
BPFTx	TR24015|c0_g1_i8	484.59	3.618	1	1	1	0	Y
BPFTx	TR24015|c0_g1_i9	402.226	5.726	1	1	1	0	Y
BPFTx	TR24172|c0_g1_i1	969.275	5.507	1	1	1	0	Y
BPFTx	TR24172|c0_g1_i2	139.886	0.422	1	1	1	0	Y
BPFTx	TR24172|c0_g1_i3	49.21	0.001	1	1	1	0	N
BPFTx	TR24172|c0_g1_i4	452.479	5.195	1	1	1	0	N

BPFTx	TR24172|c0_g1_i5	1.34	0.001	1	1	1	0	N
BPFTx	TR24172|c0_g1_i6	451.329	6.131	1	1	1	0	N
BPFTx	TR24172|c0_g1_i7	375.867	0.001	1	1	1	0	N
BPFTx	TR24403|c1_g1_i1	125.016	10.525	0	0	1	0	N
BPFTx	TR24403|c1_g2_i2	3427.34	14.058	1	1	1	0	N
BPFTx	TR24403|c1_g6_i1	635.919	21.05	0	0	1	0	N
BPFTx	TR24598|c0_g10_i1	657.298	0.001	1	1	1	0	N
BPFTx	TR24598|c0_g19_i1	323.574	0.001	0	0	1	0	N
BPFTx	TR24598|c0_g20_i1	46.008	0.001	0	0	1	0	N
BPFTx	TR24598|c0_g7_i1	595.721	16.833	1	1	0	0	N
BPFTx	TR994|c0_g1_i1	0.001	0	0	1	0	0	N
C-type Lectin	TR11675|c0_g1_i1	0.001	3.736	0	0	0	1	Y
C-type Lectin	TR11675|c0_g3_i1	1.079	1.864	0	0	0	1	Y
C-type Lectin	TR12780|c0_g2_i1	28.613	6.89	0	1	0	1	Y
C-type Lectin	TR14907|c0_g2_i1	1.352	3.871	0	1	0	0	Y
C-type Lectin	TR16475|c0_g2_i1	0.001	4.267	0	1	0	1	N
C-type Lectin	TR18573|c0_g1_i1	39.676	3.93	0	1	0	1	Y
C-type Lectin	TR19087|c0_g1_i2	129.392	0.261	0	1	0	1	Y
C-type Lectin	TR21453|c0_g1_i1	13.447	5.448	0	0	0	1	Y
C-type Lectin	TR22217|c0_g1_i1	3.154	0.001	1	1	0	1	Y
C-type Lectin	TR22217|c0_g1_i2	3.154	0.001	1	1	0	0	Y
C-type Lectin	TR3201|c0_g2_i1	1.447	0.001	0	0	0	1	Y
C-type Lectin	TR9820|c0_g1_i1	21.134	0	0	0	1	1	Y
C-type lectin galactose-binding isoform (CTL) (Venom C-type lectin galactose binding isoform)	TR13111|c0_g1_i1	1.802	1.628	0	0	0	1	N
C-type lectin galactose-binding isoform (CTL) (Venom C-type lectin galactose binding isoform)	TR7841|c0_g1_i1	5.229	1.611	0	0	0	1	Y
C-type lectin lectoxin-Enh6 (CTL)	TR17884|c0_g2_i1	7.708	6.165	0	0	0	1	Y
C-type lectin lectoxin-Lio1 (CTL)	TR3201|c0_g1_i1	0.001	1.855	0	0	0	1	N
C-type lectin lectoxin-Phi1 (CTL)	TR11675|c0_g2_i1	0.001	9.353	0	0	0	1	Y
C-type lectin mannose-binding isoform (CTL) (Venom C-type lectin mannose-binding isoform 1)	TR21502|c0_g1_i1	10.055	5.465	0	0	0	1	Y
C-type lectin TsL (Galactose-binding lectin) (CTL)	TR23607|c2_g1_i2	2.289	1.096	0	0	0	1	N
CAP	TR11262|c0_g1_i1	0.001	0.001	1	1	1	1	N
CAP	TR12732|c0_g3_i1	4.945	0.001	0	1	1	1	N
CAP	TR13866|c0_g1_i1	4.126	2.353	0	1	1	1	Y
CAP	TR18570|c0_g2_i1	59.016	0.001	1	1	1	1	Y
CAP	TR19492|c0_g1_i1	175.518	0.945	0	1	1	1	Y
CAP	TR22150|c0_g1_i3	10.613	3.272	0	1	1	1	Y
CAP	TR23597|c1_g4_i2	89.253	0.001	1	1	1	1	N

CAP	TR23597|c1_g5_i1	2683.6	7.379	1	1	1	1	N
CAP	TR23597|c1_g8_i1	4126.56	6.688	1	1	1	1	N
CAP	TR23597|c1_g8_i3	2345.15	0.001	1	1	1	1	N
CAP	TR23597|c1_g9_i1	2466.08	0.001	1	1	1	1	N
CAP	TR24670|c2_g10_i1	4857.67	5.499	1	1	1	1	N
CAP	TR24670|c2_g2_i1	3.51	0.001	1	1	1	1	N
CAP	TR24670|c2_g20_i1	8865.57	12.945	1	1	1	1	N
CAP	TR24670|c2_g21_i1	859.046	13.19	1	1	1	1	N
CAP	TR24670|c2_g24_i1	68.324	2.032	1	1	1	1	Y
CAP1	TR11262|c0_g3_i1	67.53	16.411	1	1	1	1	N
CAP1	TR11262|c0_g4_i1	11.656	2.842	1	1	1	1	N
CAP1	TR12732|c0_g1_i1	3.296	0.759	0	1	1	1	N
CAP1	TR18551|c0_g1_i1	362.218	6.932	1	1	1	1	Y
CAP1	TR23597|c1_g9_i2	0.001	0.001	1	1	1	1	N
CAP2	TR11262|c0_g2_i1	51.166	21.539	1	1	1	1	N
CAP2	TR17674|c0_g1_i1	3008.82	1.24	1	1	1	1	Y
CAP2	TR22150|c0_g1_i1	1.15	19.692	0	1	1	1	Y
CAP2	TR22150|c0_g1_i2	10.115	3.146	0	1	1	1	Y
CAP2	TR23597|c1_g1_i1	1809.41	19.017	1	1	1	1	N
CAP2	TR23597|c1_g4_i1	2114.93	8.863	1	1	1	1	N
CAP2	TR23597|c1_g7_i1	6512.92	22.837	1	1	1	1	N
CAP2	TR24670|c2_g11_i1	2122.06	0.001	1	1	1	1	N
CAP2	TR24670|c2_g17_i1	1771.02	5.085	1	1	1	1	N
CAP2	TR24670|c2_g23_i1	615.689	2.741	1	1	1	1	Y
CAP2	TR26097|c0_g1_i1	6.723	3.449	0	1	1	1	N
CAP3	TR23597|c1_g9_i3	4687.55	7.388	1	1	1	1	N
Cathepsin	TR20007|c0_g1_i1	2.3	3.55	0	1	0	0	Y
Cathepsin	TR20258|c0_g1_i1	10.411	5.473	0	1	0	0	Y
Cathepsin	TR22181|c0_g1_i1	181.649	45.498	1	1	0	0	Y
Cathepsin	TR22508|c0_g1_i1	106.589	48.871	0	1	0	1	Y
Cathepsin	TR22971|c0_g1_i1	263.372	32.03	0	1	0	0	Y
Cathepsin	TR24330|c0_g2_i1	84.475	16.15	0	1	0	0	Y
Chitinase	TR23222|c0_g1_i1	4.494	3.255	0	0	1	0	Y
Chitinase	TR23781|c0_g1_i1	2.443	0.784	0	0	1	0	N
Chitinase	TR23781|c0_g1_i2	16.53	4.444	0	0	1	0	Y
Coagulation factor V [Cleaved into: Coagulation factor V heavy chain; Coagulation factor V light chain]	TR11000|c0_g1_i1	5.739	2.007	0	0	0	1	Y
Coagulation factor X isoform 2 (PFX2) (EC 3.4.21.6) [Cleaved into: Factor X light chain; Factor X heavy chain; Activated factor Xa heavy chain]	TR23333|c0_g1_i2	2.336	0.001	0	0	0	1	Y
COEsteraseB	TR13080|c0_g1_i1	0.001	3.854	0	0	0	1	N
COEsteraseB	TR16515|c0_g1_i1	0.605	1.872	0	0	1	1	N

COEsteraseB	TR16515|c0_g2_i1	0.001	3.103	0	0	0	1	N
COEsteraseB	TR20006|c0_g1_i1	9.379	2.884	0	1	1	1	N
COEsteraseB	TR20006|c0_g1_i2	1.447	0.001	0	1	1	1	N
COEsteraseB	TR20112|c0_g1_i1	856.579	5.262	1	1	1	1	Y
COEsteraseB	TR21794|c0_g2_i1	0.001	2.952	0	1	1	1	Y
COEsteraseB	TR21794|c0_g2_i2	2.941	1.375	0	1	1	1	Y
COEsteraseB	TR22772|c0_g1_i1	28.138	10.66	0	1	1	1	Y
COEsteraseB	TR23019|c0_g1_i1	6.32	1.678	0	1	1	1	Y
COEsteraseB	TR23019|c0_g1_i2	0.296	0.001	0	1	1	1	Y
COEsteraseB	TR23312|c0_g1_i1	12.036	3.567	0	1	1	1	Y
COEsteraseB	TR24371|c0_g1_i4	2.336	1.105	0	0	1	1	N
COEsteraseB	TR24593|c0_g1_i1	10.21	13.392	0	1	1	1	Y
COEsteraseB	TR33564|c0_g1_i1	5.3	0.776	0	0	1	1	N
COEsteraseB	TR3702|c0_g1_i1	0.001	0.978	0	1	1	1	N
COEsteraseB	TR3702|c0_g2_i1	1.459	1.847	0	0	1	1	N
Colipase	TR21743|c0_g1_i2	25.352	11.672	0	0	1	0	Y
Cystine-knot cytokine domain TGF-beta-like peptide	TR697|c0_g1_i1	1270.4	2.361	1	1	0	0	Y
DUF1397	TR21935|c0_g1_i2	8.229	2.361	0	0	1	0	N
DUF1397	TR22011|c0_g1_i1	57.403	5.937	0	0	1	0	Y
DUF1397	TR22236|c0_g1_i1	79.471	30.166	0	0	1	0	Y
DUF1397	TR23948|c0_g3_i3	38.324	14.539	0	0	1	0	Y
DUF1397	TR23948|c0_g3_i4	32.656	18.865	0	0	1	0	Y
DUF3472	TR23452|c0_g1_i1	11.787	0.001	0	1	1	0	N
DUF3472	TR7722|c0_g1_i1	489.926	448.45	0	0	0	1	Y
Galactose-specific lectin nattectin (CTL)	TR12780|c0_g1_i1	17.988	17.921	0	1	0	1	Y
Galactose-specific lectin nattectin (CTL)	TR16817|c0_g2_i1	47.04	19.734	0	0	0	1	Y
Galactose-specific lectin nattectin (CTL)	TR19878|c0_g1_i1	0.001	33.303	0	0	0	1	Y
GDH	TR14437|c0_g1_i2	0.001	1.653	0	0	1	0	N
GDH	TR18680|c0_g1_i3	0.001	3.694	0	1	1	0	Y
GDH	TR20499|c1_g1_i2	0.001	0.001	0	1	1	0	N
GDH	TR22292|c0_g1_i1	2.182	0.557	0	0	1	0	Y
GDH	TR22292|c0_g1_i3	36.878	1.366	0	1	1	0	Y
GDH	TR22292|c0_g1_i4	17.051	1.138	0	1	1	0	Y
GDH	TR22427|c0_g1_i1	2.727	5.811	0	1	1	0	Y
GDH	TR23793|c0_g1_i1	45.214	0.379	0	1	1	0	Y
GDH	TR24241|c0_g1_i1	494.076	5.701	1	1	1	0	N
GDH	TR24353|c1_g2_i1	1.043	4.234	0	1	1	0	N
GDH	TR24353|c1_g2_i2	29.289	0.001	1	1	1	0	N
GDH	TR24353|c1_g2_i3	1.447	0.001	1	1	1	0	N
GDH	TR24769|c0_g2_i1	13.719	15.568	0	1	1	0	Y
GDH	TR24769|c0_g3_i1	10.518	3.525	0	1	1	0	N
GDH	TR27796|c0_g1_i1	0.001	10.306	0	0	1	0	N

GDH	TR40183|c0_g1_i1	3.7	0.632	0	0	1	0	N
GDH	TR670|c0_g1_i1	2.419	0.001	0	0	1	0	N
GDH	TR670|c0_g2_i1	1.209	0.624	0	0	1	0	N
GGT	TR24861|c1_g28_i2	1627.83	3.205	1	1	1	0	N
GGT	TR24861|c1_g28_i3	6303.53	6.544	1	1	1	0	N
Hyal	TR15320|c0_g1_i1	166.127	0.801	1	1	1	1	Y
Icarapin-like (Venom carbohydrate-rich protein)	TR18812|c0_g1_i1	532.827	52.194	1	1	0	1	Y
Insulin-like growth factor-binding protein-related protein 1 (IGFBP-rP1)	TR20904|c0_g1_i1	85.779	104.514	0	0	0	1	Y
Insulin-like growth factor-binding protein-related protein 1 (IGFBP-rP1)	TR20904|c0_g1_i2	120.854	82.992	0	0	0	1	Y
Kunitz-type serine protease inhibitor 1 (Kunitz protease inhibitor 1) (Kunitz protease inhibitor I)	TR22638|c0_g2_i2	45.842	28.623	0	0	0	1	Y
Kunitz-type serine protease inhibitor conotoxin Cal9.1d (Fragment)	TR17719|c0_g1_i1	5.68	9.622	0	0	0	1	Y
Kunitz-type serine protease inhibitor kunitoxin-Phi1	TR17719|c0_g1_i2	4.174	0.001	0	0	0	1	Y
LDLA	TR13632|c0_g1_i1	39.723	22.77	0	0	0	1	Y
LDLA	TR16285|c0_g1_i1	467.041	1.923	1	1	1	0	Y
LDLA	TR16784|c0_g1_i1	1051.12	5.861	1	1	1	0	Y
LDLA	TR18693|c0_g1_i1	2124.66	3.264	1	1	1	0	Y
LDLA	TR18693|c0_g2_i1	0.001	0.001	0	1	1	0	N
LDLA	TR18707|c0_g1_i1	2740.3	3.905	1	1	1	0	Y
LDLA	TR18753|c0_g2_i1	747.891	1.56	1	1	1	0	Y
LDLA	TR19024|c0_g2_i1	328.827	0.001	1	1	1	0	Y
LDLA	TR19024|c0_g2_i2	335.823	1.594	1	1	0	0	Y
LDLA	TR19364|c1_g1_i1	416.467	2.125	1	1	1	0	Y
LDLA	TR19970|c0_g1_i2	2345.46	1.518	1	1	1	0	N
LDLA	TR19970|c0_g9_i1	1366.57	1.518	1	1	0	0	N
LDLA	TR20240|c0_g1_i1	608.919	3.348	1	1	1	0	Y
LDLA	TR20240|c0_g1_i2	199.767	0.835	1	1	1	0	Y
LDLA	TR21056|c0_g1_i1	37.957	21.96	0	0	1	0	Y
LDLA	TR23314|c2_g1_i1	1968.33	44.595	1	1	1	0	N
LDLA	TR23314|c2_g2_i1	65.739	0.001	1	1	1	0	N
LDLA	TR23314|c2_g3_i1	488.906	0.953	1	1	1	0	N
LDLA	TR23314|c2_g4_i1	224.182	3.036	1	1	1	0	N
LDLA	TR23314|c2_g5_i1	369.226	0.928	1	1	1	0	N
LDLA	TR23314|c2_g6_i1	823.401	0.001	1	1	1	0	N
LDLA	TR23314|c2_g7_i1	0.001	0.001	1	1	1	0	N
LDLA	TR23314|c2_g8_i1	143.016	0.001	1	1	0	0	N
LDLA	TR23419|c0_g16_i1	840.299	3.441	1	1	1	0	N
LDLA	TR23419|c0_g16_i2	885.121	11.537	1	1	1	0	N
LDLA	TR23419|c0_g5_i1	306.357	14.716	1	1	1	0	N

LDLA	TR23419|c0_g6_i1	526.543	7.742	1	1	1	0	Y
LDLA	TR23419|c0_g7_i1	85.471	3.947	1	1	1	0	N
LDLA	TR23529|c0_g1_i1	210.854	1.957	1	1	1	0	N
LDLA	TR23529|c0_g14_i1	63.249	1.459	1	1	1	0	N
LDLA	TR23529|c0_g17_i1	7.186	0.001	1	1	1	0	N
LDLA	TR23529|c0_g4_i1	5055.51	6.131	0	1	1	0	N
LDLA	TR24610|c0_g2_i2	21.285	7.936	0	0	1	0	N
LDLA	TR24746|c0_g1_i1	2.763	2.193	0	0	0	1	Y
LDLA	TR28335|c0_g1_i1	324.036	0.001	0	1	1	0	N
LysoC	TR17964|c0_g1_i2	7.091	10.306	0	0	1	0	Y
LysoC	TR20567|c0_g1_i1	26.111	7.548	0	0	1	0	Y
LysoC	TR21900|c0_g1_i1	15	3.854	0	0	1	0	Y
M12A	TR19297|c0_g1_i1	23.241	6.688	0	0	0	1	Y
M12A	TR19522|c0_g3_i1	0.605	0.464	0	0	1	1	N
M12A	TR19522|c0_g3_i2	0.605	0.464	0	0	1	1	N
M12A	TR23788|c2_g2_i2	13.885	3.736	0	0	1	0	Y
Neprilysin-1 (EC 3.4.24.-)	TR18353|c0_g1_i1	7.648	1.628	0	0	0	1	N
Neprilysin-1 (EC 3.4.24.-)	TR23546|c0_g2_i2	3.925	0.001	0	0	0	1	N
Peptidylglycine alpha-hydroxylating monooxygenase	TR21936|c0_g1_i1	151.317	8.754	1	1	0	0	Y
Peptidylglycine alpha-hydroxylating monooxygenase	TR24300|c0_g1_i1	13.731	7.742	0	1	0	0	Y
Peroxiredoxin-4 (EC 1.11.1.15) (Fragments)	TR16950|c0_g1_i1	122.36	28.538	0	0	0	1	Y
Phosphatidylethanolamine-binding protein	TR16002|c0_g1_i1	35.099	0.001	1	1	0	0	Y
Phosphatidylethanolamine-binding protein	TR16002|c0_g1_i2	72.32	20.105	1	1	0	0	Y
Phospholipase A1 (EC 3.1.1.32) (allergen Pol a 1)	TR19711|c0_g1_i2	0.937	0.001	0	0	0	1	N
Phospholipase A1 (EC 3.1.1.32) (allergen Pol a 1)	TR19711|c0_g1_i3	4.447	2.488	0	0	0	1	N
Phospholipase A1 2 (EC 3.1.1.32) (allergen Pol d 1.02) (Fragment)	TR19114|c0_g1_i1	0.462	3.365	0	0	0	1	N
Phospholipase B (PLB) (EC 3.1.1.-)	TR24850|c1_g8_i1	24.154	11.385	0	0	0	1	Y
PLA2	TR14704|c0_g1_i1	6.652	2.159	0	0	0	1	Y
PLA2	TR19781|c0_g3_i1	75.937	2.285	0	1	1	1	N
PLA2	TR19781|c0_g3_i2	13.364	2.15	0	0	1	1	N
PLA2	TR19940|c0_g1_i1	152.55	5.912	0	1	1	1	Y
PLA2	TR20511|c0_g1_i1	20.763	10.989	0	1	1	1	N
PLA2	TR20889|c1_g1_i1	593.859	0.001	1	1	1	1	Y
PLA2	TR20889|c1_g1_i2	375.155	0.001	1	1	1	1	N
PLA2	TR20889|c1_g2_i1	945.785	2.024	1	1	1	1	Y
PLA2	TR20889|c1_g2_i2	337.471	0.001	1	1	0	0	Y
PLA2	TR36549|c0_g1_i1	5.099	0.001	0	1	1	1	N

Plancitoxin-1 (EC 3.1.22.1) (Plancitoxin I) (Plan-I) [Cleaved into: Plancitoxin-1 subunit alpha; Plancitoxin-1 subunit beta]	TR16978|c0_g1_i1	7.376	2.783	0	0	0	1	Y
pM12A	TR15609|c0_g1_i1	806.409	0.506	1	1	1	0	Y
pM12A	TR17723|c0_g1_i1	584.836	1.425	1	1	1	0	Y
pM12A	TR18344|c0_g1_i1	2.976	10.322	0	0	0	1	Y
pM12A	TR19522|c0_g4_i1	0.996	0.287	0	1	1	1	Y
pM12A	TR19522|c0_g4_i2	0.498	1.999	0	1	1	1	Y
pM12A	TR20169|c0_g1_i1	564.535	9.268	1	1	1	0	Y
pM12A	TR21073|c0_g1_i1	1.945	2.412	0	0	0	1	N
pM12A	TR21073|c0_g1_i2	0.001	4.537	0	0	0	1	N
pM12A	TR21550|c0_g2_i1	507.44	1.847	1	1	1	0	N
pM12A	TR22071|c0_g1_i1	976.414	1.257	1	1	1	0	Y
pM12A	TR22258|c0_g1_i1	1508.47	3.449	1	1	1	0	Y
pM12A	TR2263|c0_g1_i1	169.317	0.001	0	1	1	0	N
pM12A	TR22816|c0_g1_i1	288.38	7.742	1	1	1	0	Y
pM12A	TR23204|c0_g1_i1	10.435	9.631	0	0	1	0	Y
pM12A	TR23612|c0_g1_i1	270.819	2.167	1	1	1	0	Y
pM12A	TR23612|c0_g1_i2	242.965	2.994	1	1	1	0	Y
pM12A	TR23756|c1_g1_i1	570.772	1.206	1	1	1	0	Y
pM12A	TR23770|c4_g1_i1	6482.85	16.167	1	1	1	0	N
pM12A	TR24122|c1_g2_i2	666.832	3.432	1	1	1	0	N
pM12A	TR24122|c1_g2_i3	131.372	3.061	1	1	1	0	Y
pM12A	TR24122|c1_g2_i4	423.914	0.531	1	1	1	0	N
pM12A	TR24122|c1_g2_i5	50.052	1.467	1	1	1	0	N
pM12A	TR24398|c1_g1_i1	42.474	0.001	1	1	1	0	N
pM12A	TR24398|c1_g1_i2	174.759	0.001	1	1	1	0	Y
pM12A	TR24398|c1_g1_i3	133.471	0.001	1	1	1	0	Y
pM12A	TR24398|c1_g1_i4	220.767	0.001	1	1	1	0	Y
pM12A	TR24398|c1_g1_i5	25.198	0.43	1	1	1	0	Y
pM12A	TR24398|c1_g1_i6	77.016	0.228	1	1	1	0	N
pM12A	TR24398|c1_g1_i7	0.095	0.001	1	1	1	0	Y
pM12A	TR24398|c1_g1_i8	120.249	1.847	1	1	1	0	Y
pM12A	TR24398|c1_g1_i9	107.194	0.001	1	1	1	0	Y
Prokineticin Bm8-e	TR17286|c0_g1_i1	47.597	22.365	0	0	0	1	Y
Putative lysosomal acid lipase/cholesteryl ester hydrolase (Acid cholesteryl ester hydrolase) (LAL) (EC 3.1.1.13) (Cholesteryl esterase) (Lipase A) (Sterol esterase)	TR14815|c0_g2_i1	0.001	2.016	0	0	0	1	N

Putative lysosomal acid lipase/cholesteryl ester hydrolase (Acid cholesteryl ester hydrolase) (LAL) (EC 3.1.1.13) (Cholesteryl esterase) (Lipase A) (Sterol esterase)	TR22710|c0_g1_i1	0.486	20.619	0	0	0	1	N
Putative lysosomal acid lipase/cholesteryl ester hydrolase (Acid cholesteryl ester hydrolase) (LAL) (EC 3.1.1.13) (Cholesteryl esterase) (Lipase A) (Sterol esterase)	TR22710|c0_g1_i2	1.802	7.404	0	0	0	1	N
Putative lysosomal acid lipase/cholesteryl ester hydrolase (Acid cholesteryl ester hydrolase) (LAL) (EC 3.1.1.13) (Cholesteryl esterase) (Lipase A) (Sterol esterase)	TR22710|c0_g1_i3	0.001	4.9	0	0	0	1	N
Putative lysosomal acid lipase/cholesteryl ester hydrolase (Acid cholesteryl ester hydrolase) (LAL) (EC 3.1.1.13) (Cholesteryl esterase) (Lipase A) (Sterol esterase)	TR22710|c0_g1_i4	2.158	18.233	0	0	0	1	N
Putative lysosomal acid lipase/cholesteryl ester hydrolase (Acid cholesteryl ester hydrolase) (LAL) (EC 3.1.1.13) (Cholesteryl esterase) (Lipase A) (Sterol esterase)	TR23736|c0_g2_i1	20.111	3.373	0	0	0	1	Y
Putative lysosomal acid lipase/cholesteryl ester hydrolase (Acid cholesteryl ester hydrolase) (LAL) (EC 3.1.1.13) (Cholesteryl esterase) (Lipase A) (Sterol esterase)	TR6336|c0_g1_i1	0.001	3.702	0	0	0	1	Y
Reticulocalbin-2 (Taipoxin-associated calcium-binding protein 49 homolog)	TR22945|c0_g1_i1	50.81	27.712	0	0	0	1	Y
Reticulocalbin-2 (Taipoxin-associated calcium-binding protein 49 homolog)	TR22945|c0_g1_i2	59.087	10.618	0	0	0	1	Y
Ryncolin-1	TR22434|c0_g1_i1	65.597	23.984	0	0	0	1	N
Ryncolin-1	TR22879|c0_g2_i1	5.443	5.009	0	0	0	1	Y
Ryncolin-1	TR22879|c0_g2_i3	28.186	10.246	0	0	0	1	Y
Ryncolin-2	TR19263|c0_g1_i1	5.775	2.969	0	0	0	1	Y
Ryncolin-2	TR22894|c0_g1_i3	2.099	1.476	0	0	0	1	Y
Ryncolin-2	TR22894|c0_g2_i3	15.225	15.669	0	0	0	1	Y
Ryncolin-3	TR21469|c0_g1_i1	19.387	3.475	0	0	0	1	Y
Ryncolin-3	TR22894|c0_g1_i2	3.664	8.914	0	0	0	1	Y
Ryncolin-3	TR22894|c0_g2_i2	9.617	12.017	0	0	0	1	Y

Ryncolin-3	TR23904|c0_g1_i1	11.929	6.974	0	0	0	1	N
Ryncolin-3	TR23904|c0_g1_i2	18.64	5.541	0	0	0	1	N
Ryncolin-4	TR15485|c0_g1_i1	13.601	0.776	0	0	0	1	N
Ryncolin-4	TR18824|c0_g1_i1	11.158	5.532	0	0	0	1	Y
Ryncolin-4	TR21327|c0_g1_i2	17.206	3.078	0	0	0	1	Y
Ryncolin-4	TR22879|c0_g2_i2	0.806	58.51	0	0	0	1	Y
S1	TR16324|c0_g1_i1	4.293	2.977	0	0	0	1	Y
S1	TR17834|c0_g1_i1	2.893	8.163	0	1	1	1	Y
S1	TR18561|c0_g1_i1	12.439	4.562	0	1	1	1	Y
S1	TR19813|c0_g1_i1	41.016	0.219	0	1	1	1	Y
S1	TR19863|c0_g1_i1	7.174	5.22	0	1	1	1	N
S1	TR20081|c0_g1_i2	8.443	1.779	0	1	1	1	Y
S1	TR20089|c0_g1_i1	330.724	2.226	1	1	1	1	Y
S1	TR20969|c0_g1_i1	487.816	2.041	1	1	1	1	Y
S1	TR21969|c0_g1_i1	201.914	134.798	0	1	1	1	Y
S1	TR23063|c0_g1_i1	935.101	0.001	1	1	1	1	N
S1	TR23063|c0_g1_i2	171.712	1.729	1	1	1	1	N
S1	TR23063|c0_g1_i3	874.935	0.422	1	1	1	1	Y
S1	TR23063|c0_g1_i4	232.661	0.001	1	1	1	1	Y
S1	TR23063|c0_g1_i5	96.344	3.39	1	1	1	1	Y
S1	TR23364|c0_g1_i2	5.597	3.179	0	1	1	1	Y
S1	TR30852|c0_g1_i1	114.166	0.236	1	1	1	1	N
S1	TR4716|c0_g1_i1	2.822	1.037	0	1	1	1	Y
S1	TR5471|c0_g1_i1	15.368	0.001	0	1	1	1	Y
S10	TR21895|c0_g1_i1	144.629	31.844	1	1	0	1	Y
S10	TR22394|c0_g1_i2	26.063	7.101	0	1	0	1	Y
SCTX01	TR32344|c0_g1_i1	4.364	0.001	0	1	1	1	Y
SLPTX01	TR16565|c0_g1_i1	144.747	84.367	0	0	1	0	Y
SLPTX01	TR17671lc0_g1_i1	62.36	0	1	1	1	0	Y
SLPTX01	TR20858lc0_g1_i1	361.685	0	1	1	1	0	Y
SLPTX01	TR20858lc0_g1_i2	2494.36	5.212	1	1	1	0	Y
SLPTX01	TR21784|c0_g1_i1	2.182	0.363	0	1	1	0	Y
SLPTX01	TR25567|c0_g1_i1	3.51	2.707	0	1	1	0	Y
SLPTX01	TR27944|c0_g1_i1	6.64	9.563	0	1	1	0	Y
SLPTX01	TR28339|c0_g1_i1	2.241	1.729	0	1	1	0	Y
SLPTX01	TR8371|c0_g1_i1	5.502	7.421	0	1	1	0	Y
SLPTX01	TR8785|c0_g1_i1	0.001	3.66	0	1	1	0	N
SLPTX01	TR9209|c0_g1_i1	9.51	6.123	0	1	1	0	N
SLPTX01	TR9209|c0_g2_i1	4.767	36.17	0	1	1	0	N
SLPTX03	TR11442|c0_g1_i1	617.93	4.056	1	1	1	1	Y
SLPTX03	TR15561|c0_g1_i1	121.791	0.001	0	1	1	1	Y
SLPTX03	TR17861|c0_g1_i1	90517.8	793.828	1	1	1	1	Y
SLPTX03	TR17861|c0_g1_i2	26827.8	5.313	1	1	1	1	Y
SLPTX03	TR18290|c0_g1_i1	32.538	0.001	1	1	1	1	Y
SLPTX03	TR18290|c0_g1_i2	11.573	0.001	1	1	1	1	Y

SLPTX03	TR18979|c0_g1_i1	15134.3	17.575	1	1	1	1	Y
SLPTX03	TR7650|c0_g1_i1	751.14	6.308	1	1	1	0	Y
SLPTX04	TR12173|c0_g1_i1	8.81	0.001	0	0	1	1	N
SLPTX04	TR16094|c1_g1_i1	7202.88	2.825	1	1	1	0	Y
SLPTX04	TR16094|c1_g2_i1	1239.92	24.651	1	1	1	0	Y
SLPTX04	TR16094|c1_g2_i2	30411.8	40.758	1	1	1	0	Y
SLPTX04	TR16094|c2_g1_i1	4148.02	23.015	1	1	1	0	Y
SLPTX04	TR16094|c2_g2_i1	17838.5	8.762	1	1	0	0	Y
SLPTX04	TR16094|c2_g3_i1	150.878	3.162	1	1	1	0	Y
SLPTX04	TR16815|c0_g1_i3	1.636	0.675	0	0	1	0	N
SLPTX04	TR17816|c0_g1_i1	3.569	0.455	0	0	1	0	Y
SLPTX04	TR19682|c0_g1_i1	0.001	4.107	0	1	0	0	Y
SLPTX04	TR20546|c0_g1_i2	0.001	3.997	0	0	0	1	Y
SLPTX04	TR23333|c0_g1_i1	30.51	6.519	0	0	0	1	Y
SLPTX04	TR30593|c0_g1_i1	14.395	0.001	0	1	1	0	Y
SLPTX04	TR5896|c0_g1_i1	2.099	2.167	0	0	0	1	N
SLPTX04	TR7755|c0_g1_i1	1175.2	2.555	1	1	1	0	Y
SLPTX04	TR9372|c0_g1_i1	0.001	8.163	0	0	1	0	Y
SLPTX05	TR10171|c0_g1_i1	3.711	12.372	0	1	1	0	Y
SLPTX05	TR10863|c0_g1_i1	1.885	5.33	0	0	1	1	Y
SLPTX05	TR12168|c0_g1_i1	4.233	5.439	0	0	1	1	Y
SLPTX05	TR12173|c0_g2_i1	0.001	0.001	0	0	1	1	N
SLPTX05	TR14514|c0_g1_i1	21	16.959	0	0	1	1	Y
SLPTX05	TR15370|c0_g1_i1	16.091	7.894	0	1	1	1	Y
SLPTX05	TR16174|c0_g1_i1	76.613	103.376	0	0	1	1	Y
SLPTX05	TR16190|c0_g1_i1	1970.34	2.091	1	1	1	0	Y
SLPTX05	TR16190|c0_g2_i1	362.871	4.174	1	1	1	1	Y
SLPTX05	TR16815|c0_g1_i1	1.968	0.001	0	1	1	0	N
SLPTX05	TR16815|c0_g1_i2	23.087	1.299	0	0	1	0	N
SLPTX05	TR17410|c0_g1_i1	12.202	3.424	0	1	1	1	Y
SLPTX05	TR17420|c0_g1_i1	34.233	183.467	0	0	1	0	Y
SLPTX05	TR18313|c0_g1_i1	532.424	206.304	0	1	1	1	Y
SLPTX05	TR19433|c0_g1_i1	38.395	28.521	0	1	1	1	Y
SLPTX05	TR19800|c0_g1_i1	2.988	9.201	0	1	1	1	Y
SLPTX05	TR19800|c0_g1_i2	1.198	1.231	0	0	1	1	Y
SLPTX05	TR20505|c0_g1_i1	16.198	9.361	0	0	1	0	N
SLPTX05	TR20794|c0_g2_i3	12.036	28.387	0	0	1	0	Y
SLPTX05	TR23449|c0_g1_i1	42.012	53.012	0	0	1	1	Y
SLPTX05	TR23947|c0_g4_i2	35.087	21.859	0	0	1	1	N
SLPTX05	TR23947|c0_g4_i3	85.814	63.84	0	0	1	1	Y
SLPTX05	TR23947|c0_g4_i4	47.158	38.178	0	0	1	0	Y
SLPTX05	TR23947|c0_g4_i6	40.15	20.366	0	0	1	0	Y
SLPTX05	TR39901|c0_g1_i1	3.984	0.683	0	1	1	0	Y
SLPTX05	TR6208|c0_g2_i1	1.269	3.247	0	0	1	0	Y
SLPTX06	TR14501|c0_g1_i1	2619.59	2.091	0	0	1	0	Y

SLPTX07	TR10974|c0_g1_i1	9.996	0.001	0	0	0	1	N
SLPTX07	TR12345|c0_g1_i1	31.044	26.784	0	0	1	0	N
SLPTX07	TR14500|c0_g1_i1	37385.9	134.469	1	1	1	1	Y
SLPTX07	TR14500|c0_g2_i1	14505.8	33.506	1	1	1	1	Y
SLPTX07	TR14512|c0_g1_i1	10.957	3.466	0	0	0	1	Y
SLPTX07	TR15501|c0_g1_i1	3508.44	9.547	1	1	0	0	Y
SLPTX07	TR15501|c0_g2_i1	906.085	1.704	1	1	1	1	Y
SLPTX07	TR15501|c0_g3_i1	8235.53	47.749	1	1	1	1	Y
SLPTX07	TR17289|c1_g1_i1	107.597	82.031	0	1	1	1	Y
SLPTX07	TR17289|c1_g1_i2	204.487	222.673	0	0	1	0	Y
SLPTX07	TR18741|c0_g1_i1	13.15	25.359	0	0	1	0	Y
SLPTX07	TR18741|c0_g1_i2	5.692	4.394	0	0	1	0	Y
SLPTX07	TR18741|c0_g1_i3	2.028	2.083	0	0	1	0	Y
SLPTX07	TR18741|c0_g1_i4	12.498	12.211	0	0	1	0	Y
SLPTX07	TR19685|c0_g1_i1	0.001	42.976	0	0	1	0	N
SLPTX07	TR19685|c0_g2_i1	5.763	8.189	0	0	1	0	Y
SLPTX07	TR19685|c0_g2_i2	0.001	0.001	0	0	1	0	Y
SLPTX07	TR19685|c0_g2_i3	4.174	27.467	0	0	1	0	Y
SLPTX08	TR10676|c0_g1_i1	18.605	19.135	0	0	1	0	N
SLPTX08	TR12021|c0_g1_i1	5.597	3.356	0	0	1	1	Y
SLPTX08	TR12060|c0_g2_i1	250.767	0.001	0	0	1	0	N
SLPTX08	TR12060|c0_g2_i2	19.198	0.001	0	0	1	0	N
SLPTX08	TR12747|c0_g2_i1	30.012	0.001	0	0	1	0	N
SLPTX08	TR12747|c0_g3_i1	297.357	0.001	0	0	1	0	N
SLPTX08	TR21270|c0_g4_i2	1896.93	1.653	0	0	1	0	Y
SLPTX08	TR22949|c1_g2_i1	56.372	0.001	0	0	1	0	N
SLPTX08	TR22965|c1_g1_i1	5460.52	29.137	0	0	1	0	Y
SLPTX08	TR22965|c2_g1_i1	62.312	0.978	0	0	1	0	N
SLPTX09	TR16575|c1_g1_i1	0.001	0.001	1	1	1	1	Y
SLPTX09	TR18971|c0_g15_i1	2058.63	4.014	1	1	1	1	Y
SLPTX10	TR15721|c0_g1_i1	2.348	0.902	0	0	0	1	Y
SLPTX10	TR15721|c0_g2_i1	11.632	7.185	0	0	0	1	Y
SLPTX10	TR18770|c0_g1_i1	5204.9	28.134	1	1	1	0	Y
SLPTX10	TR18770|c0_g2_i1	1631.15	0.869	1	1	1	0	Y
SLPTX10	TR18770|c2_g10_i1	2857.76	58.03	1	1	1	0	N
SLPTX10	TR18770|c2_g11_i1	2982.35	17.938	1	1	1	0	Y
SLPTX10	TR18770|c2_g4_i1	1394.97	1.299	0	0	1	0	Y
SLPTX10	TR18770|c2_g7_i1	0.001	0.001	1	1	1	0	N
SLPTX10	TR21712|c0_g1_i1	16.233	2.412	0	0	0	1	Y
SLPTX10	TR21712|c0_g1_i2	1.802	0.001	0	0	0	1	N
SLPTX11	TR1079|c0_g1_i1	1152.56	20.425	0	0	1	0	N
SLPTX11	TR13891|c0_g1_i1	4043.99	29.348	1	1	1	1	Y
SLPTX11	TR15540|c0_g1_i1	6972.68	35.268	1	1	1	0	Y
SLPTX11	TR17307|c0_g1_i1	3.664	5.65	0	0	1	0	Y
SLPTX11	TR18191|c0_g1_i1	65.68	1.088	0	1	1	0	Y

SLPTX11	TR18613|c0_g1_i1	1699.32	15.112	1	1	1	0	Y
SLPTX11	TR18660|c1_g1_i1	1648.07	7.219	1	1	1	0	Y
SLPTX11	TR19632|c0_g1_i1	1891.63	11.689	1	1	1	0	N
SLPTX11	TR19670|c0_g1_i1	10487.5	36.036	1	1	1	0	Y
SLPTX11	TR19632|c1_g1_i1	4769.53	24.642	1	1	1	0	Y
SLPTX11	TR18660|c0_g1_i1	1189.5	4.436	1	1	1	0	N
SLPTX11	TR24034|c3_g1_i1	93.759	0.001	1	1	1	0	Y
SLPTX11	TR24034|c3_g1_i2	44.644	0.001	1	1	1	0	Y
SLPTX11	TR24034|c3_g1_i3	53.455	0.001	1	1	1	0	Y
SLPTX11	TR19632|c0_g2_i1	605.409	0.531	1	1	1	0	Y
SLPTX11	TR27662|c0_g1_i1	321.689	0.261	1	1	1	1	Y
SLPTX11	TR19632|c1_g1_i1	4769.53	24.642	1	1	1	0	Y
SLPTX12	TR16575|c0_g1_i1	7287.62	9.757	1	1	1	1	N
SLPTX12	TR16754|c0_g1_i1	1088.01	0.472	1	1	1	0	Y
SLPTX12	TR18971|c0_g11_i1	862.164	0.001	1	1	1	1	Y
SLPTX12	TR18971|c0_g13_i1	13.079	0.001	1	1	1	1	Y
SLPTX12	TR18971|c0_g14_i1	5113.98	10.795	1	1	1	1	Y
SLPTX12	TR18971|c0_g2_i1	208.222	6.823	1	1	1	1	Y
SLPTX12	TR18971|c0_g4_i1	3971.35	22.821	1	1	1	1	Y
SLPTX13	TR17575|c0_g1_i1	12168.6	27.366	1	1	1	1	Y
SLPTX13	TR17575|c1_g1_i1	4653.3	46.096	1	1	1	1	Y
SLPTX13	TR17575|c1_g2_i1	1424.17	1.855	1	1	0	0	Y
SLPTX14	TR11950|c0_g1_i1	1.292	5.305	0	1	1	0	N
SLPTX14	TR13552|c0_g1_i1	11.739	67.424	0	0	1	0	Y
SLPTX14	TR14498|c0_g1_i1	0.001	1.037	0	1	1	0	Y
SLPTX14	TR14498|c0_g2_i1	0.001	6.418	0	1	1	0	Y
SLPTX14	TR15808|c0_g1_i1	4.399	22.981	0	1	1	0	Y
SLPTX14	TR16226|c0_g2_i1	5.099	4.318	0	0	0	1	N
SLPTX14	TR17762|c0_g1_i1	201.285	323.645	0	0	1	0	Y
SLPTX14	TR18319|c0_g1_i1	6.794	47.867	0	0	1	0	Y
SLPTX14	TR18955|c0_g2_i1	6.794	5.423	0	0	1	0	Y
SLPTX14	TR19329|c0_g1_i1	11.087	10.365	0	1	1	0	Y
SLPTX14	TR20235|c0_g1_i1	71.68	9.285	0	0	1	0	Y
SLPTX14	TR22155|c0_g3_i1	1313.79	7.759	1	1	1	0	Y
SLPTX14	TR22155|c0_g5_i1	1428.6	59.708	1	1	1	0	Y
SLPTX14	TR22155|c0_g6_i1	140.87	0.001	1	1	1	0	Y
SLPTX14	TR6948|c0_g1_i1	0.001	24.971	0	1	1	0	N
SLPTX15	TR10447|c0_g1_i1	1.767	0.001	1	1	1	1	Y
SLPTX15	TR10447|c0_g2_i1	8537.8	1.931	1	1	0	0	Y
SLPTX15	TR11265|c0_g1_i1	5581.26	3.424	1	1	1	1	Y
SLPTX15	TR13776|c0_g1_i1	19.044	2.943	0	1	1	0	Y
SLPTX15	TR14522|c1_g1_i1	8748.58	0.86	1	1	1	0	Y
SLPTX15	TR16482|c0_g1_i1	8228.99	106.622	1	1	1	1	Y
SLPTX15	TR17133|c1_g1_i1	586.555	21.168	1	1	1	0	N
SLPTX15	TR17276|c0_g1_i1	1483.29	5.541	1	1	1	1	Y

SLPTX15	TR17276|c0_g2_i1	1847.06	3.407	1	1	1	1	Y
SLPTX15	TR17276|c0_g3_i1	2408.95	6.974	1	1	1	1	Y
SLPTX15	TR17462|c0_g1_i1	40.648	31.355	0	1	1	1	Y
SLPTX15	TR19470|c2_g3_i1	7486.56	0.001	1	1	1	0	Y
SLPTX15	TR19470|c2_g5_i1	3302.08	1.4	1	1	1	0	Y
SLPTX15	TR20957|c0_g1_i1	61048.5	28.58	1	1	0	0	Y
SLPTX15	TR20957|c0_g2_i1	44668.1	83.675	1	1	1	0	Y
SLPTX15	TR20957|c0_g3_i1	48709.4	21.497	1	1	1	0	Y
SLPTX15	TR30850|c0_g1_i1	1785.89	723.224	0	1	1	0	Y
SLPTX15	TR3944|c0_g1_i1	7617.32	8.509	1	1	1	1	Y
SLPTX15	TR4154|c0_g2_i1	1388.6	3.196	1	1	1	0	Y
SLPTX15	TR5924|c0_g1_i1	6.403	0.658	0	0	0	1	N
SLPTX15	TR7644|c0_g1_i1	29.692	20.341	0	1	1	0	Y
SLPTX16	TR10889|c0_g1_i2	6.012	10.879	0	1	1	1	Y
SLPTX16	TR11960|c0_g1_i1	36.771	100.255	0	1	1	1	Y
SLPTX16	TR11969|c0_g1_i1	6.735	8.804	0	1	1	1	Y
SLPTX16	TR11969|c0_g2_i1	0.001	6.848	0	1	1	1	Y
SLPTX16	TR11969|c0_g3_i1	0.001	6.848	0	1	1	1	Y
SLPTX16	TR13490|c0_g1_i2	4.02	4.065	0	0	0	1	Y
SLPTX16	TR14582|c0_g4_i1	2.87	0.001	0	1	1	1	Y
SLPTX16	TR15524|c0_g1_i1	4.482	19.979	0	0	1	1	Y
SLPTX16	TR16248|c0_g1_i1	63.534	6.249	0	0	0	1	Y
SLPTX16	TR16248|c0_g1_i2	25.482	0.001	0	0	0	1	Y
SLPTX16	TR16413|c0_g1_i1	880.105	0.001	0	1	1	0	N
SLPTX16	TR16413|c1_g1_i1	1201.14	0.001	1	1	1	1	Y
SLPTX16	TR17757|c0_g1_i1	728.243	347.014	0	1	1	0	Y
SLPTX16	TR18437|c0_g1_i1	142.364	67.247	0	0	0	1	Y
SLPTX16	TR18472|c0_g1_i1	12.949	64.928	0	0	1	0	N
SLPTX16	TR18472|c0_g2_i2	46.969	79.062	0	1	1	1	Y
SLPTX16	TR19255|c0_g3_i1	43.779	34.442	0	1	1	0	N
SLPTX16	TR19565|c0_g1_i1	168.404	338.218	0	1	1	1	Y
SLPTX16	TR20122|c0_g1_i1	24.463	17.01	0	1	1	0	Y
SLPTX16	TR20168|c0_g4_i1	69.664	111.767	0	1	1	1	Y
SLPTX16	TR20168|c0_g4_i2	92.348	248.504	0	1	1	1	N
SLPTX16	TR20786|c0_g1_i1	4.518	15.492	0	0	1	1	Y
SLPTX16	TR20786|c0_g1_i2	1.838	16.588	0	1	1	1	Y
SLPTX16	TR20786|c0_g1_i3	3.664	8.206	0	1	1	1	Y
SLPTX16	TR20786|c0_g2_i1	3.13	39.451	0	0	1	1	Y
SLPTX16	TR20786|c0_g3_i1	1.636	5.895	0	0	1	1	Y
SLPTX16	TR20786|c0_g4_i1	19.459	71.902	0	1	1	1	Y
SLPTX16	TR23350|c0_g1_i1	20.383	25.022	0	0	0	1	Y
SLPTX16	TR24750|c0_g1_i2	1.968	5.448	0	0	0	1	N
SLPTX16	TR33227|c0_g1_i1	5.253	0.388	0	0	1	0	N
SLPTX16	TR5548|c0_g1_i1	1.767	1.265	0	0	1	1	Y
SLPTX16	TR6911|c0_g1_i1	4.447	12.954	0	0	1	1	Y

SLPTX16	TR8305|c0_g1_i1	64.245	193.772	0	0	1	0	N
SLPTX16	TR8556|c0_g2_i1	5.395	1.417	0	1	1	1	Y
SLPTX16	TR8556|c0_g3_i1	13.482	43.558	0	1	1	1	Y
SLPTX17	TR11220|c0_g1_i1	7.032	2.066	0	0	1	0	Y
SLPTX17	TR11773|c0_g1_i1	420.464	12.279	1	1	1	0	Y
SLPTX17	TR16146|c0_g3_i1	69.534	11.604	0	0	1	0	N
SLPTX17	TR16752|c0_g1_i1	59.419	109.996	0	0	1	0	Y
SLPTX17	TR17036|c0_g1_i2	0.984	2.53	0	0	1	0	Y
SLPTX17	TR18448|c0_g1_i1	17.419	11.284	0	0	1	0	Y
SLPTX17	TR18448|c0_g3_i1	11.431	12.726	0	0	1	0	Y
SLPTX17	TR18755|c0_g1_i1	152.633	95.608	0	0	1	0	Y
SLPTX17	TR18755|c0_g1_i2	121.838	115.047	0	0	1	0	Y
SLPTX17	TR18837|c0_g3_i1	116.016	0.001	1	1	1	0	N
SLPTX17	TR18894|c0_g1_i1	3845.67	1802.106	0	0	1	0	Y
SLPTX17	TR22204|c0_g5_i1	29.846	51.418	0	0	1	0	Y
SLPTX17	TR22204|c0_g6_i1	62.858	64.616	0	0	1	0	Y
SLPTX17	TR22204|c0_g6_i2	28.565	2.353	0	0	1	0	Y
SLPTX17	TR5383|c0_g1_i1	1.423	2.699	0	1	1	0	Y
SLPTX17	TR608|c0_g1_i1	0.001	1.982	0	0	1	0	N
SLPTX18	TR17281|c0_g1_i1	9.617	4.394	0	0	1	0	Y
SLPTX18	TR17281|c0_g1_i2	5.336	5.49	0	0	1	0	Y
SLPTX18	TR21743|c0_g1_i1	53.348	24.305	0	0	1	0	Y
SLPTX19	TR21403|c0_g1_i1	19.802	10.483	0	0	1	0	Y
SLPTX27	TR13532|c0_g1_i1	23.348	159.187	0	0	1	0	N
SLPTX27	TR14827|c0_g1_i1	335.562	209.593	0	0	1	0	Y
SLPTX27	TR18510|c0_g1_i1	10.21	15.096	0	0	1	0	Y
SLPTX27	TR18713|c0_g1_i1	0.001	11.478	0	0	1	0	Y
SLPTX27	TR18716|c0_g1_i1	5113.06	12.296	0	0	1	0	Y
SLPTX27	TR21542|c0_g5_i1	41.965	230.153	0	0	1	0	Y
SLPTX27	TR21542|c0_g5_i2	26.763	215.21	0	0	1	0	Y
SLPTX27	TR21542|c0_g5_i3	7.257	12.009	0	0	1	0	Y
SLPTX27	TR2770|c0_g1_i1	0.001	12.802	0	0	1	0	Y
SLPTX27	TR2770|c0_g2_i1	2.253	0.001	0	0	1	0	N
SLPTX27	TR5036|c0_g1_i1	0.001	0.001	0	0	1	0	N
SLPTX28	TR15500|c0_g1_i2	5610.49	2.893	0	0	1	0	Y
SLPTX29	TR13658|c0_g1_i1	15.806	3.129	0	1	0	0	Y
SLPTX29	TR16516|c0_g1_i1	9.937	12.127	0	1	0	0	Y
SLPTX29	TR16849|c0_g1_i1	0.001	5.094	0	1	0	0	Y
SLPTX29	TR16849|c0_g2_i1	0.001	0.001	0	1	0	0	Y
SLPTX29	TR16849|c0_g3_i1	0.001	2.547	0	1	0	0	Y
SLPTX29	TR16849|c0_g4_i1	12.391	0.001	0	1	0	0	Y
SLPTX29	TR17378|c0_g1_i1	8.3	0.658	0	1	0	0	Y
SLPTX29	TR17378|c0_g1_i2	20.466	4.487	0	1	0	0	Y
SLPTX29	TR18566|c0_g1_i1	9.202	62.668	0	1	0	0	Y
SLPTX29	TR20398|c0_g1_i1	178.02	60.661	1	1	0	0	Y

SLPTX29	TR9140|c0_g1_i1	1.802	8.332	0	1	0	0	Y
SLPTX30	TR11088|c0_g1_i1	1.233	6.317	0	1	0	0	Y
SLPTX30	TR14992|c0_g1_i1	4.174	13.156	0	1	0	0	Y
SLPTX30	TR16900|c0_g2_i2	12.451	5.735	0	1	0	0	Y
SLPTX30	TR17377|c0_g1_i1	968.505	1.198	1	1	0	0	N
SLPTX30	TR17377|c0_g4_i1	5685.34	39.89	1	1	0	0	N
SLPTX30	TR17377|c0_g6_i1	0.001	0.001	0	1	0	0	N
SLPTX30	TR17377|c0_g9_i1	1837.71	20.628	0	1	0	0	N
SLPTX30	TR17649|c0_g1_i1	15.296	0.523	0	1	0	0	N
SLPTX30	TR17937|c0_g1_i1	2.36	10.314	0	1	0	0	Y
SLPTX30	TR19075|c0_g1_i2	35760.7	31.296	1	1	0	0	Y
SLPTX30	TR20371|c0_g4_i2	50.739	19.995	0	1	0	0	Y
SLPTX30	TR23271|c1_g4_i3	40.802	49.394	0	1	0	0	Y
SLPTX30	TR23271|c1_g5_i1	170.692	65.232	0	1	0	0	Y
SLPTX30	TR23271|c1_g5_i2	56.206	27.358	0	1	0	0	Y
SLPTX30	TR8258|c0_g1_i1	2182.81	0.001	1	1	0	0	Y
SLPTX31	TR20670|c0_g1_i1	6757.79	2.142	1	1	0	0	Y
SLPTX31	TR20670|c1_g3_i1	1245.56	1.063	1	1	0	0	N
SLPTX31	TR20670|c1_g4_i2	45	0.001	0	1	0	0	Y
SLPTX31	TR20670|c1_g6_i1	6246.22	0.877	1	1	0	0	Y
SLPTX31	TR20670|c1_g6_i2	3382.91	2.716	1	1	0	0	Y
SLPTX31	TR20670|c1_g7_i1	75.486	0.001	0	1	0	0	N
Snaclec A10 (C-type lectin A10)	TR11739|c0_g1_i1	2.028	25.106	0	0	0	1	N
Snaclec A14 (C-type lectin A14)	TR28449|c0_g1_i1	3.617	0.001	0	0	0	1	N
Snaclec agkicetin-C subunit beta (Antithrombin A subunit B)	TR10075|c0_g1_i1	4.221	10.82	0	0	0	1	N
Snaclec B9 (C-type lectin B9)	TR24705|c0_g1_i1	16.044	5.735	0	0	0	1	Y
Snaclec B9 (C-type lectin B9)	TR24705|c0_g1_i2	0.001	0.001	0	0	0	1	Y
Snaclec coagulation factor IX/factor X-binding protein subunit B (IX/X-bp subunit B)	TR24705|c0_g1_i3	1.731	0.7	0	0	0	1	N
Transferrin	TR23345|c0_g1_i2	24.036	0.001	0	1	1	0	Y
Transferrin	TR23345|c0_g1_i3	24.972	0.001	0	1	1	0	Y
Transferrin	TR23627|c0_g2_i1	9.664	2.656	0	1	1	0	Y
Transferrin	TR24679|c0_g6_i1	255.807	10.483	1	1	1	0	N
Turripeptide Gsg9.2	TR19477|c0_g1_i1	2.609	0.001	0	0	0	1	Y
Turripeptide Gsg9.2	TR19477|c0_g1_i3	224.787	466.16	0	0	0	1	Y
Turripeptide Gsp9.3	TR10015|c0_g1_i1	217.151	20.813	0	0	0	1	Y
Turripeptide Gsp9.3	TR22969|c0_g1_i8	11.478	0.001	0	0	0	1	Y
Turripeptide Ici9.2	TR10015|c0_g2_i1	666.369	581.561	0	0	0	1	Y
Turripeptide Lol9.1 (Turripeptide OL11)	TR17065|c0_g1_i1	240.036	221.602	0	0	0	1	Y
Turripeptide OL11-like (Fragment)	TR19477|c0_g1_i2	68.858	265.481	0	0	0	1	Y
Turripeptide Pal9.2	TR22969|c0_g1_i3	0.001	4.368	0	0	0	1	Y

U-actitoxin-Avd3j (U-AITX-Avd3j) (AsKC7)	TR22638|c0_g2_i1	54.036	45.903	0	0	0	1	Y
U1-hexatoxin-Iw1a (U1-HXTX-Iw1a) (Atracotoxin-Hs20f7358) (AcTx-Hs20f7358)	TR36276|c0_g1_i1	0.001	6.704	0	0	0	1	N
U24-ctenitoxin-Pn1a (U24-CNTX-Pn1a) (Venom protein PN16C3)	TR19696|c0_g2_i1	18.605	2.909	0	0	0	1	Y
Unchar	TR21205|c0_g2_i1	1139.93	3.348	1	1	1	0	Y
Unchar	TR23452|c0_g1_i3	7.127	0.001	0	0	1	0	N
Unchar02	TR15561|c0_g1_i7	271.542	0.001	1	1	0	0	Y
Unchar02	TR24670|c2_g15_i1	12.652	0.001	1	1	1	1	N
Unchar05	TR20861|c0_g1_i1	115.874	0.001	0	0	1	0	N
Unchar05	TR20861|c1_g1_i1	433.436	1.096	1	1	1	0	Y
Unchar06	TR16224|c0_g1_i1	3249.04	198.453	1	1	1	0	Y
Unchar08	TR2247|c0_g1_i1	5.134	1.316	0	0	1	0	N
Unchar10	TR15456|c0_g1_i1	6.166	0.001	0	0	1	0	N
Unchar10	TR24015|c0_g1_i10	292.21	1.088	1	1	1	0	N
Unchar10	TR24569|c0_g1_i1	15.273	3.879	1	1	1	0	N
Unchar10	TR24569|c0_g16_i1	136.21	17.263	1	1	1	0	N
Unchar10	TR24569|c0_g17_i1	60.961	7.759	1	1	1	0	N
Unchar10	TR24569|c0_g27_i1	138.759	6.654	1	1	1	0	N
Unchar10	TR24569|c0_g27_i2	101.099	2.682	1	1	1	0	N
Unchar10	TR24569|c0_g8_i1	0.001	2.876	1	1	1	0	N
Unchar10	TR24569|c0_g8_i2	44.822	0.001	1	1	1	0	N
Unchar10	TR24569|c0_g8_i3	0.001	0.001	1	1	1	0	N
Unchar10	TR25114|c0_g1_i1	0.001	0.902	0	1	1	1	N
Unchar11	TR12347|c0_g1_i1	89.526	496.385	0	1	1	0	Y
Unchar11	TR14080|c0_g1_i1	54.498	27.636	0	1	1	0	Y
Unchar11	TR19607|c0_g1_i1	54.546	325.973	0	1	1	0	Y
Unchar11	TR20772|c0_g2_i1	16357.5	197.938	1	1	1	0	Y
Unchar11	TR20772|c0_g2_i2	156.273	12.591	1	1	1	0	Y
Unchar11	TR20772|c0_g3_i1	5652.98	559.263	1	1	1	0	Y
Unchar11	TR21070|c0_g4_i1	13894.6	196.952	1	1	1	0	Y
Unchar11	TR21070|c0_g4_i3	6257.32	613.085	0	0	1	0	N
Unchar11	TR35624|c0_g1_i1	2.787	1.189	0	0	0	1	N
Uncharacterized12	TR14806|c0_g1_i1	1.684	4.318	0	1	0	0	N
Uncharacterized12	TR23551|c0_g1_i2	21.676	5.701	1	1	0	0	N
Uncharacterized13	TR20632|c0_g1_i1	79.862	0.32	1	1	0	0	Y
Uncharacterized13	TR9580|c0_g1_i1	80.633	27.931	0	1	0	0	Y
Uncharacterized14	TR21637|c0_g3_i1	9.332	1.906	1	1	0	0	Y
Uncharacterized15	TR17179|c0_g1_i1	3917.54	28.547	1	1	0	0	Y
Veficolin-1 (Fragment)	TR22894|c0_g2_i1	13.518	21.699	0	0	0	1	Y
Venom acid phosphatase Acph-1 (EC 3.1.3.2) (allergen Api m 3)	TR22183|c0_g1_i1	3.783	1.611	0	0	0	1	Y

Venom acid phosphatase Acph-1 (EC 3.1.3.2) (allergen Api m 3)	TR22183|c0_g1_i2	3.036	0.548	0	0	0	1	Y
Venom acid phosphatase Acph-1 (EC 3.1.3.2) (allergen Api m 3)	TR22183|c0_g1_i3	0.001	0.001	0	0	0	1	Y
Venom acid phosphatase Acph-1 (EC 3.1.3.2) (allergen Api m 3)	TR22183|c0_g1_i4	2.075	1.746	0	0	0	1	Y
Venom dipeptidyl peptidase 4 (Allergen C) (Venom dipeptidyl peptidase IV) (EC 3.4.14.5) (allergen Api m 5)	TR22897|c0_g1_i1	0.723	2.808	0	0	0	1	Y
Venom dipeptidyl peptidase 4 (Allergen C) (Venom dipeptidyl peptidase IV) (EC 3.4.14.5) (allergen Api m 5)	TR22897|c0_g1_i2	5.111	2.597	0	0	0	1	Y
Venom dipeptidyl peptidase 4 (EC 3.4.14.5) (Venom dipeptidyl peptidase IV) (allergen Ves v 3)	TR22897|c0_g1_i4	0.001	1.923	0	0	0	1	Y
Venom peptide SjAPI-2 (Ascaris-type protease inhibitor-2)	TR20998|c0_g2_i1	9.024	5.701	0	0	0	1	Y
Venom phosphodiesterase 1 (Ectonucleotide pyrophosphatase/phosphodiesterase family member 3) (PDE-3) [Includes: Alkaline phosphodiesterase I (PDE) (EC 3.1.4.1); Nucleotide pyrophosphatase (NPPase) (EC 3.6.1.9) (Nucleotide diphosphatase)]	TR20520|c0_g1_i2	6.225	0.001	0	0	0	1	N
Venom phosphodiesterase 1 (Ectonucleotide pyrophosphatase/phosphodiesterase family member 3) (PDE-3) [Includes: Alkaline phosphodiesterase I (PDE) (EC 3.1.4.1); Nucleotide pyrophosphatase (NPPase) (EC 3.6.1.9) (Nucleotide diphosphatase)]	TR21503|c0_g1_i3	0.001	0.001	0	0	0	1	Y
Venom phosphodiesterase 2 (Ectonucleotide pyrophosphatase/phosphodiesterase family member 3) (PDE-3) [Includes: Alkaline phosphodiesterase I (PDE) (EC 3.1.4.1); Nucleotide pyrophosphatase (NPPase) (EC 3.6.1.9) (Nucleotide diphosphatase)]	TR20030|c0_g1_i1	2.478	2.547	0	0	0	1	Y

Venom phosphodiesterase 2 (Ectonucleotide pyrophosphatase/phosphodiesterase family member 3) (PDE-3) [Includes: Alkaline phosphodiesterase I (PDE) (EC 3.1.4.1); Nucleotide pyrophosphatase (NPPase) (EC 3.6.1.9) (Nucleotide diphosphatase)]	TR20520|c0_g1_i1	11.312	1.046	0	0	0	1	N
Venom phosphodiesterase 2 (Ectonucleotide pyrophosphatase/phosphodiesterase family member 3) (PDE-3) [Includes: Alkaline phosphodiesterase I (PDE) (EC 3.1.4.1); Nucleotide pyrophosphatase (NPPase) (EC 3.6.1.9) (Nucleotide diphosphatase)]	TR20843|c0_g1_i1	11.217	6.915	0	0	0	1	N
Venom prothrombin activator notecarin-D1 (vPA) (EC 3.4.21.6) (Venom coagulation factor Xa-like protease) [Cleaved into: Notecarin-D1 light chain; Notecarin-D1 heavy chain]	TR24746|c0_g1_i2	4.779	1.999	0	0	0	1	N
Venom prothrombin activator oscutarin-C non-catalytic subunit (vPA) (Venom coagulation factor Va-like protein) [Cleaved into: Oscutarin-C non-catalytic subunit heavy chain; Oscutarin-C non-catalytic subunit light chain]	TR22552|c0_g1_i1	6.7	53.847	0	0	0	1	N
Zinc metalloproteinase-disintegrin-like EoVMP2 (EC 3.4.24.-) (Haemorrhagic 56 kDa metalloproteinase) (Snake venom metalloproteinase) (SVMP) (Zinc metalloproteinase-disintegrin-like Eoc22)	TR13064|c0_g1_i1	2.419	1.451	0	0	0	1	N

TABLE S2

Protein summary from Protein Pilot search of combined crude venom and all rpHPLC fraction datasetas well as additional contigs identified by concatenated individual Protein Pilot data searches (last 66 rows)

N	Unused	Total	%Cov	%Cov(50)	%Cov(95)	Accession	Peptides(95%)
1	99.32	99.32	85.71000099	83.19	83.19000006	TR15540|c0_g1_i1|CDS3	173
2	98.02	98.02	92.46000051	85.509998	85.50999761	TR23240|c0_g1_i4|CDS2	158
3	91.94	91.94	85.07000208	83.770001	80.4099977	TR24241|c0_g1_i1|CDS3	83
4	91.72	91.72	72.75000215	64.899999	61.6599977	TR21205|c0_g2_i1|CDS2	90
5	88.71	88.71	87.66999841	85.390002	85.39000154	TR19670|c0_g1_i1|CDS1	171
6	80.57	80.57	85.53000093	78.750002	76.74000263	TR20112|c0_g1_i1|CDS1	84
7	79.81	79.81	82.31999874	76.76	70.70000172	TR22258|c0_g1_i1|CDS1	79
8	72.59	72.59	78.40999961	75.43	75.42999983	TR23756|c1_g1_i1|CDS2	70
9	66.84	66.84	72.81000018	71.929997	71.92999721	TR14746|c0_g1_i1|CDS1	85
10	66.64	66.64	74.44999814	71.05	71.05000019	TR23970|c2_g1_i1|CDS1	84
11	61.45	61.45	83.91000032	74.779999	74.77999926	TR18660|c1_g1_i1|CDS1	93
12	56.6	56.6	76.3899982	65.280002	60.8799994	TR15609|c0_g1_i1|CDS1	46
13	53.84	59.08	83.32999945	75.739998	71.81000113	TR22071|c0_g1_i1|CDS2	40
14	52.15	52.15	87.1900022	80.070001	76.87000036	TR23770|c4_g2_i1|CDS2	47
15	50.56	50.56	75.18000007	59.850001	59.85000134	TR22931|c0_g3_i1|CDS1	92
16	50.46	52.62	87.1999979	70.770001	63.28999996	TR24398|c1_g1_i8|CDS2	49
17	47.89	47.89	99.32000041	91.890001	90.53999782	TR23597|c1_g3_i3|CDS3	103
18	47.61	50.29	81.08000159	71.429998	71.42999768	TR23770|c4_g1_i1|CDS2	65
18	0	50.29	81.70999885	75.199997	75.19999743	TR23770|c3_g1_i1|CDS1	65
19	43.42	45.52	69.599998	63.18	57.23999739	TR24122|c1_g2_i3|CDS2	48
20	43.13	51.17	68.45999956	61.680001	58.88000131	TR20169|c0_g1_i1|CDS1	64
21	41.2	41.2	70.70000172	56.900001	51.81999803	TR23612|c0_g1_i2|CDS1	32
22	40.73	40.73	65.21999836	59.780002	59.78000164	TR19230|c0_g1_i1|CDS1	63
22	0	40.73	82.19000101	75.340003	75.34000278	TR14522|c1_g2_i1|CDS2	63
23	40.61	40.61	86.36000156	86.360002	81.36000037	TR18707|c0_g1_i1|CDS1	43
24	39.82	39.84	68.22000146	67.290002	63.55000138	TR23597|c1_g8_i1|CDS1	78
24	0	39.84	68.22000146	67.290002	63.55000138	TR23597|c1_g4_i1|CDS1	78
24	0	39.84	91.8900013	91.890001	91.8900013	TR23597|c1_g6_i1|CDS1	78
25	39.58	41.43	93.07000041	75.25	75.24999976	TR18770|c0_g1_i1|CDS1	42
26	39.42	39.42	89.88999724	75.800002	62.5	TR14535|c0_g1_i1|CDS3	24
26	0	39.42	88.55999708	75.800002	62.5	TR18128|c0_g1_i1|CDS2	24
27	38.2	38.2	94.80000138	84.390002	84.39000249	TR21139|c1_g1_i1|CDS3	67
28	37.88	37.88	81.4800024	62.959999	62.95999885	TR17133|c3_g1_i1|CDS1	44
29	37.19	37.19	57.0299983	52.969998	51.0800004	TR22084|c0_g1_i1|CDS1	26
30	36.78	36.78	69.37999725	59.549999	59.54999924	TR20255|c0_g1_i1|CDS1	25
31	36.14	36.14	69.48000193	65.41	61.33999825	TR20170|c1_g1_i1|CDS1	27
32	35.45	35.45	73.22000265	69.04	65.2700007	TR23063|c0_g1_i2|CDS1	31
33	35.17	42.29	70.42000294	55.500001	48.4100014	TR22816|c0_g1_i1|CDS1	26
34	34.65	34.68	76.46999955	69.800001	67.83999801	TR17674|c0_g1_i1|CDS2	40

35	34.38	39.41	87.15999722	84.399998	82.10999966	TR18693|c0_g1_i1|CDS1	33
36	34.16	34.16	95.34999728	89.529997	89.52999711	TR20046|c0_g1_i2|CDS1	58
37	34.02	34.02	80.37999868	66.790003	66.79000258	TR20969|c0_g1_i1|CDS1	40
38	33.38	37.41	85.55999994	76.169997	76.16999745	TR21550|c0_g1_i1|CDS1	34
39	33.15	33.15	85.85000038	80.190003	77.35999823	TR18613|c0_g1_i1|CDS1	30
40	32.97	32.97	54.60000038	48.469999	40.70000052	TR24034|c3_g1_i4|CDS1	16
41	32.34	32.34	69.37000155	59.689999	59.16000009	TR15320|c0_g1_i1|CDS3	29
42	32.02	32.02	61.43000126	61.430001	61.43000126	TR17861|c0_g1_i2|CDS2	30
43	30.43	30.43	49.70000088	39.08	31.8599999	TR18551|c0_g1_i1|CDS2	22
44	29.26	29.28	84.65999961	81.599998	73.00999761	TR23419|c0_g15_i1|CDS1	53
45	28.75	63.57	96.82999849	90.499997	90.49999714	TR24861|c1_g19_i4|CDS1	110
46	27.9	27.9	67.61000156	61.269999	61.26999855	TR24569|c0_g6_i1|CDS1	27
47	27.87	31.85	87.63999939	81.650001	79.03000116	TR23063|c0_g1_i3|CDS1	31
48	26.66	26.66	97.46000171	97.460002	91.53000116	TR5441|c0_g2_i1|CDS1	34
49	26.29	32.84	76.03999972	67.71	60.93999743	TR24122|c1_g2_i5|CDS1	36
50	26.01	26.01	81.97000027	81.97	80.33000231	TR19632|c0_g2_i1|CDS1	34
51	25.91	25.91	57.31999874	51.050001	48.53999913	TR19364|c1_g1_i1|CDS1	21
52	25.7	25.7	77.68999934	64.880002	62.40000129	TR23050|c0_g2_i1|CDS7	14
53	25.59	25.59	86.9599998	74.529999	70.19000053	TR24569|c0_g3_i1|CDS2	18
53	0	24.76	84.46999788	74.529999	64.60000277	TR24569|c0_g3_i2|CDS2	17
54	24.93	32.44	91.39999747	87.099999	87.09999919	TR13307|c0_g3_i1|CDS1	76
54	0	32.44	91.39999747	87.099999	87.09999919	TR13307|c0_g2_i1|CDS1	76
55	24.81	24.81	78.22999954	70.069999	70.0699985	TR20889|c1_g2_i2|CDS2	25
55	0	24.81	78.22999954	70.069999	70.0699985	TR20889|c1_g2_i1|CDS1	25
56	24.7	24.93	65.63000083	61.979997	58.32999945	TR24403|c1_g2_i2|CDS1	28
57	24.59	24.71	77.54999995	63.950002	59.85999703	TR10278|c0_g1_i1|CDS1	25
57	0.03	24.71	77.54999995	63.950002	59.85999703	TR10278|c0_g3_i1|CDS1	23
58	23.35	29.12	81.51999712	76.300001	70.1399982	TR7566|c0_g2_i1|CDS1	39
58	0	27.08	81.51999712	73.460001	67.29999781	TR7566|c0_g1_i1|CDS1	38
59	23.04	30.26	90.71999788	82.47	68.04000139	TR18770|c2_g11_i1|CDS1	36
59	0	30.26	89.28999901	78.570002	78.57000232	TR18770|c2_g2_i1|CDS1	36
60	22.92	34.76	59.52000022	46.900001	36.43000126	TR17723|c0_g1_i1|CDS1	26
61	22.75	22.95	81.51999712	81.519997	68.48000288	TR15592|c0_g1_i1|CDS2	24
62	22.04	36.47	94.37000155	88.730001	88.73000145	TR24670|c2_g1_i1|CDS2	119
63	21.89	23.59	78.94999981	57.889998	56.58000112	TR17276|c0_g1_i1|CDS2	19
64	21.85	22.04	68.83999705	57.880002	57.88000226	TR20502|c0_g2_i1|CDS1	13
65	21.84	21.85	76.46999955	69.999999	69.99999881	TR20861|c1_g1_i1|CDS4	27
66	21.28	21.28	75.63999891	64.099997	64.09999728	TR14500|c0_g1_i1|CDS1	26
67	20.04	20.04	57.13999867	53.710002	53.71000171	TR16784|c0_g1_i1|CDS2	25
68	20.02	20.02	60.74000001	54.809999	54.80999947	TR19075|c0_g1_i2|CDS2	11
69	19.3	29.13	80.97000122	69.029999	69.02999878	TR24122|c1_g2_i6|CDS3	28
69	0	29.13	80.97000122	69.029999	69.02999878	TR24122|c1_g2_i1|CDS2	28
70	19.24	21.42	85.29000282	85.290003	83.32999945	TR24569|c0_g10_i1|CDS2	15
71	19.23	19.23	56.27999902	45.899999	43.16999912	TR16224|c0_g1_i1|CDS1	16
72	19.04	25.04	85.32000184	85.320002	85.32000184	TR24670|c2_g4_i1|CDS1	38

73	18.61	18.61	59.78999734	56.699997	56.69999719	TR21070|c0_g4_i1|CDS2	19
73	0	18.61	59.78999734	56.699997	56.69999719	TR20772|c0_g2_i1|CDS1	19
74	18	18	56.36000037	51.819998	51.81999803	TR10789|c0_g2_i1|CDS2	17
75	18	18	83.14999938	69.099998	69.09999847	TR19024|c0_g2_i2|CDS2	13
75	0	18	83.14999938	69.099998	69.09999847	TR19024|c0_g2_i1|CDS2	13
76	17.71	17.71	70.0699985	63.499999	54.00999784	TR24598|c0_g17_i2|CDS1	11
77	17.7	19.75	76.3199985	63.160002	61.83999777	TR20957|c0_g3_i1|CDS2	24
77	0	19.75	82.81000257	75	73.43999743	TR19470|c2_g2_i1|CDS2	24
77	0	19.75	71.61999941	64.859998	63.51000071	TR19470|c2_g10_i1|CDS1	24
78	17.55	17.55	55.07000089	55.070001	55.07000089	TR16094|c1_g1_i1|CDS1	19
79	17.26	18.22	55.93000054	34.18	27.68000066	TR21868|c2_g1_i1|CDS1	9
80	17.11	20.49	46.88999951	40.25	40.25000036	TR18753|c0_g2_i1|CDS1	11
81	16.75	16.79	41.17999971	35.879999	35.8799994	TR22070|c0_g1_i1|CDS2	8
82	16.35	17.81	78.38000059	47.299999	47.299999	TR20957|c0_g2_i1|CDS2	27
83	16.14	16.14	71.42999768	59.05	59.04999971	TR24598|c0_g7_i1|CDS1	11
83	0	16.14	71.42999768	59.05	59.04999971	TR24598|c0_g13_i1|CDS1	11
84	15.59	15.59	52.75999904	50.389999	50.38999915	TR697|c0_g1_i1|CDS5	11
85	15.52	15.52	81.29000068	66.909999	62.58999705	TR24693|c0_g25_i4|CDS1	9
86	15.34	35.75	77.45000124	60.780001	58.1700027	TR24398|c1_g1_i6|CDS1	22
87	15.24	15.24	82.78999925	52.460003	52.4600029	TR24693|c0_g24_i1|CDS2	12
88	14.99	14.99	78.75000238	57.499999	54.36999798	TR14597|c0_g1_i1|CDS3	12
89	14.84	34.62	97.86000252	95.190001	85.0300014	TR23970|c2_g2_i1|CDS2	42
90	14.74	14.74	84.76999998	54.970002	49.00999963	TR24676|c1_g20_i2|CDS1	18
91	14.32	31.74	86.14000082	86.140001	80.19999862	TR24861|c1_g24_i2|CDS1	77
92	14.07	15.35	49.41000044	32.06	28.2400012	TR24172|c0_g1_i4|CDS1	8
93	13.92	14.44	44.11999881	30.720001	28.09999883	TR24015|c0_g1_i5|CDS2	7
94	13.88	32.39	65.57000279	60.329998	55.4099977	TR24122|c1_g2_i4|CDS2	25
95	13.48	15.21	56.94000125	39.579999	37.85000145	TR21868|c1_g1_i1|CDS1	8
96	13.36	13.36	33.57999921	33.579999	33.57999921	TR20089|c0_g1_i1|CDS1	12
97	13.18	28.3	100	92.110002	92.11000204	TR16426|c0_g2_i1|CDS2	59
98	12.72	13.76	85.92000008	85.92	85.92000008	TR18690|c0_g6_i1|CDS2	34
98	0	15.13	77.4600029	77.460003	77.4600029	TR18690|c0_g3_i1|CDS1	34
98	0	13.76	85.92000008	85.92	85.92000008	TR18690|c0_g5_i1|CDS1	34
98	0	13.76	85.92000008	85.92	85.92000008	TR18690|c0_g1_i1|CDS1	34
98	0	10.78	85.92000008	85.92	56.33999705	TR18690|c0_g4_i1|CDS1	31
99	12.6	12.6	70.59000134	70.590001	63.73000145	TR24403|c1_g8_i2|CDS2	21
99	0	12.6	70.59000134	70.590001	63.73000145	TR24403|c1_g8_i1|CDS2	21
99	0	12.6	70.59000134	70.590001	63.73000145	TR10032|c1_g1_i1|CDS1	21
100	12.48	14.57	70.1300025	55.839998	55.83999753	TR20957|c0_g1_i1|CDS2	26
100	0	14.57	70.1300025	55.839998	55.83999753	TR19470|c2_g3_i1|CDS2	26
101	12.4	12.82	98.90999794	85.869998	80.43000102	TR23314|c3_g4_i2|CDS1	16
102	12.08	12.08	71.84000015	39.809999	39.80999887	TR21511|c0_g1_i6|CDS2	6
102	0	12.08	71.84000015	39.809999	39.80999887	TR21511|c0_g1_i5|CDS1	6
102	0	12.08	71.84000015	39.809999	39.80999887	TR21511|c0_g1_i4|CDS1	6
102	0	12.08	71.84000015	39.809999	39.80999887	TR21511|c0_g1_i3|CDS1	6

102	0	12.08	71.84000015	39.809999	39.80999887	TR21511|c0_g1_i2|CDS2	6
102	0	12.08	71.84000015	39.809999	39.80999887	TR21511|c0_g1_i1|CDS1	6
102	0	12.08	71.84000015	39.809999	39.80999887	TR11788|c0_g1_i1|CDS3	6
103	12.06	12.06	68.22999716	43.23	43.23000014	TR23050|c0_g2_i1|CDS1	9
104	11.85	11.87	59.21000242	56.580001	56.58000112	TR17276|c0_g3_i1|CDS2	13
105	11.77	11.77	28.24999988	23.09	23.09000045	TR24353|c1_g2_i2|CDS1	7
105	0	9.74	28.0400008	21.439999	21.43999934	TR24353|c1_g2_i3|CDS1	6
106	11.54	14	89.60999846	89.609998	89.60999846	TR19498|c0_g4_i1|CDS1	10
106	0.01	14	89.60999846	89.609998	89.60999846	TR19498|c0_g3_i1|CDS1	12
107	11.2	11.2	37.05999851	30.989999	29.06999886	TR20632|c0_g1_i1|CDS1	6
108	11.11	11.11	38.40000033	25.2	25.20000041	TR27662|c0_g1_i1|CDS3	6
109	10.73	22	95.08000016	88.520002	88.52000237	TR30849|c0_g1_i1|CDS2	19
110	10.55	10.55	45.41000128	42.359999	36.68000102	TR20240|c0_g1_i1|CDS1	11
111	10.42	17.46	54.89000082	54.890001	51.09000206	TR21550|c0_g2_i1|CDS1	12
112	10.26	14.32	74.36000109	61.540002	51.27999783	TR11265|c0_g1_i1|CDS1	10
113	10.09	10.09	52.63000131	52.630001	52.63000131	TR18971|c0_g13_i1|CDS1	9
113	0	9.78	52.63000131	52.630001	52.63000131	TR18971|c0_g15_i1|CDS1	9
113	0	8.01	43.84000003	43.84	43.84000003	TR18971|c0_g6_i1|CDS3	8
113	0	8.01	43.84000003	43.84	43.84000003	TR18971|c0_g3_i1|CDS2	8
114	10.01	10.01	26.64000094	12.87	12.8700003	TR21895|c0_g1_i1|CDS1	5
115	10	10	81.40000105	30.230001	30.23000062	TR6481|c0_g2_i1|CDS1	9
115	0	10	44.87000108	33.329999	33.32999945	TR16482|c0_g1_i1|CDS1	9
115	0	10	45.59000134	38.240001	38.24000061	TR6481|c0_g1_i1|CDS1	9
116	10	10	62.33999729	62.339997	62.33999729	TR4154|c0_g2_i1|CDS2	5
116	0	8	61.04000211	61.040002	61.04000211	TR4154|c0_g1_i1|CDS2	4
117	10	10	25.67000091	20.299999	20.29999942	TR22181|c0_g1_i1|CDS2	5
118	10	10	26.08999908	12.5	12.5	TR13710|c0_g1_i1|CDS1	6
119	10	10	61.90000176	53.170002	53.17000151	TR7650|c0_g1_i1|CDS2	5
120	10	10	56.02999926	56.029999	56.02999926	TR7596|c0_g2_i1|CDS2	12
121	10	10	37.16000021	37.16	37.16000021	TR22217|c0_g1_i2|CDS1	7
121	0	10	37.16000021	37.16	37.16000021	TR22217|c0_g1_i1|CDS1	7
122	9.89	9.89	43.23999882	43.239999	43.23999882	TR17389|c0_g1_i1|CDS1	5
123	9.85	9.85	42.86000133	41.56	41.56000018	TR13891|c0_g1_i1|CDS2	9
124	9.71	30.54	68.56999993	68.57	68.56999993	TR18979|c0_g1_i1|CDS1	47
125	9.52	9.52	37.79000044	23.93	13.52999955	TR24679|c0_g6_i1|CDS2	6
126	9.22	9.22	59.57000256	59.570003	59.57000256	TR24403|c1_g2_i3|CDS1	7
127	8.96	8.96	64.46999907	64.469999	64.46999907	TR17575|c1_g2_i1|CDS2	11
127	0	8.96	64.46999907	64.469999	64.46999907	TR17575|c1_g1_i1|CDS2	11
127	0	8.96	64.46999907	64.469999	64.46999907	TR17575|c0_g1_i1|CDS1	11
128	8.7	8.7	46.2500006	37.5	37.5	TR18971|c0_g5_i1|CDS3	6
128	0	8.7	62.70999908	50.849998	50.849998	TR16575|c1_g1_i1|CDS1	6
129	8.64	10.78	51.45000219	29.480001	24.27999973	TR15180|c0_g1_i1|CDS2	6
130	8.51	21.34	69.73999739	57.889998	57.88999796	TR17276|c0_g2_i1|CDS1	20
131	8.34	13.89	93.75	67.860001	67.86000133	TR24693|c0_g13_i1|CDS1	9
132	8.22	8.22	48.03000093	33.860001	28.34999859	TR7977|c0_g2_i1|CDS2	6

132	0	8.22	49.1899997	34.68	29.03000116	TR12929|c0_g1_i1|CDS2	6
133	8.01	12.01	51.55000091	42.269999	35.56999862	TR20772|c0_g3_i1|CDS4	7
134	8	23.9	77.17000246	77.170002	71.64999843	TR24861|c1_g28_i1|CDS1	29
135	8	8	68.48999858	52.05	46.57999873	TR11442|c0_g1_i1|CDS1	5
136	8	8	63.1099999	29.13	29.12999988	TR23314|c2_g9_i1|CDS1	9
136	0	8	57.0200026	26.320001	26.32000148	TR23314|c2_g8_i1|CDS2	9
136	0	8	57.0200026	26.320001	26.32000148	TR23314|c2_g6_i1|CDS2	9
136	0	8	53.50999832	26.320001	26.32000148	TR23314|c2_g4_i1|CDS2	9
136	0	8	53.50999832	26.320001	26.32000148	TR23314|c2_g2_i1|CDS2	9
136	0	6.51	55.08000255	28.81	28.81000042	TR23314|c2_g3_i1|CDS1	7
137	8	8	27.03000009	16.859999	16.8599993	TR21936|c0_g1_i1|CDS4	4
138	8	8	91.43000245	67.619997	67.61999726	TR16190|c0_g2_i1|CDS3	7
139	7.86	7.86	31.99999928	18.4	18.40000004	TR17034|c0_g1_i1|CDS2	4
139	0	7.86	27.9700011	16.08	16.07999951	TR12023|c0_g3_i1|CDS2	4
139	0	7.86	27.9700011	16.08	16.07999951	TR12023|c0_g2_i1|CDS2	4
139	0	7.86	27.9700011	16.08	16.07999951	TR12023|c0_g1_i1|CDS2	4
140	7.74	7.74	35.2699995	28.729999	28.72999907	TR17179|c0_g1_i1|CDS3	5
141	7.72	7.72	85.71000099	78.570002	67.14000106	TR23314|c3_g4_i1|CDS1	12
142	7.49	7.49	33.79999995	28.7	24.07000065	TR21728|c0_g1_i1|CDS1	5
143	7.17	7.17	52.07999945	52.079999	52.07999945	TR23529|c0_g1_i2|CDS2	7
143	0	7.17	52.07999945	52.079999	52.07999945	TR23529|c0_g10_i2|CDS2	7
143	0	6	65.14999866	65.149999	65.14999866	TR23529|c0_g17_i1|CDS2	6
144	7.15	7.15	10.57000011	8.5900001	8.590000123	TR33137|c0_g1_i1|CDS1	4
144	0	5.06	13.39000016	6.696	6.69599995	TR22764|c1_g1_i1|CDS2	3
145	6.84	8.2	96.05000019	81.580001	81.58000112	TR24241|c0_g4_i1|CDS2	10
145	0	8.2	96.05000019	81.580001	81.58000112	TR24241|c0_g2_i1|CDS2	10
145	0	8.13	96.05000019	81.580001	81.58000112	TR24241|c0_g3_i1|CDS2	10
145	0	6.78	86.44000292	77.969998	77.96999812	TR24241|c0_g8_i1|CDS1	7
145	0	6.78	86.44000292	77.969998	77.96999812	TR24241|c0_g6_i1|CDS1	7
145	0	6.78	86.44000292	77.969998	77.96999812	TR24241|c0_g5_i1|CDS1	7
145	0	6.78	86.44000292	77.969998	77.96999812	TR24241|c0_g10_i1|CDS1	7
146	6.59	13.03	77.13999748	57.139999	52.13999748	TR24676|c1_g1_i1|CDS2	17
146	0	8.58	83.99999738	69.330001	68.00000072	TR24676|c1_g18_i1|CDS2	10
147	6.44	21.72	96.89999819	96.899998	70.53999901	TR24861|c1_g28_i2|CDS1	30
147	0	21.72	77.17000246	77.170002	71.64999843	TR24861|c1_g28_i3|CDS2	30
148	6.41	16.1	51.46999955	51.47	51.46999955	TR16094|c2_g1_i1|CDS1	46
149	6.38	6.38	52.17000246	36.230001	24.63999987	TR22155|c0_g6_i1|CDS1	3
150	6.29	10.33	68.4499979	66.070002	42.86000133	TR16285|c0_g1_i1|CDS1	5
151	6.28	8.35	84.61999893	53.850001	53.8500011	TR3944|c0_g1_i1|CDS2	6
151	0	6.36	84.61999893	51.279998	34.61999893	TR10447|c0_g2_i1|CDS2	4
151	0	6.36	84.61999893	51.279998	34.61999893	TR10447|c0_g1_i1|CDS2	4
151	0	6.36	92.41999984	60.610002	40.90999961	TR10447|c0_g3_i1|CDS2	4
152	6.23	6.23	23.01999927	23.019999	17.46000051	TR275|c0_g2_i1|CDS2	3
152	0	4.2	15.56999981	15.57	9.836000204	TR275|c0_g1_i1|CDS2	2
153	6.18	15.4	100	66.670001	66.67000055	TR24861|c1_g31_i1|CDS2	26

153	0	32.88	100	82.349998	82.34999776	TR20046|c0_g1_i3|CDS1	56
153	0	28.38	96.29999995	82.72	82.71999955	TR24861|c1_g20_i1|CDS1	47
153	0	16.41	100	66.670001	66.67000055	TR24861|c1_g9_i2|CDS2	27
153	0	16.41	100	66.670001	66.67000055	TR24861|c1_g9_i1|CDS3	27
153	0	14.27	87.5	71.880001	71.88000083	TR24861|c1_g8_i1|CDS2	26
154	6.13	6.19	26.19000077	9.9569999	8.224999905	TR19570|c0_g1_i1|CDS1	3
154	0	6.19	26.19000077	9.9569999	8.224999905	TR18872|c0_g1_i1|CDS2	3
155	6.11	20.25	74.36000109	64.099997	64.09999728	TR15501|c0_g1_i1|CDS1	22
155	0	20.41	75.63999891	64.099997	64.09999728	TR15501|c0_g3_i1|CDS1	23
155	0	20.25	74.36000109	64.099997	64.09999728	TR14500|c0_g2_i1|CDS1	22
156	6.11	6.11	51.34999752	45.050001	39.64000046	TR991|c0_g1_i1|CDS1	15
157	6.07	40.24	63.67999911	55.690002	54.24000025	TR23612|c0_g1_i1|CDS1	34
158	6.07	15.06	83.79999995	61.970001	54.22999859	TR24598|c0_g18_i1|CDS2	8
158	0	12.95	94.67999935	71.280003	71.28000259	TR24598|c0_g4_i1|CDS2	7
159	6.07	6.07	34.09000039	30.3	30.30000031	TR13276|c0_g1_i2|CDS2	4
159	0	4.05	29.06000018	18.799999	18.79999936	TR13276|c0_g1_i1|CDS3	3
160	6.06	6.06	54.17000055	40.000001	40.0000006	TR13655|c0_g2_i1|CDS1	3
160	0	6.06	48.33000004	40.000001	40.0000006	TR13655|c0_g1_i1|CDS1	3
161	6.02	16.11	67.61999726	52.380002	52.38000154	TR24676|c1_g12_i1|CDS1	22
161	0.01	20.52	56.8599999	47.710001	47.71000147	TR24676|c1_g4_i1|CDS1	24
161	0	14.1	55.23999929	40.000001	40.0000006	TR24676|c1_g16_i1|CDS1	18
162	6.01	17.08	93.33000183	81.330001	81.33000135	TR24861|c1_g23_i1|CDS3	27
162	0	13.04	67.69000292	67.690003	67.69000292	TR24861|c1_g26_i1|CDS3	18
163	6	40.08	99.08000231	95.410001	87.15999722	TR24670|c2_g29_i1|CDS2	92
164	6	32.84	77.07999945	69.79	64.06000257	TR24122|c1_g2_i2|CDS2	39
165	6	31.72	96.50999904	89.529997	89.52999711	TR24861|c1_g18_i1|CDS1	44
166	6	31.5	77.53000259	73.409998	70.03999949	TR23063|c0_g1_i5|CDS1	29
167	6	15.24	68.56999993	58.099997	58.09999704	TR24693|c0_g4_i1|CDS2	10
167	0	14.03	67.77999997	54.439998	54.43999767	TR24693|c0_g9_i1|CDS2	9
167	0	9.07	66.22999907	55.839998	55.83999753	TR24693|c0_g6_i1|CDS2	6
167	0	6.07	62.30000257	52.460003	52.4600029	TR24693|c0_g27_i1|CDS2	3
167	0	6.07	62.30000257	52.460003	52.4600029	TR24693|c0_g20_i1|CDS2	3
167	0	4	39.21999931	39.219999	39.21999931	TR24693|c0_g14_i1|CDS2	2
168	6	14	78.22999954	59.179997	59.17999744	TR20889|c1_g1_i1|CDS1	17
168	0	14	72.72999883	53.149998	53.14999819	TR20889|c1_g1_i2|CDS1	17
169	6	8	33.6499989	33.649999	33.6499989	TR13177|c0_g3_i1|CDS2	4
169	0	8	33.6499989	33.649999	33.6499989	TR13177|c0_g1_i1|CDS2	4
169	0	6	42.86000133	42.860001	42.86000133	TR13177|c0_g2_i1|CDS2	3
170	6	6	23.17000031	12.93	10.72999984	TR14264|c0_g1_i1|CDS1	3
170	0	6	19.07999963	11.62	9.649000317	TR23903|c0_g4_i2|CDS2	3
170	0	6	19.2900002	11.75	9.75600034	TR23903|c0_g4_i1|CDS3	3
171	6	6	64.37000036	64.37	64.37000036	TR16754|c0_g1_i1|CDS1	4
172	6	6	45.95000148	45.950001	45.95000148	TR16413|c1_g1_i1|CDS1	4
173	5.92	8	73.55999947	54.02	54.0199995	TR24693|c0_g12_i1|CDS1	4
174	5.87	18.1	68.41999888	61.839998	61.83999777	TR19470|c2_g5_i1|CDS2	18

175	5.82	12	60.00000238	52.219999	52.21999884	TR19498|c0_g1_i1|CDS1	8
176	5.47	5.47	42.96000004	25.929999	25.92999935	TR19643|c0_g1_i2|CDS8	3
176	0	5.47	42.96000004	25.929999	25.92999935	TR19643|c0_g1_i1|CDS3	3
177	5.39	12.52	85.58999896	66.949999	55.93000054	TR17681|c0_g1_i1|CDS2	11
177	0	12.12	76.27000213	47.459999	42.37000048	TR17681|c0_g3_i1|CDS2	10
178	5.13	34.96	91.67000055	87.040001	87.04000115	TR19010|c0_g2_i1|CDS1	50
179	4.87	17.57	82.69000053	62.5	55.76999784	TR19970|c0_g9_i1|CDS1	16
179	0	17.57	82.69000053	62.5	55.76999784	TR19970|c0_g1_i2|CDS1	16
179	0	17.57	58.03999901	58.039999	51.78999901	TR19970|c0_g5_i1|CDS1	16
179	0	8.99	36.35999858	36.359999	36.35999858	TR19970|c0_g1_i1|CDS1	6
180	4.83	16.94	68.83999705	44.220001	34.67000127	TR24403|c1_g2_i1|CDS3	18
181	4.57	20.38	82.38999844	76.059997	64.07999992	TR24569|c0_g14_i1|CDS1	16
182	4.37	48.59	85.50999761	67.390001	59.89999771	TR24398|c1_g1_i9|CDS2	40
182	0	47.43	81.63999915	67.869997	60.39000154	TR24398|c1_g1_i7|CDS1	39
182	0	45.43	71.00999951	57.969999	50.48000216	TR24398|c1_g1_i5|CDS1	38
182	0	32.1	69.80999708	52.170002	42.75000095	TR24398|c1_g1_i2|CDS1	19
183	4.3	19.4	82.08000064	82.080001	80.19000292	TR24569|c0_g10_i4|CDS2	13
183	0	17.33	90.63000083	80.21	78.13000083	TR24569|c0_g12_i1|CDS1	12
184	4.24	4.29	34.61999893	24.04	20.19000053	TR18837|c0_g4_i1|CDS2	3
184	0	4.28	34.61999893	24.04	24.04000014	TR18837|c0_g5_i1|CDS2	3
184	0	4.28	58.06000233	40.32	40.32000005	TR18837|c0_g3_i1|CDS2	3
185	4.23	13.29	82.0900023	48.51	40.29999971	TR24676|c1_g15_i2|CDS1	10
185	0	10.77	68.48999858	52.05	45.21000087	TR24676|c1_g20_i1|CDS2	8
185	0	9.13	54.79000211	35.620001	35.62000096	TR24676|c1_g15_i1|CDS2	7
186	4.11	44.33	100	100	100	TR23597|c1_g3_i7|CDS3	116
187	4.1	19.7	75.3099978	75.309998	75.3099978	TR24861|c1_g3_i1|CDS3	30
188	4.08	5.8	26.01999938	12.57	12.56999969	TR14903|c0_g1_i1|CDS1	3
189	4.06	6.26	78.32999825	78.329998	66.67000055	TR24569|c0_g28_i1|CDS2	4
190	4.04	4.04	14.49999958	8.3970003	8.397000283	TR6338|c0_g1_i1|CDS3	2
190	0.02	2.09	20.40999979	7.3469996	4.081999883	TR11559|c0_g1_i6|CDS2	1
190	0.02	2.09	20.40999979	7.3469996	4.081999883	TR11559|c0_g1_i1|CDS2	1
190	0.02	2.09	16.32999927	7.3469996	4.081999883	TR11559|c0_g1_i5|CDS2	1
190	0.02	2.09	16.32999927	7.3469996	4.081999883	TR11559|c0_g1_i3|CDS1	1
190	0.02	2.09	16.32999927	7.3469996	4.081999883	TR11559|c0_g1_i2|CDS2	1
191	4.03	4.03	25.45000017	16.36	16.35999978	TR15111|c0_g1_i1|CDS1	2
192	4.02	24.07	85.61000228	85.610002	84.09000039	TR19632|c1_g1_i1|CDS1	34
193	4	27.95	76.74000263	76.740003	76.74000263	TR1026|c0_g1_i1|CDS1	31
194	4	7.17	44.94999945	44.949999	44.94999945	TR23529|c0_g1_i1|CDS2	9
194	0	7.17	41.87999964	41.88	41.87999964	TR23529|c0_g13_i1|CDS1	9
194	0	4	55.1699996	55.17	55.1699996	TR23529|c0_g14_i1|CDS2	4
194	0	4	62.74999976	62.75	62.74999976	TR23529|c0_g12_i1|CDS2	4
194	0	4	62.74999976	62.75	62.74999976	TR23529|c0_g11_i2|CDS1	4
194	0	4	62.74999976	62.75	62.74999976	TR23529|c0_g11_i1|CDS2	4
195	4	7.12	70.14999986	62.690002	62.69000173	TR23314|c3_g1_i1|CDS2	7
196	4	6	44.92999911	44.929999	44.92999911	TR16094|c2_g3_i1|CDS1	14

197	4	4.44	24.74000007	21.65	14.42999989	TR24403|c1_g7_i1|CDS1	4
198	4	4	17.08000004	3.0130001	2.455000021	TR15597|c0_g1_i1|CDS3	2
198	0	2	63.2700026	10.2	10.19999981	TR7556|c0_g2_i1|CDS1	1
198	0	2	11.14000008	3.2609999	3.260999918	TR7187|c0_g1_i1|CDS1	1
198	0	2	65.96000195	21.28	21.27999961	TR7556|c0_g1_i1|CDS2	1
199	4	4	28.2400012	25.569999	18.32000017	TR30852|c0_g1_i1|CDS1	2
200	4	4	31.25	18.23	18.23000014	TR23986|c0_g2_i1|CDS3	2
200	0	2	12.16000021	12.16	12.16000021	TR23986|c0_g8_i1|CDS3	1
201	4	4	55.93000054	16.38	16.38000011	TR23538|c0_g1_i2|CDS1	2
201	0	2	12.24000007	12.24	12.24000007	TR23538|c1_g2_i1|CDS4	1
202	4	4	21.60000056	12.68	12.68000007	TR16002|c0_g1_i2|CDS3	2
202	0	4	25.13999939	14.749999	14.74999934	TR16002|c0_g1_i1|CDS3	2
203	4	4	18.75	18.75	18.75	TR20858|c0_g1_i2|CDS1	2
203	0	4	18.49000007	18.49	18.49000007	TR20858|c0_g1_i1|CDS2	2
203	0	2	21.53999954	21.54	21.53999954	TR1389|c1_g1_i1|CDS2	1
204	4	4	22.73000032	22.73	22.73000032	TR17671|c0_g1_i2|CDS1	3
204	0	4	20.7100004	20.71	20.7100004	TR17671|c0_g1_i1|CDS2	3
205	3.9	25.54	68.27999949	65.05	56.98999763	TR23419|c0_g6_i1|CDS1	30
206	3.7	26.49	78.45000029	77.590001	57.76000023	TR23597|c1_g9_i2|CDS1	64
206	0	32.7	94.51000094	94.510001	73.62999916	TR23597|c1_g8_i2|CDS1	81
206	0	27.79	71.28999829	67.330003	67.33000278	TR24670|c2_g11_i1|CDS4	64
206	0	26.49	77.59000063	74.14	57.76000023	TR23597|c1_g9_i3|CDS1	64
206	0	17.14	62.69000173	56.720001	56.72000051	TR24670|c2_g5_i1|CDS4	39
207	3.68	3.68	54.10000086	54.100001	40.9799993	TR23538|c1_g1_i1|CDS2	3
207	0	3.61	49.18000102	39.340001	26.23000145	TR23538|c1_g5_i1|CDS2	2
207	0	1.91	47.87999988	10.3	10.30000001	TR23538|c1_g6_i1|CDS2	2
208	3.4	3.4	16.45999998	9.4509996	6.706999987	TR23314|c3_g3_i1|CDS1	2
208	0	2.93	21.17000073	11.04	6.747999787	TR23314|c3_g2_i1|CDS1	2
208	0	2.86	46.59999907	21.359999	21.35999948	TR23314|c0_g2_i1|CDS1	2
208	0	2.86	46.59999907	21.359999	21.35999948	TR23314|c0_g1_i1|CDS1	2
209	3.39	5.48	43.4799999	43.48	43.4799999	TR16094|c2_g2_i1|CDS1	11
209	0	5.48	43.4799999	43.48	43.4799999	TR16094|c1_g2_i2|CDS2	11
209	0	5.48	43.4799999	43.48	43.4799999	TR16094|c1_g2_i1|CDS2	11
210	3.19	30.14	68.56999993	68.57	68.56999993	TR17861|c0_g1_i1|CDS3	30
211	3.12	18.28	72.72999883	62.629998	62.62999773	TR21139|c0_g1_i1|CDS1	30
212	2.94	3.02	79.68999743	79.689997	48.44000041	TR7070|c0_g1_i1|CDS1	3
212	0	2.58	68.75	68.75	48.44000041	TR7070|c0_g2_i1|CDS1	3
213	2.92	2.92	67.86000133	35.710001	35.71000099	TR19261|c0_g2_i1|CDS1	3
213	0	2.92	31.67999983	19.8	19.79999989	TR19261|c0_g3_i1|CDS1	3
214	2.89	2.89	33.32999945	25.639999	17.94999987	TR24679|c0_g4_i1|CDS2	2
215	2.85	5.54	32.74999857	10.53	7.602000237	TR23549|c0_g3_i1|CDS1	4
215	0.01	4.98	34.09000039	10.8	7.954999804	TR23549|c0_g3_i3|CDS1	4
215	0	2.9	20.74999958	20.75	20.74999958	TR23549|c0_g2_i1|CDS3	2
216	2.74	4.81	48.60999882	47.220001	47.22000062	TR24861|c0_g1_i1|CDS1	9
217	2.55	26.72	83.7199986	79.070002	74.41999912	TR23419|c0_g3_i1|CDS2	25

218	2.47	10.69	71.42999768	71.429998	63.63999844	TR24598|c0_g17_i1|CDS1	7
218	0	8.66	73.01999927	73.019999	63.48999739	TR24598|c0_g1_i1|CDS1	6
218	0	8	72.72999883	72.729999	72.72999883	TR24598|c0_g16_i1|CDS1	6
219	2.44	75.18	91.3500011	86.860001	86.86000109	TR23240|c0_g1_i2|CDS2	132
220	2.41	17.7	71.64000273	71.640003	70.14999986	TR19470|c2_g8_i1|CDS2	21
221	2.38	6.51	87.5	87.5	87.5	TR24569|c0_g10_i2|CDS1	7
222	2.37	2.37	26.19999945	11.23	6.417000294	TR8545|c0_g1_i1|CDS1	1
223	2.34	29.18	71.02000117	64.490002	62.44999766	TR23063|c0_g1_i1|CDS1	31
224	2.28	11.93	54.25999761	41.49	41.49000049	TR24693|c0_g26_i1|CDS1	8
225	2.27	14.96	61.76000237	49.020001	42.1600014	TR24569|c0_g27_i1|CDS2	12
225	0	8.11	64.78999853	46.48	36.62	TR24569|c0_g17_i1|CDS2	8
225	0	8.11	52.10999846	46.48	36.62	TR24569|c0_g1_i1|CDS2	8
226	2.23	2.71	63.74999881	23.75	23.7499997	TR10208|c0_g1_i1|CDS2	3
226	0	2.71	23.7499997	23.75	23.7499997	TR10208|c0_g2_i1|CDS2	3
227	2.2	2.2	40.4399991	9.5590003	5.14700003	TR12392|c0_g1_i1|CDS3	1
227	0	2	44.67999935	14.89	14.89000022	TR16242|c0_g1_i1|CDS3	1
227	0	2	14.89000022	14.89	14.89000022	TR18411|c0_g1_i2|CDS5	1
227	0	2	14.89000022	14.89	14.89000022	TR18411|c0_g1_i1|CDS5	1
228	2.17	2.17	32.35000074	13.240001	13.24000061	TR7755|c0_g1_i1|CDS2	2
229	2.15	8	91.43000245	67.619997	67.61999726	TR16190|c0_g1_i1|CDS3	7
230	2.14	2.14	11.25999987	1.513	0.924399961	TR22297|c0_g1_i1|CDS1	2
230	0	2.09	78.13000083	28.130001	17.19000041	TR17429|c0_g1_i1|CDS2	2
230	0	2	26.55999959	17.19	17.19000041	TR17429|c0_g3_i1|CDS2	2
230	0	2	29.82000113	19.3	19.30000037	TR17429|c0_g2_i1|CDS2	2
231	2.12	28	71.78999782	71.789998	71.78999782	TR17133|c1_g1_i1|CDS1	36
233	2.1	2.1	21.53999954	21.54	18.45999956	TR20670|c1_g6_i4|CDS2	2
233	0	2.1	18.67000014	18.67	15.99999964	TR20670|c1_g6_i3|CDS2	2
234	2.09	7.19	65.68999887	45.100001	33.32999945	TR16766|c0_g2_i2|CDS1	6
234	0	12.16	72.28000164	31.52	25	TR24598|c0_g10_i1|CDS1	7
234	0	7.19	65.68999887	45.100001	33.32999945	TR16766|c0_g2_i1|CDS1	6
235	2.04	2.04	17.38999933	17.389999	17.38999933	TR20670|c1_g5_i1|CDS3	6
235	0	2.04	22.22000062	22.220001	22.22000062	TR20670|c1_g3_i1|CDS3	6
235	0	2.04	17.38999933	17.389999	17.38999933	TR20670|c1_g2_i1|CDS3	6
235	0	2.04	17.38999933	17.389999	17.38999933	TR20670|c0_g1_i1|CDS3	6
236	2.03	12.32	82.80000091	55.909997	44.08999979	TR24398|c0_g2_i1|CDS1	6
236	0	12.31	70.96999884	63.440001	44.08999979	TR24398|c0_g1_i1|CDS1	6
237	2.03	2.05	15.57999998	4.5230001	4.523000121	TR20664|c0_g1_i3|CDS1	1
237	0	2.04	12.83999979	3.303	3.302999958	TR20664|c0_g1_i1|CDS1	1
238	2.02	28.7	87.72000074	87.720001	75.44000149	TR24861|c1_g19_i6|CDS1	34
239	2.02	10.23	76.46999955	72.549999	72.54999876	TR24676|c1_g19_i1|CDS1	14
239	0	9.33	85.39000154	73.030001	73.03000093	TR24676|c0_g1_i1|CDS1	13
240	2.02	10	57.34999776	57.349998	57.34999776	TR24670|c2_g9_i1|CDS2	8
240	0	10	57.34999776	57.349998	57.34999776	TR24670|c2_g3_i1|CDS2	8
241	2.02	2.02	14.21999931	10.55	10.54999977	TR18570|c0_g2_i1|CDS1	1
242	2.01	37.72	72.89999723	67.290002	63.55000138	TR23597|c1_g5_i1|CDS1	71

242	0	31.58	84.42000151	83.12	76.6200006	TR23597|c1_g8_i4|CDS1	63
242	0	23.36	63.63999844	57.58	57.58000016	TR23597|c1_g1_i1|CDS1	48
243	2.01	6.08	70.17999887	61.400002	61.40000224	TR23419|c0_g11_i1|CDS1	16
244	2.01	2.01	41.67000055	9.3139999	3.430999815	TR23551|c0_g1_i2|CDS2	1
244	0	2.01	35.55999994	7.9499997	2.928999998	TR23551|c0_g1_i1|CDS1	1
245	2.01	2.01	24.07000065	8.642	8.64199996	TR15561|c0_g1_i7|CDS1	1
245	0	2.01	24.07000065	8.642	8.64199996	TR15561|c0_g1_i5|CDS1	1
245	0	2.01	24.07000065	8.642	8.64199996	TR15561|c0_g1_i2|CDS1	1
245	0	2.01	24.07000065	8.642	8.64199996	TR15561|c0_g1_i1|CDS1	1
245	0	2	22.13000059	11.48	11.47999987	TR15561|c0_g1_i6|CDS3	1
245	0	2	22.13000059	11.48	11.47999987	TR15561|c0_g1_i4|CDS3	1
245	0	2	22.13000059	11.48	11.47999987	TR15561|c0_g1_i3|CDS3	1
246	2.01	2.01	13.54999989	3.7379999	3.737999871	TR39793|c0_g1_i1|CDS1	1
247	2.01	2.01	12.78000027	6.1670002	6.16700016	TR18812|c0_g1_i1|CDS4	1
248	2.01	2.01	15.94000012	15.94	15.94000012	TR20670|c1_g6_i2|CDS3	3
248	0	2.01	15.94000012	15.94	15.94000012	TR20670|c1_g6_i1|CDS3	3
249	2	39	84.83999968	71.01	65.16000032	TR20070|c0_g1_i1|CDS4	25
249	0	39	84.83999968	71.01	65.16000032	TR18310|c0_g1_i2|CDS4	25
249	0	39	84.83999968	71.01	65.16000032	TR18310|c0_g1_i1|CDS4	25
250	2	35.75	80.43000102	63.910002	55.95999956	TR24398|c1_g1_i1|CDS1	21
251	2	30.03	100	92.110002	92.11000204	TR16426|c0_g1_i1|CDS2	66
252	2	27.19	71.60000205	67.460001	67.46000051	TR5610|c0_g1_i1|CDS1	34
253	2	26.49	77.59000063	74.14	57.76000023	TR23597|c1_g8_i3|CDS1	59
253	0	21.26	69.73999739	64.469999	64.46999907	TR24670|c2_g14_i1|CDS4	55
253	0	17.14	62.69000173	56.720001	56.72000051	TR24670|c2_g25_i1|CDS4	34
254	2	24.1	83.06000233	66.13	66.13000035	TR18660|c0_g1_i1|CDS1	29
255	2	23.04	88.41000199	60.140002	60.14000177	TR23970|c2_g2_i2|CDS1	20
255	0	8.79	78.7899971	51.520002	51.52000189	TR10196|c0_g1_i1|CDS1	7
256	2	16.11	81.70999885	80.489999	80.48999906	TR23756|c1_g1_i2|CDS1	12
257	2	14	89.60999846	89.609998	89.60999846	TR19498|c0_g2_i1|CDS1	11
258	2	12.25	92.93000102	91.92	90.90999961	TR24670|c2_g19_i1|CDS2	33
259	2	11.94	84.52000022	76.190001	67.86000133	TR24569|c0_g23_i1|CDS1	10
260	2	10.03	88.51000071	68.970001	68.97000074	TR24693|c0_g18_i1|CDS1	5
261	2	8	50	28.81	28.81000042	TR23314|c2_g1_i1|CDS1	9
262	2	6	55.32000065	54.259998	54.25999761	TR20502|c0_g3_i2|CDS1	3
262	0	6	57.77999759	56.669998	56.66999817	TR20502|c0_g3_i1|CDS1	3
262	0	6	57.77999759	56.669998	56.66999817	TR20502|c0_g1_i1|CDS1	3
263	2	4.8	56.33999705	56.339997	56.33999705	TR18690|c0_g2_i1|CDS2	16
264	2	4	52.9399991	52.939999	52.9399991	TR23419|c0_g5_i1|CDS2	9
264	0	4	63.16000223	63.160002	63.16000223	TR23419|c0_g18_i1|CDS2	9
264	0	4	63.16000223	63.160002	63.16000223	TR23419|c0_g12_i1|CDS2	9
264	0	4	63.16000223	63.160002	63.16000223	TR23419|c0_g10_i1|CDS2	9
265	2	2.09	56.94000125	40.279999	31.94000125	TR19532|c0_g2_i1|CDS2	1
265	0	2.09	58.32999945	40.279999	31.94000125	TR19532|c0_g1_i1|CDS2	1
265	0	2.09	50	40.279999	31.94000125	TR19532|c0_g3_i1|CDS2	1

266	2	2.03	10.00000015	2.857	2.857000008	TR15608|c0_g1_i1|CDS3	1
266	0	2	12.98999935	4.5200001	4.520000145	TR15608|c0_g1_i2|CDS1	1
266	0	2	34.92000103	12.7	12.70000041	TR18489|c0_g1_i1|CDS4	1
267	2	2.02	12.09999993	5.7319999	5.731999874	TR23186|c0_g4_i4|CDS1	1
267	0	2.02	10.61000004	6.8180002	6.818000227	TR23186|c0_g6_i1|CDS1	1
267	0	2.02	10.61000004	6.8180002	6.818000227	TR23186|c0_g5_i1|CDS1	1
267	0	2.02	8.917000145	5.7319999	5.731999874	TR23186|c0_g4_i2|CDS1	1
267	0	2.02	10.61000004	6.8180002	6.818000227	TR23186|c0_g2_i1|CDS1	1
267	0	2	21.87999934	14.06	14.05999959	TR23186|c0_g1_i1|CDS1	1
268	2	2	29.19000089	1.863	1.862999983	TR22055|c0_g1_i3|CDS1	1
268	0	2	26.06000006	1.664	1.664000005	TR22055|c0_g1_i2|CDS1	1
268	0	2	37.16000021	2.244	2.243999951	TR22055|c0_g1_i1|CDS1	1
268	0	2	22.20000029	1.6249999	1.62499994	TR22055|c0_g1_i4|CDS1	1
269	2	2	16.75000042	4.569	4.569000006	TR27975|c0_g1_i1|CDS2	1
270	2	2	32.85000026	11.68	7.299000025	TR8258|c0_g2_i1|CDS1	1
270	0	2	32.85000026	11.68	7.299000025	TR8258|c0_g1_i1|CDS1	1
270	0	2	36.59000099	13.01	8.129999787	TR10812|c0_g1_i1|CDS1	1
271	2	2	17.12000072	4.5049999	4.504999891	TR21637|c0_g3_i1|CDS4	1
272	2	2	8.433999866	2.7109999	2.710999921	TR21606|c0_g1_i1|CDS1	1
273	2	2	11.05000004	2.9069999	2.906999923	TR18366|c0_g1_i1|CDS1	1
275	2	2	23.81000072	19.840001	19.84000057	TR30943|c0_g1_i1|CDS1	1
276	2	2	15.79000056	9.2110001	9.211000055	TR19620|c0_g1_i1|CDS2	1
277	2	2	38.17999959	23.639999	23.63999933	TR15571|c0_g2_i1|CDS3	2
277	0	2	38.17999959	23.639999	23.63999933	TR15571|c0_g1_i1|CDS3	2
278	2	2	24.24000055	14.139999	14.13999945	TR15225|c0_g1_i1|CDS1	1
280	2	2	13.33000064	13.330001	13.33000064	TR9217|c0_g1_i1|CDS3	1
281	2	2	15.90999961	15.91	15.90999961	TR918|c0_g1_i1|CDS11	1
282	2	2	9.782999754	9.7829998	9.782999754	TR8170|c0_g2_i1|CDS2	1
282	0	2	9.782999754	9.7829998	9.782999754	TR8170|c0_g1_i1|CDS3	1
283	2	2	15.99999964	16	15.99999964	TR30993|c0_g1_i1|CDS1	1
284	2	2	13.86000067	13.860001	13.86000067	TR23986|c0_g7_i2|CDS2	1
284	0	2	9.928999841	9.9289998	9.928999841	TR23986|c0_g7_i1|CDS2	1
284	0	2	9.928999841	9.9289998	9.928999841	TR23986|c0_g6_i1|CDS2	1
284	0	2	9.928999841	9.9289998	9.928999841	TR23986|c0_g4_i1|CDS2	1
285	2	2	55.22000194	55.220002	55.22000194	TR23063|c1_g1_i1|CDS1	1
286	2	2	11.54000014	11.54	11.54000014	TR21689|c0_g1_i2|CDS6	1
286	0	2	11.54000014	11.54	11.54000014	TR21689|c0_g1_i1|CDS6	1
287	2	2	12.96000034	12.96	12.96000034	TR20398|c0_g1_i1|CDS3	1
288	2	2	2.212999947	2.2129999	2.212999947	TR17890|c0_g1_i1|CDS1	1
289	2	2	10.96000001	10.96	10.96000001	TR16368|c0_g1_i1|CDS4	1

BEST N	BEST Unused	BEST Total	BEST %Cov	BEST %Cov(50)	BEST %Cov(95)	Accession	BEST Peptides(95%)
49	0	10.64	43.04000139	43.040001	41.76999927	TR18770|c2_g7_i1|CDS1	12
30	0	2	7.207000256	7.2070003	7.207000256	TR11262|c0_g1_i1|CDS1	1
30	0	2	7.207000256	7.2070003	7.207000256	TR11262|c0_g2_i1|CDS1	1

30	0	2	7.207000256	7.2070003	7.207000256	TR11262|c0_g3_i1|CDS1	1
30	0	2	7.207000256	7.2070003	7.207000256	TR11262|c0_g4_i1|CDS1	1
20	0	4	34.25000012	34.25	34.25000012	TR11773|c0_g1_i1|CDS2	2
3	12.36	12.36	50	50	50	TR15501|c0_g2_i1|CDS1	9
56	0	8.22	51.27999783	51.279998	51.27999783	TR16575|c0_g1_i1|CDS3	7
18	0	3.42	22.85999954	21.9	21.89999968	TR17377|c0_g1_i1|CDS2	2
18	0	3.42	21.24000043	20.35	20.35000026	TR17377|c0_g4_i1|CDS2	2
61	0	1.36	11.11000031	11.11	11.11000031	TR18290|c0_g1_i1|CDS1	1
61	1.36	1.36	11.11000031	11.11	11.11000031	TR18290|c0_g1_i2|CDS1	1
25	2	10	50.49999952	44.549999	44.54999864	TR18770|c0_g2_i1|CDS1	6
5	0	10	51.46999955	51.47	51.46999955	TR18770|c2_g10_i1|CDS1	7
19	0	11.79	48.10000062	41.769999	41.76999927	TR18770|c2_g7_i1|CDS1	11
56	1.74	8.22	52.63000131	52.630001	52.63000131	TR18971|c0_g11_i1|CDS1	7
3	0	2	23.28999937	23.289999	23.28999937	TR18971|c0_g14_i1|CDS3	1
3	0	2	22.37000018	22.37	22.37000018	TR18971|c0_g2_i1|CDS1	1
3	0	2	22.37000018	22.37	22.37000018	TR18971|c0_g4_i1|CDS1	1
18	0	14.25	75.44999719	75.449997	69.09000278	TR19632|c0_g1_i1|CDS1	9
33	0	2.54	11.2999998	11.3	7.825999707	TR20240|c0_g1_i2|CDS1	1
40	2	14	46.38999999	43.3	43.29999983	TR20772|c0_g2_i2|CDS4	8
27	9.24	24.55	70.41000128	61.799997	61.79999709	TR23063|c0_g1_i4|CDS1	20
29	0	6	15.25000036	15.25	15.25000036	TR23314|c2_g5_i1|CDS1	6
29	0	6	15.25000036	15.25	15.25000036	TR23314|c2_g7_i1|CDS1	6
55	2.06	2.06	11.03999987	3.681	3.68099995	TR23314|c3_g2_i2|CDS1	1
35	0	8	54.17000055	54.170001	54.17000055	TR23419|c0_g16_i1|CDS1	5
35	0	8	53.60999703	53.609997	53.60999703	TR23419|c0_g16_i2|CDS1	5
31	0	2	33.32999945	33.329999	33.32999945	TR23419|c0_g7_i1|CDS2	1
33	0	1.52	2.082999982	2.083	2.082999982	TR23549|c0_g3_i2|CDS1	1
16	0	2	11.11000031	11.11	11.11000031	TR23597|c1_g4_i2|CDS1	1
18	0	2	11.11000031	11.11	11.11000031	TR23597|c1_g4_i2|CDS1	1
12	0	5.03	50	50	50	TR23597|c1_g7_i1|CDS1	4
52	0	10.84	59.21000242	59.210002	59.21000242	TR23597|c1_g9_i1|CDS1	10
42	0	4.01	10.33999994	6.0339998	6.033999845	TR24015|c0_g1_i1|CDS2	2
30	0.04	7.6	25.15999973	21.24	16.67000055	TR24015|c0_g1_i3|CDS2	4
42	0	4.01	10.17000005	5.9319999	5.931999907	TR24015|c0_g1_i4|CDS2	2
22	4.9	4.93	23.69000018	16.869999	16.86999947	TR24015|c0_g1_i7|CDS2	3
42	2	4.01	10.33999994	6.0339998	6.033999845	TR24015|c0_g1_i8|CDS2	2
13	0	7.88	16.42999947	14.99	12.68000007	TR24015|c0_g1_i9|CDS2	4
11	0	7.41	12.0099999	9.4690003	9.46900025	TR24034|c3_g1_i1|CDS1	4
11	0	7.41	13.6500001	10.76	10.76000035	TR24034|c3_g1_i2|CDS1	4
11	0	7.41	11.90000027	9.3819998	9.38199982	TR24034|c3_g1_i3|CDS1	4
31	5.89	5.9	10.53000018	8.4799998	8.479999751	TR24172|c0_g1_i1|CDS1	3
31	0	3.75	9.0640001	5.263	5.262999982	TR24172|c0_g1_i2|CDS1	2
35	0	4.02	18.05000007	15.16	12.63999939	TR24172|c0_g1_i3|CDS1	2
13	6	6	17.00000018	14.17	14.16999996	TR24172|c0_g1_i5|CDS1	3
31	0	3.75	10.11999995	7.2870001	7.28700012	TR24172|c0_g1_i6|CDS1	2

35	0	4.02	20.23999989	17	14.16999996	TR24172|c0_g1_i7|CDS1	2
5	20.16	20.36	45.41000128	35.27	33.57000053	TR24398|c1_g1_i3|CDS1	11
10	0	16.45	43.00000072	35.27	32.13	TR24398|c1_g1_i4|CDS1	10
49	0	10.07	68.12000275	65.219998	53.61999869	TR24569|c0_g16_i1|CDS2	8
49	0	9.87	43.68000031	43.68	42.53000021	TR24569|c0_g27_i2|CDS2	8
33	0	12.2	52.9399991	49.020001	41.17999971	TR24569|c0_g8_i1|CDS1	9
33	6.01	12.2	52.9399991	49.020001	41.17999971	TR24569|c0_g8_i2|CDS1	9
53	0	10.34	51.34999752	51.349998	51.34999752	TR24569|c0_g8_i3|CDS1	7
51	0	2.8	44.44000125	44.440001	31.74999952	TR24598|c0_g22_i1|CDS1	2
16	0	2	12.70000041	12.7	12.70000041	TR24670|c2_g10_i1|CDS2	1
16	0	2	13.1099999	13.11	13.1099999	TR24670|c2_g15_i1|CDS1	1
16	0	2	11.11000031	11.11	11.11000031	TR24670|c2_g17_i1|CDS1	1
16	0	2	11.11000031	11.11	11.11000031	TR24670|c2_g2_i1|CDS4	1
16	0	2	13.78999949	13.789999	13.78999949	TR24670|c2_g20_i1|CDS1	1
16	0	2	15.09000063	15.090001	15.09000063	TR24670|c2_g21_i1|CDS1	1
16	0	2	12.70000041	12.7	12.70000041	TR24670|c2_g23_i1|CDS2	1
12	0	5.03	45.32999992	45.33	45.32999992	TR24670|c2_g24_i1|CDS4	4
50	4	11.74	54.43999767	54.439998	43.32999885	TR24693|c0_g21_i1|CDS2	6

TABLE S3

Protein summary of Protein Pilot search of rpHPLC fraction 1 (Figure 2)

N	Unused	Total	%Cov	%Cov(50)	%Cov(95)	Accession	Peptides(95%)
1	18.64	18.64	67.14000106	50	50	TR18979|c0_g1_i1|CDS1	22
2	6	16.25	67.14000106	50	50	TR17861|c0_g1_i1|CDS3	11
3	6	6	26.73999965	26.74	26.73999965	TR6481|c0_g2_i1|CDS1	3
3	0	6	33.82000029	33.82	33.82000029	TR6481|c0_g1_i1|CDS1	3
3	0	6	29.49000001	29.49	29.49000001	TR16482|c0_g1_i1|CDS1	3
4	2	12.24	58.56999755	50	50	TR17861|c0_g1_i2|CDS2	9
5	2	2	7.156000286	3.303	3.302999958	TR20664|c0_g1_i1|CDS1	1
5	0	2	7.286000252	4.5230001	4.523000121	TR20664|c0_g1_i3|CDS1	1

TABLE S4

Protein summary of Protein Pilot search of rpHPLC fraction 2 (Figure 2)

N	Unused	Total	%Cov	%Cov(50)	%Cov(95)	Accession	Peptides(95%)
1	0	24.01	68.48999858	67.119998	67.11999774	TR14522|c1_g1_i1|CDS2	28
2	18.35	18.35	82.58000016	78.030002	72.72999883	TR19632|c1_g1_i1|CDS1	10
3	12.12	12.12	62.13999987	39.809999	39.80999887	TR21511|c0_g1_i6|CDS2	7
3	0	12.12	62.13999987	39.809999	39.80999887	TR21511|c0_g1_i5|CDS1	7
3	0	12.12	62.13999987	39.809999	39.80999887	TR21511|c0_g1_i4|CDS1	7
3	0	12.12	62.13999987	39.809999	39.80999887	TR21511|c0_g1_i3|CDS1	7
3	0	12.12	62.13999987	39.809999	39.80999887	TR21511|c0_g1_i2|CDS2	7
3	0	12.12	62.13999987	39.809999	39.80999887	TR21511|c0_g1_i1|CDS1	7
3	0	12.12	62.13999987	39.809999	39.80999887	TR11788|c0_g1_i1|CDS3	7
4	12.01	12.01	50	50	42.73000062	TR10789|c0_g2_i1|CDS2	6
5	8.32	8.32	33.86000097	33.070001	27.55999863	TR7977|c0_g2_i1|CDS2	5
5	0	8.32	34.67999995	33.87	28.22999954	TR12929|c0_g1_i1|CDS2	5
5	0	6.27	47.76000082	46.270001	35.82000136	TR7977|c0_g1_i1|CDS1	3
6	8	8	18.40000004	18.4	18.40000004	TR17034|c0_g1_i1|CDS2	4
6	0	8	16.07999951	16.08	16.07999951	TR12023|c0_g3_i1|CDS2	4
6	0	8	16.07999951	16.08	16.07999951	TR12023|c0_g2_i1|CDS2	4
6	0	8	16.07999951	16.08	16.07999951	TR12023|c0_g1_i1|CDS2	4
7	8	8	8.590000123	8.5900001	8.590000123	TR33137|c0_g1_i1|CDS1	4
7	0	6	6.69599995	6.696	6.69599995	TR22764|c1_g1_i1|CDS2	3
8	6.15	6.15	60.53000093	35.530001	27.63000131	TR17276|c0_g2_i1|CDS1	3
9	6.01	6.01	46.57999873	36.989999	36.98999882	TR11442|c0_g1_i1|CDS1	3
10	4.15	4.15	10.61000004	6.7100003	4.978	TR19570|c0_g1_i1|CDS1	2
10	0	4.15	10.61000004	6.7100003	4.978	TR18872|c0_g1_i1|CDS2	2
11	4.06	4.06	11.45000011	8.3970003	8.397000283	TR6338|c0_g1_i1|CDS3	2
11	0	2.13	13.47000003	4.0819999	4.081999883	TR11559|c0_g1_i5|CDS2	1
11	0	2.13	13.47000003	4.0819999	4.081999883	TR11559|c0_g1_i3|CDS1	1
11	0	2.13	13.47000003	4.0819999	4.081999883	TR11559|c0_g1_i2|CDS2	1
11	0	2.13	13.47000003	4.0819999	4.081999883	TR11559|c0_g1_i1|CDS2	1
12	4	4	15.56999981	6.7979999	6.797999889	TR23903|c0_g4_i2|CDS2	2
12	0	4	15.73999971	6.8740003	6.874000281	TR23903|c0_g4_i1|CDS3	2
12	0	4	17.31999964	7.5609997	7.560999691	TR14264|c0_g1_i1|CDS1	2
12	0	2	9.230999649	5.7689998	5.76899983	TR23903|c0_g4_i3|CDS2	1
13	4	4	50	39.739999	39.73999918	TR15501|c0_g2_i1|CDS1	2
13	0	4	50	39.739999	39.73999918	TR14500|c0_g1_i1|CDS1	2
13	0	2	41.02999866	20.51	20.50999999	TR15501|c0_g3_i1|CDS1	1
13	0	2	41.02999866	20.51	20.50999999	TR15501|c0_g1_i1|CDS1	1
13	0	2	41.02999866	20.51	20.50999999	TR14500|c0_g2_i1|CDS1	1
14	2.28	2.28	48.53000045	36.759999	23.53000045	TR16094|c2_g1_i1|CDS1	3
15	2.2	2.2	33.09000134	9.5590003	5.14700003	TR12392|c0_g1_i1|CDS3	1
15	0	2	14.89000022	14.89	14.89000022	TR18411|c0_g1_i2|CDS5	1
15	0	2	14.89000022	14.89	14.89000022	TR18411|c0_g1_i1|CDS5	1

15	0	2	14.89000022	14.89	14.89000022	TR16242|c0_g1_i1|CDS3	1
16	2	39.08	85.6400013	71.01	65.16000032	TR20070|c0_g1_i1|CDS4	23
16	0	39.08	85.6400013	71.01	65.16000032	TR18310|c0_g1_i2|CDS4	23
16	0	39.08	85.6400013	71.01	65.16000032	TR18310|c0_g1_i1|CDS4	23
17	2	16.29	78.68999839	73.769999	68.02999973	TR19632|c0_g2_i1|CDS1	10
17	0	14.25	75.44999719	75.449997	69.09000278	TR19632|c0_g1_i1|CDS1	9
18	2	4.11	39.46999907	22.37	14.47000057	TR17276|c0_g1_i1|CDS2	2
19	2	2	46.81000113	21.28	21.27999961	TR7556|c0_g1_i1|CDS2	1
19	0	2	21.43000066	10.2	10.19999981	TR7556|c0_g2_i1|CDS1	1
19	0	2	2.343999967	1.116	1.116000023	TR15597|c0_g1_i1|CDS3	1
20	2	2	44.92999911	33.329999	33.32999945	TR16094|c2_g3_i1|CDS1	1
21	2	2	34.77999866	23.190001	23.19000065	TR16094|c2_g2_i1|CDS1	1
21	0	2	34.77999866	23.190001	23.19000065	TR16094|c1_g2_i2|CDS2	1
21	0	2	34.77999866	23.190001	23.19000065	TR16094|c1_g2_i1|CDS2	1
22	2	2	4.216999933	2.7109999	2.710999921	TR21606|c0_g1_i1|CDS1	1
23	2	2	23.28999937	23.289999	23.28999937	TR18971|c0_g8_i1|CDS2	1
23	0	2	23.28999937	23.289999	23.28999937	TR18971|c0_g6_i1|CDS3	1
23	0	2	22.37000018	22.37	22.37000018	TR18971|c0_g4_i1|CDS1	1
23	0	2	23.28999937	23.289999	23.28999937	TR18971|c0_g3_i1|CDS2	1
23	0	2	22.37000018	22.37	22.37000018	TR18971|c0_g2_i1|CDS1	1
23	0	2	22.37000018	22.37	22.37000018	TR18971|c0_g15_i1|CDS1	1
23	0	2	23.28999937	23.289999	23.28999937	TR18971|c0_g14_i1|CDS3	1
23	0	2	22.37000018	22.37	22.37000018	TR18971|c0_g13_i1|CDS1	1
23	0	2	23.28999937	23.289999	23.28999937	TR18971|c0_g12_i1|CDS3	1
23	0	2	22.37000018	22.37	22.37000018	TR18971|c0_g11_i1|CDS1	1
23	0	2	21.78999931	21.789999	21.78999931	TR16575|c0_g1_i1|CDS3	1

TABLE S5

Protein summary of Protein Pilot search of rpHPLC fraction 3 (Figure 2)

N	Unused	Total	%Cov	%Cov(50)	%Cov(95)	Accession	Peptides(95%)
1	95.16	95.16	83.19000006	83.19	83.19000006	TR15540|c0_g1_i1|CDS3	126
2	60.56	60.56	83.56000185	83.560002	83.56000185	TR19670|c0_g1_i1|CDS1	67
3	22	22	62.04000115	62.040001	61.1100018	TR17133|c3_g1_i1|CDS1	15
4	14	14	63.09999824	63.099998	63.09999824	TR18770|c2_g2_i1|CDS1	9
4	0	14	54.64000106	54.640001	54.64000106	TR18770|c2_g11_i1|CDS1	9
5	10.95	12	41.58000052	41.580001	41.58000052	TR18770|c0_g1_i1|CDS1	9
5	0	10	51.46999955	51.47	51.46999955	TR18770|c2_g10_i1|CDS1	7
6	10.06	10.06	61.54000163	51.279998	38.46000135	TR11265|c0_g1_i1|CDS1	5
7	10	29.67	84.2599988	84.259999	84.2599988	TR19010|c0_g2_i1|CDS1	21
8	10	10	46.88000083	46.880001	46.88000083	TR14597|c0_g1_i1|CDS3	7
9	6	15.55	80.33000231	80.330002	80.33000231	TR30849|c0_g1_i1|CDS2	11
10	6	8	51.95000172	51.950002	51.95000172	TR20957|c0_g1_i1|CDS2	4
10	0	8	51.95000172	51.950002	51.95000172	TR19470|c2_g3_i1|CDS2	4
11	6	6	35.44999957	28.18	28.18000019	TR10789|c0_g2_i1|CDS2	3
12	5.32	5.32	20.46999931	20.469999	20.46999931	TR697|c0_g1_i1|CDS5	4
13	4	4	24.32000041	12.16	12.16000021	TR23597|c1_g3_i3|CDS3	2
13	0	4	14.05999959	14.06	14.05999959	TR23597|c1_g3_i8|CDS1	2
13	0	4	13.1400004	13.14	13.1400004	TR23597|c1_g3_i6|CDS1	2
13	0	4	21.43000066	21.430001	21.43000066	TR23597|c1_g3_i1|CDS1	2
14	4	4	42.86000133	38.960001	38.96000087	TR13891|c0_g1_i1|CDS2	2
15	4	4	28.36999893	28.369999	28.36999893	TR7596|c0_g2_i1|CDS2	2
15	0	4	28.36999893	28.369999	28.36999893	TR7596|c0_g1_i1|CDS2	2
16	4	4	28.56999934	28.569999	28.56999934	TR4154|c0_g2_i1|CDS2	2
16	0	4	28.56999934	28.569999	28.56999934	TR4154|c0_g1_i1|CDS2	2
17	3.41	3.41	19.23000067	19.230001	19.23000067	TR15501|c0_g3_i1|CDS1	2
17	0	3.41	19.23000067	19.230001	19.23000067	TR15501|c0_g1_i1|CDS1	2
17	0	3.41	19.23000067	19.230001	19.23000067	TR14500|c0_g2_i1|CDS1	2
17	0	2	10.26000008	10.26	10.26000008	TR15501|c0_g2_i1|CDS1	1
17	0	2	10.26000008	10.26	10.26000008	TR14500|c0_g1_i1|CDS1	1
18	2.32	2.32	18.17999929	18.179999	18.17999929	TR21139|c0_g1_i1|CDS1	2
18	0	2	6.357999891	6.3579999	6.357999891	TR21139|c1_g1_i1|CDS3	1
19	2	20.09	75	75	75	TR19010|c0_g1_i1|CDS1	16
20	2	16	69.23000216	69.230002	67.94999838	TR17133|c1_g1_i1|CDS1	12
21	2	4	39.73999918	23.08	23.08000028	TR3944|c0_g1_i1|CDS2	2
21	0	4	46.97000086	27.270001	27.27000117	TR10447|c0_g3_i1|CDS2	2
21	0	4	39.73999918	23.08	23.08000028	TR10447|c0_g2_i1|CDS2	2
21	0	4	39.73999918	23.08	23.08000028	TR10447|c0_g1_i1|CDS2	2
22	2	2	14.40999955	14.41	14.40999955	TR991|c0_g1_i1|CDS1	1
23	2	2	18.45999956	18.46	18.45999956	TR20670|c1_g6_i4|CDS2	1
23	0	2	15.99999964	16	15.99999964	TR20670|c1_g6_i3|CDS2	1
24	2	2	15.24000019	15.24	15.24000019	TR16190|c0_g1_i1|CDS3	1

TABLE S6

Protein summary of Protein Pilot search of rpHPLC fraction 4 (Figure 2)

N	Unused	Total	%Cov	%Cov(50)	%Cov(95)	Accession	Peptides(95%)
1	49.57	49.57	69.73999739	69.739997	69.73999739	TR14746|c0_g1_i1|CDS1	57
2	35.44	35.44	49.63999987	47.029999	43.23000014	TR24122|c1_g2_i3|CDS2	34
3	32.29	32.55	75	67.189997	64.06000257	TR24122|c1_g2_i2|CDS2	33
4	29.75	29.75	57.66000152	57.660002	57.66000152	TR22931|c0_g3_i1|CDS1	33
5	29.04	29.04	81.59999847	53.299999	53.29999924	TR18613|c0_g1_i1|CDS1	18
6	20.07	20.07	69.99999881	69.999999	69.99999881	TR20861|c1_g1_i1|CDS4	20
7	19.27	29.05	66.36999846	66.369998	66.36999846	TR24122|c1_g2_i6|CDS3	26
7	0	29.05	66.36999846	66.369998	66.36999846	TR24122|c1_g2_i1|CDS2	26
8	12.07	12.07	39.12999928	35.65	35.64999998	TR18660|c1_g1_i1|CDS1	6
9	10.91	10.91	74.150002	59.179997	59.17999744	TR20889|c1_g1_i1|CDS1	9
9	0	8.85	60.83999872	45.449999	45.44999897	TR20889|c1_g1_i2|CDS1	8
10	10.04	10.04	49.32000041	35.139999	35.13999879	TR23597|c1_g3_i3|CDS3	5
10	0	10	60.5799973	37.959999	37.95999885	TR23597|c1_g3_i6|CDS1	5
10	0	10	40.63000083	40.630001	40.63000083	TR23597|c1_g3_i8|CDS1	5
10	0	10	61.90000176	61.900002	61.90000176	TR23597|c1_g3_i1|CDS1	5
11	7.98	7.98	40.200001	40.200001	33.32999945	TR24569|c0_g27_i1|CDS2	4
11	0	5.54	28.74000072	28.740001	20.69000006	TR24569|c0_g27_i2|CDS2	3
12	7.17	7.17	44.85999942	44.859999	44.85999942	TR23597|c1_g8_i1|CDS1	5
12	0	9.19	56.87999725	56.879997	46.7900008	TR24670|c2_g29_i1|CDS2	6
12	0	7.17	59.21000242	59.210002	59.21000242	TR23597|c1_g9_i1|CDS1	5
12	0	7.17	49.45000112	49.450001	49.45000112	TR23597|c1_g8_i2|CDS1	5
12	0	7.17	64.85999823	64.859998	64.85999823	TR23597|c1_g6_i1|CDS1	5
12	0	7.17	44.85999942	44.859999	44.85999942	TR23597|c1_g4_i1|CDS1	5
12	0	5.03	51.52000189	51.520002	51.52000189	TR24670|c2_g30_i1|CDS4	4
12	0	5.03	45.32999992	45.33	45.32999992	TR24670|c2_g24_i1|CDS4	4
12	0	5.03	50	50	50	TR23597|c1_g7_i1|CDS1	4
12	0	5.03	28.0400008	28.040001	28.0400008	TR23597|c1_g5_i1|CDS1	4
13	6.03	6.03	51.34999752	39.64	39.64000046	TR991|c0_g1_i1|CDS1	9
14	6	32.55	71.88000083	64.060003	60.93999743	TR24122|c1_g2_i5|CDS1	28
15	4.16	4.16	28.8500011	24.04	24.04000014	TR18837|c0_g5_i1|CDS2	3
15	0	4.16	48.39000106	40.32	40.32000005	TR18837|c0_g3_i1|CDS2	3
16	4	4	32.64999986	24.49	24.49000031	TR10278|c0_g1_i1|CDS1	2
16	0	4	25.70999861	25.709999	25.70999861	TR24676|c1_g1_i3|CDS1	2
16	0	4	24.49000031	24.49	24.49000031	TR10278|c0_g3_i1|CDS1	2
16	0	2	12.42000014	9.1499999	9.149999917	TR24676|c1_g4_i1|CDS1	1
16	0	2	25.74000061	13.860001	13.86000067	TR24676|c1_g1_i2|CDS2	1
16	0	2	25.70999861	25.709999	13.33000064	TR24676|c1_g12_i1|CDS1	1
16	0	2	36.07000113	36.070001	36.07000113	TR24676|c1_g9_i1|CDS3	1
16	0	2	12.72999942	12.729999	12.72999942	TR24676|c1_g1_i4|CDS1	1
16	0	2	13.33000064	13.330001	13.33000064	TR24676|c1_g16_i1|CDS1	1
16	0	2	36.07000113	36.070001	36.07000113	TR24676|c1_g14_i1|CDS1	1

16	0	2	23.65999967	23.66	23.65999967	TR10278|c0_g2_i1|CDS1	1
17	3.5	3.5	18.0399999	10.59	10.58999971	TR17674|c0_g1_i1|CDS2	2
17	0	1.32	32.53000081	9.6390001	9.639000148	TR28572|c0_g1_i1|CDS1	1
18	3.42	3.42	28.15000117	17.039999	17.03999937	TR19075|c0_g1_i2|CDS2	2
18	0	3.42	21.24000043	20.35	20.35000026	TR17377|c0_g4_i1|CDS2	2
18	0	3.42	22.85999954	21.9	21.89999968	TR17377|c0_g3_i1|CDS2	2
18	0	3.42	22.85999954	21.9	21.89999968	TR17377|c0_g1_i1|CDS2	2
18	0	2.01	53.57000232	26.789999	26.78999901	TR17377|c0_g7_i1|CDS1	1
19	3.16	3.16	35.58999896	24.58	15.25000036	TR17681|c0_g2_i1|CDS2	2
19	0	3.16	35.58999896	24.58	15.25000036	TR17681|c0_g1_i1|CDS2	2
19	0	3.16	24.58000034	24.58	15.25000036	TR17681|c0_g3_i1|CDS2	2
20	2.01	2.5	20.76999992	7.7289999	3.62300016	TR24398|c1_g1_i7|CDS1	1
20	0	2.49	15.28999954	9.7860001	4.586999863	TR24398|c1_g1_i1|CDS1	1
20	0	2.06	16.91000015	5.0719999	3.62300016	TR24398|c1_g1_i4|CDS1	1
20	0	2.06	12.74999976	6.8630002	4.90199998	TR24398|c1_g1_i6|CDS1	1
21	2.01	2.01	9.578999877	3.7379999	3.737999871	TR20169|c0_g1_i1|CDS1	1
21	0	2	30.86000085	19.750001	19.75000054	TR6278|c0_g1_i1|CDS2	1
22	2.01	2.01	16.83000028	9.9009998	9.900999814	TR18770|c0_g1_i1|CDS1	1
22	0	2	11.90000027	11.9	11.90000027	TR18770|c2_g9_i1|CDS1	1
22	0	2	12.65999973	12.66	12.65999973	TR18770|c2_g7_i1|CDS1	1
22	0	2	14.71000016	14.71	14.71000016	TR18770|c2_g10_i1|CDS1	1
23	2	6.05	40.32000005	33.87	33.86999965	TR18660|c0_g1_i1|CDS1	3
24	2	4.7	27.88999975	27.89	27.88999975	TR20889|c1_g2_i2|CDS2	4
24	0	4.7	27.88999975	27.89	27.88999975	TR20889|c1_g2_i1|CDS1	4
25	2	4.16	28.8500011	20.190001	20.19000053	TR18837|c0_g4_i1|CDS2	3
26	2	4.01	28.8500011	24.04	24.04000014	TR18837|c0_g2_i1|CDS2	2
26	0	4.01	41.10000134	34.25	34.25000012	TR11773|c0_g1_i1|CDS2	2
26	0	2.01	19.23000067	10.58	10.58000028	TR18837|c0_g6_i1|CDS2	1
27	2	4	38.02999854	25.350001	25.35000145	TR24670|c2_g1_i1|CDS2	2
28	2	4	22.85999954	22.86	22.85999954	TR24676|c1_g1_i1|CDS2	2
28	0	2	13.33000064	13.330001	13.33000064	TR24676|c1_g18_i1|CDS2	1
29	2	2	20.58999985	15.69	15.69000036	TR24676|c1_g19_i1|CDS1	1
29	0	2	17.98000038	17.98	17.98000038	TR24676|c0_g1_i1|CDS1	1
30	2	2	8.474999666	8.4749997	8.474999666	TR5441|c0_g2_i1|CDS1	1
30	0	2	9.615000337	9.6150003	9.615000337	TR19970|c0_g9_i1|CDS1	1
30	0	2	17.54000038	17.54	17.54000038	TR19970|c0_g8_i1|CDS1	1
30	0	2	17.54000038	17.54	17.54000038	TR19970|c0_g7_i1|CDS1	1
30	0	2	8.92900005	8.9290001	8.92900005	TR19970|c0_g5_i1|CDS1	1
30	0	2	17.54000038	17.54	17.54000038	TR19970|c0_g4_i1|CDS1	1
30	0	2	17.54000038	17.54	17.54000038	TR19970|c0_g3_i1|CDS1	1
30	0	2	9.615000337	9.6150003	9.615000337	TR19970|c0_g1_i2|CDS1	1
31	2	2	5.792000145	5.7920001	5.792000145	TR23770|c4_g1_i1|CDS2	1
31	0	2	6.09799996	6.098	6.09799996	TR23770|c3_g1_i1|CDS1	1
32	2	2	1.62499994	1.6249999	1.62499994	TR22055|c0_g1_i4|CDS1	1
32	0	2	1.862999983	1.863	1.862999983	TR22055|c0_g1_i3|CDS1	1

32	0	2	1.664000005	1.664	1.664000005	TR22055|c0_g1_i2|CDS1	1
32	0	2	2.243999951	2.244	2.243999951	TR22055|c0_g1_i1|CDS1	1
33	2	2	23.28999937	23.289999	23.28999937	TR18971|c0_g8_i1|CDS2	1
33	0	2	23.28999937	23.289999	23.28999937	TR18971|c0_g6_i1|CDS3	1
33	0	2	22.37000018	22.37	22.37000018	TR18971|c0_g4_i1|CDS1	1
33	0	2	23.28999937	23.289999	23.28999937	TR18971|c0_g3_i1|CDS2	1
33	0	2	22.37000018	22.37	22.37000018	TR18971|c0_g2_i1|CDS1	1
33	0	2	22.37000018	22.37	22.37000018	TR18971|c0_g15_i1|CDS1	1
33	0	2	23.28999937	23.289999	23.28999937	TR18971|c0_g14_i1|CDS3	1
33	0	2	22.37000018	22.37	22.37000018	TR18971|c0_g13_i1|CDS1	1
33	0	2	23.28999937	23.289999	23.28999937	TR18971|c0_g12_i1|CDS3	1
33	0	2	22.37000018	22.37	22.37000018	TR18971|c0_g11_i1|CDS1	1
33	0	2	21.78999931	21.789999	21.78999931	TR16575|c0_g1_i1|CDS3	1
34	2	2	15.79000056	15.790001	15.79000056	TR16426|c0_g2_i1|CDS2	1
34	0	2	15.79000056	15.790001	15.79000056	TR16426|c0_g1_i1|CDS2	1

TABLE S7

Protein summary of Protein Pilot search of rpHPLC fraction 5 (Figure 2)

N	Unused	Total	%Cov	%Cov(50)	%Cov(95)	Accession	Peptides(95%)
1	50.25	50.25	69.91000175	62.5	59.2599988	TR15609|c0_g1_i1|CDS1	31
2	49.7	49.7	79.14999723	71.429998	71.42999768	TR23770|c4_g1_i1|CDS2	56
2	0	49.7	83.32999945	75.199997	75.19999743	TR23770|c3_g1_i1|CDS1	56
3	48.78	48.78	62.15000153	60.509998	57.01000094	TR20169|c0_g1_i1|CDS1	53
4	47.21	47.21	75.44000149	71.890002	70.10999918	TR23770|c4_g2_i1|CDS2	41
5	46.4	48.21	69.98000145	58.600003	52.06000209	TR22258|c0_g1_i1|CDS1	34
6	33.21	33.21	83.63999724	83.179998	80.90999722	TR18707|c0_g1_i1|CDS1	27
7	31.33	31.33	76.34999752	76.349998	74.32000041	TR23597|c1_g3_i3|CDS3	50
8	25.34	25.34	47.54000008	47.54	44.58999932	TR24122|c1_g2_i4|CDS2	19
9	22.47	22.47	66.36000276	62.620002	62.62000203	TR23597|c1_g8_i1|CDS1	37
9	0	22.47	66.36000276	62.620002	62.62000203	TR23597|c1_g4_i1|CDS1	37
9	0	22.47	90.53999782	90.539998	90.53999782	TR23597|c1_g6_i1|CDS1	37
10	20.73	20.73	51.12000108	49.380001	44.67000067	TR23756|c1_g1_i1|CDS2	14
11	17.87	17.87	31.06000125	22.040001	20.4400003	TR18551|c0_g1_i1|CDS2	11
12	16.93	16.93	79.40999866	73.53	72.54999876	TR20046|c0_g1_i3|CDS1	16
13	16.46	18.57	85.32000184	85.320002	85.32000184	TR24670|c2_g4_i1|CDS1	26
14	16.26	16.26	70.0699985	59.859997	58.49999785	TR10278|c0_g3_i1|CDS1	10
14	0	14.78	70.0699985	51.700002	50.34000278	TR10278|c0_g1_i1|CDS1	9
15	14.36	14.36	51.02999806	50	46.38999999	TR21070|c0_g4_i1|CDS2	11
15	0	14.36	51.02999806	50	46.38999999	TR20772|c0_g2_i1|CDS1	11
16	12.8	12.8	21.78999931	21.789999	21.78999931	TR23240|c0_g1_i2|CDS2	20
17	12.47	12.47	51.37000084	40.439999	37.70000041	TR16224|c0_g1_i1|CDS1	9
18	9.89	9.89	43.23999882	43.239999	43.23999882	TR17389|c0_g1_i1|CDS1	5
19	8.42	8.51	31.88000023	20.53	16.42999947	TR24398|c1_g1_i8|CDS2	5
19	0	8.51	30.68000078	20.53	16.42999947	TR24398|c1_g1_i4|CDS1	5
19	0	6.12	25.11999905	14.98	10.86999997	TR24398|c1_g1_i5|CDS1	4
20	8	8	33.57999921	33.579999	33.57999921	TR24676|c1_g15_i2|CDS1	4
21	7.82	7.82	42.73000062	42.730001	42.73000062	TR10789|c0_g2_i1|CDS2	4
22	6.93	9.67	44.49999928	39.910001	32.57000148	TR18693|c0_g1_i1|CDS1	6
23	6.91	12.51	84.50999856	84.509999	84.50999856	TR24670|c2_g1_i1|CDS2	34
24	6.5	20.17	70.30000091	66.339999	66.33999944	TR24670|c2_g11_i1|CDS4	32
25	6.05	8.06	68.84999871	68.849999	68.84999871	TR24670|c2_g27_i1|CDS1	12
25	0	6.05	78.68999839	31.150001	31.15000129	TR24670|c2_g26_i1|CDS2	8
26	6	11.06	87.99999952	73.329997	73.32999706	TR24861|c1_g23_i1|CDS3	14
26	0	8.98	67.69000292	67.690003	67.69000292	TR24861|c1_g26_i1|CDS3	11
27	6	6	30.30000031	30.3	30.30000031	TR13276|c0_g1_i2|CDS2	3
27	0	3.82	8.296000212	4.1480001	4.148000106	TR22904|c0_g1_i1|CDS3	2
27	0	3.82	18.79999936	18.799999	18.79999936	TR13276|c0_g1_i1|CDS3	2
28	5.26	5.26	18.01999956	12.79	12.79000044	TR20170|c1_g1_i1|CDS1	3
28	0	4	46.23999894	33.329999	33.32999945	TR16730|c0_g1_i1|CDS4	2
29	5.19	5.19	18.75	18.75	18.75	TR24403|c1_g2_i2|CDS1	3

29	0	3.14	38.98000121	38.980001	38.98000121	TR24403|c1_g3_i1|CDS1	2
30	4.17	13.2	23.7499997	19.71	17.57999957	TR24122|c1_g2_i3|CDS2	8
31	4.04	28.99	90.82999825	87.159997	87.15999722	TR24670|c2_g29_i1|CDS2	48
32	4.03	4.06	15.64999968	6.8460003	4.645000026	TR22816|c0_g1_i1|CDS1	2
33	4	25.57	71.2199986	71.219999	67.62999892	TR23770|c4_g2_i2|CDS1	30
33	0	9.84	45.78000009	45.78	39.75999951	TR23770|c2_g1_i1|CDS4	9
34	4	22.47	66.36000276	62.620002	62.62000203	TR23597|c1_g5_i1|CDS1	35
34	0	17.07	81.81999922	81.819999	75.31999946	TR23597|c1_g8_i4|CDS1	30
34	0	16.95	63.63999844	57.58	57.58000016	TR23597|c1_g1_i1|CDS1	25
35	3.96	4	25.56999922	25.569999	18.32000017	TR30852|c0_g1_i1|CDS1	2
36	3.49	3.49	14.47000057	11.84	11.84	TR14746|c0_g1_i1|CDS1	2
36	0	3.49	23.08000028	18.880001	18.88000071	TR12357|c0_g1_i1|CDS1	2
36	0	3.47	19.2900002	19.29	19.2900002	TR12357|c0_g2_i1|CDS1	2
37	3.35	7.9	61.43000126	24.29	22.85999954	TR24676|c1_g1_i1|CDS2	4
37	0	3.35	61.33000255	13.330001	13.33000064	TR24676|c1_g18_i1|CDS2	2
38	3.28	10.59	44.44000125	33.989999	33.9899987	TR24676|c1_g4_i1|CDS1	7
38	0	8.55	38.10000122	38.100001	38.10000122	TR24676|c1_g16_i1|CDS1	6
38	0	8.55	38.10000122	38.100001	38.10000122	TR24676|c1_g12_i1|CDS1	6
38	0	6.29	41.58000052	25.740001	25.74000061	TR24676|c1_g2_i1|CDS1	4
38	0	3.28	30.00000119	30.000001	30.00000119	TR24676|c1_g8_i1|CDS2	2
38	0	3.28	30.00000119	30.000001	30.00000119	TR24676|c1_g17_i1|CDS2	2
39	2.91	5.41	17.52000004	11.92	9.976000339	TR23970|c2_g1_i1|CDS1	4
39	0	2	10.70000008	8.021	8.021000028	TR23970|c2_g2_i1|CDS2	1
40	2.44	10.76	60.00000238	56.43	47.13999927	TR24676|c1_g1_i3|CDS1	7
41	2.32	2.32	23.96000028	17.19	7.812999934	TR24122|c1_g2_i2|CDS2	1
42	2.16	7.62	28.99000049	19.320001	15.45999944	TR24398|c1_g1_i3|CDS1	4
42	0	5.16	28.09999883	16.670001	13.40000033	TR24398|c1_g1_i6|CDS1	3
42	0	3.01	20.76999992	11.11	7.24600032	TR24398|c1_g1_i2|CDS1	2
43	2.03	6.05	40.40000141	25.830001	25.83000064	TR24676|c1_g20_i2|CDS1	4
43	0	4	31.11000061	31.110001	31.11000061	TR24676|c1_g7_i1|CDS1	3
44	2	14	80.9499979	80.949998	80.9499979	TR18707|c1_g1_i1|CDS1	12
45	2	10.47	92.93000102	90.91	90.90999961	TR24670|c2_g19_i1|CDS2	18
46	2	6.05	70.59000134	27.939999	27.9399991	TR24670|c2_g28_i1|CDS2	8
46	0	6.05	78.68999839	31.150001	31.15000129	TR24670|c2_g26_i1|CDS2	8
46	0	2.98	30.88000119	27.939999	27.9399991	TR24670|c2_g9_i1|CDS2	5
46	0	2.98	30.88000119	27.939999	27.9399991	TR24670|c2_g3_i1|CDS2	5
47	2	4	43.1400001	27.450001	27.45000124	TR24676|c1_g19_i1|CDS1	2
47	0	4	31.45999908	31.459999	31.45999908	TR24676|c0_g1_i1|CDS1	2
48	2	2.03	26.55999959	17.19	17.19000041	TR24676|c1_g10_i1|CDS1	1

TABLE S8

Protein summary of Protein Pilot search of rpHPLC fraction 6 (Figure 2)

N	Unused	Total	%Cov	%Cov(50)	%Cov(95)	Accession	Peptides(95%)
1	55.29	55.29	71.78000212	67.640001	67.64000058	TR23970|c2_g1_i1|CDS1	43
2	47.1	47.1	71.06000185	59.030002	57.41000175	TR15609|c0_g1_i1|CDS1	32
3	44.17	44.17	68.9800024	67.739999	64.27000165	TR23756|c1_g1_i1|CDS2	29
4	38.07	38.07	60.76999903	56.900001	43.83000135	TR23612|c0_g1_i2|CDS1	30
5	37.84	37.84	85.90999842	83.639997	80.90999722	TR18707|c0_g1_i1|CDS1	29
6	37.13	37.13	66.40999913	66.409999	66.40999913	TR23770|c4_g1_i1|CDS2	24
6	0	37.13	69.91999745	69.919997	69.91999745	TR23770|c3_g1_i1|CDS1	24
7	35.03	35.03	96.61999941	96.619999	96.61999941	TR23597|c1_g3_i3|CDS3	53
8	31.03	37.79	87.15999722	82.11	71.56000137	TR18693|c0_g1_i1|CDS1	29
9	30.47	30.47	67.29000211	66.360003	60.75000167	TR23597|c1_g8_i1|CDS1	50
9	0	30.47	67.29000211	66.360003	60.75000167	TR23597|c1_g4_i1|CDS1	50
9	0	30.47	90.53999782	90.539998	87.84000278	TR23597|c1_g6_i1|CDS1	50
10	29.94	29.94	60.00000238	60.000002	57.35999942	TR20969|c0_g1_i1|CDS1	25
11	28.66	28.66	71.36999965	69.800001	64.70999718	TR17674|c0_g1_i1|CDS2	25
12	24.86	24.86	76.16000175	69.749999	69.74999905	TR23770|c4_g2_i1|CDS2	17
13	24.36	25.09	49.75000024	48.53	38.47999871	TR22071|c0_g1_i1|CDS2	12
14	23.73	23.73	94.19000149	81.400001	81.40000105	TR20046|c0_g2_i1|CDS1	23
15	20.79	29.19	92.96000004	88.730001	88.73000145	TR24670|c2_g1_i1|CDS2	65
16	20.53	20.53	29.57000136	22.03	22.03000039	TR23240|c0_g1_i4|CDS2	29
16	0	20.53	29.64999974	22.09	22.08999991	TR23240|c0_g1_i3|CDS2	29
17	16.11	31.2	85.0300014	85.030001	85.0300014	TR23970|c2_g2_i1|CDS2	26
18	14.61	18.13	42.0599997	33.180001	29.44000065	TR20169|c0_g1_i1|CDS1	10
19	12	12	37.16000021	37.16	37.16000021	TR22217|c0_g1_i2|CDS1	7
19	0	12	37.16000021	37.16	37.16000021	TR22217|c0_g1_i1|CDS1	7
20	10.13	10.13	48.08999896	34.43	34.43000019	TR16224|c0_g1_i1|CDS1	6
21	10	10	45.57999969	38.100001	38.10000122	TR10278|c0_g3_i1|CDS1	5
21	0	10	45.57999969	38.100001	38.10000122	TR10278|c0_g1_i1|CDS1	5
21	0	8	45.15999854	45.159999	45.15999854	TR10278|c0_g2_i1|CDS1	4
22	9.89	9.89	42.26999879	36.08	36.0799998	TR21070|c0_g4_i1|CDS2	6
22	0	9.89	42.26999879	36.08	36.0799998	TR20772|c0_g2_i1|CDS1	6
23	9.08	9.08	20.29999942	20.299999	20.29999942	TR22181|c0_g1_i1|CDS2	5
24	8.08	17.93	95.34999728	76.740003	76.74000263	TR24861|c1_g18_i1|CDS1	10
25	8	33.18	100	100	100	TR23597|c1_g3_i7|CDS3	61
26	8	8	16.8599993	16.859999	16.8599993	TR21936|c0_g1_i1|CDS4	4
27	7.72	12.19	75.3099978	67.900002	67.900002	TR24861|c1_g3_i1|CDS3	19
28	7.33	7.33	17.33999997	13.54	13.53999972	TR24122|c1_g2_i3|CDS2	4
29	7.15	8.21	24.4599998	16.46	14.04000074	TR22258|c0_g1_i1|CDS1	5
30	6.28	6.28	12.1299997	12.13	10.62999964	TR24241|c0_g1_i1|CDS3	3
31	6.06	37.15	56.90000057	54.24	46.2500006	TR23612|c0_g1_i1|CDS1	31
32	6	30.91	93.58000159	89.910001	84.39999819	TR24670|c2_g29_i1|CDS2	45
33	6	6	22.16999978	11.55	11.55000031	TR21205|c0_g2_i1|CDS2	3

34	6	6	24.56	18.86	18.86000037	TR14746|c0_g1_i1|CDS1	3
34	0	4	25	15.710001	15.71000069	TR12357|c0_g2_i1|CDS1	2
34	0	4	24.48000014	15.38	15.37999958	TR12357|c0_g1_i1|CDS1	2
35	6	6	19.26999986	19.27	19.26999986	TR23050|c0_g2_i1|CDS1	3
36	5.49	19.04	79.75999713	79.759997	79.75999713	TR23597|c1_g3_i1|CDS1	27
36	0	17.89	64.3599987	63.370001	43.56000125	TR24670|c2_g11_i1|CDS4	27
37	5.25	7.38	34.63999927	27.450001	27.45000124	TR24676|c1_g4_i1|CDS1	4
37	0	4	34.43999887	34.439999	16.67000055	TR24676|c1_g7_i1|CDS1	2
37	0	4	20.53000033	20.53	9.933999926	TR24676|c1_g20_i2|CDS1	2
37	0	4	14.71000016	14.71	14.71000016	TR24676|c1_g19_i1|CDS1	2
37	0	4	16.85000062	16.850001	16.85000062	TR24676|c0_g1_i1|CDS1	2
38	4.81	4.81	17.46000051	17.460001	17.46000051	TR275|c0_g2_i1|CDS2	3
38	0	2.72	9.836000204	9.8360002	9.836000204	TR275|c0_g1_i1|CDS2	2
39	4.55	4.55	31.11000061	25.929999	25.92999935	TR19643|c0_g1_i2|CDS8	3
39	0	4.55	31.11000061	25.929999	25.92999935	TR19643|c0_g1_i1|CDS3	3
40	4.15	4.15	30.73000014	16.150001	12.5	TR24403|c1_g2_i2|CDS1	2
40	0	2.12	12.06	9.0449996	5.527999997	TR24403|c1_g2_i1|CDS3	1
41	4.1	5.91	38.15999925	38.159999	26.32000148	TR16426|c0_g2_i1|CDS2	3
41	0	5.91	38.15999925	38.159999	26.32000148	TR16426|c0_g1_i1|CDS2	3
42	4.03	4.03	14.98000026	8.9369997	8.936999738	TR24398|c1_g1_i8|CDS2	2
42	0	4.03	17.38999933	8.9369997	8.936999738	TR24398|c1_g1_i4|CDS1	2
43	4	26.86	76.74000263	76.740003	72.67000079	TR1026|c0_g1_i1|CDS1	21
44	4	4.35	85.32000184	57.800001	57.80000091	TR24670|c2_g4_i1|CDS1	3
44	0	2.03	61.76000237	30.880001	30.88000119	TR24670|c2_g4_i2|CDS1	1
45	4	4	8.216000348	6.4130001	6.413000077	TR18551|c0_g1_i1|CDS2	2
46	4	4	16.89999998	12.68	12.68000007	TR16002|c0_g1_i2|CDS3	2
46	0	4	19.67000067	14.749999	14.74999934	TR16002|c0_g1_i1|CDS3	2
47	4	4	19.08999979	19.09	19.08999979	TR10789|c0_g2_i1|CDS2	2
48	3.77	5.39	15.08000046	8.8519998	8.851999789	TR24122|c1_g2_i4|CDS2	3
49	3.74	16.03	70.69000006	48.280001	46.54999971	TR23597|c1_g8_i3|CDS1	26
49	0	20.1	93.40999722	62.639999	60.43999791	TR23597|c1_g8_i2|CDS1	38
49	0	17.89	64.3599987	63.370001	43.56000125	TR24670|c2_g11_i1|CDS4	27
49	0	14	75	51.719999	31.02999926	TR23597|c1_g9_i2|CDS1	25
49	0	14	70.69000006	48.280001	31.02999926	TR23597|c1_g9_i3|CDS1	25
49	0	14	64.46999907	63.160002	60.53000093	TR24670|c2_g14_i1|CDS4	25
49	0	12	56.72000051	55.220002	52.24000216	TR24670|c2_g25_i1|CDS4	19
50	2.35	6.32	77.4600029	77.460003	66.20000005	TR18690|c0_g3_i1|CDS1	7
50	0	4.41	67.61000156	67.610002	56.33999705	TR18690|c0_g6_i1|CDS2	6
50	0	4.41	67.61000156	67.610002	56.33999705	TR18690|c0_g5_i1|CDS1	6
50	0	4.41	67.61000156	67.610002	56.33999705	TR18690|c0_g1_i1|CDS1	6
50	0	4	56.33999705	56.339997	56.33999705	TR18690|c0_g4_i1|CDS1	6
51	2.04	2.04	20.41999996	11.97	6.338000298	TR24569|c0_g6_i1|CDS1	1
51	0	2	15.44000059	6.6179998	6.617999822	TR24569|c0_g7_i1|CDS1	1
51	0	2	18.75	8.0360003	8.036000282	TR24569|c0_g5_i2|CDS1	1
51	0	2	15.44000059	6.6179998	6.617999822	TR24569|c0_g5_i1|CDS1	1

51	0	2	15.44000059	6.6179998	6.617999822	TR24569|c0_g4_i1|CDS1	1
51	0	2	19.53999996	10.34	10.33999994	TR24569|c0_g27_i2|CDS2	1
51	0	2	16.67000055	8.8239998	8.823999763	TR24569|c0_g27_i1|CDS2	1
51	0	2	20.99999934	9.0000004	9.000000358	TR24569|c0_g26_i1|CDS1	1
51	0	2	8.823999763	8.8239998	8.823999763	TR24569|c0_g8_i2|CDS1	1
51	0	2	8.823999763	8.8239998	8.823999763	TR24569|c0_g8_i1|CDS1	1
51	0	2	20.45000046	20.45	20.45000046	TR24569|c0_g2_i1|CDS4	1
51	0	2	13.0400002	13.04	13.0400002	TR24569|c0_g16_i1|CDS2	1
52	2	26.31	94.88999844	94.889998	94.88999844	TR23597|c1_g3_i6|CDS1	37
52	0	14.75	90.90999961	90.91	90.90999961	TR23597|c1_g3_i2|CDS1	19
53	2	25.98	70.09000182	65.420002	59.81000066	TR23597|c1_g5_i1|CDS1	43
53	0	21.84	81.81999922	80.519998	74.02999997	TR23597|c1_g8_i4|CDS1	37
53	0	14	57.58000016	56.059998	53.03000212	TR23597|c1_g1_i1|CDS1	25
54	2	18.67	100	100	100	TR24670|c2_g27_i1|CDS1	21
54	0	4	26.46999955	26.47	26.46999955	TR24670|c2_g28_i1|CDS2	5
54	0	4	29.51000035	29.51	29.51000035	TR24670|c2_g26_i1|CDS2	5
55	2	18.49	26.91999972	21.789999	21.78999931	TR23240|c0_g1_i2|CDS2	21
56	2	18.34	60.14000177	51.450002	51.45000219	TR23970|c2_g2_i2|CDS1	14
56	0	7.35	59.09000039	40.91	40.90999961	TR10196|c0_g1_i1|CDS1	4
57	2	14	71.2199986	62.589997	62.58999705	TR23770|c4_g2_i2|CDS1	9
57	0	6	37.34999895	31.330001	31.33000135	TR23770|c2_g1_i1|CDS4	4
58	2	8.3	87.99999952	54.67	54.67000008	TR24861|c1_g23_i1|CDS3	12
58	0	5.92	67.69000292	46.149999	46.1499989	TR24861|c1_g26_i1|CDS3	7
59	2	5.8	20.8100006	13.57	13.57000023	TR24861|c1_g19_i4|CDS1	3
59	0	5.8	32.60999918	32.609999	32.60999918	TR24861|c1_g5_i1|CDS2	3
59	0	5.8	32.60999918	32.609999	32.60999918	TR24861|c1_g1_i1|CDS2	3
59	0	5.8	26.55000091	26.550001	26.55000091	TR24861|c1_g19_i5|CDS2	3
59	0	5.8	26.55000091	26.550001	26.55000091	TR24861|c1_g19_i1|CDS2	3
59	0	2	24.59000051	11.48	11.47999987	TR24861|c1_g17_i1|CDS1	1
60	2	5.25	48.57000113	38.100001	38.10000122	TR24676|c1_g12_i1|CDS1	3
61	2	2.04	39.62000012	39.62	24.52999949	TR24569|c0_g9_i1|CDS2	1
61	0	2.04	39.62000012	39.62	24.52999949	TR24569|c0_g24_i1|CDS2	1
61	0	2.04	24.14000034	24.14	14.93999958	TR24569|c0_g12_i2|CDS1	1
61	0	2.04	21.87999934	21.879999	13.53999972	TR24569|c0_g12_i1|CDS1	1
61	0	2.04	19.81000006	19.81	12.25999966	TR24569|c0_g10_i4|CDS2	1
61	0	2.04	20.58999985	20.59	12.74999976	TR24569|c0_g10_i1|CDS2	1
62	2	2	9.290999919	1.956	1.955999993	TR22816|c0_g1_i1|CDS1	1
63	2	2	14.05999959	6.25	6.25	TR24122|c1_g2_i5|CDS1	1

TABLE S9

Protein summary of Protein Pilot search of rpHPLC fraction 7 (Figure 2)

N	Unused	Total	%Cov	%Cov(50)	%Cov(95)	Accession	Peptides(95%)
1	42.45	42.45	71.64000273	64.929998	57.84000158	TR24241|c0_g1_i1|CDS3	26
2	30.65	30.65	53.60000134	52.359998	48.64000082	TR23756|c1_g1_i1|CDS2	16
3	28.8	28.8	66.03999734	61.129999	61.12999916	TR20969|c0_g1_i1|CDS1	22
4	28.73	28.73	35.1000011	32.789999	31.18000031	TR21205|c0_g2_i1|CDS2	24
5	26.31	26.31	50.6099999	46.470001	41.60999954	TR23970|c2_g1_i1|CDS1	16
6	24.69	24.69	65.64000249	62.93	62.15999722	TR23770|c4_g1_i1|CDS2	14
6	0	24.69	69.11000013	66.259998	65.45000076	TR23770|c3_g1_i1|CDS1	14
7	23.54	23.54	41.38999879	39.559999	34.25000012	TR20112|c0_g1_i1|CDS1	11
8	22.27	22.27	82.5600028	82.560003	75.58000088	TR20046|c0_g1_i2|CDS1	35
8	0	19.84	82.5600028	74.419999	67.4399972	TR20046|c0_g1_i1|CDS1	32
9	20.44	20.44	83.49000216	83.490002	82.5699985	TR24670|c2_g29_i1|CDS2	16
10	18.49	18.49	60.65000296	55.599999	48.01000059	TR21550|c0_g1_i1|CDS1	14
11	16.71	18.06	35.78000069	35.780001	34.07000005	TR22071|c0_g1_i1|CDS2	11
12	14.47	14.47	85.92000008	85.92	85.92000008	TR18690|c0_g6_i1|CDS2	23
12	0	14.47	85.92000008	85.92	85.92000008	TR18690|c0_g5_i1|CDS1	23
12	0	14.47	85.92000008	85.92	85.92000008	TR18690|c0_g1_i1|CDS1	23
12	0	13.22	67.61000156	67.610002	67.61000156	TR18690|c0_g3_i1|CDS1	22
13	11.66	11.66	41.15999937	23.190001	20.87000012	TR23240|c0_g1_i4|CDS2	15
13	0	11.66	37.20999956	23.26	20.92999965	TR23240|c0_g1_i3|CDS2	15
14	10.95	10.95	60.21999717	56.989998	46.77000046	TR23419|c0_g6_i1|CDS1	7
15	9.25	15.05	65.53999782	65.539998	60.14000177	TR23597|c1_g3_i3|CDS3	9
16	8.7	8.7	41.85000062	41.850001	38.0400002	TR21550|c0_g2_i1|CDS1	7
17	8.16	8.16	32.03000128	22.059999	19.9300006	TR23770|c4_g2_i1|CDS2	4
18	7.99	7.99	41.53000116	34.43	34.43000019	TR16224|c0_g1_i1|CDS1	5
19	7.81	7.81	34.90000069	31.760001	27.84000039	TR17674|c0_g1_i1|CDS2	6
20	7.42	7.42	19.16999966	19.17	19.16999966	TR20632|c0_g1_i1|CDS1	4
21	7.01	9.85	83.56000185	75.340003	75.34000278	TR23419|c0_g17_i1|CDS1	9
22	5.6	5.6	36.6899997	36.69	36.6899997	TR24693|c0_g25_i4|CDS1	4
22	0	5.38	41.49000049	41.49	41.49000049	TR24693|c0_g25_i2|CDS1	3
22	0	3.29	34.00000036	34	34.00000036	TR24693|c0_g7_i1|CDS1	3
22	0	3.29	34.00000036	34	34.00000036	TR24693|c0_g2_i1|CDS1	3
23	5.49	7.55	71.82999849	71.829998	71.82999849	TR24670|c2_g1_i1|CDS2	11
24	5.18	7.39	68.84999871	68.849999	68.84999871	TR24670|c2_g27_i1|CDS1	5
24	0	7.83	90.31999707	90.319997	67.73999929	TR23597|c1_g3_i7|CDS3	8
24	0	3.49	26.46999955	26.47	26.46999955	TR24670|c2_g28_i1|CDS2	3
24	0	3.49	29.51000035	29.51	29.51000035	TR24670|c2_g26_i1|CDS2	3
25	4	24.38	76.74000263	69.190001	69.19000149	TR1026|c0_g1_i1|CDS1	16
26	4	12.65	47.0600009	47.060001	47.0600009	TR23970|c2_g2_i1|CDS2	9
26	0	6.18	46.66999876	46.669999	46.66999876	TR13242|c0_g1_i1|CDS1	4
27	4	4	29.35000062	29.350001	29.35000062	TR23314|c3_g4_i2|CDS1	2
28	3.8	3.8	68.41999888	45.609999	45.6099987	TR23419|c0_g11_i1|CDS1	3

29	3.58	11.75	59.81000066	57.010001	57.01000094	TR23597|c1_g5_i1|CDS1	9
29	0	10.14	59.81000066	57.010001	57.01000094	TR23597|c1_g8_i1|CDS1	11
29	0	10.14	86.48999929	82.429999	82.42999911	TR23597|c1_g6_i1|CDS1	11
29	0	10.14	59.81000066	57.010001	57.01000094	TR23597|c1_g4_i1|CDS1	11
29	0	8.37	80.51999807	76.620001	70.1300025	TR23597|c1_g8_i4|CDS1	8
30	3.5	5.68	12.03999966	8.3329998	8.332999796	TR15609|c0_g1_i1|CDS1	3
31	3.2	5.82	29.85999882	23.08	19.4600001	TR24861|c1_g19_i4|CDS1	3
31	0	3.6	42.39000082	42.390001	33.70000124	TR24861|c1_g5_i1|CDS2	2
31	0	3.6	42.39000082	42.390001	33.70000124	TR24861|c1_g1_i1|CDS2	2
31	0	3.6	34.50999856	34.509999	27.4300009	TR24861|c1_g19_i5|CDS2	2
31	0	3.6	34.50999856	34.509999	27.4300009	TR24861|c1_g19_i1|CDS2	2
31	0	2.15	26.87999904	26.879999	18.27999949	TR24861|c1_g7_i1|CDS2	1
31	0	2.15	30.12000024	30.12	20.47999948	TR24861|c1_g29_i1|CDS2	1
31	0	2.15	19.68999952	19.69	13.39000016	TR24861|c1_g19_i2|CDS1	1
31	0	2.15	30.86000085	30.860001	20.99000067	TR24861|c1_g16_i1|CDS2	1
31	0	2.15	30.12000024	30.12	20.47999948	TR24861|c1_g14_i1|CDS2	1
31	0	2.15	30.12000024	30.12	20.47999948	TR24861|c1_g10_i1|CDS2	1
32	2.87	2.87	9.818000346	9.8180003	9.818000346	TR17179|c0_g1_i1|CDS3	2
33	2.72	2.72	68.75	68.75	39.05999959	TR7070|c0_g1_i1|CDS1	1
33	0	2.34	57.81000257	57.810003	39.05999959	TR7070|c0_g2_i1|CDS1	1
34	2.54	2.54	27.68000066	19.64	19.64000016	TR24693|c0_g13_i1|CDS1	2
34	0	2.54	30.14000058	30.140001	30.14000058	TR24693|c0_g22_i1|CDS1	2
34	0	1.72	19.40000057	19.400001	19.40000057	TR24693|c0_g8_i1|CDS1	1
34	0	1.72	19.40000057	19.400001	19.40000057	TR24693|c0_g3_i1|CDS1	1
34	0	1.72	10.66000015	10.66	10.66000015	TR24693|c0_g24_i1|CDS2	1
34	0	1.72	22.03000039	22.03	22.03000039	TR24693|c0_g16_i1|CDS1	1
34	0	1.72	19.40000057	19.400001	19.40000057	TR24693|c0_g11_i1|CDS1	1
34	0	1.72	22.03000039	22.03	22.03000039	TR24693|c0_g10_i1|CDS1	1
35	2.47	2.87	11.91999987	10.75	6.075000018	TR20169|c0_g1_i1|CDS1	2
36	2.16	18.27	82.5600028	79.070002	63.95000219	TR20046|c0_g2_i1|CDS1	19
36	0	19.74	80.39000034	70.590001	64.70999718	TR20046|c0_g1_i3|CDS1	23
36	0	17.36	88.8899982	67.900002	67.900002	TR24861|c1_g20_i1|CDS1	19
36	0	6.77	81.4800024	51.849997	44.44000125	TR24861|c1_g9_i2|CDS2	8
36	0	6.77	81.4800024	51.849997	44.44000125	TR24861|c1_g9_i1|CDS3	8
36	0	6.77	81.25	65.630001	56.25	TR24861|c1_g8_i1|CDS2	8
36	0	4.37	81.4800024	51.849997	32.10000098	TR24861|c1_g31_i1|CDS2	5

TABLE S10

Protein summary of Protein Pilot search of rpHPLC fraction 8 (Figure 2)

N	Unused	Total	%Cov	%Cov(50)	%Cov(95)	Accession	Peptides(95%)
1	91.93	91.93	84.70000029	82.65	80.4099977	TR24241|c0_g1_i1|CDS3	74
2	72.69	72.69	40.88000059	40.880001	38.33999932	TR21205|c0_g2_i1|CDS2	73
3	45.79	45.79	59.52000022	54.949999	50.18000007	TR20112|c0_g1_i1|CDS1	28
4	45.38	45.38	74.26000237	67.400002	56.37000203	TR22071|c0_g1_i1|CDS2	29
5	27.43	27.43	83.7199986	79.070002	74.41999912	TR23419|c0_g3_i1|CDS2	22
6	26.41	26.41	70.03999949	55.599999	50.5400002	TR21550|c0_g1_i1|CDS1	15
7	23.92	23.92	46.7200011	41.119999	34.06000137	TR23970|c2_g1_i1|CDS1	15
8	21.55	21.55	83.49000216	83.490002	82.5699985	TR24670|c2_g29_i1|CDS2	18
9	17.96	18.33	86.29999757	78.079998	78.07999849	TR23419|c0_g17_i1|CDS1	14
10	17.22	17.22	45.1700002	39.379999	39.37999904	TR23770|c4_g1_i1|CDS2	10
10	0	17.22	47.56000042	41.460001	41.46000147	TR23770|c3_g1_i1|CDS1	10
11	15.38	15.38	66.18999839	62.589997	62.58999705	TR24693|c0_g25_i4|CDS1	8
12	15.26	15.26	79.07000184	77.91	77.91000009	TR24861|c1_g18_i1|CDS1	14
13	12.57	12.57	28.11999917	17.389999	15.07000029	TR23240|c0_g1_i4|CDS2	9
13	0	12.47	25.29000044	17.44	15.11999965	TR23240|c0_g1_i3|CDS2	9
13	0	11.52	24.68000054	16.670001	16.67000055	TR23240|c0_g1_i2|CDS2	9
14	12.45	12.45	55.62999845	37.32	37.31999993	TR24569|c0_g6_i1|CDS1	8
14	0	12.32	52.20999718	38.97	38.96999955	TR24569|c0_g7_i1|CDS1	8
14	0	12.32	52.20999718	38.97	38.96999955	TR24569|c0_g5_i1|CDS1	8
14	0	12.32	52.20999718	38.97	38.96999955	TR24569|c0_g4_i1|CDS1	8
15	12.41	12.41	38.37000132	31.84	28.15999985	TR23063|c0_g1_i1|CDS1	7
15	0	10.37	53.96000147	47.479999	41.0100013	TR16128|c0_g1_i1|CDS1	6
16	12.02	26.55	63.44000101	60.219997	56.98999763	TR23419|c0_g6_i1|CDS1	21
17	11.06	11.06	23.43000025	17.990001	13.52999955	TR24679|c0_g6_i1|CDS2	6
18	10.43	10.43	64.75999951	59.05	48.57000113	TR24598|c0_g7_i1|CDS1	8
18	0	10.43	64.75999951	59.05	48.57000113	TR24598|c0_g13_i1|CDS1	8
18	0	9.15	65.06000161	55.419999	51.81000233	TR24598|c0_g14_i1|CDS1	6
19	10.35	10.35	27.48000026	27.48	25.56000054	TR20632|c0_g1_i1|CDS1	5
20	9.41	9.41	93.48000288	68.480003	57.60999918	TR23314|c3_g4_i2|CDS1	10
21	8.92	11.06	96.05000019	80.260003	80.26000261	TR24241|c0_g4_i1|CDS2	9
21	0	11.06	96.05000019	80.260003	80.26000261	TR24241|c0_g2_i1|CDS2	9
21	0	10.93	96.05000019	80.260003	80.26000261	TR24241|c0_g3_i1|CDS2	9
21	0	8.82	86.44000292	76.270002	76.27000213	TR24241|c0_g8_i1|CDS1	6
21	0	8.82	86.44000292	76.270002	76.27000213	TR24241|c0_g6_i1|CDS1	6
21	0	8.82	86.44000292	76.270002	76.27000213	TR24241|c0_g5_i1|CDS1	6
21	0	8.82	86.44000292	76.270002	76.27000213	TR24241|c0_g10_i1|CDS1	6
22	8.79	8.79	43.79999936	43.799999	32.85000026	TR24598|c0_g17_i2|CDS1	5
23	8.41	13.06	56.76000118	50.68	44.58999932	TR23597|c1_g3_i3|CDS3	9
24	8.13	8.13	32.78999925	32.789999	28.96000147	TR16224|c0_g1_i1|CDS1	5
24	0	6.1	33.57999921	33.579999	28.47000062	TR7295|c0_g1_i1|CDS1	4
25	8.01	11.69	28.7800014	16.38	16.38000011	TR23756|c1_g1_i1|CDS2	6

26	7.95	12.05	65.64000249	52.149999	52.14999914	TR23419|c0_g15_i1|CDS1	10
27	7.84	7.84	27.3999989	24.56	22.41999954	TR23770|c4_g2_i1|CDS2	4
28	7.72	8.18	88.73000145	88.730001	88.73000145	TR24670|c2_g1_i1|CDS2	5
29	7.6	13.72	75.48999786	67.650002	67.65000224	TR20046|c0_g1_i3|CDS1	8
29	0	10.03	74.07000065	64.200002	64.20000196	TR24861|c1_g20_i1|CDS1	5
30	7.37	7.37	53.57000232	45.539999	38.38999867	TR24693|c0_g13_i1|CDS1	4
31	7.18	7.18	28.70000005	28.7	24.07000065	TR21728|c0_g1_i1|CDS1	4
32	6.67	6.67	51.55000091	37.270001	17.38999933	TR24569|c0_g3_i2|CDS2	3
32	0	6.57	51.55000091	37.270001	17.38999933	TR24569|c0_g3_i1|CDS2	3
32	0	5.84	35.62000096	29.449999	19.17999983	TR24569|c0_g14_i2|CDS1	3
32	0	5.84	36.62	30.28	19.72000003	TR24569|c0_g14_i1|CDS1	3
33	6.1	8.65	45.64999938	45.649999	28.79999876	TR21550|c0_g2_i1|CDS1	5
34	6	14.64	59.81000066	57.010001	57.01000094	TR23597|c1_g5_i1|CDS1	8
34	0	14.64	59.81000066	57.010001	57.01000094	TR23597|c1_g8_i1|CDS1	9
34	0	14.64	86.48999929	82.429999	82.42999911	TR23597|c1_g6_i1|CDS1	9
34	0	14.64	59.81000066	57.010001	57.01000094	TR23597|c1_g4_i1|CDS1	9
34	0	11.73	80.51999807	76.620001	70.1300025	TR23597|c1_g8_i4|CDS1	7
35	6	8.03	68.84999871	68.849999	68.84999871	TR24670|c2_g27_i1|CDS1	7
35	0	8.41	76.34000182	76.340002	67.73999929	TR23597|c1_g3_i7|CDS3	7
35	0	4	26.46999955	26.47	26.46999955	TR24670|c2_g28_i1|CDS2	3
35	0	4	29.51000035	29.51	29.51000035	TR24670|c2_g26_i1|CDS2	3
36	4.73	4.77	58.81999731	46.079999	46.07999921	TR24569|c0_g10_i1|CDS2	3
37	4.52	4.52	60.00000238	53.329998	35.55999994	TR24693|c0_g21_i1|CDS2	3
37	0	4.09	62.30000257	52.460003	52.4600029	TR24693|c0_g20_i1|CDS2	3
37	0	3.75	62.86000013	45.71	30.48000038	TR24693|c0_g4_i1|CDS2	3
37	0	2.52	60.00000238	40.000001	22.22000062	TR24693|c0_g9_i1|CDS2	2
38	4.25	4.25	19.78999972	19.27	13.01999986	TR23050|c0_g2_i1|CDS1	2
39	4	4	18.17999929	18.179999	18.17999929	TR10789|c0_g2_i1|CDS2	2
40	3.82	5.85	67.61000156	67.610002	67.61000156	TR18690|c0_g6_i1|CDS2	5
40	0	5.85	67.61000156	67.610002	67.61000156	TR18690|c0_g5_i1|CDS1	5
40	0	5.85	67.61000156	67.610002	67.61000156	TR18690|c0_g3_i1|CDS1	5
40	0	5.85	67.61000156	67.610002	67.61000156	TR18690|c0_g1_i1|CDS1	5
40	0	4	56.33999705	56.339997	56.33999705	TR18690|c0_g4_i1|CDS1	4
41	3.2	3.2	25.63999891	17.95	17.94999987	TR24679|c0_g4_i1|CDS2	2
42	2.8	2.8	11.86000034	10.41	3.147999942	TR23612|c0_g1_i1|CDS1	1
42	0	2.8	10.40999964	10.41	3.147999942	TR23612|c0_g1_i2|CDS1	1
43	2.67	6.77	53.25000286	53.250003	53.25000286	TR24598|c0_g17_i1|CDS1	5
43	0	4.71	50.78999996	50.79	50.78999996	TR24598|c0_g1_i1|CDS1	4
44	2.51	2.51	7.838000357	6.4130001	6.413000077	TR24122|c1_g2_i3|CDS2	2
44	0	1.77	14.92999941	14.929999	14.92999941	TR35618|c0_g1_i1|CDS1	1
44	0	1.77	4.425000027	4.425	4.425000027	TR24122|c1_g2_i6|CDS3	1
44	0	1.77	4.425000027	4.425	4.425000027	TR24122|c1_g2_i1|CDS2	1
45	2.15	7.76	67.61000156	61.970001	45.77000141	TR24598|c0_g18_i1|CDS2	6
45	0	6.83	26.62999928	20.110001	18.4799999	TR24598|c0_g10_i1|CDS1	4
45	0	5.53	79.79000211	71.280003	58.50999951	TR24598|c0_g4_i1|CDS2	4

45	0	2	32.87999928	16.44	16.43999964	TR24598|c0_g10_i4|CDS1	1
46	2.06	2.58	28.96000147	11.31	7.69200027	TR24861|c1_g19_i4|CDS1	1
46	0	2.58	43.36000085	22.12	15.03999978	TR24861|c1_g19_i5|CDS2	1
46	0	2.57	42.39000082	27.169999	18.4799999	TR24861|c1_g5_i1|CDS2	1
46	0	2.57	42.39000082	27.169999	18.4799999	TR24861|c1_g1_i1|CDS2	1
46	0	2.57	34.50999856	22.12	15.03999978	TR24861|c1_g19_i1|CDS2	1
46	0	2.52	26.87999904	26.879999	18.27999949	TR24861|c1_g7_i1|CDS2	1
46	0	2.52	30.12000024	30.12	20.47999948	TR24861|c1_g29_i1|CDS2	1
46	0	2.52	19.68999952	19.69	13.39000016	TR24861|c1_g19_i2|CDS1	1
46	0	2.52	30.86000085	30.860001	20.99000067	TR24861|c1_g16_i1|CDS2	1
46	0	2.52	30.12000024	30.12	20.47999948	TR24861|c1_g14_i1|CDS2	1
46	0	2.52	30.12000024	30.12	20.47999948	TR24861|c1_g10_i1|CDS2	1
46	0	2.52	32.89000094	32.890001	22.37000018	TR16426|c0_g1_i1|CDS2	1
47	2.05	2.49	62.76999712	36.17	19.14999932	TR24693|c0_g1_i1|CDS2	1
47	0	2.45	57.31999874	41.460001	21.95000052	TR32174|c0_g1_i1|CDS1	1
48	2	20.16	65.09000063	57.4	57.4000001	TR5610|c0_g1_i1|CDS1	14
49	2	12.15	39.03999925	39.039999	39.03999925	TR23970|c2_g2_i1|CDS2	8
49	0	7.28	46.66999876	46.669999	46.66999876	TR13242|c0_g1_i1|CDS1	4
50	2	9.11	44.20000017	44.2	38.40999901	TR23970|c2_g2_i2|CDS1	5
50	0	2.81	28.79000008	28.79	28.79000008	TR10196|c0_g1_i1|CDS1	2
51	2	9.08	69.73999739	69.739997	69.73999739	TR24241|c0_g9_i1|CDS2	7
52	2	8.02	65.52000046	59.77	59.76999998	TR24693|c0_g18_i1|CDS1	4
53	2	7.67	39.57000077	38.13	38.13000023	TR23770|c4_g2_i2|CDS1	4
53	0	3.49	28.92000079	19.28	19.28000003	TR23770|c2_g1_i1|CDS4	2
54	2	7.08	72.14999795	65.820003	65.82000256	TR24693|c0_g17_i1|CDS1	4
54	0	4	33.32999945	27.590001	27.59000063	TR24693|c0_g12_i1|CDS1	2
55	2	6.9	66.67000055	66.670001	66.67000055	TR24861|c1_g3_i1|CDS3	6
56	2	5.77	61.40000224	61.400002	61.40000224	TR23419|c0_g11_i1|CDS1	4
57	2	3.68	42.19999909	33.939999	20.18000036	TR24569|c0_g3_i4|CDS2	2
57	0	3.68	49.4599998	39.78	23.65999967	TR24569|c0_g3_i3|CDS2	2
57	0	2.13	30.00000119	30.000001	18.33000034	TR24569|c0_g28_i1|CDS2	1
58	2	2.37	39.71000016	39.71	39.71000016	TR24670|c2_g9_i1|CDS2	2
58	0	2.37	39.71000016	39.71	39.71000016	TR24670|c2_g3_i1|CDS2	2
59	2	2	6.198000163	4.132	4.131999984	TR23050|c0_g2_i1|CDS7	1
59	0	2	8.620999753	8.6209998	8.620999753	TR3315|c0_g1_i1|CDS2	1
60	2	2	9.301999956	3.4880001	3.48800011	TR20170|c1_g1_i1|CDS1	1
60	0	2	34.40999985	12.899999	12.89999932	TR16730|c0_g1_i1|CDS4	1
61	2	2	16.97999984	16.98	16.97999984	TR24679|c0_g2_i1|CDS3	1
62	2	2	6.16700016	6.1670002	6.16700016	TR18812|c0_g1_i1|CDS4	1

TABLE S11

Protein summary of Protein Pilot search of rpHPLC fraction 9 (Figure 2)

N	Unused	Total	%Cov	%Cov(50)	%Cov(95)	Accession	Peptides(95%)
1	58.82	58.82	76.23000145	73.909998	73.04000258	TR23240|c0_g1_i4|CDS2	85
2	29.53	29.53	62.5	62.5	57.5600028	TR20170|c1_g1_i1|CDS1	18
3	29.32	29.32	83.89999866	78.649998	72.65999913	TR23063|c0_g1_i3|CDS1	20
4	29.11	53.75	95.02000213	89.590001	88.23999763	TR24861|c1_g19_i4|CDS1	79
5	27.11	29.54	87.09999919	87.099999	87.09999919	TR13307|c0_g3_i1|CDS1	60
5	0	29.54	87.09999919	87.099999	87.09999919	TR13307|c0_g2_i1|CDS1	60
6	24.8	24.8	37.40999997	37.41	37.40999997	TR21205|c0_g2_i1|CDS2	15
7	19.47	19.47	53.39999795	39.01	39.01000023	TR15320|c0_g1_i1|CDS3	10
8	19.4	19.4	76.05999708	76.059997	64.07999992	TR24569|c0_g14_i1|CDS1	12
8	0	17.15	64.38000202	64.380002	52.74000168	TR24569|c0_g14_i2|CDS1	11
9	19.33	19.33	31.86999857	31.389999	26.03000104	TR23970|c2_g1_i1|CDS1	9
10	19.22	19.22	56.61000013	44.209999	38.42999935	TR23050|c0_g2_i1|CDS7	9
11	17.99	17.99	80.98000288	72.390002	72.39000201	TR23419|c0_g15_i1|CDS1	18
12	17.14	17.14	75.36000013	62.559998	58.77000093	TR7566|c0_g2_i1|CDS1	12
12	0	16.96	72.50999808	62.559998	58.77000093	TR7566|c0_g1_i1|CDS1	12
13	16.03	16.03	61.97000146	55.629998	55.62999845	TR24569|c0_g6_i1|CDS1	12
13	0	15.96	58.09000134	58.090001	58.09000134	TR24569|c0_g7_i1|CDS1	12
13	0	15.96	58.09000134	58.090001	58.09000134	TR24569|c0_g5_i1|CDS1	12
13	0	15.96	58.09000134	58.090001	58.09000134	TR24569|c0_g4_i1|CDS1	12
14	14.91	14.99	72.82999754	66.299999	66.29999876	TR15592|c0_g1_i1|CDS2	15
15	13.96	13.96	56.83000088	56.830001	56.83000088	TR24693|c0_g25_i4|CDS1	7
16	13.92	19.82	86.14000082	80.199999	80.19999862	TR24861|c1_g24_i2|CDS1	40
17	13.48	13.5	80.21000028	78.130001	78.13000083	TR24569|c0_g12_i1|CDS1	8
17	0	13.5	72.64000177	70.749998	70.74999809	TR24569|c0_g10_i4|CDS2	8
18	12.71	12.71	44.04000044	38.63	32.13	TR21550|c0_g1_i1|CDS1	8
19	12.31	12.31	97.46000171	91.530001	91.53000116	TR5441|c0_g2_i1|CDS1	15
20	12.08	12.08	46.88000083	43.23	43.23000014	TR23050|c0_g2_i1|CDS1	7
21	11.35	23.66	100	89.469999	89.46999907	TR16426|c0_g2_i1|CDS2	47
22	10.41	10.41	39.77000117	35.139999	35.13999879	TR23770|c4_g1_i1|CDS2	6
22	0	10.41	41.87000096	36.989999	36.98999882	TR23770|c3_g1_i1|CDS1	6
23	10.25	10.25	68.62999797	44.119999	38.24000061	TR20046|c0_g1_i3|CDS1	6
23	0	8.23	68.59999895	39.53	32.55999982	TR20046|c0_g2_i1|CDS1	5
24	10	19.78	96.11999989	89.920002	68.9899981	TR24861|c1_g28_i2|CDS1	21
24	0	19.78	76.3800025	70.08	70.08000016	TR24861|c1_g28_i3|CDS2	21
25	9.23	9.23	27.61000097	23.88	20.90000063	TR20089|c0_g1_i1|CDS1	5
26	8.01	8.01	14.2900005	10.99	10.98999977	TR20112|c0_g1_i1|CDS1	4
27	8	21.78	76.3800025	70.08	70.08000016	TR24861|c1_g28_i1|CDS1	16
28	7.8	7.8	75.70999861	75.709999	67.14000106	TR23314|c3_g4_i1|CDS1	8
29	7.44	7.44	29.12999988	29.13	29.12999988	TR23314|c2_g9_i1|CDS1	8
29	0	7.44	26.32000148	26.320001	26.32000148	TR23314|c2_g8_i1|CDS2	8
29	0	7.44	26.32000148	26.320001	26.32000148	TR23314|c2_g6_i1|CDS2	8

29	0	7.44	26.32000148	26.320001	26.32000148	TR23314|c2_g4_i1|CDS2	8
29	0	7.44	26.32000148	26.320001	26.32000148	TR23314|c2_g2_i1|CDS2	8
29	0	6	15.25000036	15.25	15.25000036	TR23314|c2_g7_i1|CDS1	6
29	0	6	15.25000036	15.25	15.25000036	TR23314|c2_g5_i1|CDS1	6
29	0	6	15.25000036	15.25	15.25000036	TR23314|c2_g3_i1|CDS1	6
30	6.95	6.95	67.86000133	60.710001	44.63999867	TR24693|c0_g13_i1|CDS1	3
31	6.82	9.14	24.77000058	17.76	15.88999927	TR20169|c0_g1_i1|CDS1	5
32	6.43	6.43	24.56	22.42	22.41999954	TR23770|c4_g2_i1|CDS2	4
33	6.1	6.1	44.76000071	44.760001	37.13999987	TR24598|c0_g7_i1|CDS1	3
33	0	6.1	44.76000071	44.760001	37.13999987	TR24598|c0_g13_i1|CDS1	3
33	0	6	46.9900012	46.990001	46.9900012	TR24598|c0_g14_i1|CDS1	3
34	6.06	6.06	40.88000059	25.549999	25.54999888	TR24598|c0_g17_i2|CDS1	3
34	0	4.05	46.52999938	25.740001	25.74000061	TR24598|c0_g11_i1|CDS1	2
35	6	12.61	48.9199996	45.699999	38.17000091	TR23419|c0_g6_i1|CDS1	7
35	0	12	75.55999756	75.559998	75.55999756	TR23419|c0_g13_i1|CDS2	8
35	0	10.07	55.04000187	41.859999	41.8599993	TR23419|c0_g3_i1|CDS2	6
35	0	8	53.60999703	53.609997	53.60999703	TR23419|c0_g16_i2|CDS1	5
35	0	8	54.17000055	54.170001	54.17000055	TR23419|c0_g16_i1|CDS1	5
36	5.46	5.46	60.00000238	35.56	35.55999994	TR24693|c0_g9_i1|CDS2	3
36	0	5.46	51.42999887	30.48	30.48000038	TR24693|c0_g4_i1|CDS2	3
36	0	5.46	54.54999804	41.56	41.56000018	TR24693|c0_g6_i1|CDS2	3
36	0	4	52.4600029	36.070001	36.07000113	TR24693|c0_g27_i1|CDS2	2
37	5.23	42.63	76.59999728	74.040002	73.0799973	TR23240|c0_g1_i2|CDS2	66
38	5.14	5.14	37.95999885	21.9	21.89999968	TR23597|c1_g3_i6|CDS1	3
38	0	4.25	43.23999882	28.380001	14.1900003	TR23597|c1_g3_i3|CDS3	2
38	0	4.04	33.59000087	16.410001	16.41000062	TR23597|c1_g3_i8|CDS1	2
38	0	4.04	51.19000077	25	25	TR23597|c1_g3_i1|CDS1	2
39	5.11	5.11	68.66000295	61.189997	61.1899972	TR23314|c3_g1_i1|CDS2	6
40	4.95	4.95	44.15999949	44.159999	44.15999949	TR23597|c1_g8_i4|CDS1	3
40	0	4.95	31.77999854	31.779999	31.77999854	TR23597|c1_g8_i1|CDS1	3
40	0	4.95	45.95000148	45.950001	45.95000148	TR23597|c1_g6_i1|CDS1	3
40	0	4.95	31.77999854	31.779999	31.77999854	TR23597|c1_g5_i1|CDS1	3
40	0	4.95	31.77999854	31.779999	31.77999854	TR23597|c1_g4_i1|CDS1	3
41	4.6	4.6	33.88000131	20.22	20.21999955	TR16224|c0_g1_i1|CDS1	3
41	0	2.87	35.04000008	16.79	16.7899996	TR7295|c0_g1_i1|CDS1	2
42	4.31	11.54	41.17999971	41.18	34.31000113	TR24569|c0_g27_i1|CDS2	7
42	0	9.27	42.53000021	42.53	34.47999954	TR24569|c0_g27_i2|CDS2	6
42	0	7.93	49.30000007	36.62	36.62	TR24569|c0_g1_i1|CDS2	5
42	0	7.93	49.30000007	36.62	36.62	TR24569|c0_g17_i1|CDS2	5
43	4.28	4.28	43.11999977	43.12	36.70000136	TR23529|c0_g1_i1|CDS2	3
43	0	4.28	40.169999	40.169999	34.1899991	TR23529|c0_g13_i1|CDS1	3
44	4.12	6.14	36.9599998	36.96	32.06999898	TR21550|c0_g2_i1|CDS1	3
45	4	7.4	65.11999965	65.12	65.11999965	TR13534|c0_g1_i2|CDS1	6
46	4	6	67.61000156	67.610002	67.61000156	TR18690|c0_g6_i1|CDS2	3
46	0	6	67.61000156	67.610002	67.61000156	TR18690|c0_g5_i1|CDS1	3

46	0	6	67.61000156	67.610002	67.61000156	TR18690|c0_g3_i1|CDS1	3
46	0	6	67.61000156	67.610002	67.61000156	TR18690|c0_g1_i1|CDS1	3
46	0	4	56.33999705	56.339997	56.33999705	TR18690|c0_g4_i1|CDS1	2
47	4	4	8.021999896	5.5969998	5.596999824	TR24241|c0_g1_i1|CDS3	2
48	3.64	14.94	49.07000065	45.339999	40.99000096	TR24569|c0_g3_i1|CDS2	9
48	0	14.1	45.3399986	45.339999	35.40000021	TR24569|c0_g3_i2|CDS2	8
48	0	8.08	75.99999905	75.999999	75.99999905	TR24569|c0_g22_i1|CDS1	5
49	3.03	3.27	66.67000055	66.670001	55.00000119	TR24569|c0_g28_i1|CDS2	2
50	2.69	4.74	47.22000062	47.220001	47.22000062	TR24861|c0_g1_i1|CDS1	7
51	2.19	3.21	79.57000136	64.520001	41.94000065	TR23597|c1_g3_i7|CDS3	2
51	0	2	67.61000156	42.250001	42.25000143	TR24670|c2_g1_i1|CDS2	1
52	2.15	7.91	75.99999905	75.999999	75.99999905	TR24569|c0_g20_i1|CDS1	6
53	2.03	8.91	38.24000061	29.409999	29.40999866	TR24569|c0_g8_i2|CDS1	6
53	0	10.34	51.34999752	51.349998	51.34999752	TR24569|c0_g8_i3|CDS1	7
53	0	8.91	38.24000061	29.409999	29.40999866	TR24569|c0_g8_i1|CDS1	6
54	2.03	3.56	44.8300004	27.590001	27.59000063	TR24693|c0_g12_i1|CDS1	2
54	0	6	55.69999814	55.699998	55.69999814	TR24693|c0_g17_i1|CDS1	3
55	2.01	2.01	6.617999822	3.4309998	3.430999815	TR22071|c0_g1_i1|CDS2	1
56	2	10.23	68.59999895	56.980002	50	TR20046|c0_g1_i2|CDS1	7
56	0	10.23	68.59999895	56.980002	50	TR20046|c0_g1_i1|CDS1	7
56	0	6.19	50	38.370001	31.40000105	TR24861|c1_g18_i1|CDS1	4
56	0	4.15	66.67000055	50.79	41.26999974	TR24861|c1_g15_i1|CDS2	3
57	2	10.06	76.19000077	76.190001	67.86000133	TR24569|c0_g23_i1|CDS1	6
58	2	10.03	75.48999786	66.670001	66.67000055	TR24569|c0_g10_i1|CDS2	6
58	0	7.72	62.88999915	62.889999	62.88999915	TR24569|c0_g11_i1|CDS1	5
58	0	5.68	47.42000103	47.420001	47.42000103	TR24569|c0_g19_i1|CDS1	4
58	0	5.68	61.7299974	61.729997	61.7299974	TR24569|c0_g13_i1|CDS1	4
58	0	2	56.25	56.25	56.25	TR24569|c0_g10_i2|CDS1	1
59	2	8	26.73999965	26.74	26.73999965	TR23970|c2_g2_i1|CDS2	4
59	0	4.2	40.95000029	39.050001	25.70999861	TR13242|c0_g1_i1|CDS1	2
60	2	6.23	34.77999866	33.329999	23.19000065	TR23970|c2_g2_i2|CDS1	3
60	0	2	18.17999929	18.179999	18.17999929	TR10196|c0_g1_i1|CDS1	1
61	2	5.46	60.00000238	35.56	35.55999994	TR24693|c0_g21_i1|CDS2	3
61	0	4	52.4600029	36.070001	36.07000113	TR24693|c0_g20_i1|CDS2	2
62	2	4	73.6800015	61.400002	61.40000224	TR23419|c0_g11_i1|CDS1	4
63	2	4	31.90999925	31.909999	31.90999925	TR24598|c0_g4_i1|CDS2	2
63	0	4	21.13000005	21.13	21.13000005	TR24598|c0_g18_i1|CDS2	2
63	0	4	16.30000025	16.3	16.30000025	TR24598|c0_g10_i1|CDS1	2
63	0	2	16.43999964	16.44	16.43999964	TR24598|c0_g10_i4|CDS1	1
64	2	3.51	32.10999966	24.770001	24.77000058	TR24670|c2_g29_i1|CDS2	2
65	2	2	11.98000014	6.7709997	6.77099973	TR24403|c1_g2_i2|CDS1	1
66	2	2	10.58999971	5.5879999	5.587999895	TR22070|c0_g1_i1|CDS2	1
67	2	2	6.701000035	6.701	6.701000035	TR21070|c0_g4_i1|CDS2	1
67	0	2	6.701000035	6.701	6.701000035	TR20772|c0_g2_i1|CDS1	1
68	2	2	14.55000043	14.55	14.55000043	TR10789|c0_g2_i1|CDS2	1
