# Supplementary figures and images for: True Lies: Using Proteomics to Assess the Accuracy of Transcriptome-Based Venomics in Centipedes Uncovers False Positives and Reveals Startling Intraspecific Variation in Scolopendra subspinipes
Source: Toxins (Basel). 2018 Feb 28;10(3):96. doi: 10.3390/toxins10030096 (PMC5869384; doi:10.3390/toxins10030096)

BPFTx

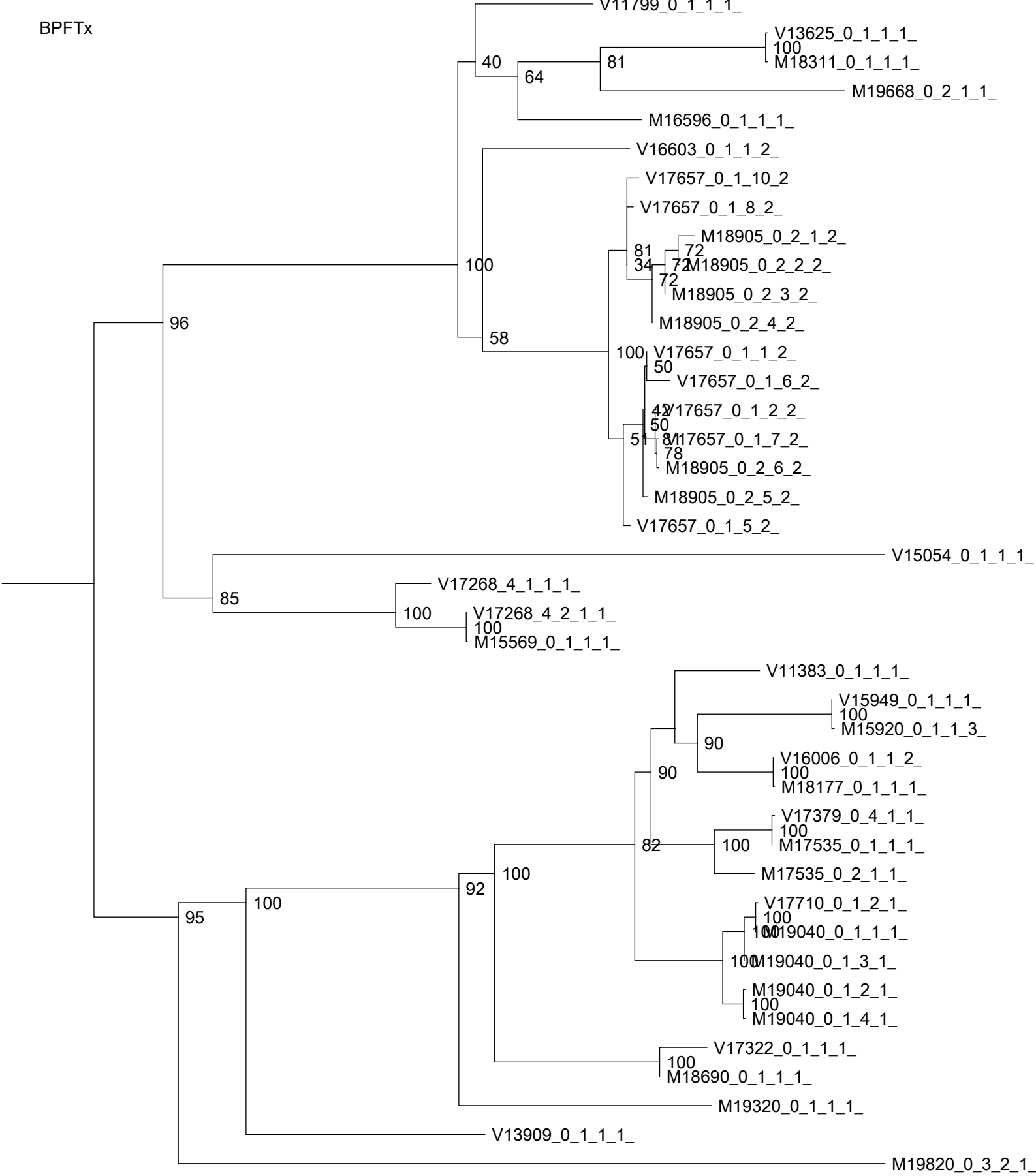

0.1

CAP1

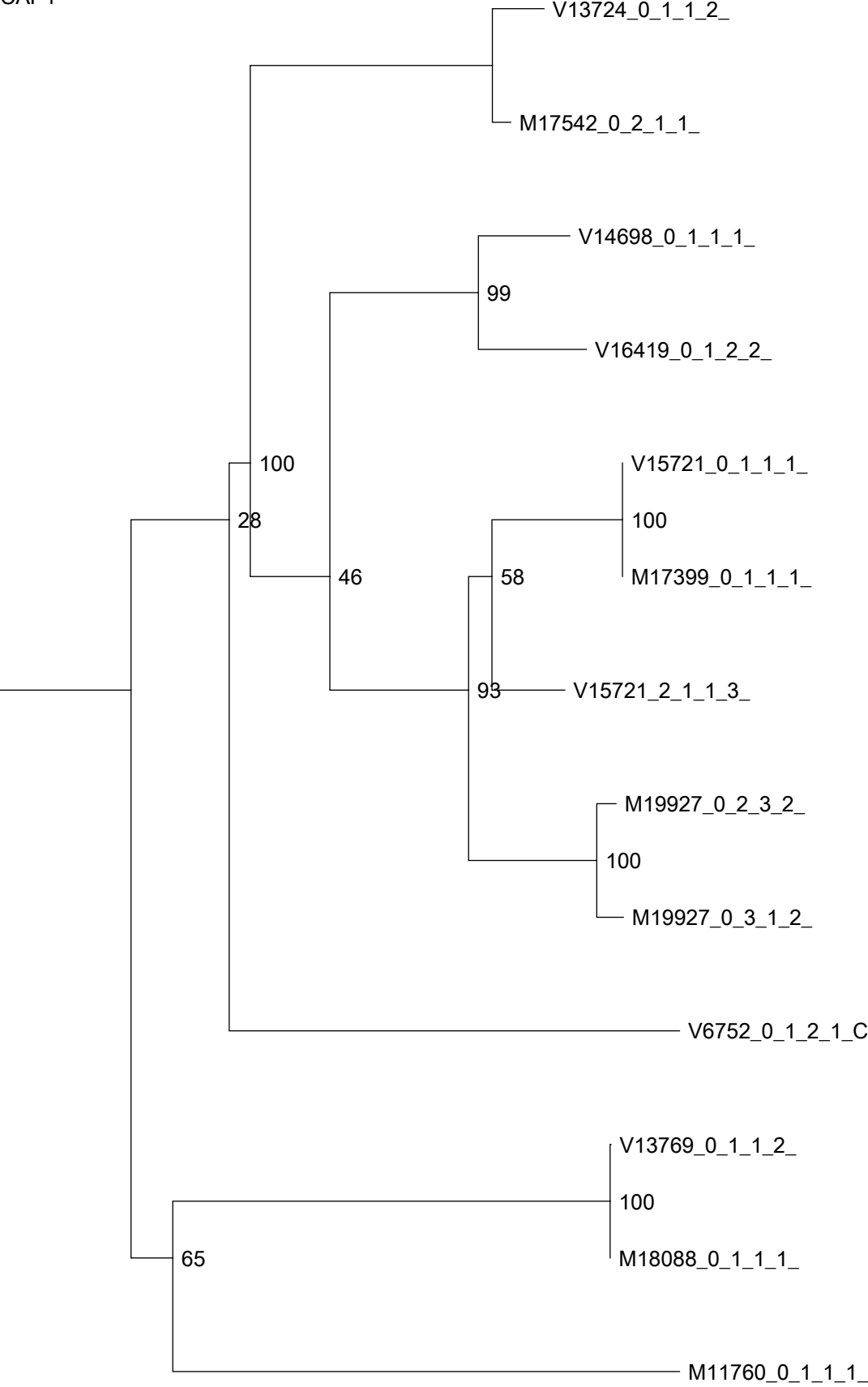

0.1

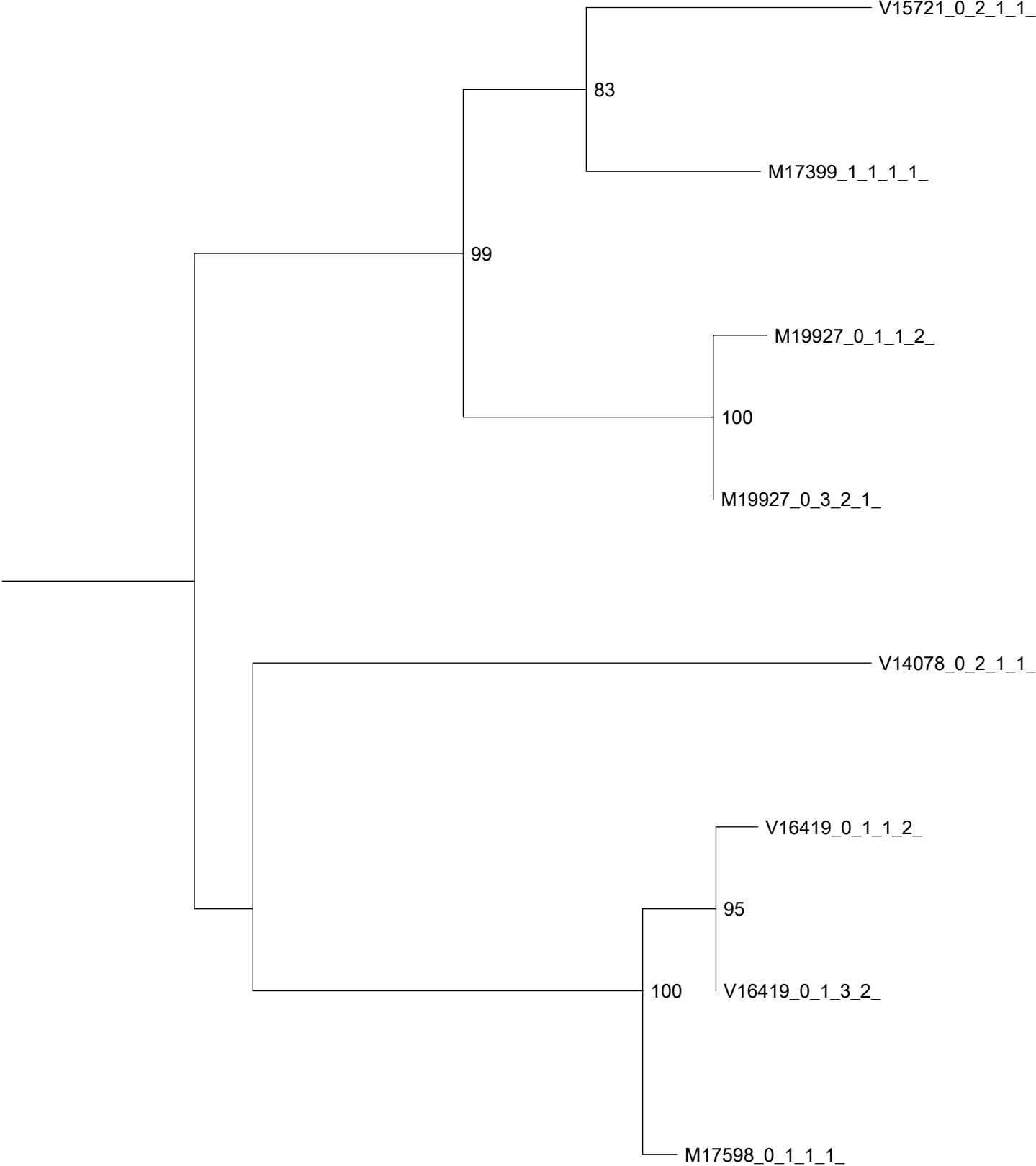

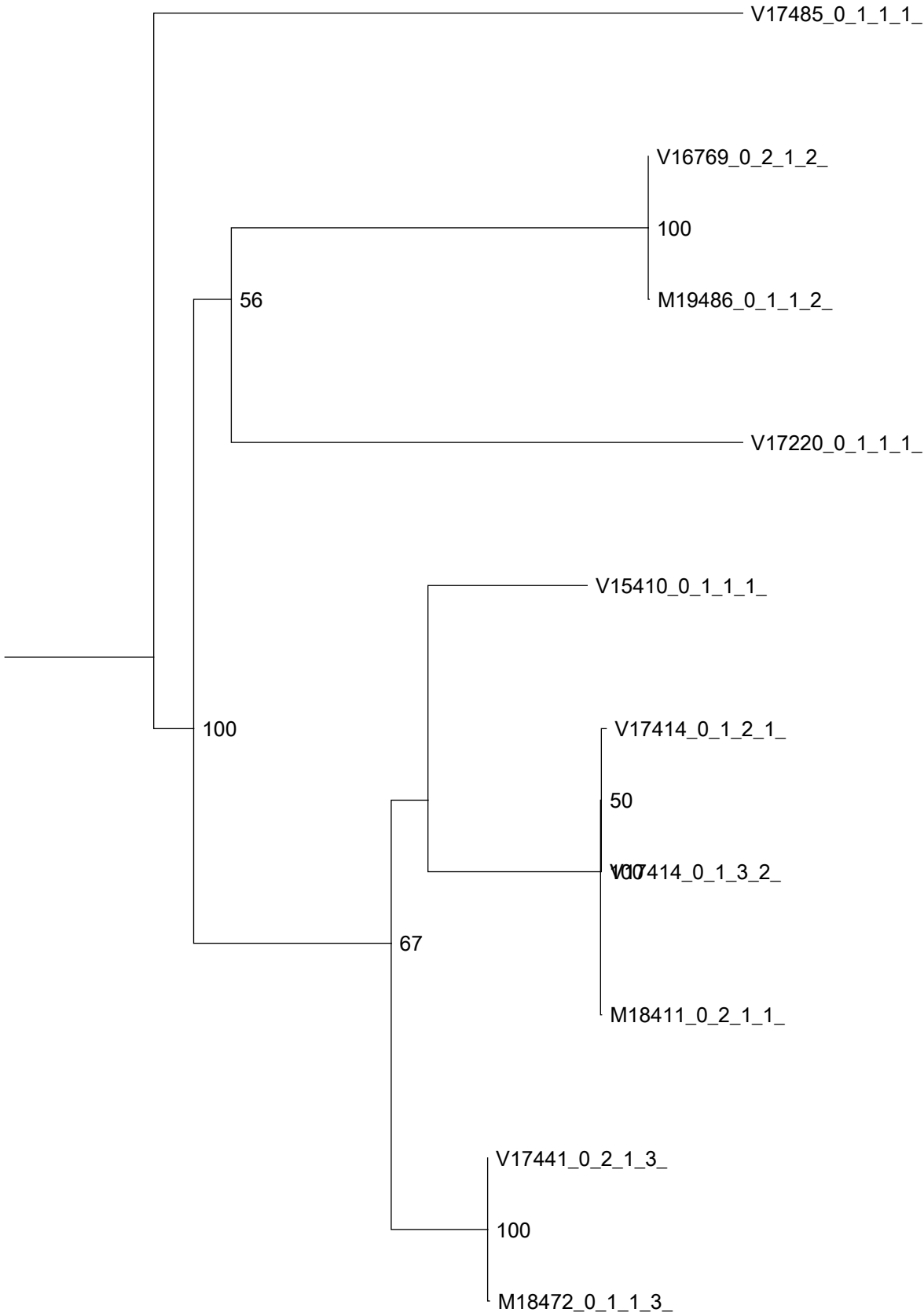

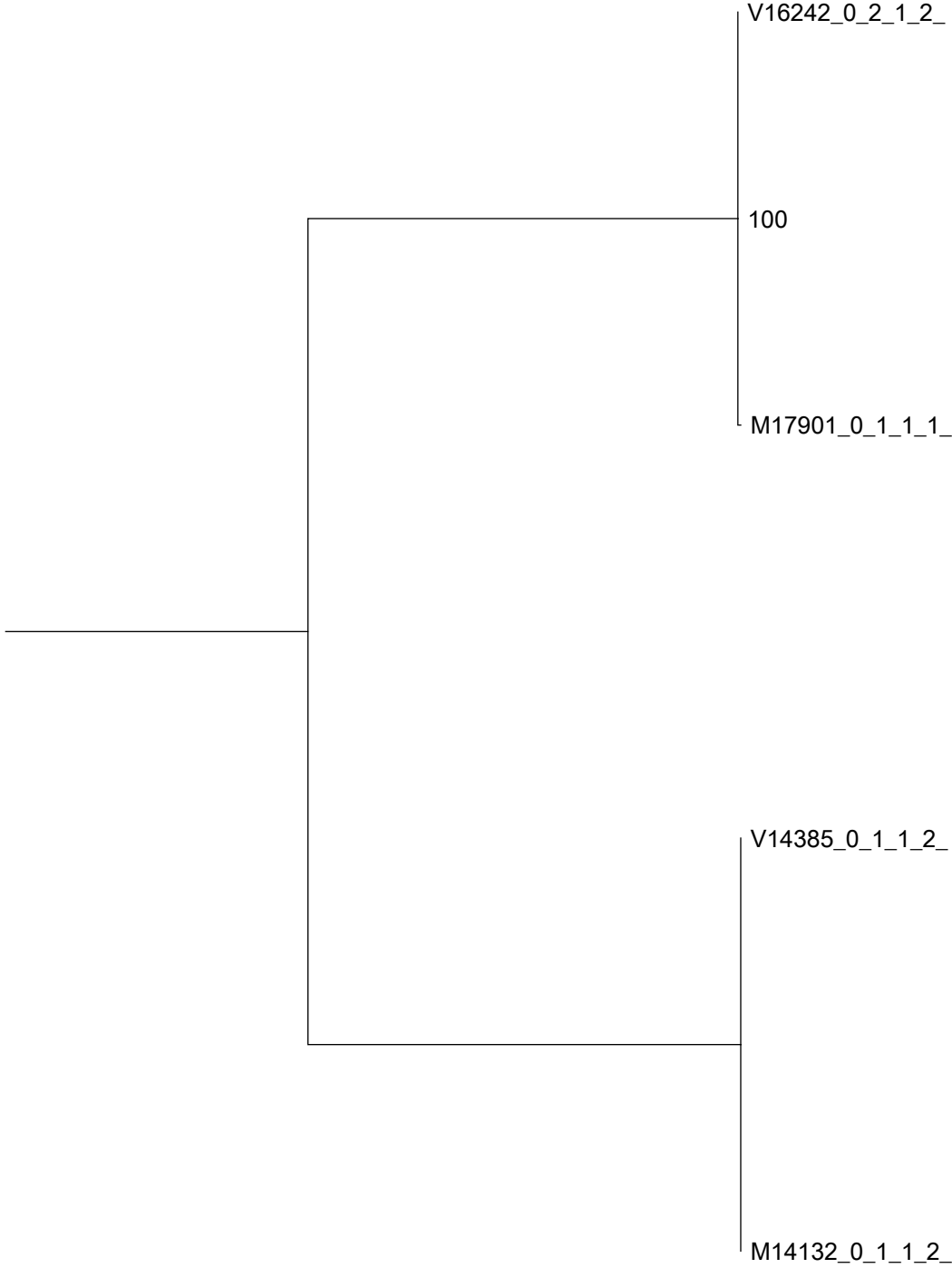

GDH

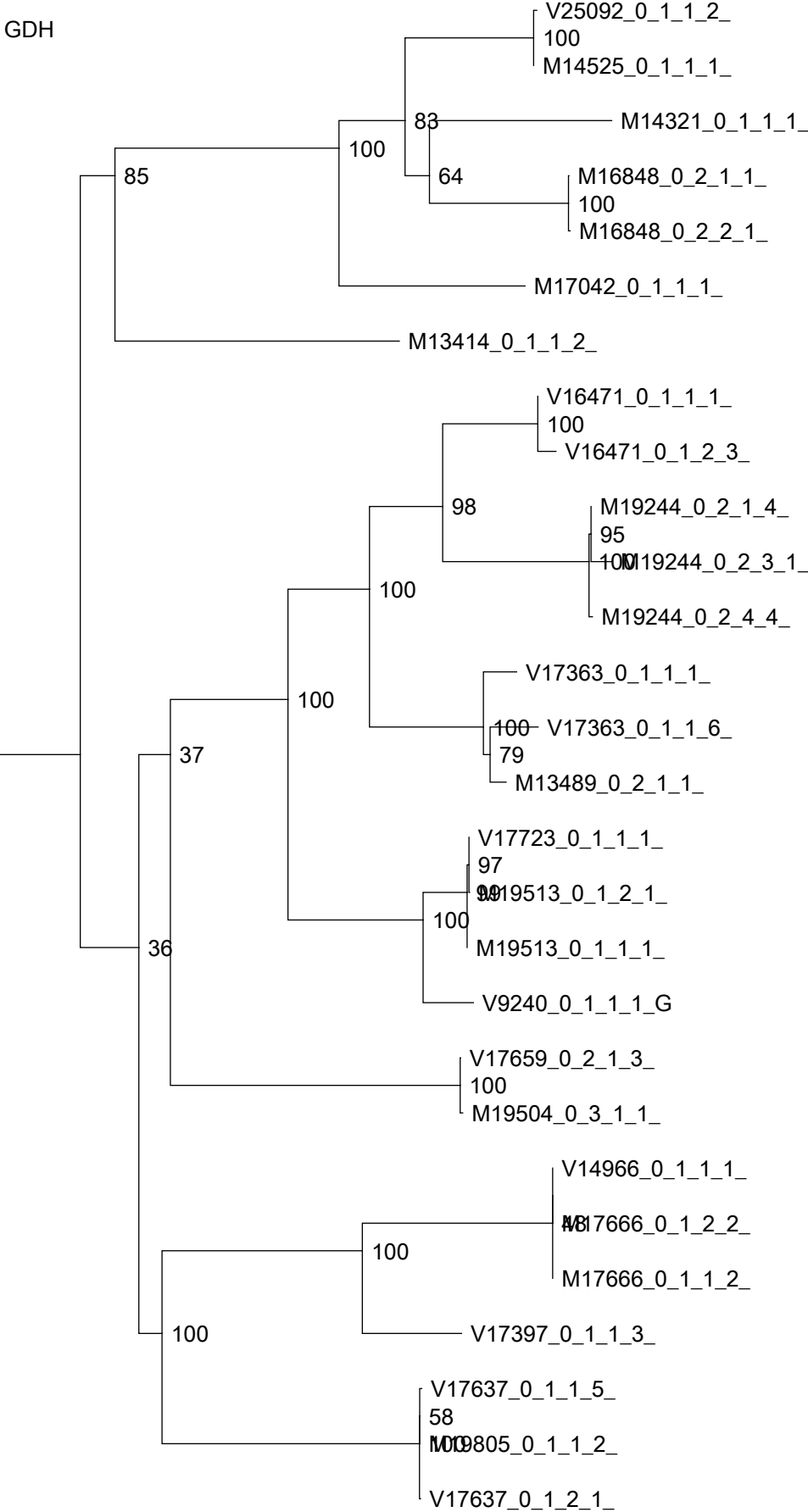

0.1

LDLA

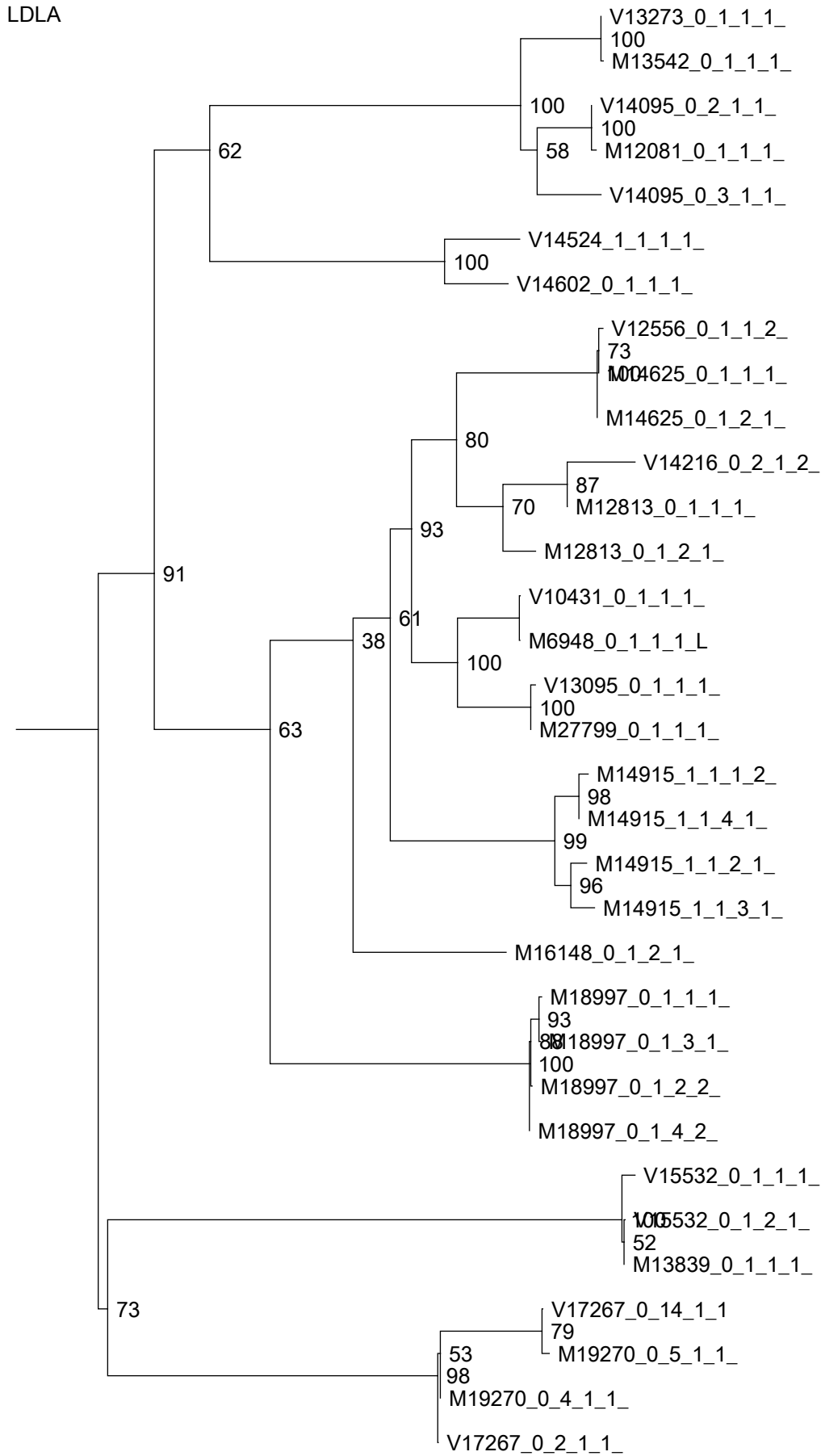

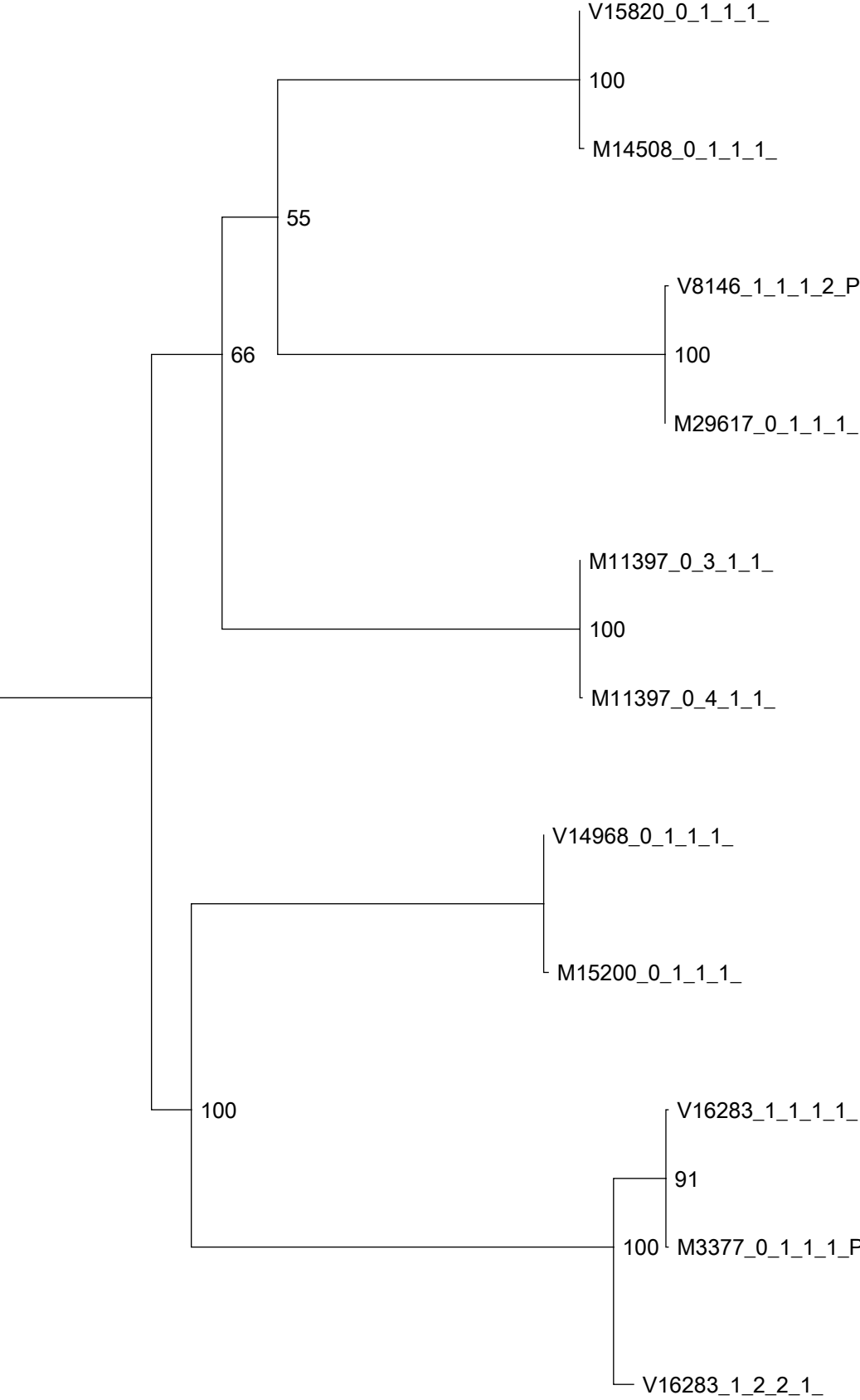

pM12A

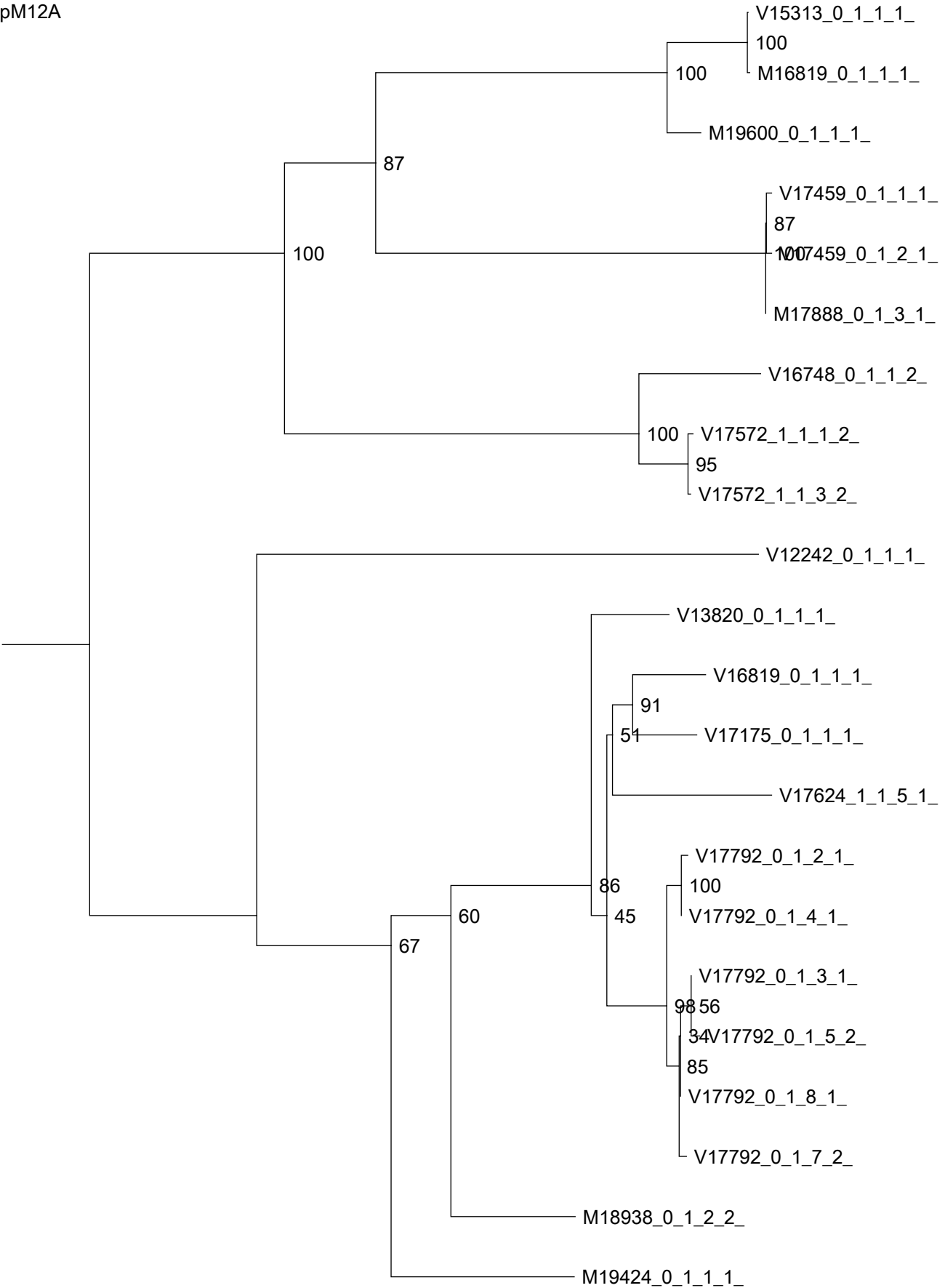

0.1

S1

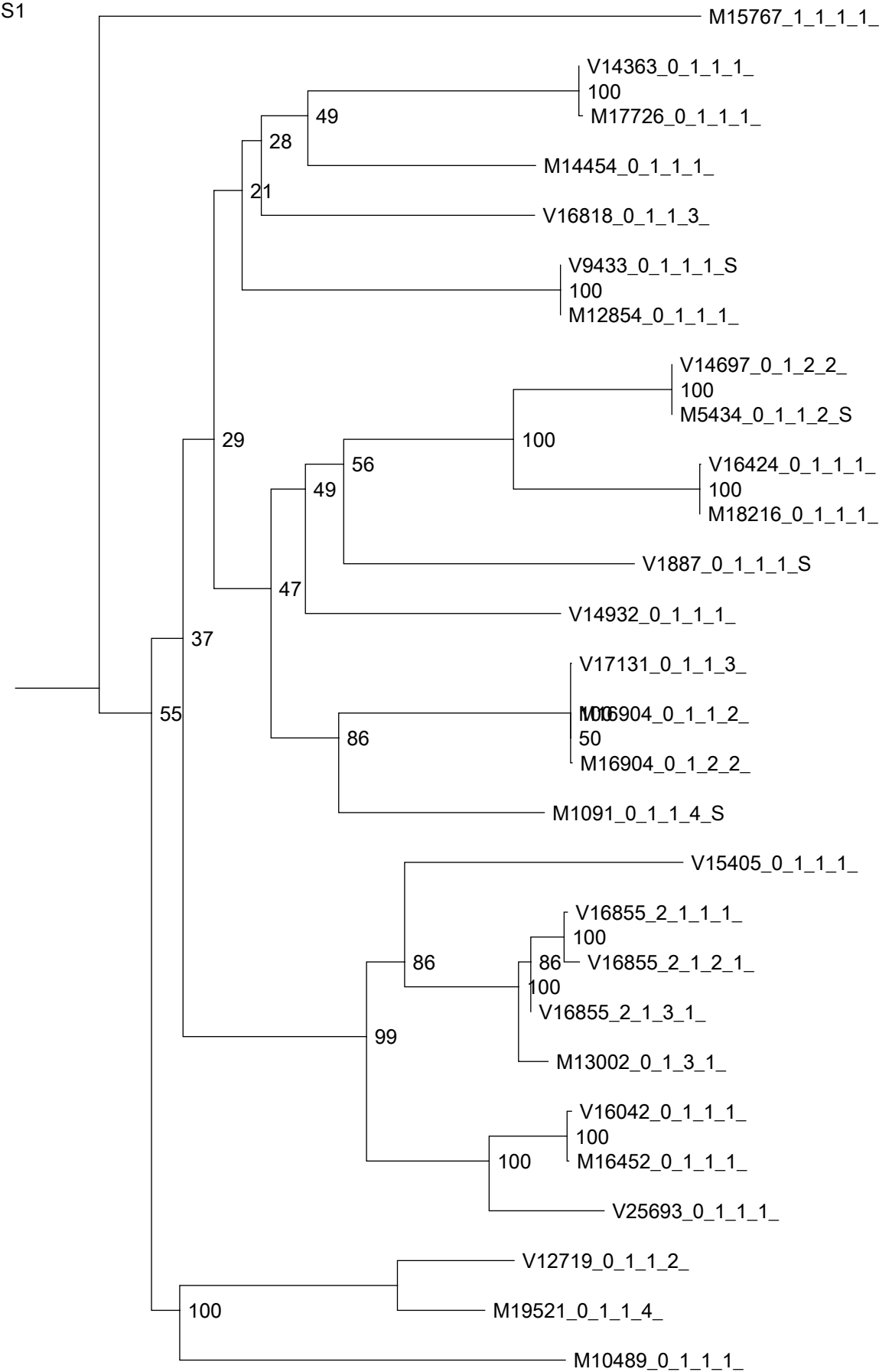

0.1

SLPTX01

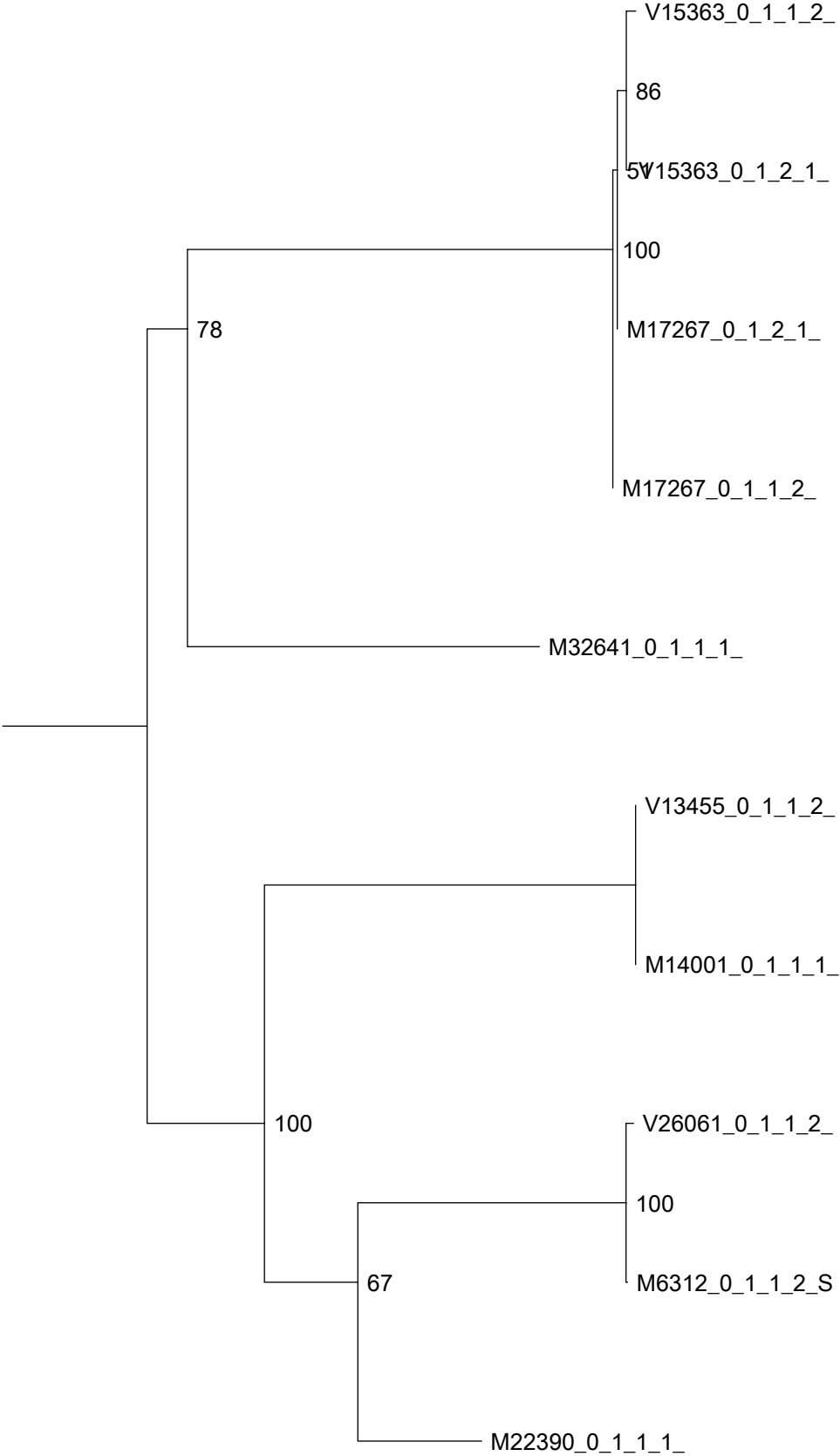

0.1

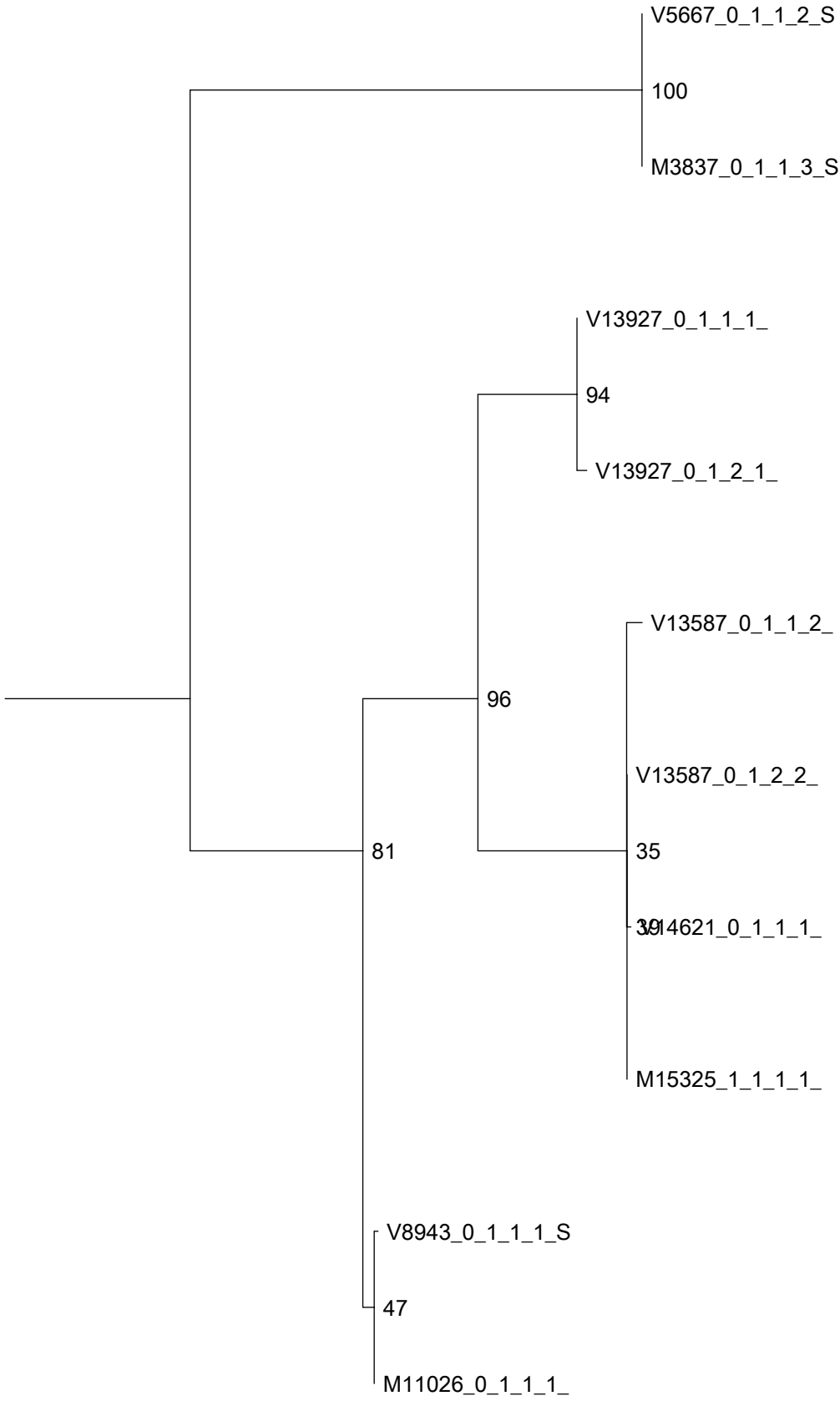

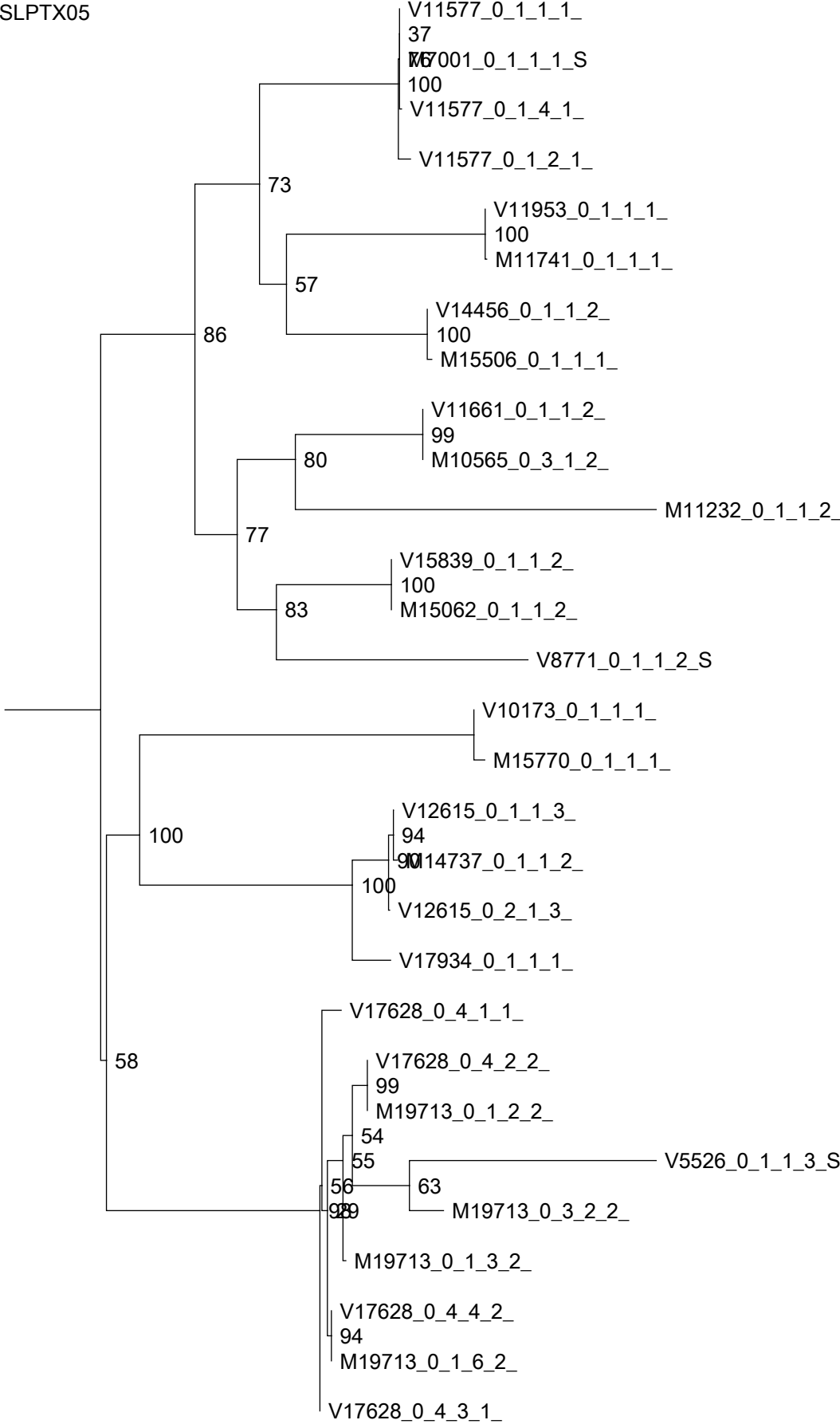

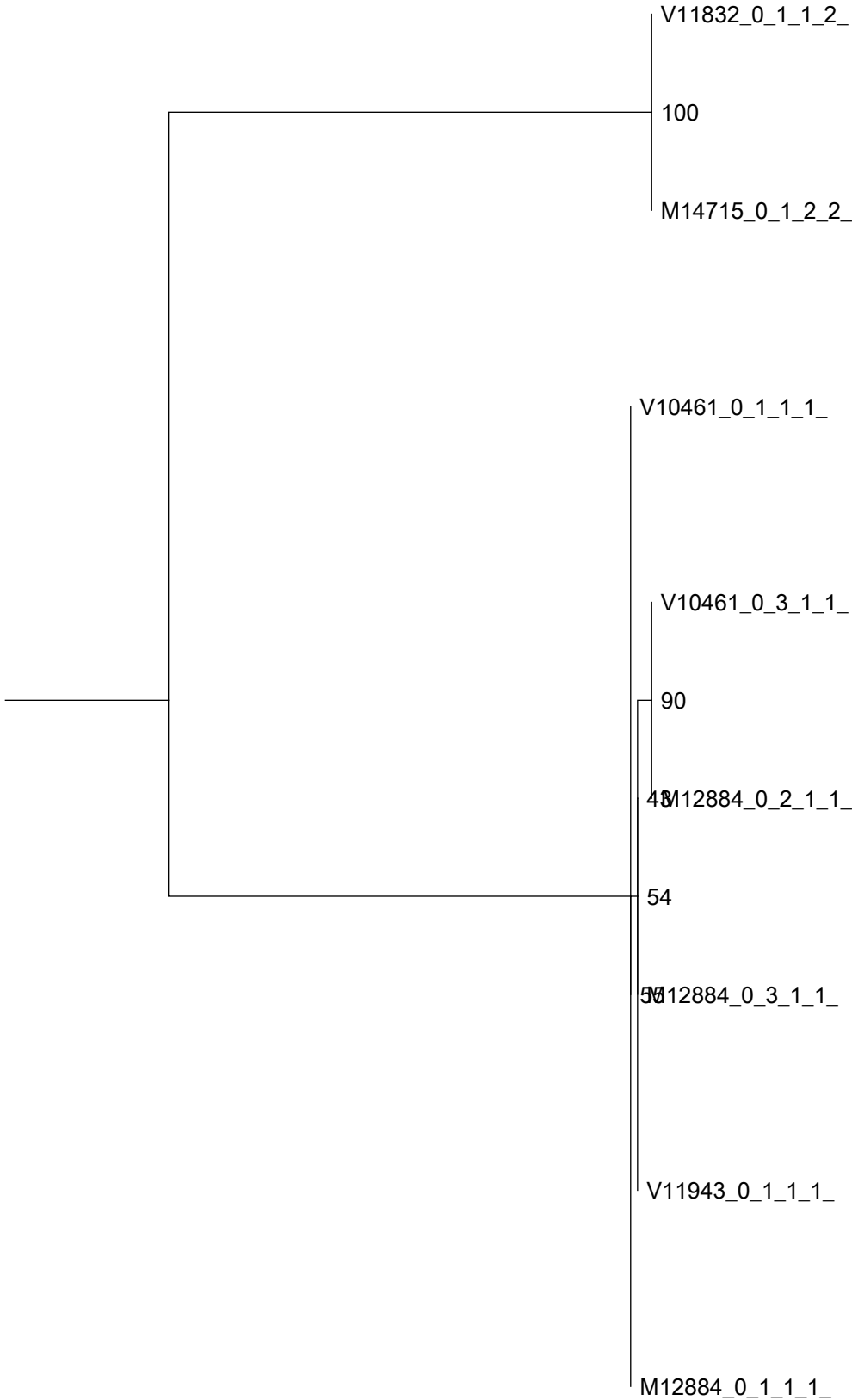

SLPTX10

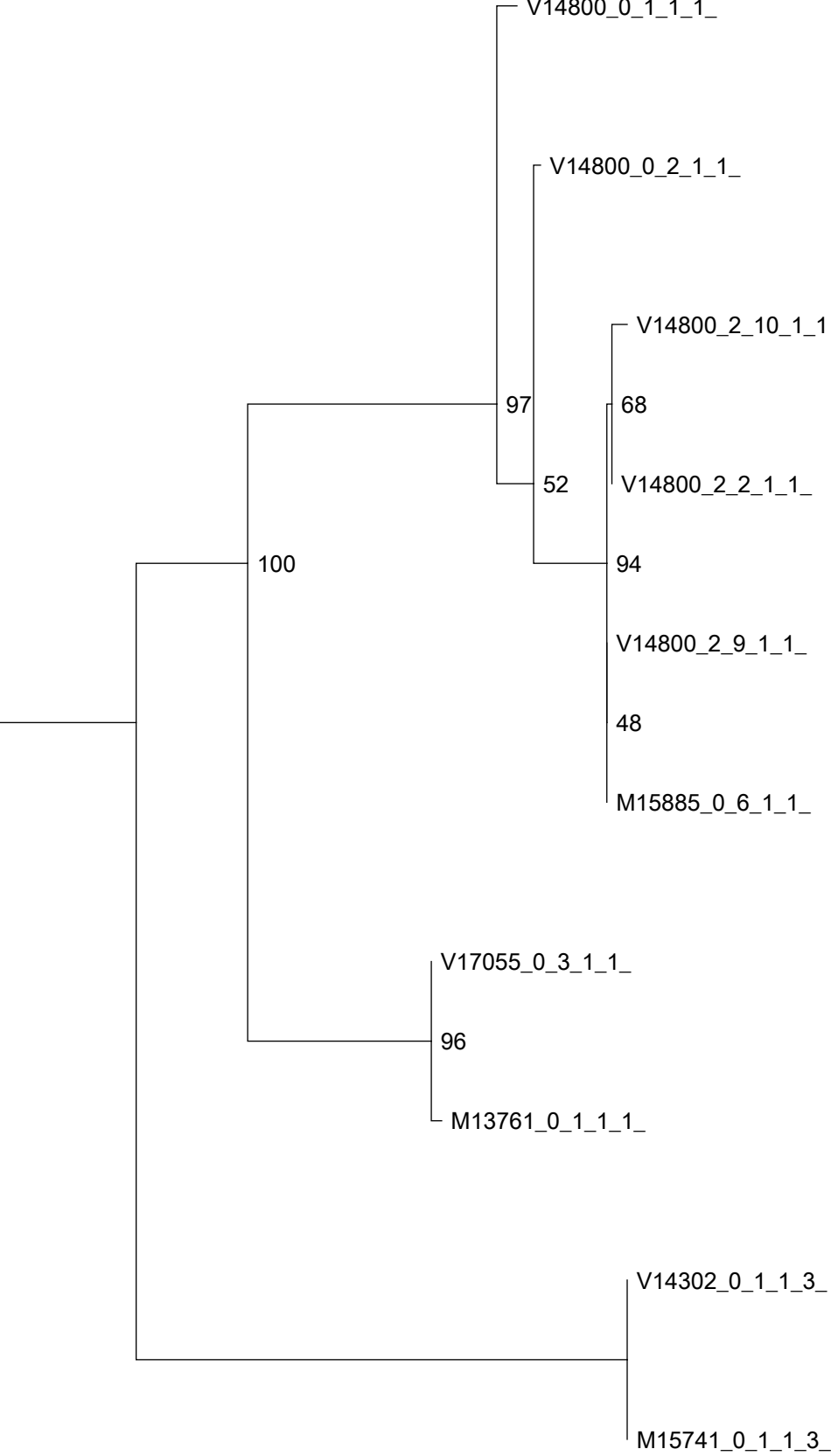

SLPTX11

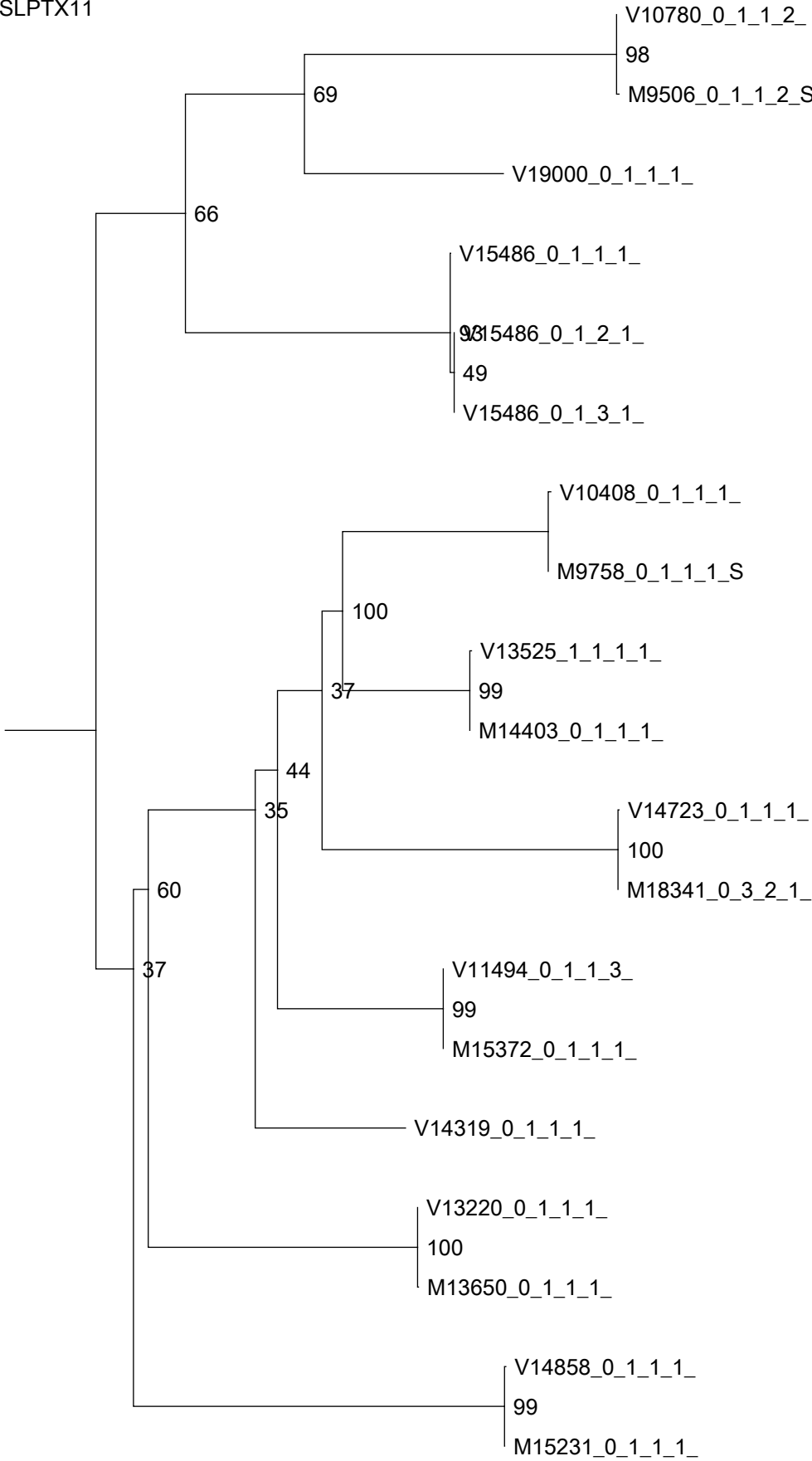

1.0

SLPTX12

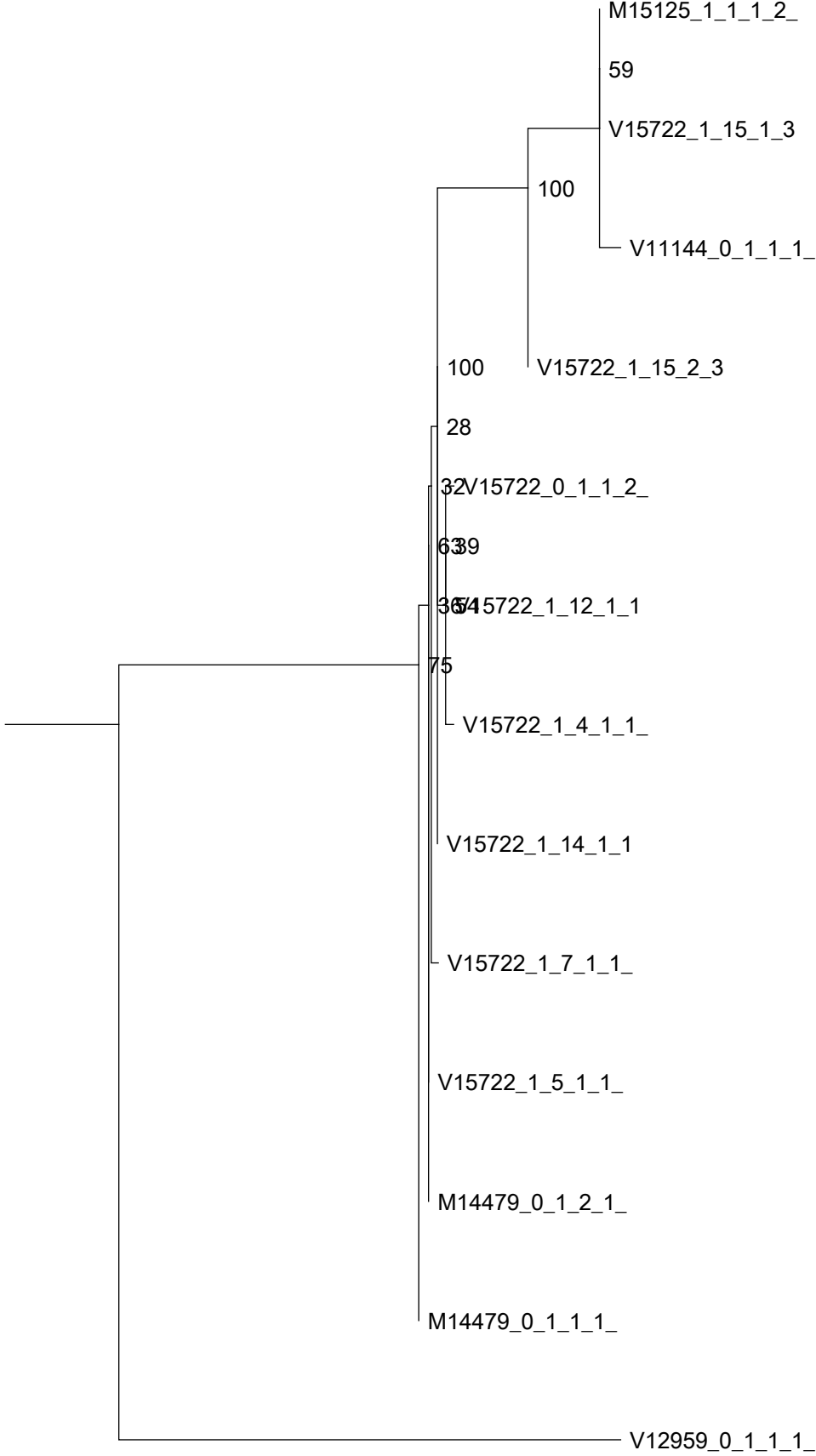

SLPTX14

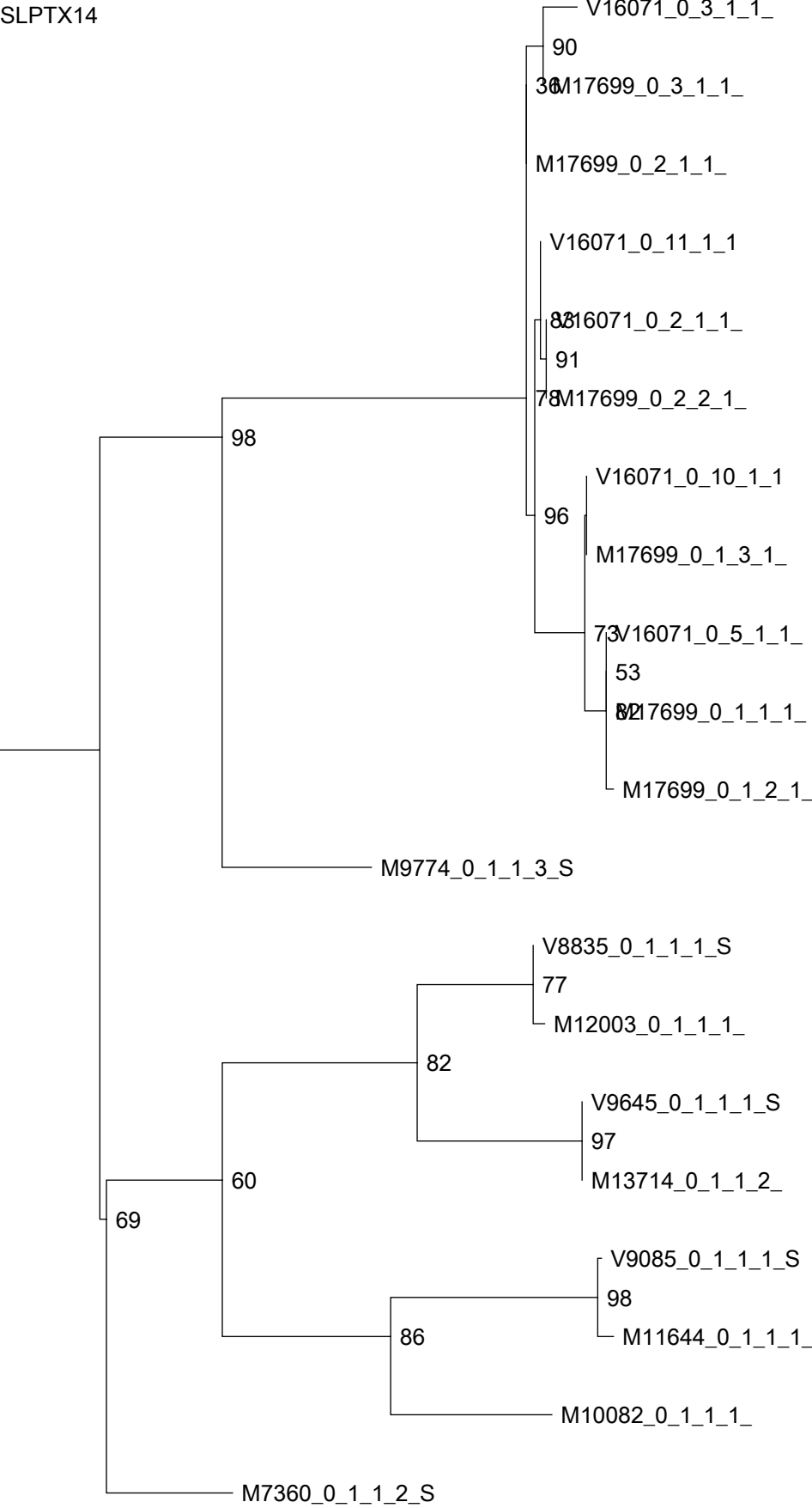

0.1

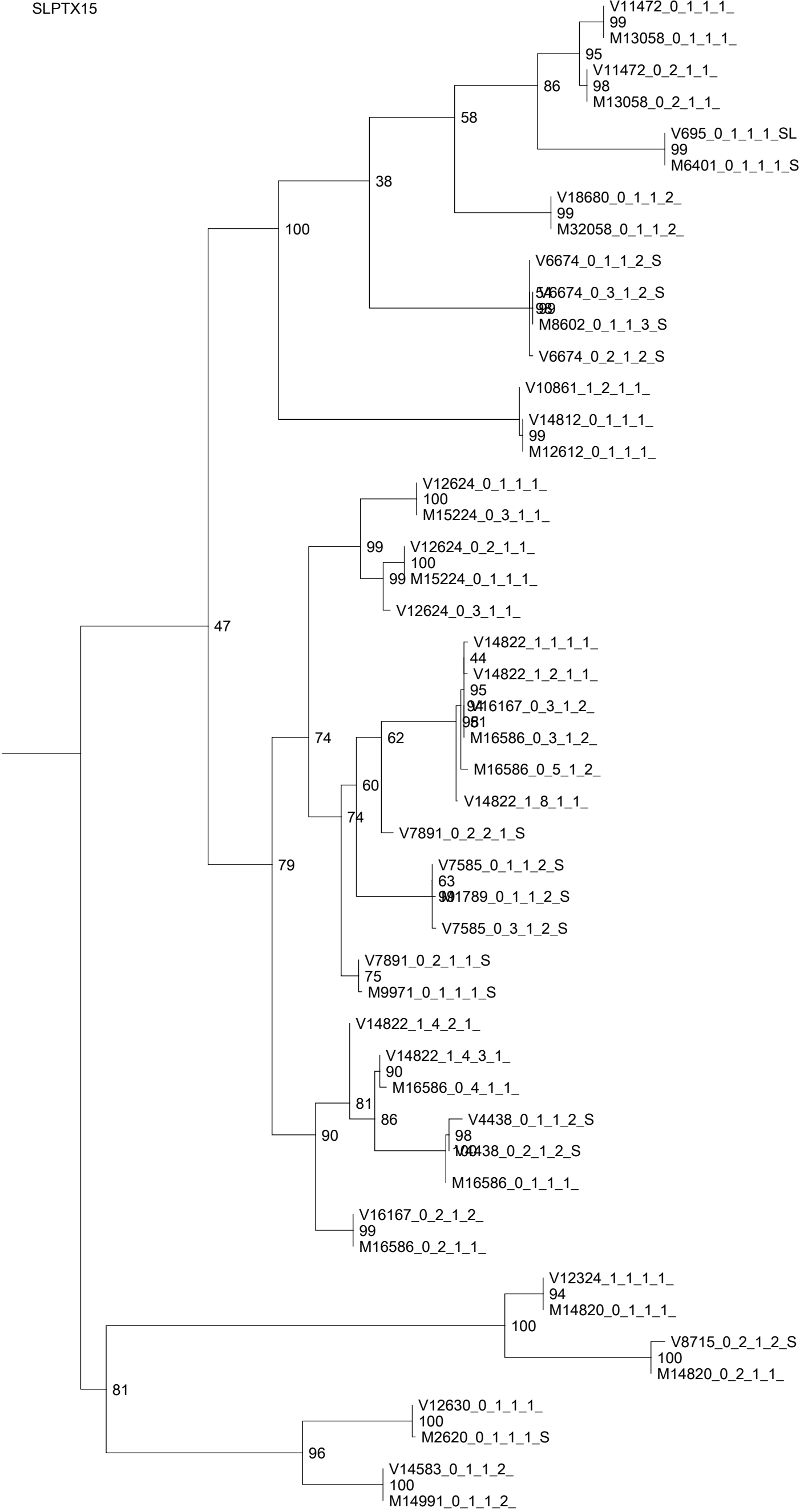

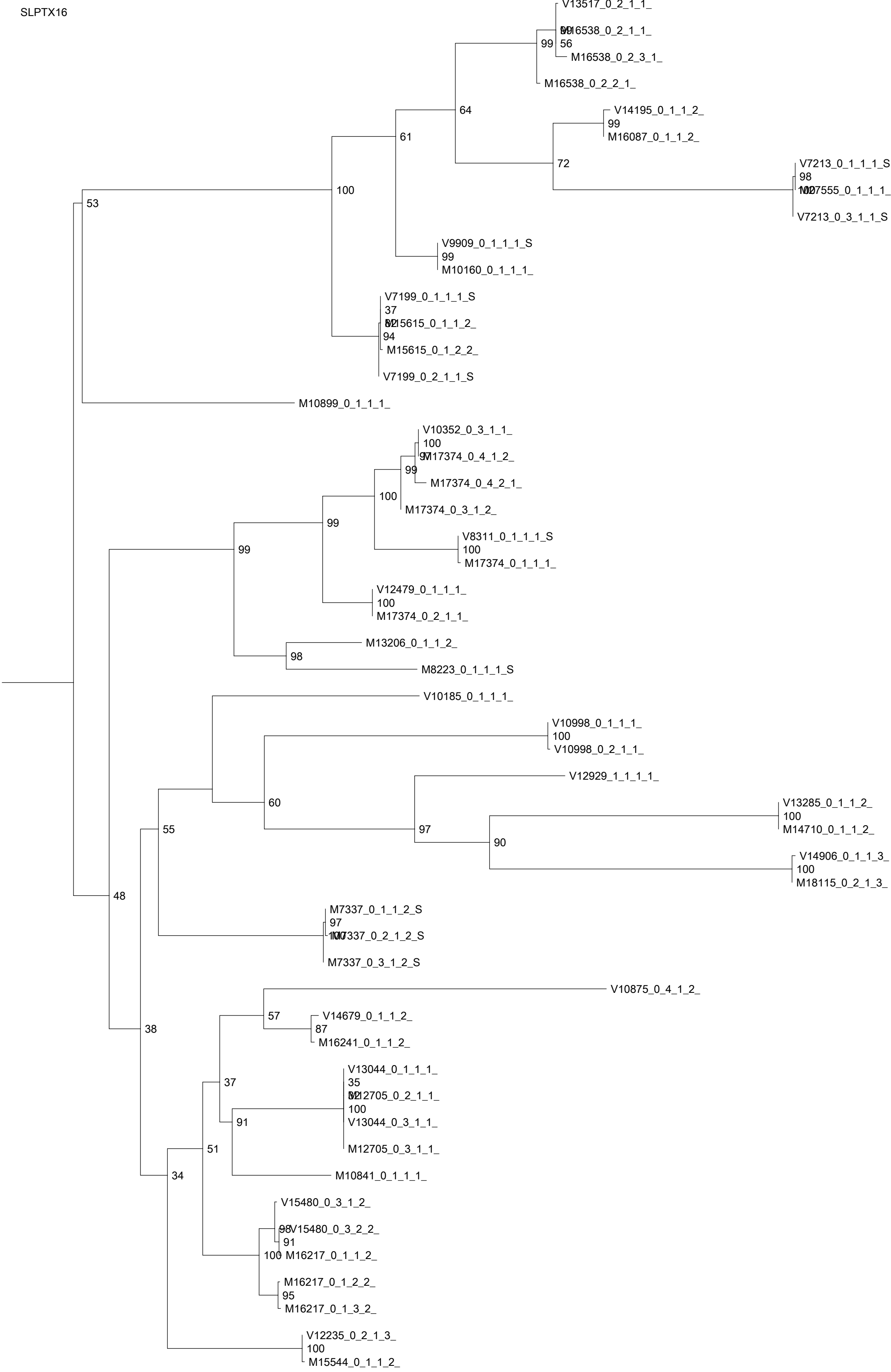

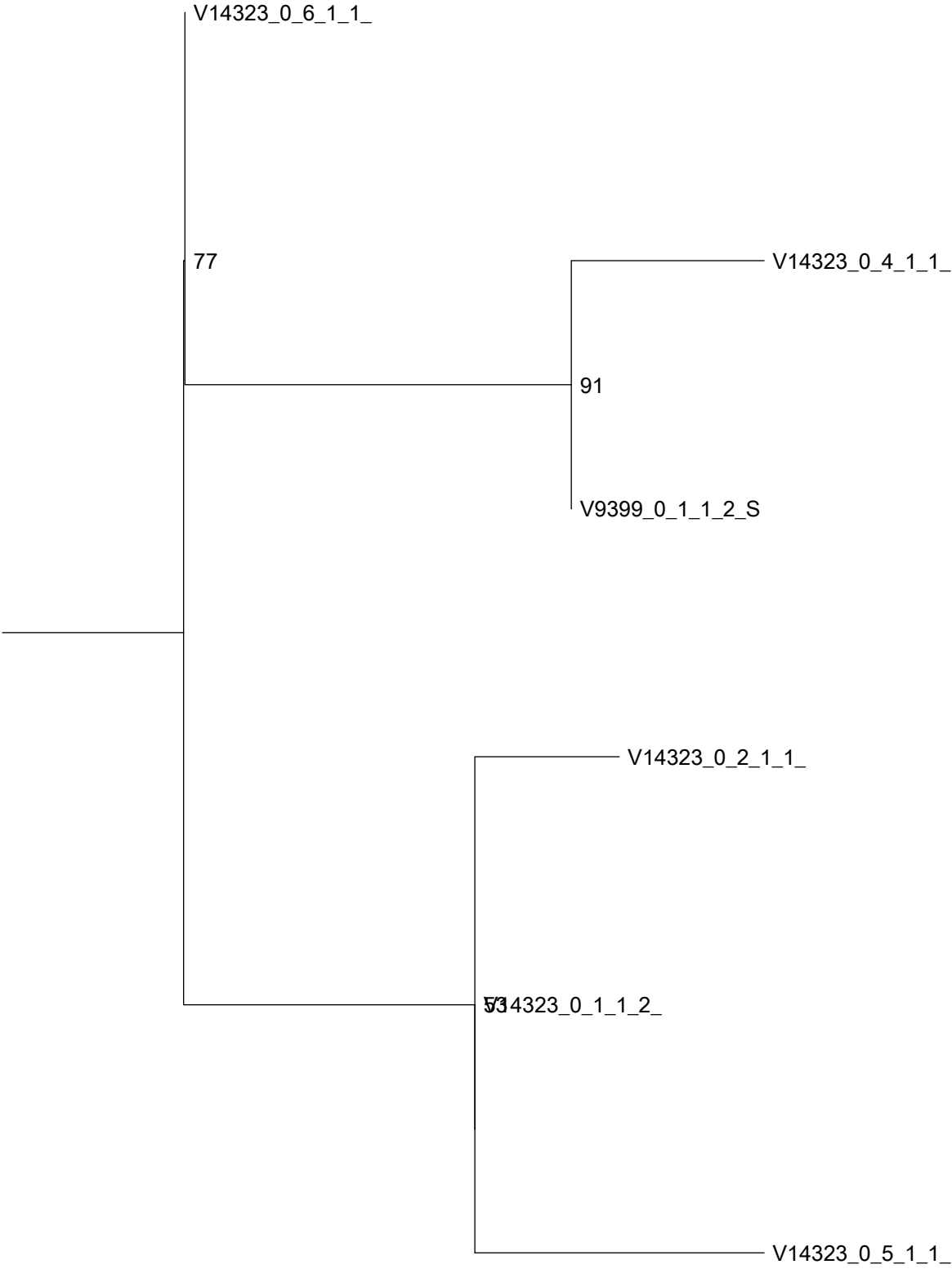

Transferrin

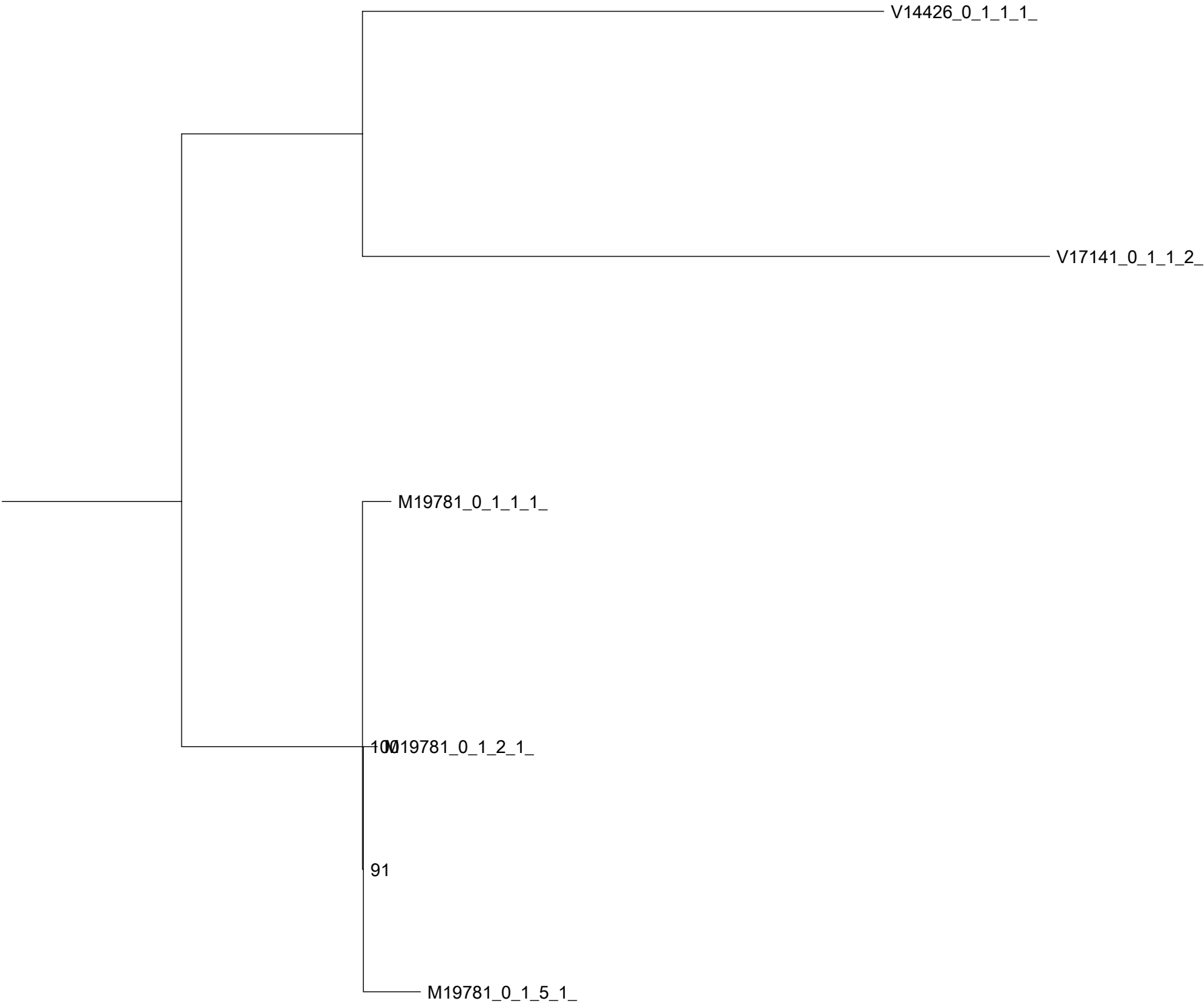

0.1

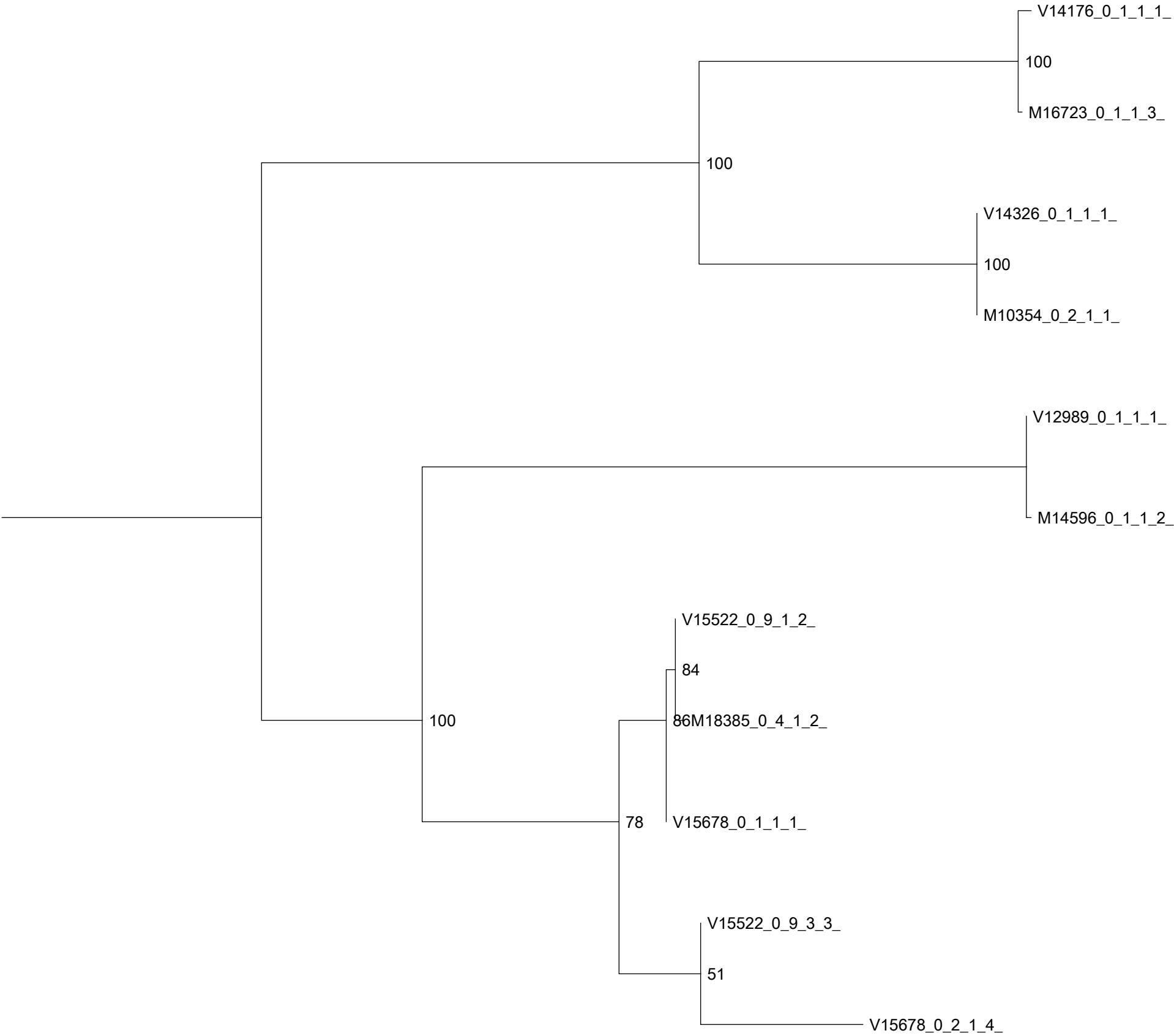

Supplement: Supplementary file 1 [file toxins-10-00096-s001.zip › Supplementary file 1.pdf]

BPFTx

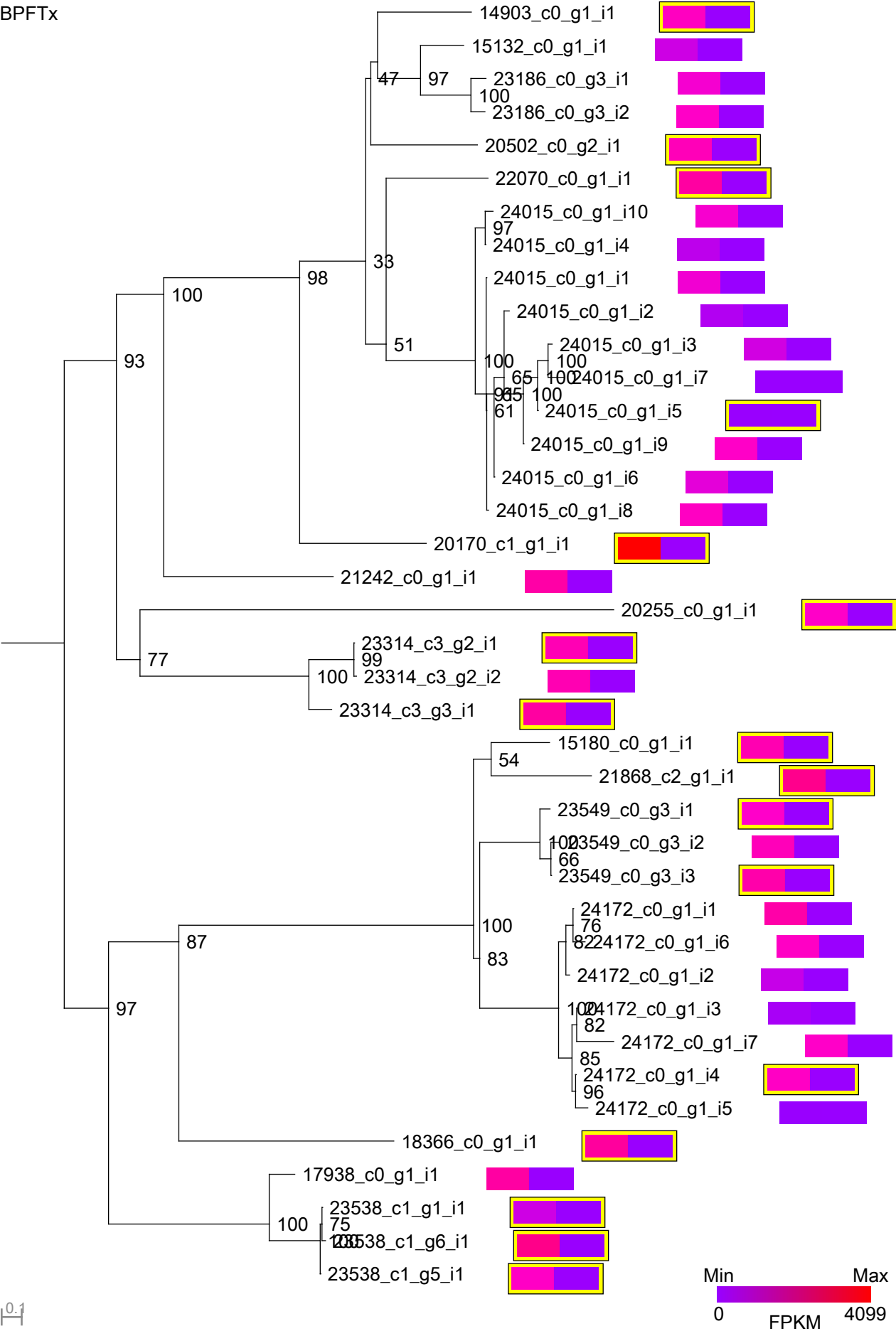

0.1

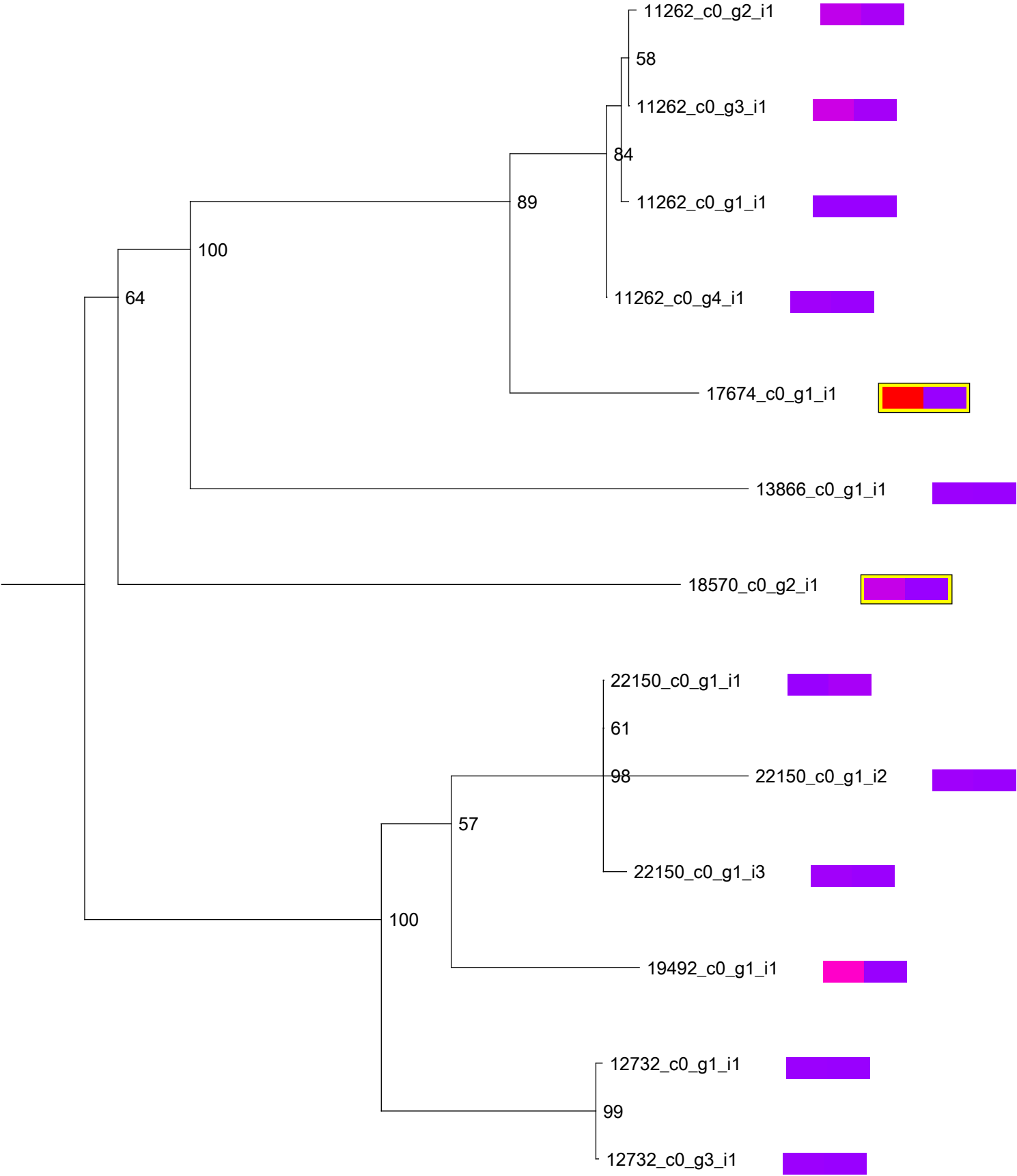

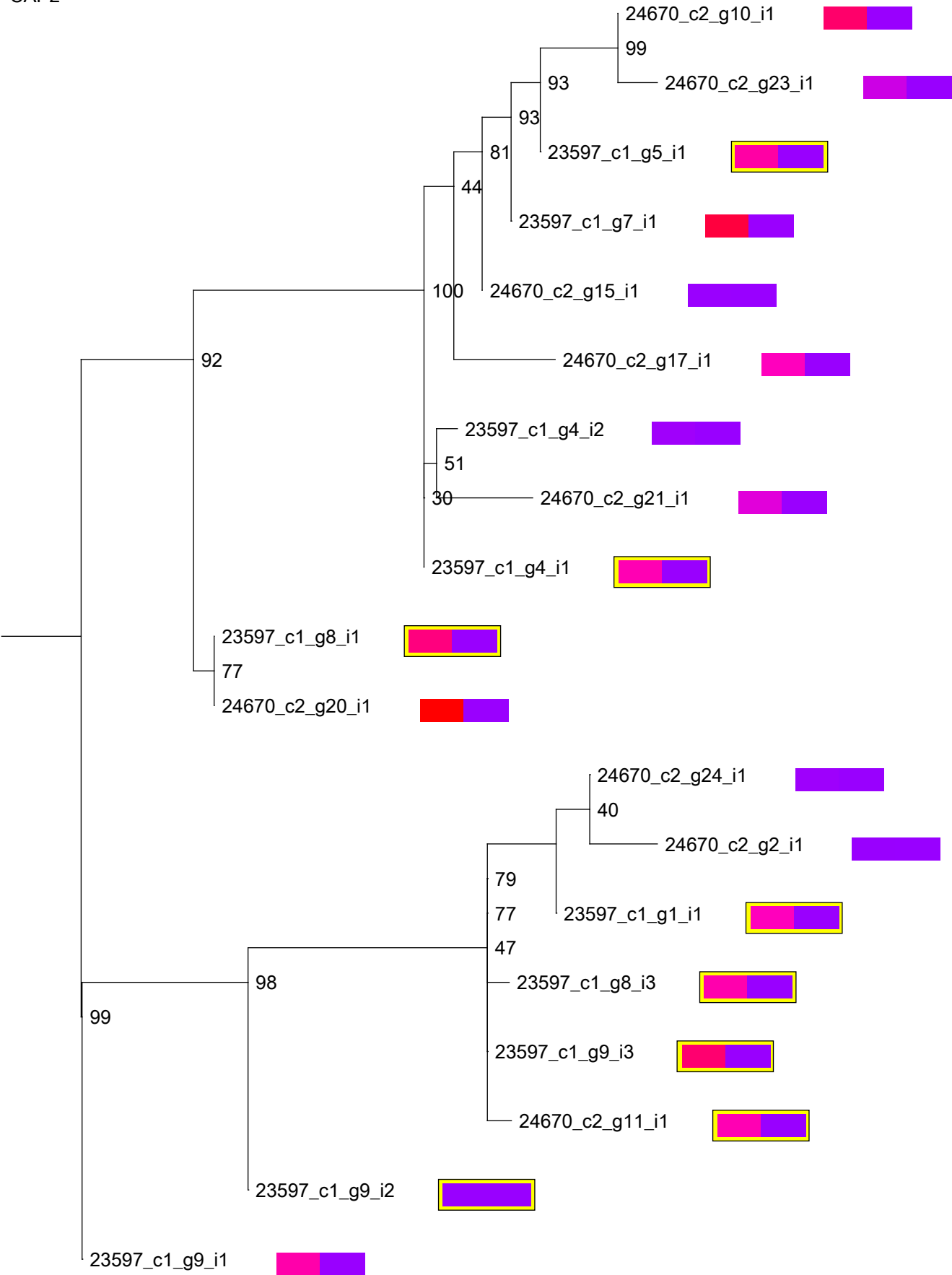

CO-Esterase B

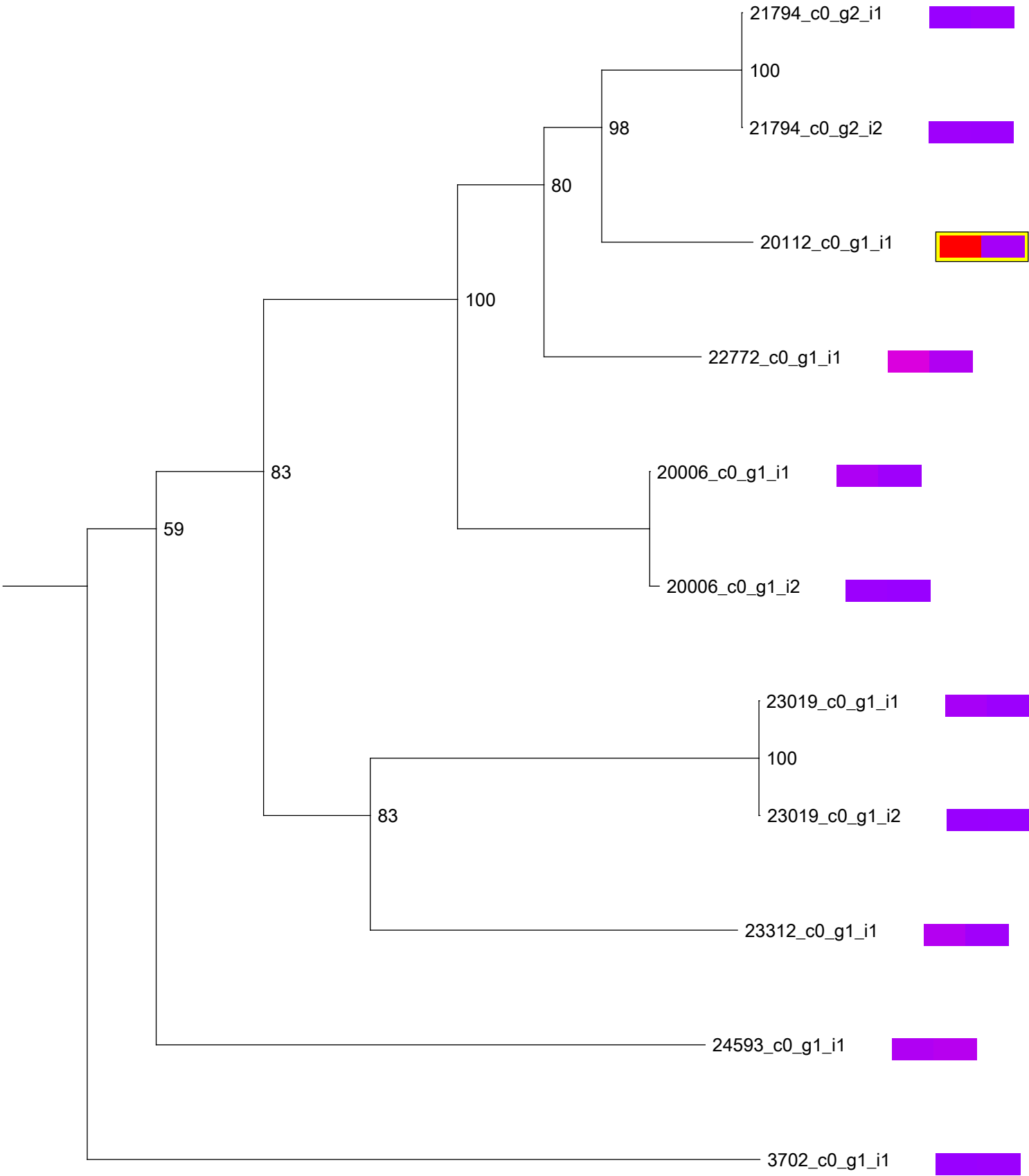

0.1

Min Max  
0 FPKM 857

GDH

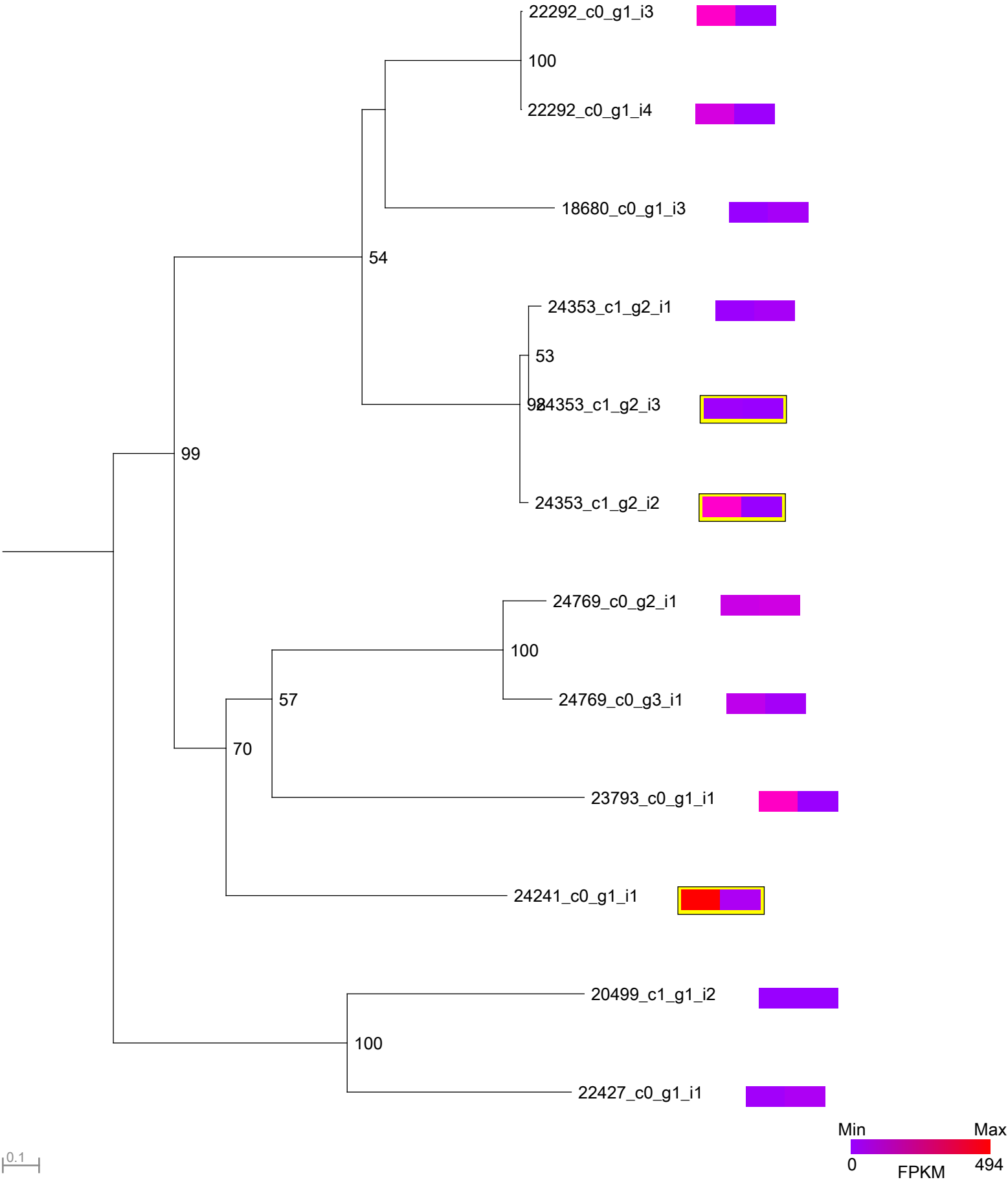

LDLA

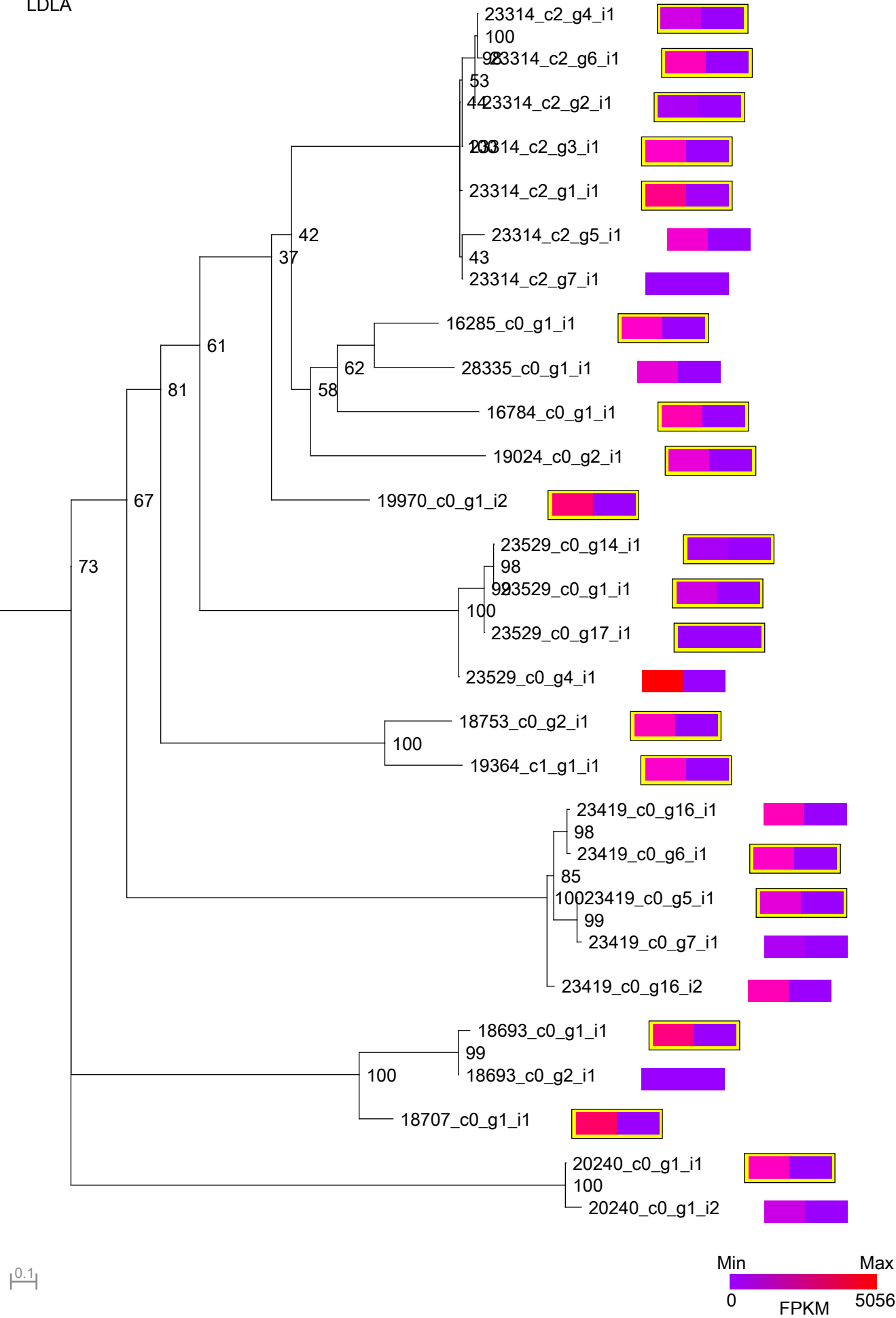

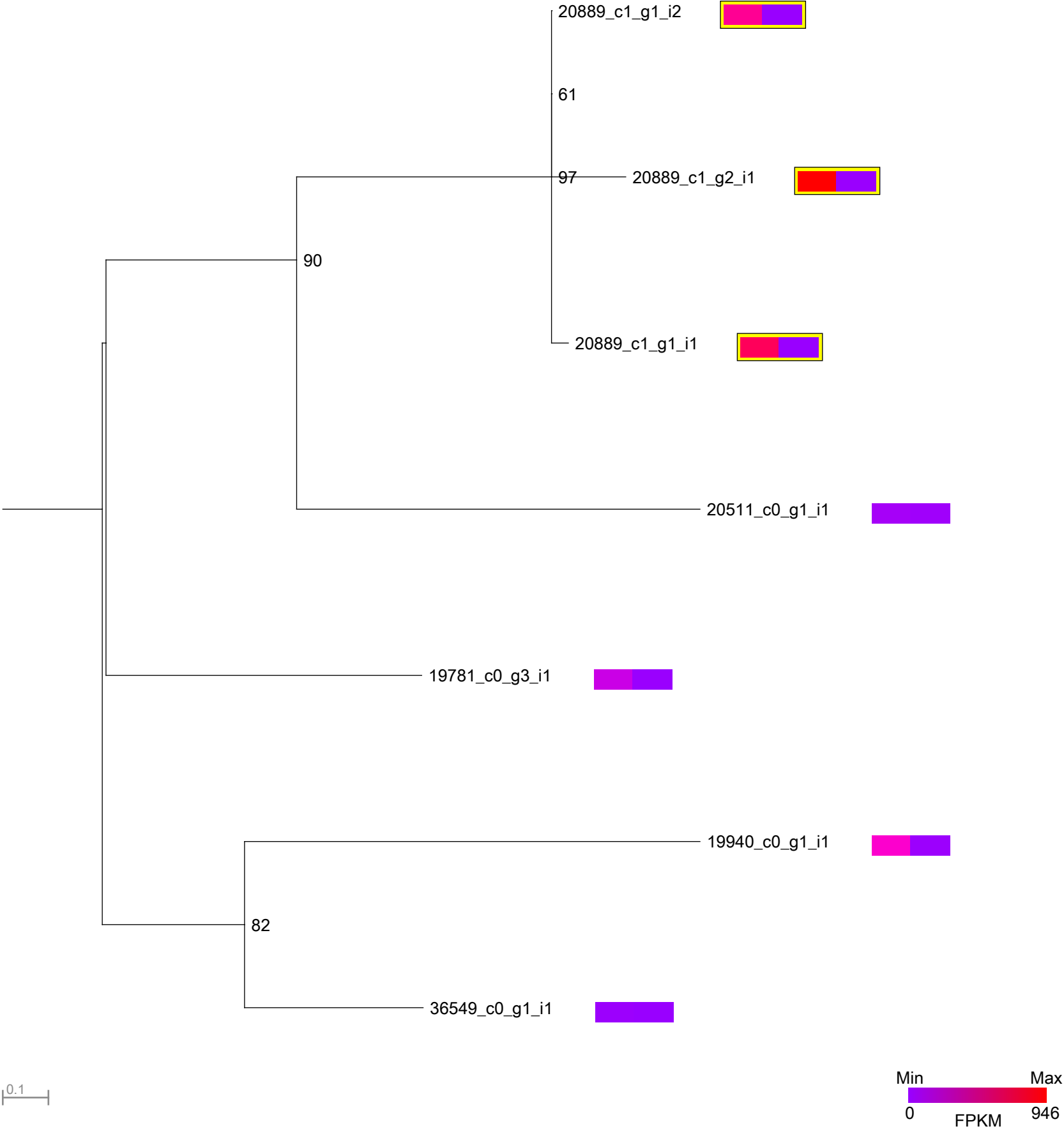

pM12A

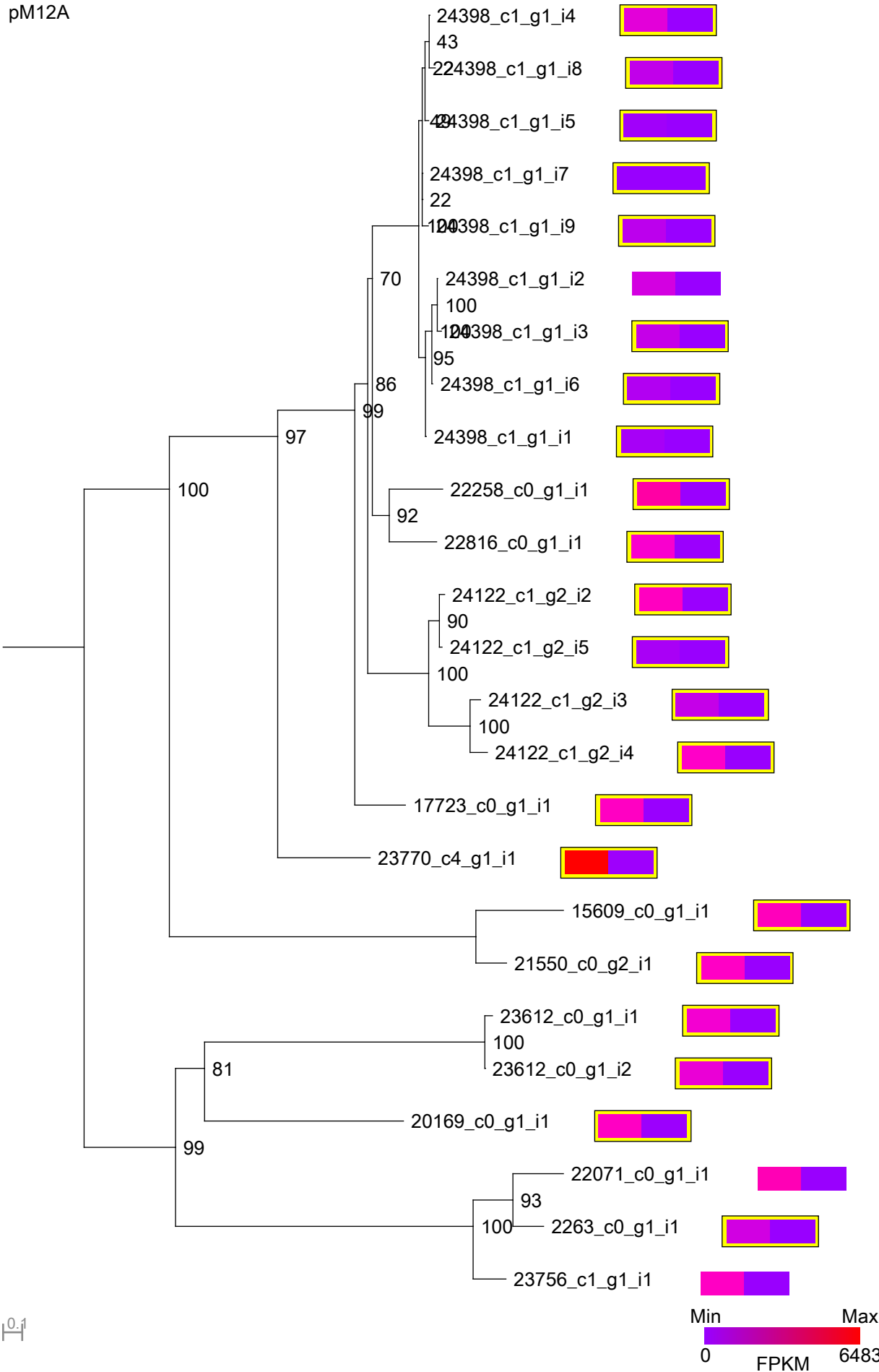

S1

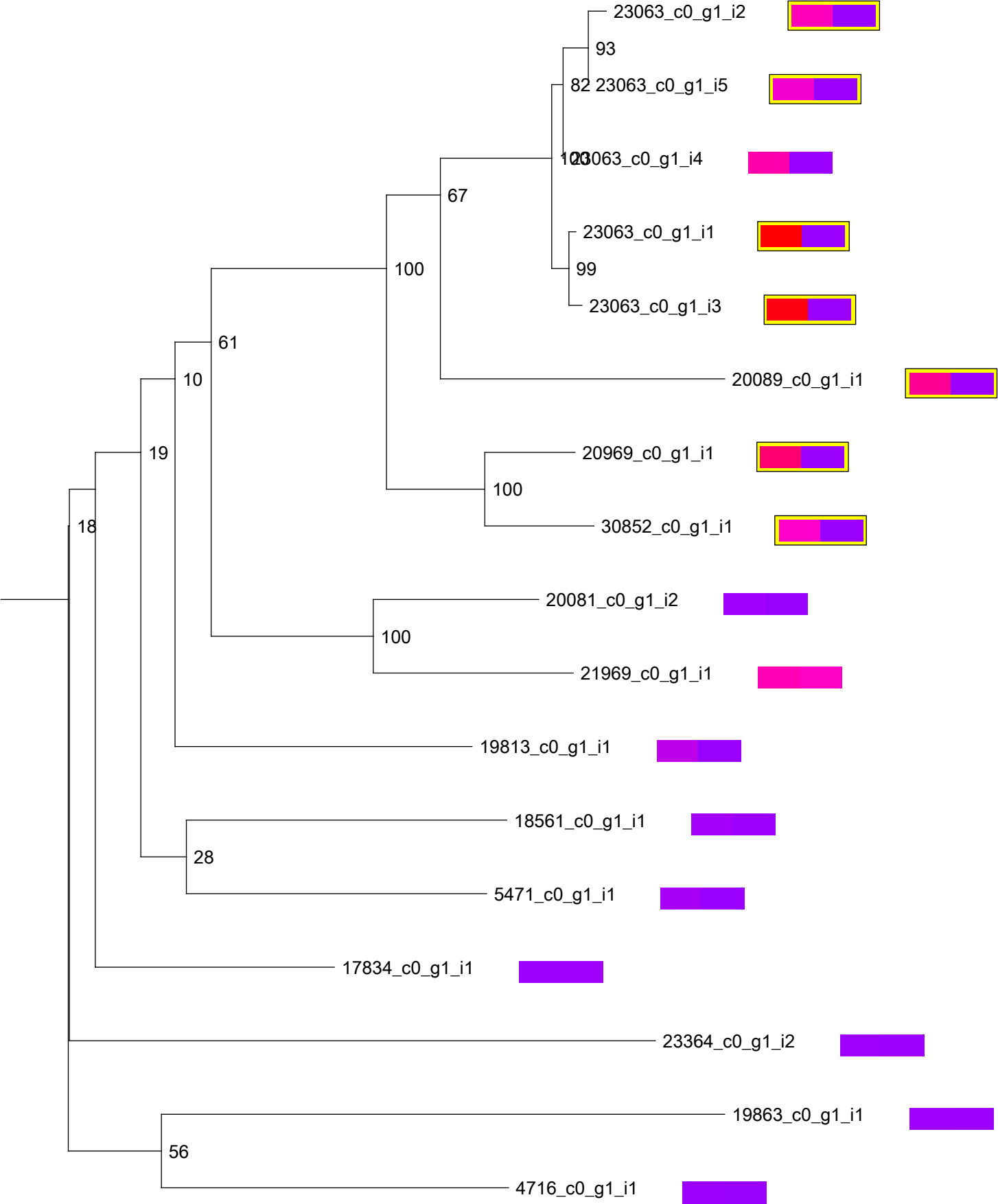

0.1

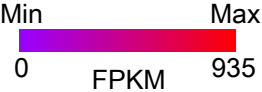

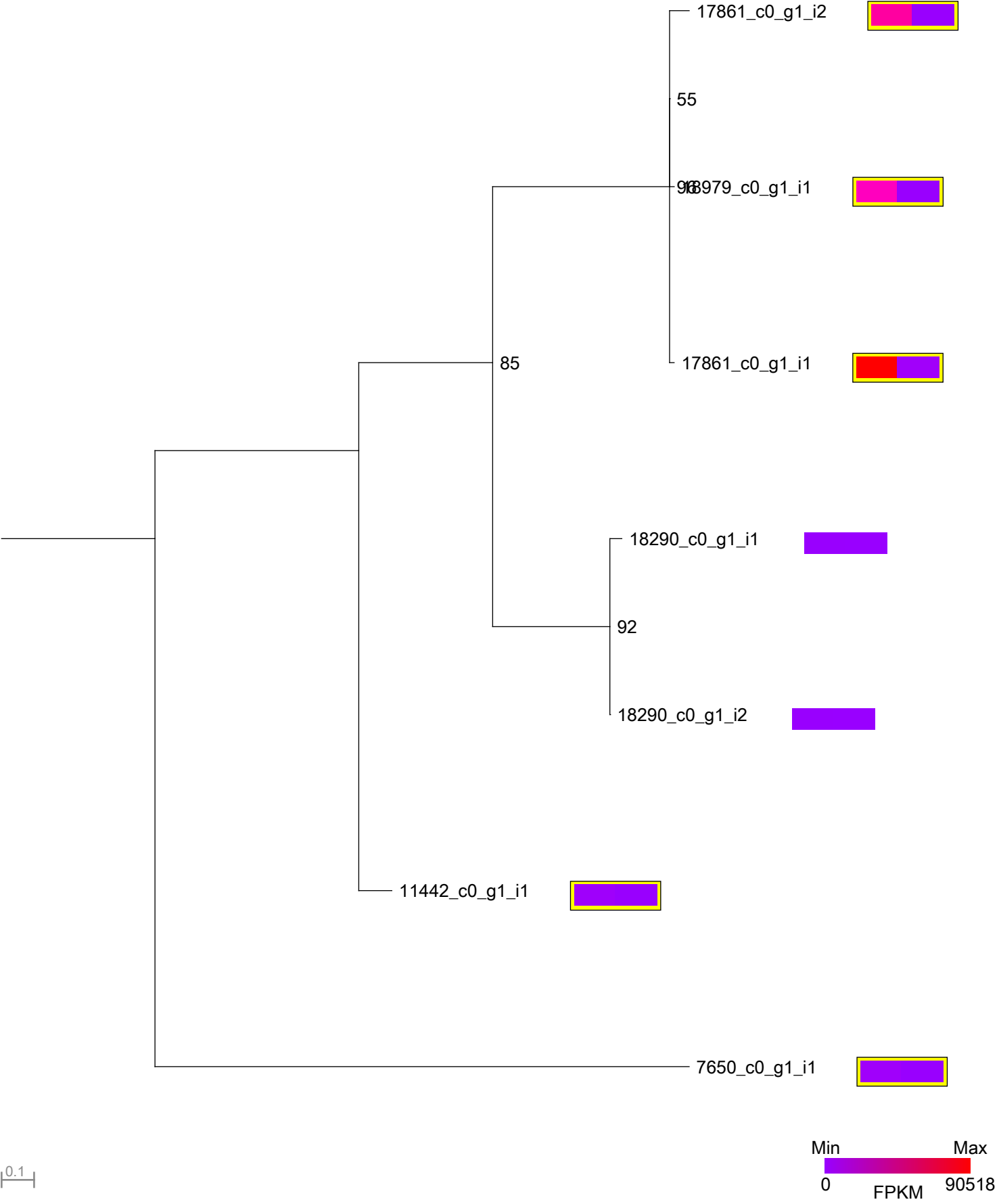

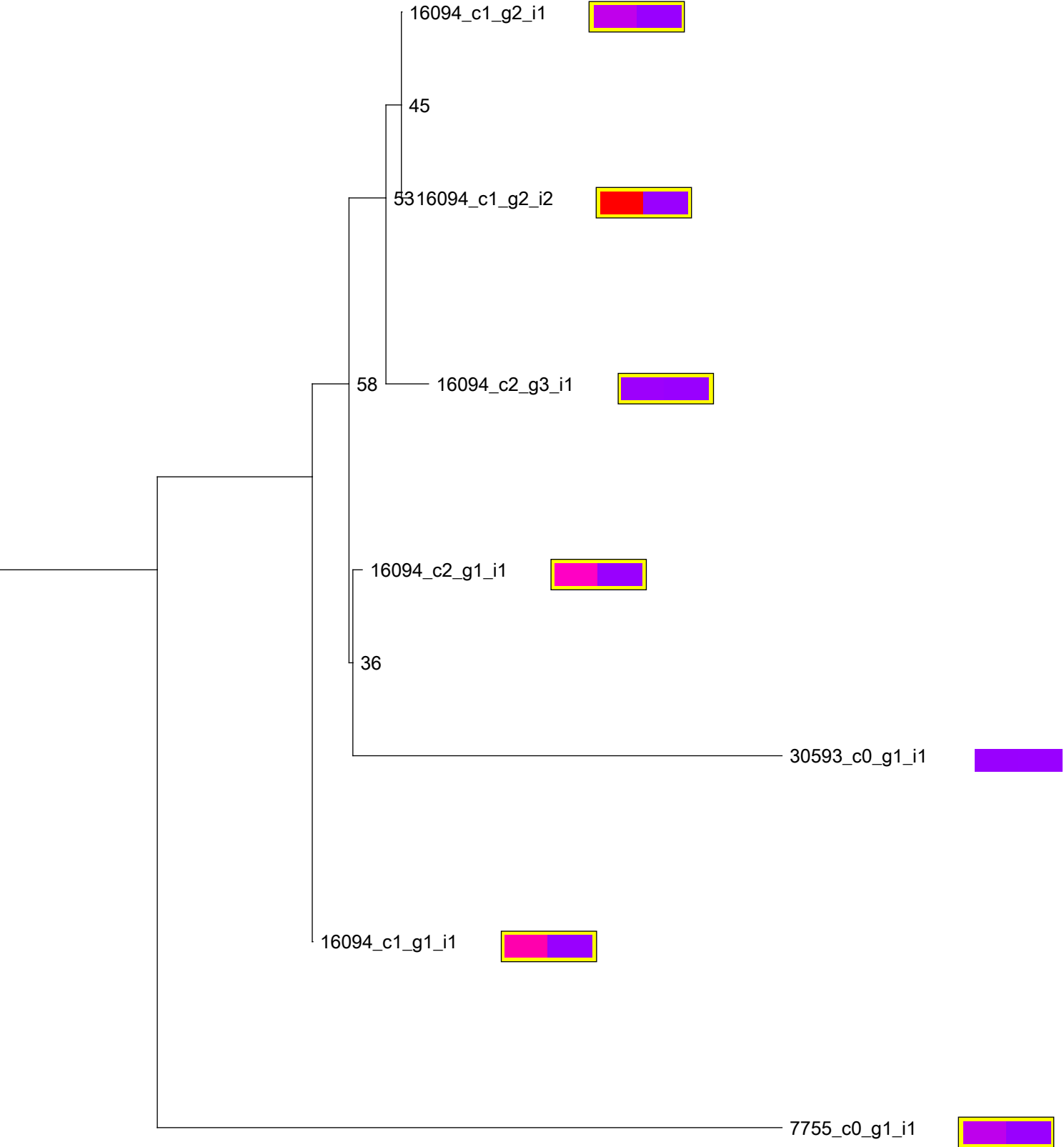

0.1

Min Max  
0 30412  
FPKM

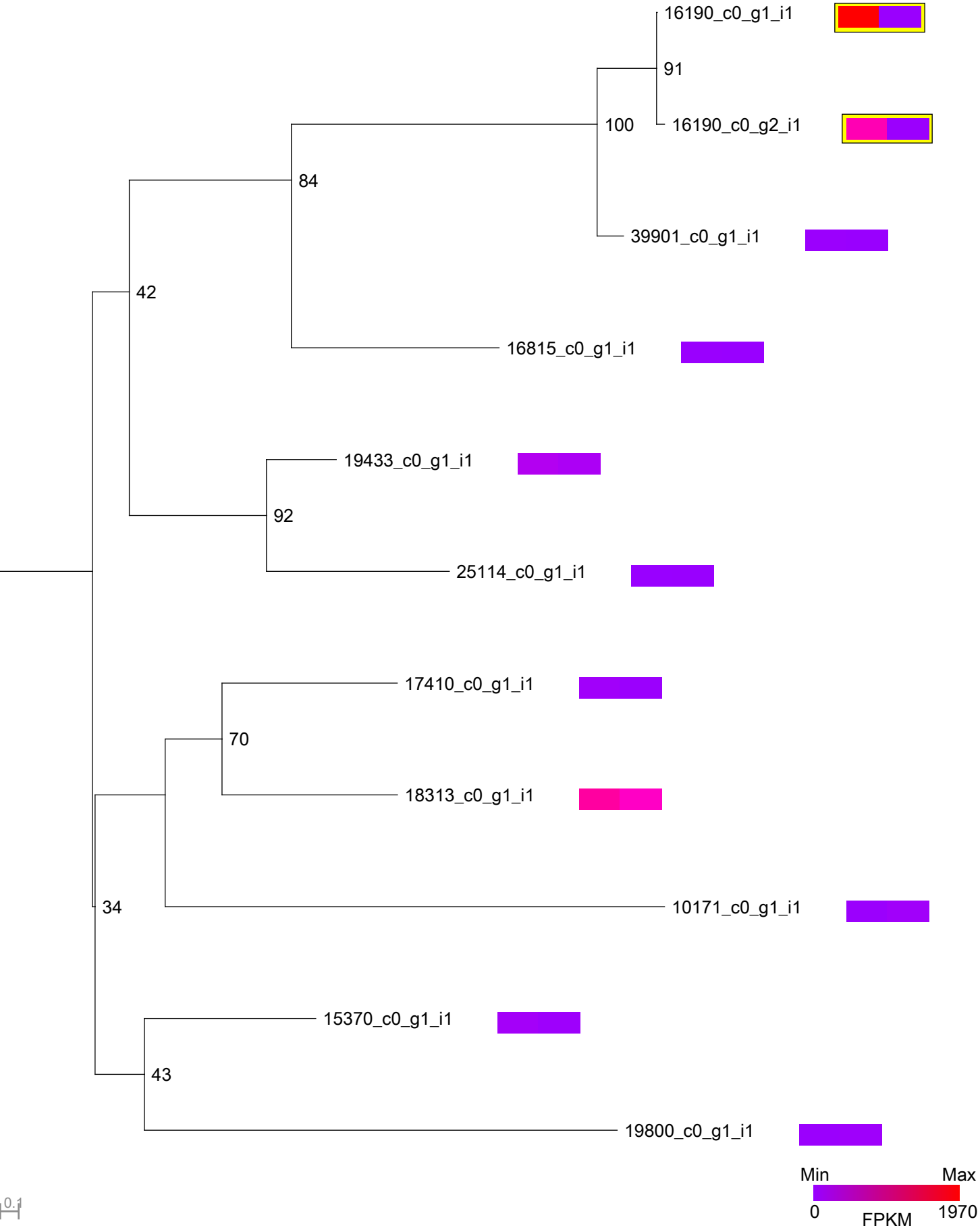

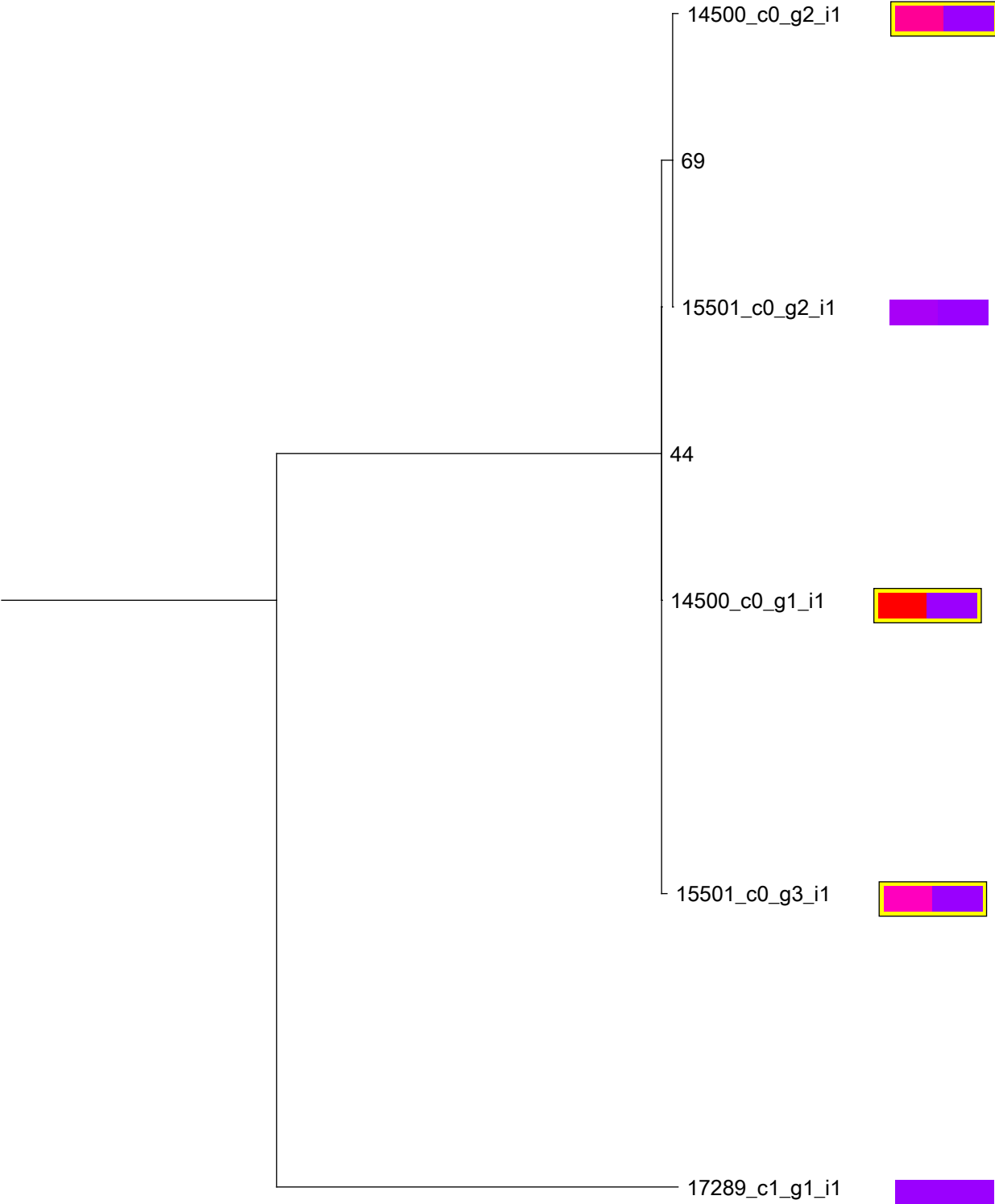

0.1

Min Max  
0 37386  
FPKM

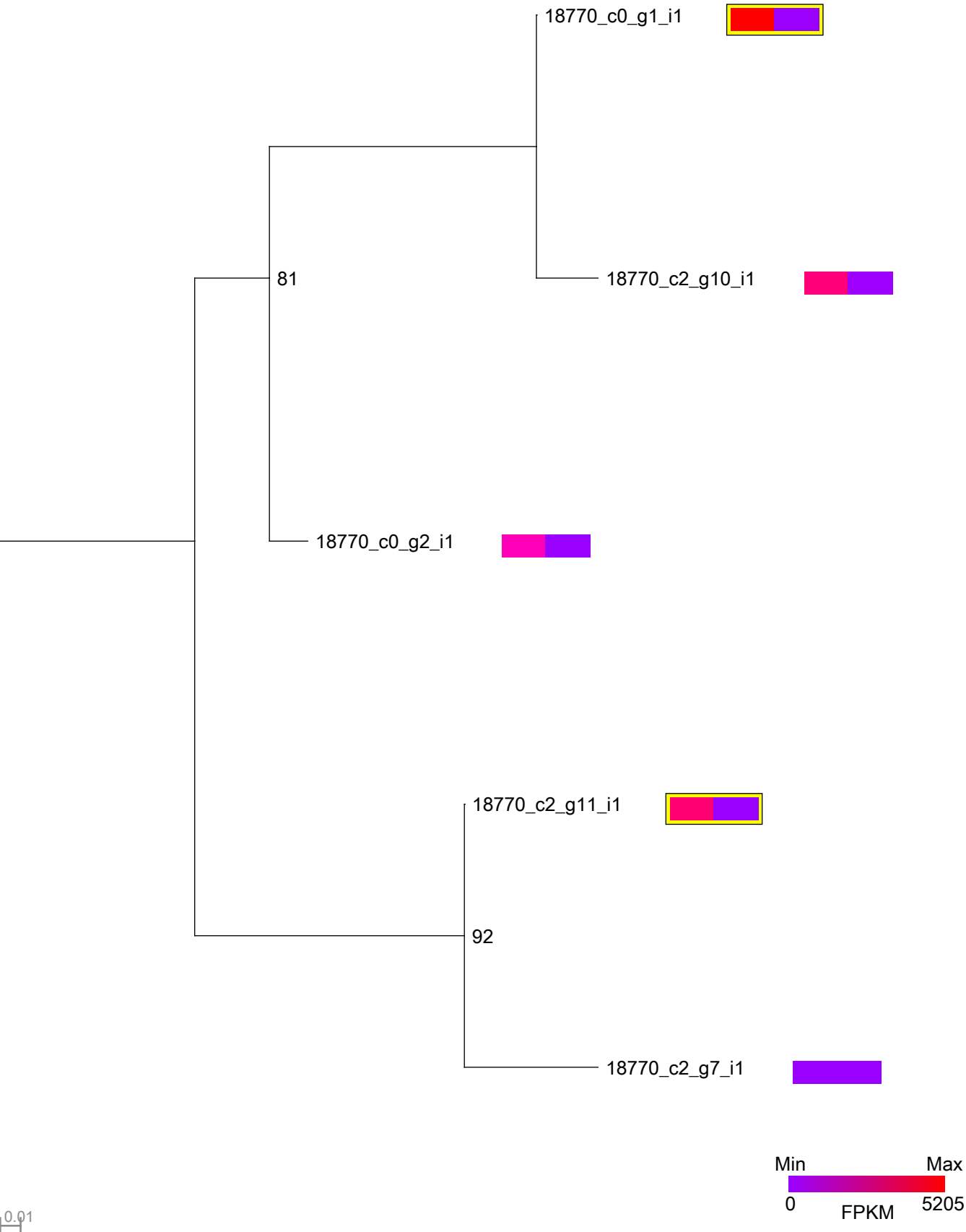

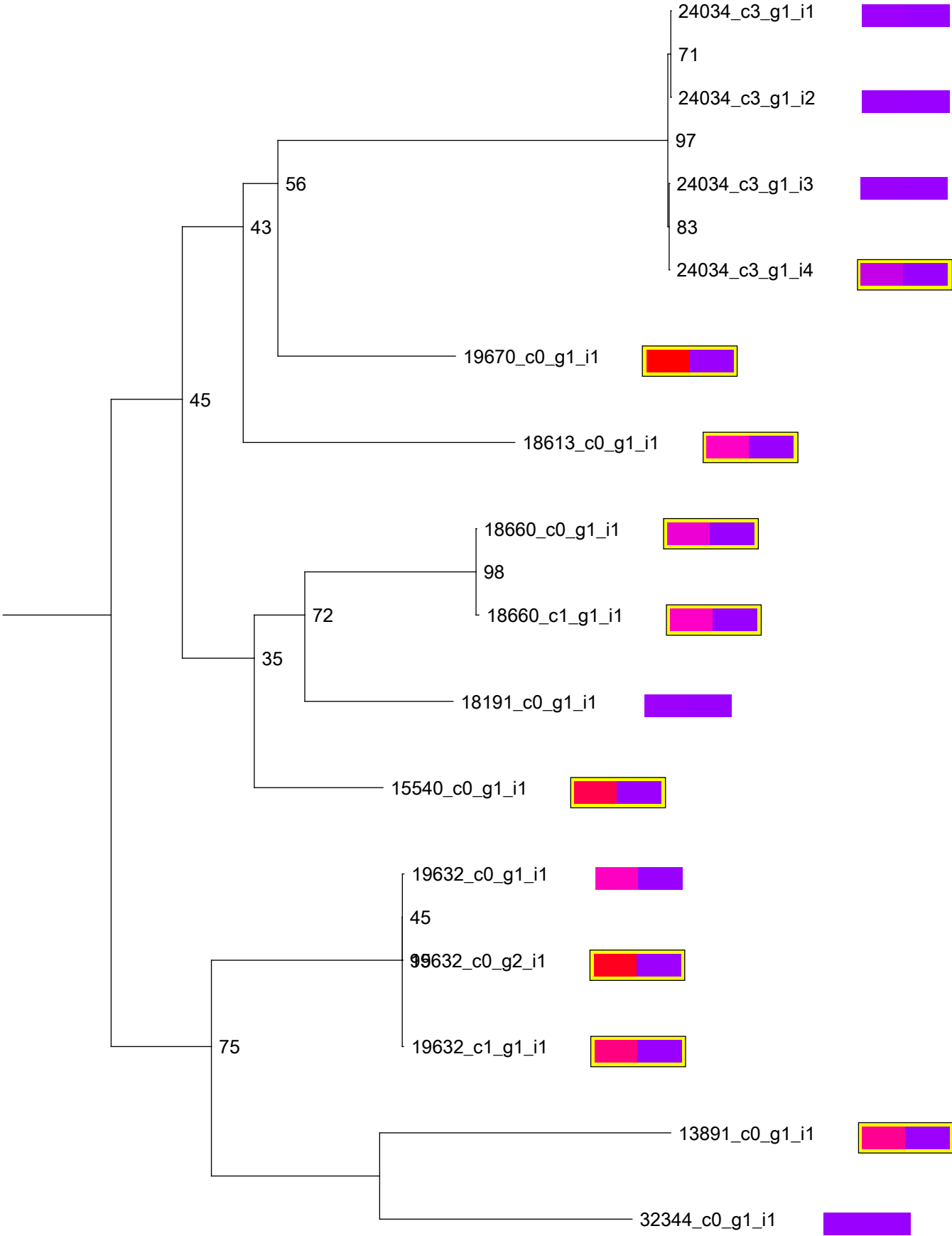

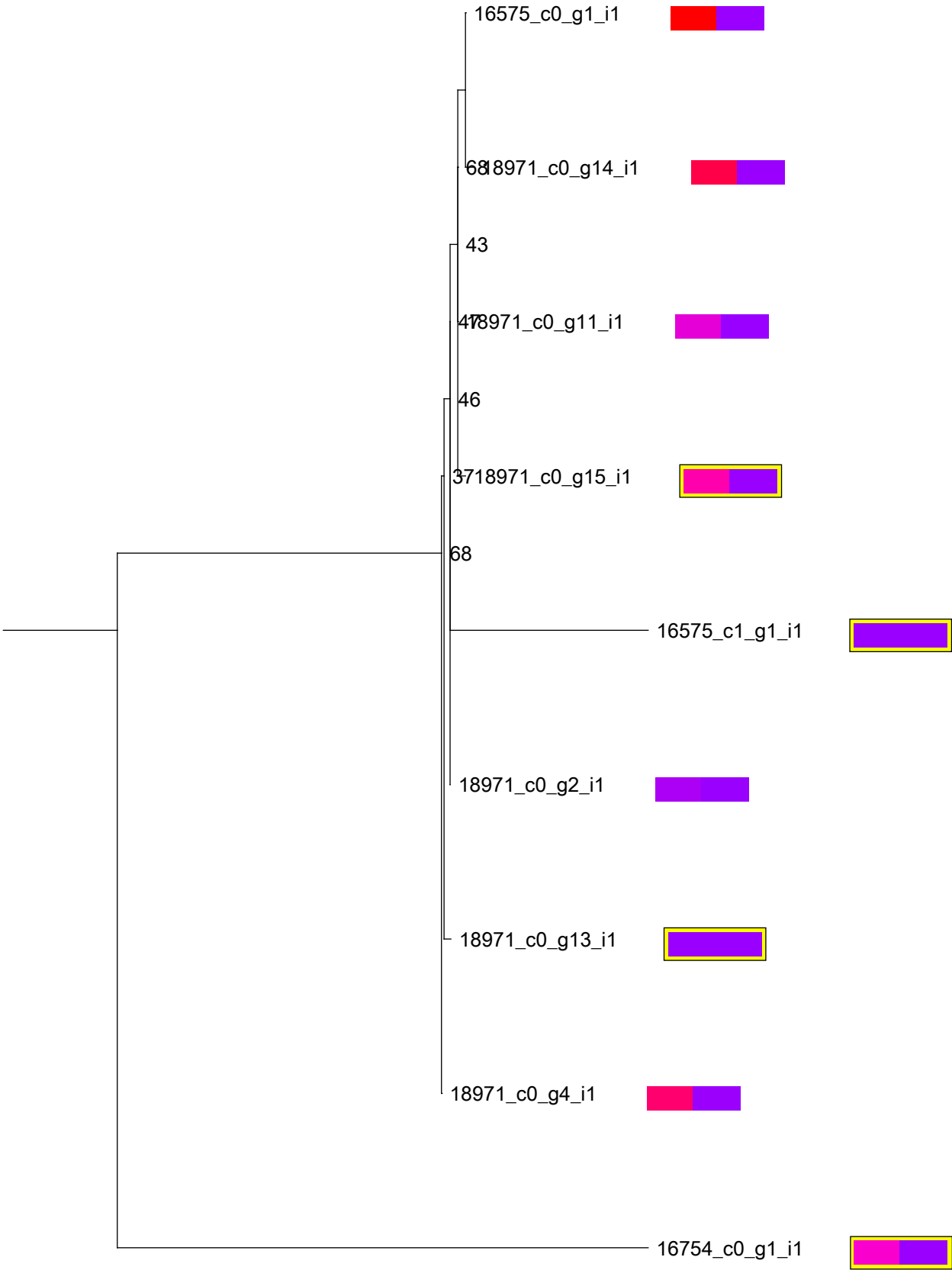

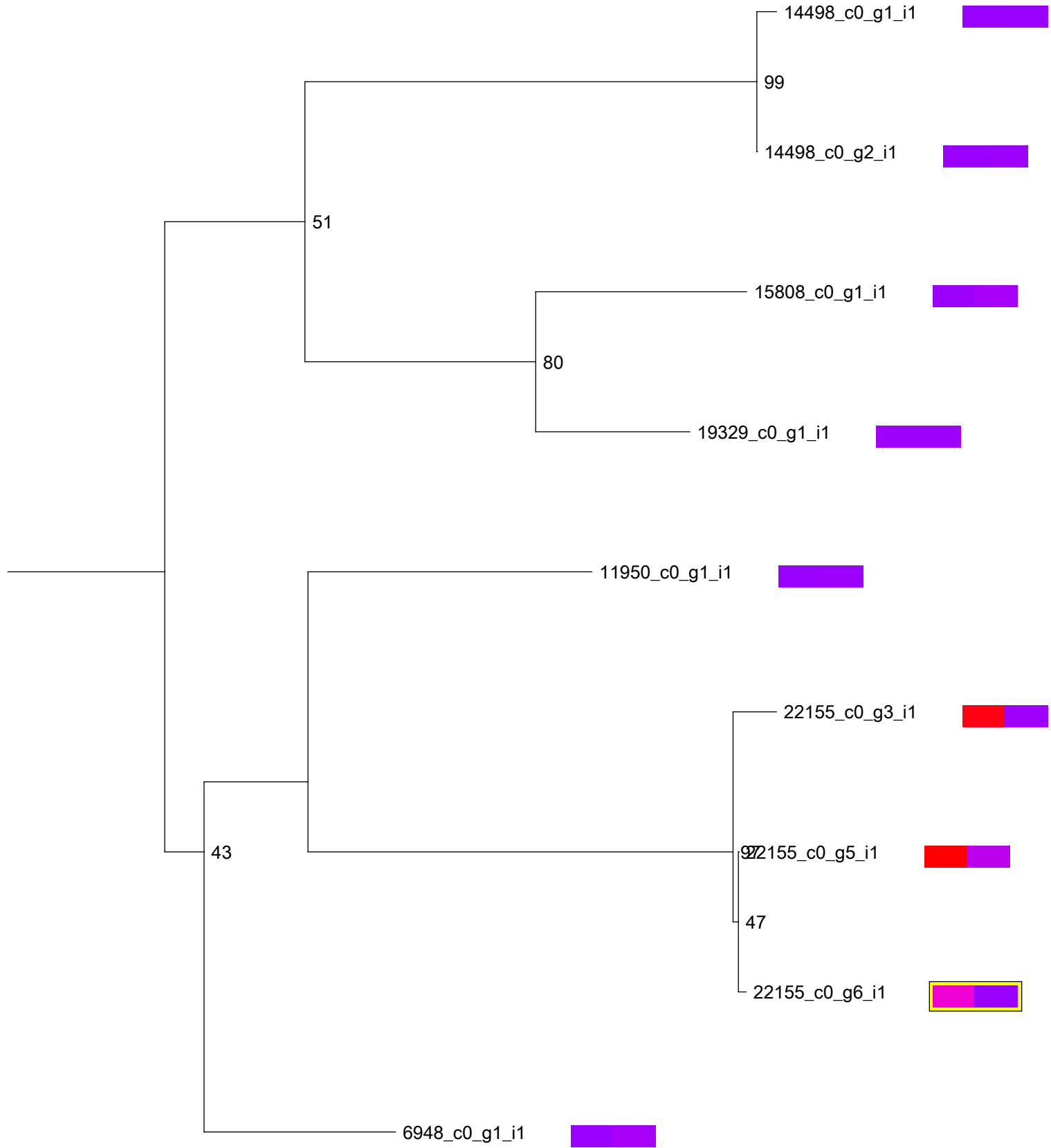

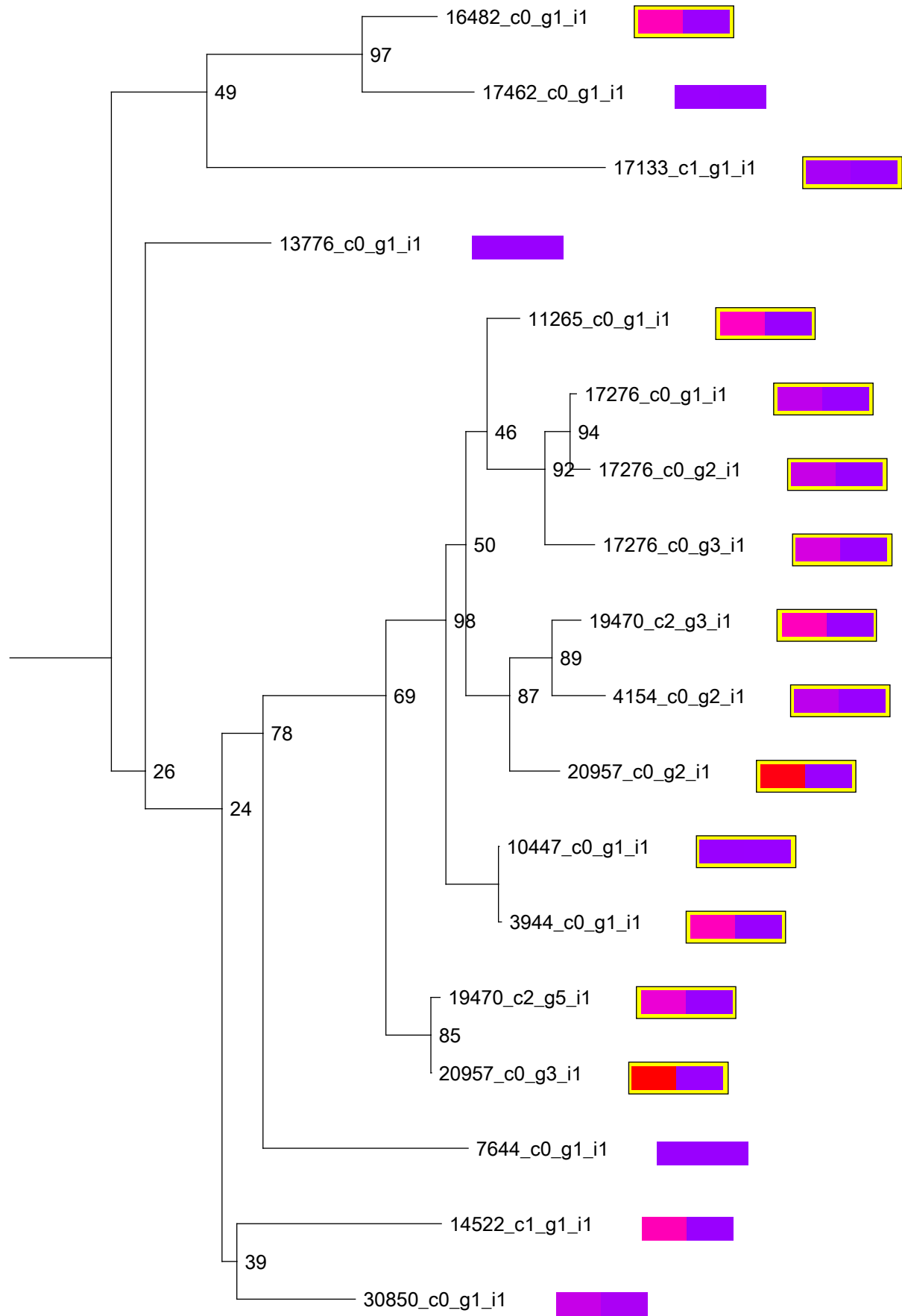

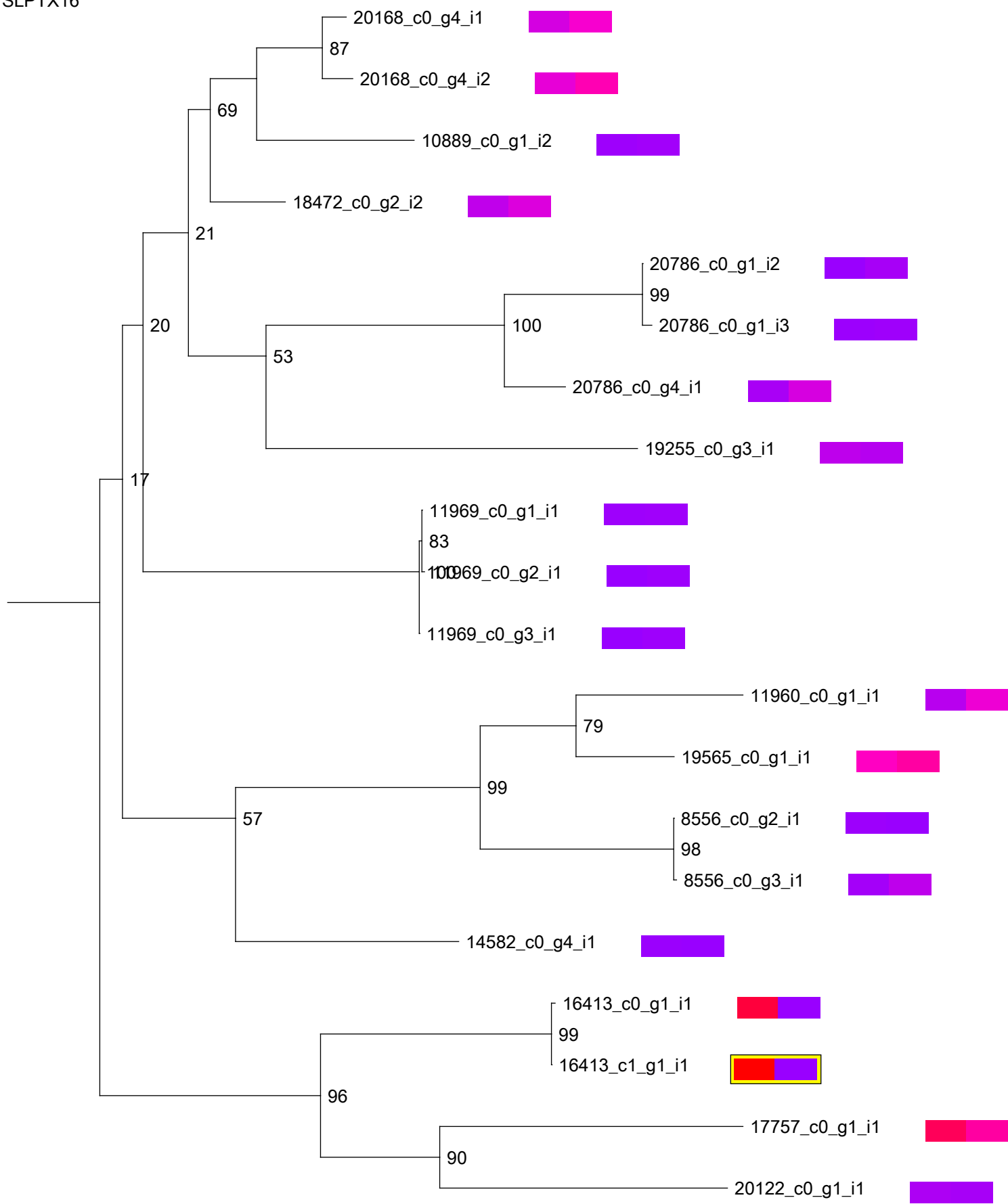

Transferrin

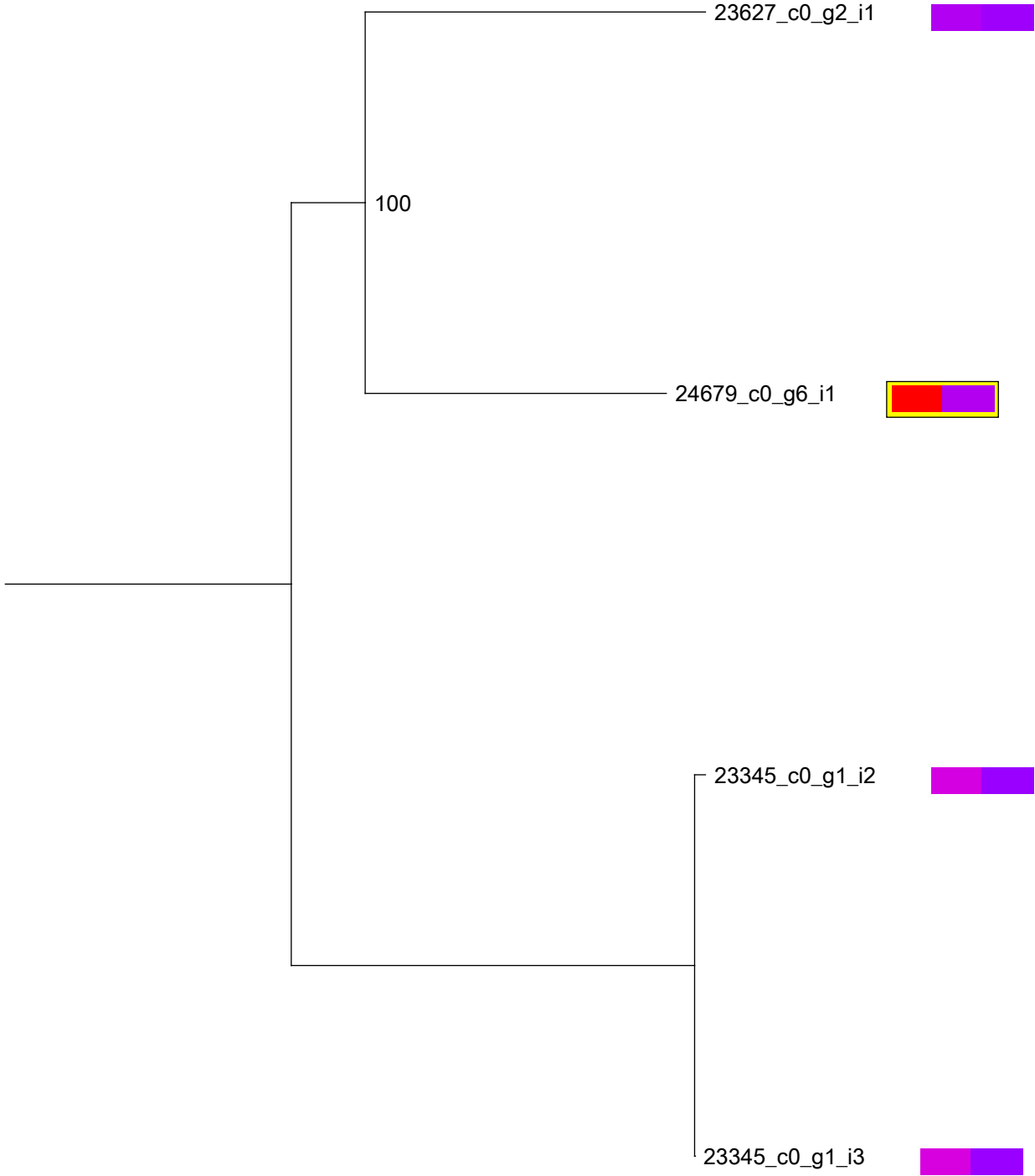

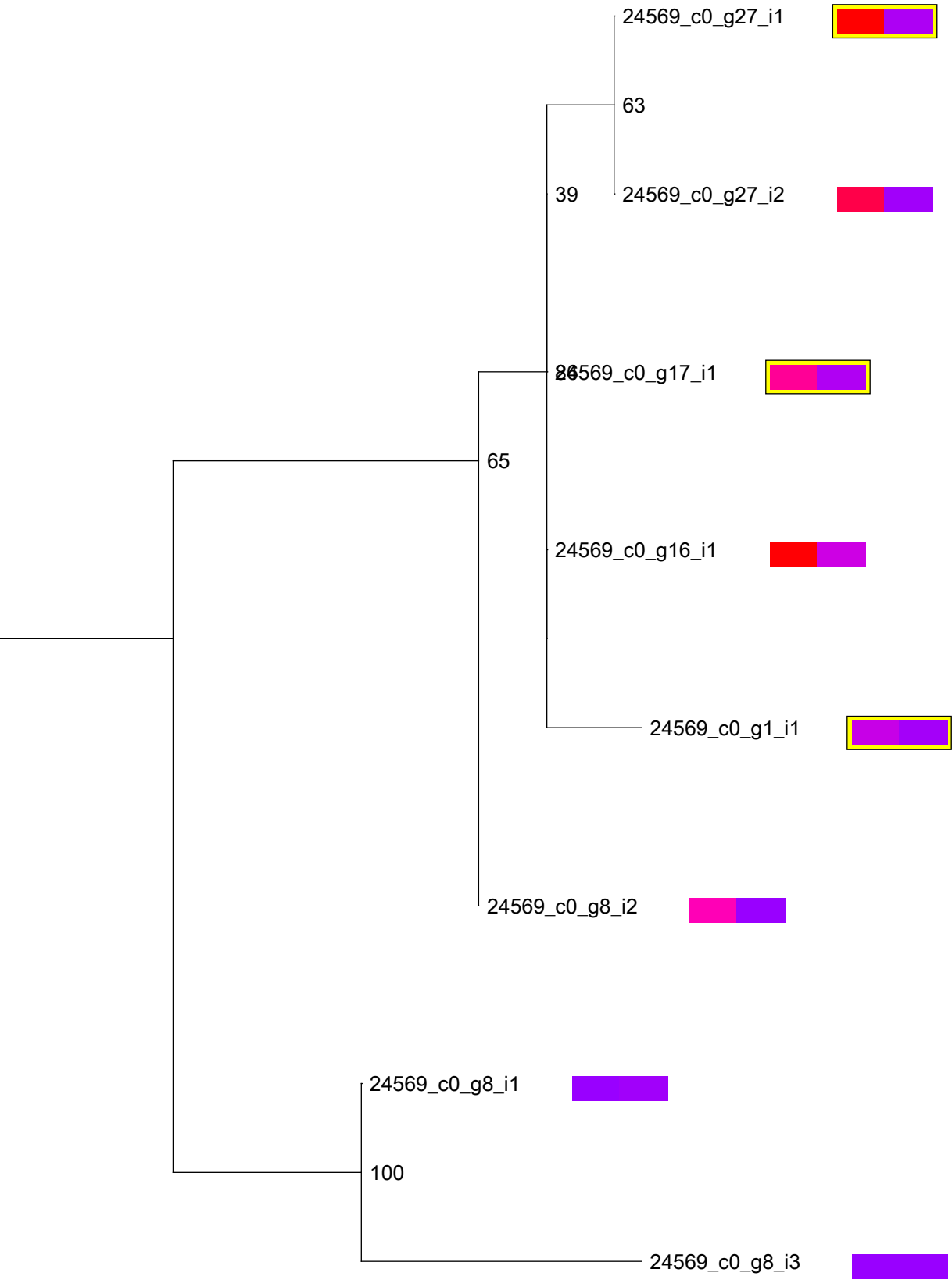

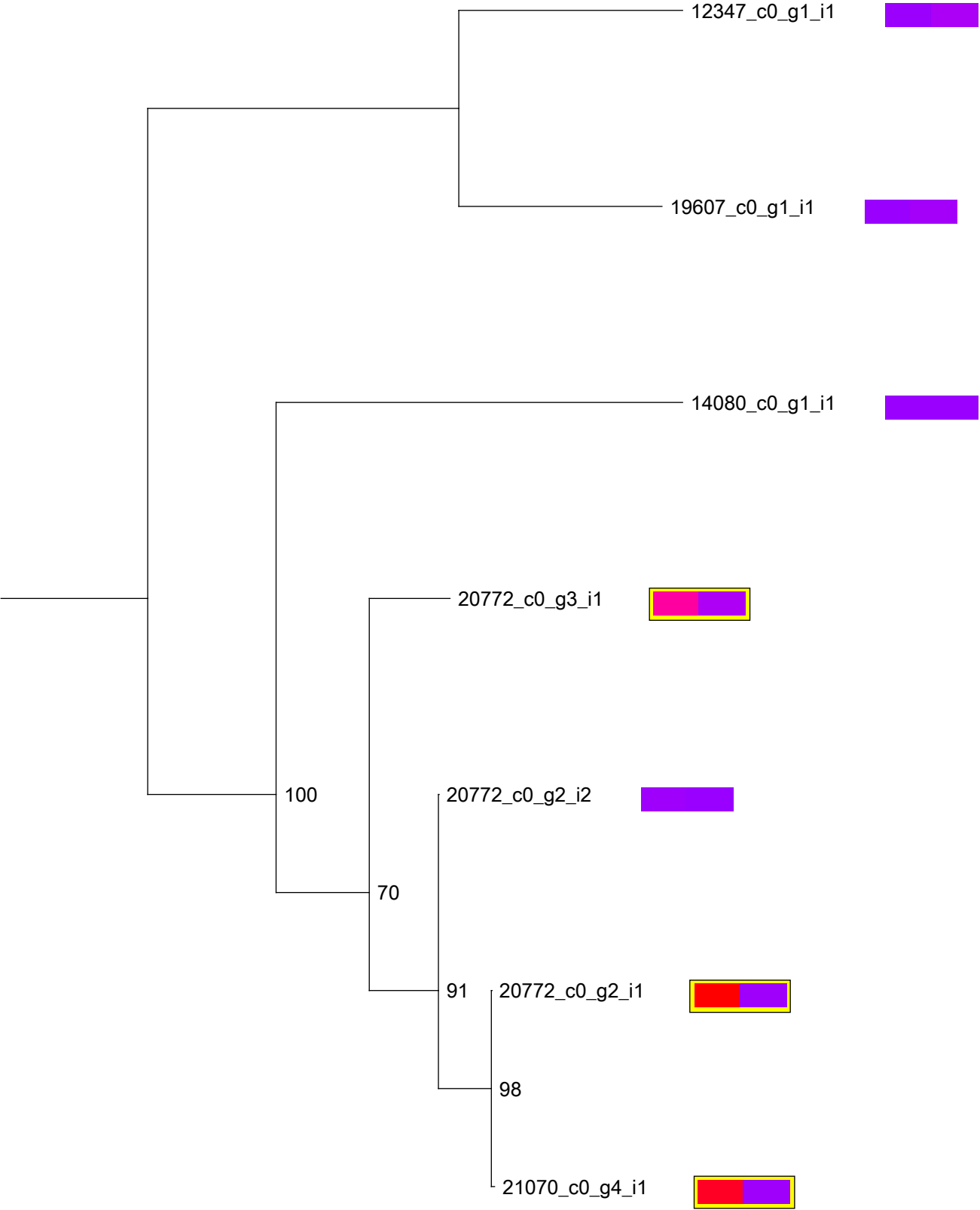

Supplement: Supplementary file 1 [file toxins-10-00096-s001.zip › Supplementary file 3.pdf]
